# Lipid-lowering drug targets influence inflammatory bowel disease through gut microbiota and inflammatory cytokines

**DOI:** 10.1016/j.jlr.2025.100871

**Published:** 2025-09-01

**Authors:** Xin Huang, Qihang Li, Ping Guo, Weiming Gong, Ying Wang, Zhongshang Yuan

**Affiliations:** 1 Key Laboratory of Endocrine Glucose & Lipids Metabolism and Brain Aging, Ministry of Education, Department of Neurology, Shandong Provincial Hospital Affiliated to Shandong First Medical University, Jinan, Shandong, China; 2 Shandong Institute of Brain Science and Brain-inspired Research, Medical Science and Technology Innovation Center, Shandong First Medical University & Shandong Academy of Medical Sciences, Jinan, Shandong, China; 3 Key Laboratory of Endocrine Glucose & Lipids Metabolism and Brain Aging, Ministry of Education, Department of Endocrinology, Shandong Provincial Hospital Affiliated to Shandong First Medical University, Jinan, Shandong, China; 4 Shandong Clinical Research Center of Diabetes and Metabolic Diseases, Jinan, Shandong, China; 5 Shandong Institute of Endocrine and Metabolic Diseases, Jinan, Shandong, China; 6 Department of Biostatistics, School of Public Health, Cheeloo College of Medicine, Shandong University, Jinan, Shandong, China; 7 Institute for Medical Dataology, Shandong University, Jinan, Shandong, China; 8 The Key Laboratory of Cardiovascular Remodeling and Function Research, Chinese Ministry of Education, Chinese National Health Commission and Chinese Academy of Medical Sciences, The State and Shandong Province Joint Key Laboratory of Translational Cardiovascular Medicine, Department of Cardiology, Qilu Hospital, Cheeloo College of Medicine, Shandong University, Jinan, China

**Keywords:** lipid-lowering drugs, inflammatory bowel disease, inflammatory cytokines, gut microbiota, drug target Mendelian randomization

## Abstract

Patients with dyslipidemia are at higher risk for inflammatory bowel disease (IBD), yet the impact of lipid-lowering medications on IBD remains unclear. This study investigates the causal relationship between lipid-lowering drug target and IBD, with a focus on the roles of gut microbiota and inflammatory cytokines. Genetic variants associated with lipid-lowering drug targets were extracted from the Global Lipids Genetics Consortium, whereas summary statistics for IBD, Crohn's disease (CD), and ulcerative colitis were sourced from the International Inflammatory Bowel Disease Genetics Consortium. Drug-target Mendelian randomization analysis revealed that inhibiting angiopoietin-like protein 3 increased the risk of IBD and CD, whereas inhibition of apolipoprotein C-III (APOC3) heightened the risk of CD. Conversely, enhancement of LPL and LDL receptor reduced the risk of IBD and CD. Mediation analysis demonstrated that gut microbiota and inflammatory cytokines partially mediated these effects, with specific pathways such as *Lachnospiraceae FCS020* (17.26%) for APOC3 and *Clostridium sensu stricto 1* (20.12%) for LPL accounting for significant portions of the effects. These findings suggest that lipid-lowering drugs targeting angiopoietin-like protein 3 and APOC3 may increase the risk of IBD, whereas those targeting LPL and LDL receptor may reduce the risk. The results highlight potential for repurposing lipid-lowering drugs for IBD prevention and warrant future clinical trials to explore these targets further.

Inflammatory bowel disease (IBD), including Crohn's disease (CD) and ulcerative colitis (UC) (1), is a chronic immune-mediated gastrointestinal disorder affecting approximately 7 million people worldwide, imposing a significant burden on global public health (2, 3). So far, the causes of IBD remain incompletely understood, and it is thought to involve a complex interaction between genetic susceptibility and environmental factors (4, 5, 6). In addition, effective treatments for IBD are still limited, and new therapeutic strategies are urgently needed.

Blood lipid abnormalities are well known to be important risk factors for IBD and are closely associated with the severity of IBD (7). Lipid-lowering drugs, especially statins, have attracted increasing attention on their potential role in the management of IBD. Some studies suggest that statins may affect the onset and progression of IBD by regulating lipid levels. However, the findings remain controversial. For instance, a population-based case-control study in Sweden found that statin use was associated with a reduced risk of CD rather than UC (8), whereas another retrospective study reported no association between statin use and IBD (9). The inconsistent results can be attributed to some well-known limitations in observational studies, including the issue of unmeasured confounding factors and reverse causality.

Previous studies had shown that lipid-lowering drugs can promote gut homeostasis and inflammatory status (10, 11). Specifically, statins can lead to alterations in gut microbiota and gut metabolites, which may contribute to maintaining gut homeostasis (12, 13, 14). In addition, statins can reduce various proinflammatory cytokines, such as interleukin-18 (IL-18) and IFN-gamma, which play critical roles in the inflammatory process (15, 16). An imbalance in the gut microbiota and elevated inflammatory cytokines are known to contribute to the pathogenesis of IBD (17, 18, 19). In the treatment of IBD, anti-inflammatory drugs, such as immunosuppressants, work by reducing proinflammatory cytokines (19), and drugs targeting the gut microbiota have also shown positive effects (20), suggesting that lipid-lowering drugs could potentially offer a therapeutic strategy by modulating both the gut microbiota and inflammatory pathways.

However, while observational studies provide valuable insights into the association between lipid-lowering drugs and IBD, they are limited in their ability to establish causal relationships. Recently, drug-target Mendelian randomization (MR) has emerged as a powerful tool for understanding the causal effects of drug targets on health outcomes, using genetic data as proxies for drug exposure (21, 22). By selecting genetic variants as instrumental variables (IVs), drug target MR can infer the causal effect of a drug target gene on the outcome. In this context, the term "causal" refers to causality within the MR framework. Well-designed drug target MR analyses can help narrow down the list of potential experimental targets and provide evidence for target prioritization. Furthermore, previous studies have shown that drug target genes supported by genetic evidence often have a higher success rate in clinical trials (23).

In this study, we aimed to evaluate the repurposing of commonly used lipid-lowering drugs for the treatment of IBD, CD, or UC, followed by two-step mediation MR analysis to explore the potential mediating roles of gut microbiota and inflammatory cytokines. We selected commonly used lipid-lowering drugs, focusing on those whose primary pharmacological actions target low-density lipoprotein cholesterol (LDL-C), including HMG-CoA reductase (HMGCR; target of statins), Niemann-Pick C1-like protein 1 (NPC1L1; target of ezetimibe), proprotein convertase subtilisin/kexin type 9 (PCSK9; target of evolocumab), apolipoprotein B-100 (APOB; target of mipomersen), and ATP-binding cassette subfamily G members 5/8 (ABCG5/ABCG8; target of colesevelam). We also included triglyceride (TG)-lowering drugs, such as angiopoietin-like protein 3 (ANGPTL3; target of evinacumab), apolipoprotein C-III (APOC3; target of volanesorsen), peroxisome proliferator-activated receptor alpha (PPARA; target of fibrates), and microsomal triglyceride transfer protein large subunit (MTTP; target of lomitapide). In addition, we included two critical drug targets involved in both LDL-C and TG metabolism: LDL receptor (LDLR) (24) and lipoprotein lipase (LPL) (25). The findings could provide valuable insights into the use of lipid-lowering drugs for individuals at potential risk of IBD and help identify promising candidate drugs for IBD treatment in future clinical trials.

## Materials and methods

### Study design

**Fig. 1:**
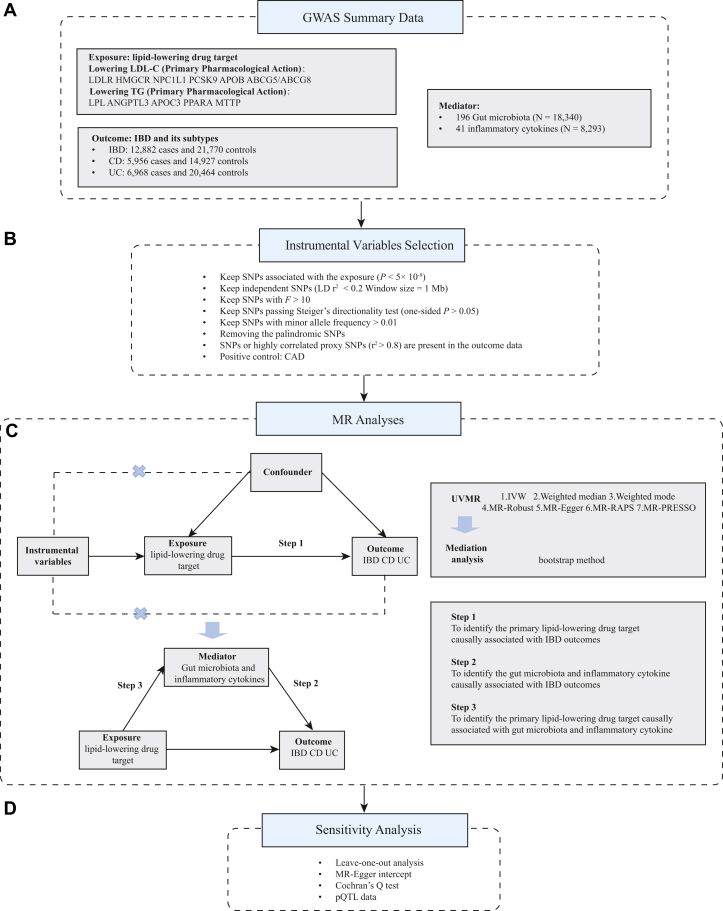
Schematic representation of the study design. We have first listed the data source for the exposure (lipid-lowering drug targets), mediating variables (196 gut microbiota and 41 inflammatory factors), and outcome variables (IBD, CD, and UC) in (A), followed by the rigorous selection procedure of IVs in (B). Then, we systematically explored the potential effects of lipid-lowering drug on IBD with drug target MR analyses and further investigate the possible mechanisms using two-step mediation MR analysis taking the gut microbiota and inflammatory factors as the mediators in (C), followed by the finally multiple sensitivity analysis to validate the robustness of the results in (D). IVW, inverse-variance weighted; LD, linkage disequilibrium; MR-RAPS, Robust Adjusted Profile Score.

Figure 1 provides a schematic of the study design. We first conducted drug-target MR to explore the potential causal associations between lipid-lowering drug targets and IBD, CD, and UC, followed by a two-step mediation MR analysis to examine the potential mediating role of 196 gut microbiota and 41 inflammatory cytokines in the effect of lipid-lowering drug target on these diseases. All analyses followed the STrengthening the Reporting of OBservational studies in Epidemiology using MR guidelines, as outlined in supplemental Table S1.

### Genetic variant selection

Common lipid-lowering drugs and novel therapeutics were selected based on recent guidelines for the management of dyslipidemia and ChEMBL databases (Table 1) (26, 27). We utilized drug substance information to identify pharmacologically active protein targets and obtain the corresponding target genes from DrugBank database (https://go.drugbank.com/) and relevant reviews (28, 29, 30). We focused on LDL-C and TG, which are commonly measured to assess the physiological response to these lipid-lowering drugs. We classified these drug target genes into two categories based on their own primary pharmacological actions: those involved in lowering LDL-C (including *LDLR*, *HMGCR*, *NPC1L1*, *PCSK9*, *APOB*, *ABCG5*, and *ABCG8*) and those associated with lowering TG (including *LPL*, *ANGPTL3*, *APOC3, PPARA*, and *MTTP*). Genome-wide association study (GWAS) summary statistics for LDL-C and TG were obtained from the Global Lipids Genetics Consortium, which includes approximately 1.3 million individuals of European ancestry, and we extracted genetic variants associated with these biomarkers (31).

**Table 1:** Classes of lipid-lowering drugs and target genes

Primary pharmacological action	Examples of drugs	Drug targets	General function	Gene encoding target	Gene region
Reduced LDL-C					
	Statins	HMG-CoA reductase	Catalyzes the conversion of HMG-CoA to mevalonic acid, the rate-limiting step in the synthesis of cholesterol and other isoprenoids. It plays a critical role in cellular cholesterol homeostasis and is a key target in managing cholesterol levels	*HMGCR*	chr5: 74632154–74657929
	Ezetimibe	Niemann-Pick C1-like protein 1	Involved in cholesterol homeostasis, NPC1L1 facilitates the absorption of cholesterol from the intestine into enterocytes and participates in plant sterol absorption, although it transports sitosterol at lower rates than cholesterol. It is crucial for maintaining cholesterol levels in the body	*NPC1L1*	chr7: 44552134–44580914
	Evolocumab	Proprotein convertase subtilisin/kexin type 9	Plays a crucial role in regulating plasma cholesterol levels by binding to LDLRs, including those for VLDLR, apolipoprotein E receptors (ApoE-R), and Apo receptor 2. It promotes the degradation of these receptors in intracellular acidic compartments, reducing cholesterol uptake	*PCSK9*	chr1: 55505221–55530525
	Mipomersen	Apolipoprotein B-100	A major protein component of LDL and VLDL particles, ApoB-100 is essential for the recognition and binding of these lipoproteins to the ApoB/E receptor, facilitating their internalization and the delivery of cholesterol to cells	*APOB*	chr2: 21224301–21266945
	Colesevelam	ATP Binding Cassette Subfamily G Member 5/ATP Binding Cassette Subfamily G Member 8	In the intestine, ABCG5 and ABCG8 transport plant sterols and cholesterol out of enterocytes into the intestinal lumen, limiting cholesterol absorption and reducing the intake of dietary plant sterols. In the liver, these transporters facilitate the movement of sterols into bile for excretion, thereby promoting cholesterol clearance	*ABCG5/ABCG8*^a^	chr2: 44039611–44066004/chr2: 44066103–44105605
	Key regulator	LDL Receptor^b^	Found in the liver and many tissues, the LDLR binds to ApoB-100 and ApoE on LDL particles, enabling their endocytosis. This process leads to the degradation of LDL particles in lysosomes, releasing cholesterol that is used by cells or stored for future use	*LDLR*	chr19: 11200038–11244492
Reduced TG					
	Evinacumab	Aangiopoietin-like protein 3	Acts as a hepatokine involved in lipid and glucose metabolism regulation. It increases plasma TGs by inhibiting LPL activity, thus suppressing TG clearance from the bloodstream and leading to elevated TG levels	*ANGPTL3*	chr1: 63063158–63071830
	Volanesorsen	Apolipoprotein C-III	ApoC-III is a key component of TG-rich lipoproteins, including VLDL and HDL. It plays a critical role in regulating TG metabolism, specifically by modulating lipoprotein clearance and lipid homeostasis. Targeting ApoC-III mRNA can reduce TG levels and improve lipid profiles	*APOC3*	chr11: 116700422–116703788
	Fibrates	Peroxisome proliferator-activated receptor-alpha	A ligand-activated transcription factor that regulates lipid metabolism. PPAR-α is activated by endogenous lipids, such as 1-palmitoyl-2-oleoyl-*sn*-glycerol-3-phosphocholine, and is involved in fatty acid oxidation and the regulation of lipid levels, thus influencing overall lipid homeostasis and metabolic health	*PPAR*A^c^	chr22: 46546424–46639653
	Lomitapide	Microsomal triglyceride transfer protein large subunit	Catalyzes the transfer of TGs, cholesteryl esters, and phospholipids between phospholipid surfaces, playing a key role in lipoprotein assembly and the movement of lipid molecules within cells	*MTTP*^c^	chr4: 100485287–100545154
	Key regulator	Lipoprotein Lipase^b^	LPL is a key enzyme in TG metabolism, responsible for hydrolyzing TGs from chylomicrons and VLDL, allowing for the clearance of lipids from the bloodstream. This process is essential for lipid utilization, storage, and overall metabolic health	*LPL*	chr8: 19796764–19824770

chr, chromosome.

a Due to the proximity of the genes encoding *ABCG5* and *ABCG8*, variants in the vicinity of these genes were combined in our analyses.

b LDL receptor and lipoprotein lipase are central players in LDL-C and TG metabolisms and are extensively involved in the lipid-lowering action.

c Since none of the variants survived through instrument construction, they were excluded from further evaluation.

Following previous studies (32), we selected genetic variants located near (±250 kb) or within the drug target genes as proxies for lipid-lowering drug classes, demonstrating associations with LDL-C or TG at a significance level of *P* = 5 × 10^−8^. Subsequently, we calculated the F-statistics for each SNP and retained only those variants with F-statistic >10 to avoid weak instrument bias (33). We further clumped these SNPs according to a lenient linkage disequilibrium threshold of *r*^2^ <0.2 and a physical distance of 10,000 kb using a reference dataset of the European ancestry from the 1000 Genomes Project. SNPs with minor allele frequency <0.01 were removed, and the forward strand alleles were inferred using allele frequency information when palindromic SNPs existed. We also applied MR Steiger filtering to remove those genetic variants that explained less variance in the exposure than the outcome, given that Steiger filtering was able to infer the causal direction for each SNP on the exposures and outcomes by calculating and comparing the variance explained by the genetic instruments (34).

Since none of the genetic variants of PPARA and MTTP were selected, we excluded them from further analysis. Due to the proximity of the genes encoding ABCG5 and ABCG8, variants in the vicinity of these genes were combined in our analyses. Finally, nine drug targets were included: HMGCR, NPC1L1, PCSK9, APOB, ABCG5/ABCG8, LDLR, ANGPTL3, APOC3, and LPL.

### Outcome data source

The GWAS summary statistics for the outcomes were derived from the International IBD Genetics Consortium (https://www.ibdgenetics.org/), including IBD (25,042 cases, 34,915 controls), UC (12,366 cases, 33,609 controls), and CD (12,194 cases, 28,072 controls) of European ancestry (35). All cases were diagnosed by accepted radiologic, endoscopic, and histopathologic evaluations in the International IBD Genetics Consortium. The genetic associations were adjusted for age, sex, and up to 20 genetic principal components.

The GWAS summary statistics for coronary artery disease (CAD), comprising data from 1,165,690 individuals of European ancestry, were obtained from Aragam *et al.* (36) and used as a positive control to validate the effectiveness of the IVs.

### Mediation data source

GWAS summary statistics for gut microbiota were obtained from a large-scale GWAS study by MiBioGen consortium (https://mibiogen.gcc.rug.nl), involving 18,340 participants from Europe, North America, and East Asia with 122,110 loci of variation (37). After excluding unknown bacterial taxa, gut microbiota was finally classified into 196 taxa, including 9 phyla, 16 classes, 20 orders, 32 families, and 119 genera. Furthermore, GWAS summary statistics for 41 inflammatory cytokines were extracted from a GWAS study involving 8,293 Finnish individuals (38), with combining results from The Cardiovascular Risk in Young Finns Study and FINRISK surveys. The details are provided in supplemental Table S2.

### Statistical analysis

#### Drug-target MR analysis

First, a positive control analysis was conducted to validate the effectiveness of genetic proxies for lipid-lowering drug target genes by investigating the association between genetically proxied lipid-lowering drug target genes and the risk of CAD, given the anticipated cardiovascular protective effect of lipid-lowering drugs.

In addition, we conducted drug-target MR analysis to assess the causal effect of 9 lipid-lowering drug target on IBD, CD, and UC. Specifically, we matched alleles for drug target IVs of exposure with that of outcome. If the variants were unavailable in the GWAS of outcomes, we searched for a proxy (*r*^2^ ≥ 0.8) for these SNPs, and SNPs without any proxies were further removed. Ambiguous SNPs with palindromic genotypes and minor allele frequencies between 0.4 and 0.5 were excluded from the analysis. We used inverse-variance weighted method (39) as primary MR analysis to obtain the causal effect estimates.

To validate the significant causal effects of drug target genes on IBD, CD, and UC in the primary analyses, we further conducted drug-target MR analysis using cis-protein quantitative trait loci (pQTL) from the deCODE study to corroborate our findings. The pQTL data were obtained from a large-scale GWAS on blood proteome, involving 27 million variant sites and 4,907 plasma protein SOMAmers, with 35,559 Icelanders participating in the deCODE Genetics research (40). We selected IVs located within ±500 kb of the drug target genes with significant associations with IBD in the primary analyses, employing the same stringent criteria as above.

#### Mediation MR analysis

To investigate potential mediating factors between lipid-lowering drug target genes and disease outcomes (IBD, CD, and UC), we performed a two-step MR analysis to quantify the effects of lipid-lowering drug target genes on disease outcomes via the gut microbiome and inflammatory cytokines. Briefly, in the first step, we employed a two-sample MR approach to assess the causal relationships between the gut microbiome and IBD, CD, and UC, as well as between inflammatory cytokines and these diseases. We set a looser threshold *P* value of 1 × 10^−5^ to maximize the number of IVs for each gut microbiome and cytokine, and the gut microbiota and cytokines with significant causal effects on IBD, CD, and UC were subjected to the second step. In the second step, we further examine the causal effects of lipid-lowering drug target genes on the gut microbiota or inflammatory cytokines that were significantly associated with disease outcomes in the first step. The mediating proportion of each inflammatory marker and gut microbiota between lipid-lowering drug target genes and IBD, CD, and UC was also calculated, with the 95% confidence intervals (CIs) for the mediation effects estimated using the Bootstrap method.

#### Sensitivity analysis

Multiple MR methods under different model assumptions were used to evaluate the robustness and reliability of the findings, including *1*) weighted median method, which could obtain consistent estimates if at least 50% of the IVs are valid (41); *2*) weighted mode method, which could provide an unbiased estimate if the SNPs contributing to the largest cluster are valid (42); *3*) inverse-variance weighted method using robust regression (MR-Robust), which could reduce the standard error of estimates (43); *4*) MR-Egger, which could detect the bias due to directional pleiotropy, with the intercept indicating the presence of directional pleiotropic bias (44); *5*) MR-Robust Adjusted Profile Score, which was robust to both systematic and idiosyncratic pleiotropy; and *6*) Mendelian Randomization Pleiotropy RESidual Sum and Outlier (MR-PRESSO), which could identify horizontal pleiotropic outliers and correct for pleiotropy via outlier removal (45). Furthermore, we assessed the heterogeneity of the estimates by applying Cochran’s Q test (46), leave-one-out analysis, and whether the MR-Egger intercept (44) is significantly deviated from zero. Besides MR-Egger intercept test, we further conducted a phenome-wide association study (PheWAS) analysis at the SNP level to assess the potential pleiotropy for those drug target genes significantly associated with IBD, we using 1,419 binary phenotypes from the PheWAS portal database (http://pheweb.sph.umich.edu/SAIGE-UKB).

We performed MR analysis using R packages “MendelianRandomization” (47), “MRPRESSO” (45), and “mr.raps.” All estimates (odds ratios [ORs] and effect estimates [beta value]) were scaled to 1 mmol/l decrease in LDL-C or TG. The statistical significance was set to be *P* < 0.05. Given that Bonferroni correction for multiple nonindependent tests may be too stringent, we used the false discovery rate at 0.05 based on Benjamini-Hochberg method for multiple testing correction, denoted as *P*-adjusted.

## Results

### Genetic IV selection and validation

We identified a total of nine drug target and selected instrumental SNPs within or near drug target genes as proxies (Table 1). Through a rigorous instrument screening procedure, we obtained a total of 111 proxy SNPs, with details provided in supplemental TableS3. The *F-*statistics for all selected instrumental SNPs were greater than 10, indicating the analyses were less likely to suffer from weak instrument bias. Briefly, 7 instrumental SNPs were selected for *ANGPTL3*, 21 for *APOC3*, 20 for *APOB*, 7 for *ABCG5*/*ABCG8*, 3 for *HMGCR*, 23 for *LPL*, 15 for *LDLR*, 2 for *NPC1L1*, and 13 for *PCSK9* (supplemental TableS3). In addition, we searched the GWAS Catalog (https://www.ebi.ac.uk/) and identified 1,145 IBD-associated genes (supplemental TableS4), which have no overlap with these lipid-lowering drug target genes. Indeed, the IBD-associated genes identified from GWAS are mainly obtained through the IBD-associated SNPs, which typically reflects statistical associations, rather than direct causal relationships or functional roles of the genes, and may not be directly involved in lipid metabolism or regulation. As expected, the genetic proxies for lipid-lowering drug target genes demonstrated a protective effect on CAD, suggesting the validity of the IVs (supplemental Fig. S1).

### Identification of lipid-lowering drug target causally associated with IBD, CD, and UC

**Fig. 2:**
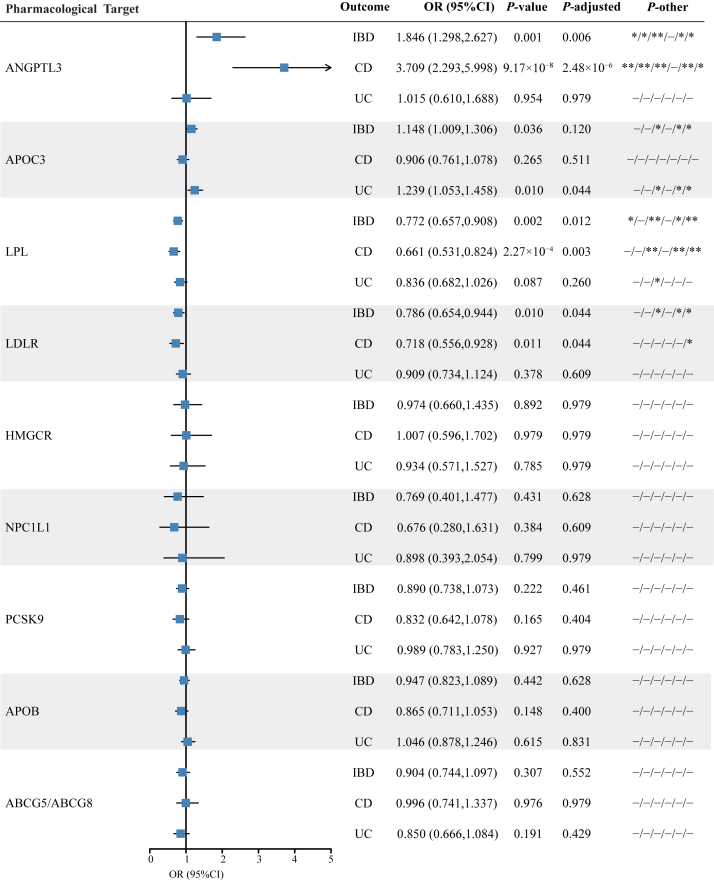
The causal effect estimates of lipid-lowering drug target on risk of IBD, CD, and UC from drug target MR analysis. The forest plots showed the causal effect of lipid-lowering drug target on IBD risk per a 1 mmol/l (LDL-C, 38.7 mg/dl; TG, 88.9 mg/dl) change in the lipid levels. Data are represented as ORs with 95% CI. OR <1.00 suggests a decreased risk associated with lipid-lowering drug targets. *P* values are derived from the inverse-variance weighted MR method. *P*-adjusted values are false discovery rate-adjusted *P* values obtained using the Benjamini-Hochberg procedure to control the false discovery rate across multiple comparisons. The *P*-other column summarizes the significance of results from other MR methods. In this column: ∗ indicates a *P* < 0.05. ∗∗ indicates a *P* < 0.01. ∗∗∗ indicates a *P* < 0.001. - indicates no statistical significance. The methods included in the *P*-other column, in order, are weighted median, weighted mode, MR-Robust, MR-Egger, MR-RAPS, and MR-PRESSO. IIBDGC, the International Inflammatory Bowel Disease Genetics Consortium.

Through comprehensive drug-target MR analysis, we investigated the causal associations between lipid-lowering drug target and the risk of IBD, CD, and UC. A total of four drug targets were identified to have significant causal associations with IBD, CD, or UC (Fig. 2 and supplemental TableS5). Specifically, genetically proxied ANGPTL3 inhibition demonstrated a significant association with an increased risk of IBD (OR = 1.846; *P*-adjusted = 0.006) and CD (OR = 3.709; *P*-adjusted = 2.48 × 10^−6^), respectively. In addition, genetically proxied APOC3 inhibition showed a significant association with a higher UC risk (OR = 1.239; *P*-adjusted = 0.044). In contrast, genetically proxied LPL enhancement was significantly associated with a lower risk of IBD and CD (OR = 0.772, *P*-adjusted = 0.012; OR = 0.661, *P*-adjusted = 0.003, respectively), so as genetically proxied LDLR enhancement (OR = 0.786, *P*-adjusted = 0.044; OR = 0.718, *P*-adjusted = 0.044, respectively). No significant causal associations can be found between other genetically proxied drug target and IBD, CD, or UC. In summary, various lipid-lowering drugs with different pharmacological mechanisms have the potential to exert distinct effects on IBD and its subtypes.

Importantly, the findings were further consistently found across multiple MR methods, including weighted median method, weighted mode method, MR-Robust, MR-Egger, MR-Robust Adjusted Profile Score, and MR-PRESSO (supplemental TableS5). Further analysis suggested that IVs of each lipid-lowering drug target genes had no horizontal pleiotropy (Egger intercept *P* > 0.05) and no heterogeneity (Cochran’s Q *P* > 0.05) (supplemental TableS6). In addition, there was no distortion in the leave-one-out plot for each lipid-lowering drug target genes, suggesting that no single SNP had a substantial impact on the MR estimate (supplemental Figs. S2–S5). For the IBD-associated drug target genes including ANGPTL3, APOC3, LDLR, and LPL, the PheWAS analysis at the SNP level (supplemental Figs. S6–S9) revealed three instrumental SNPs (rs4628268, rs1441771, and rs1534649) for LPL illustrated the potential pleiotropy and are significantly associated with swelling of limb (*P* = 1.3 × 10^−5^), other diseases of the teeth and supporting structures (*P* = 2.6 × 10^−5^), and disorders of refraction and accommodation; blindness and low vision (*P* = 2.0 × 10^−5^), respectively, so as one instrumental SNP (rs376642) for LDLR was associated with otorrhea (*P* = 4.5 × 10^−5^). Subsequently, we excluded these pleiotropic SNPs and reconducted the MR analysis for LPL and LDLR, where we found the results were highly consistent with the original analysis (supplemental TableS7), demonstrating the robustness of the findings against pleiotropy.

### Identification of gut microbiota and inflammatory cytokines causally associated with IBD, CD, and UC

In the first step of mediation MR analysis, we need to perform a two-sample MR analysis to assess the causal associations between 196 gut microbiota and 41 inflammatory cytokines with IBD, CD, and UC. Overall, significant associations can be found between 28 gut microbiota and IBD, CD, and UC (supplemental Tables S8–S10 and supplemental Fig. S10) as well as between nine inflammatory cytokines and IBD, CD, and UC (supplemental Tables S11–S13 and supplemental Fig. S11).

Thirteen of 28 gut microbiota exhibited potential risk effects, whereas 15 gut microbiota showed protective effects. In particularly, *Lachnospiraceae ND3007* (OR = 1.706, 95% CI: 1.131, 2.5742, *P* = 0.011), *Oxalobacter* (OR = 1.292, 95% CI: 1.126, 1.482, *P* = 2.50 × 10^−04^), and *Eubacterium eligens* (OR = 1.405, 95% CI: 1.058, 1.865, *P* = 0.019) demonstrated the strongest risk effects on IBD, CD, and UC, respectively. In contrast, *Clostridium sensu stricto 1* (*OR* = 0.765, 95% CI: 0.62, 0.942, *P* = 0.012), *Ruminococcaceae UCG009* (OR = 0.861, 95% CI: 0.772, 0.960, *P* = 0.007), and *Eubacterium ventriosum* (OR = 0.741, 95% CI: 0.552, 0.995, *P* = 0.046) exhibited the strongest protective effects on IBD, CD, and UC, respectively. Eight inflammatory cytokines showed potential risk effects, including the effect of genetically determined IL-10 on IBD (OR = 1.088, 95% CI: 1.013, 1.168, *P* = 0.032), the effect of macrophage inflammatory protein (MIP)-1a on CD (OR = 1.125, 95% CI: 1.022, 1.238, *P* = 0.016), and the effect of fibroblast growth factor basic on UC (OR = 1.133, 95% CI: 1.001, 1.282, *P* = 0.032). While only with IFN-gamma-induced protein 10 displayed protective effect on IBD (OR = 0.919, 95% CI: 0.851, 0.993, *P* = 0.032).

### Effect of lipid-lowering drug target on identified gut microbiota and inflammatory cytokines

In the second step of mediation MR analysis, we need to utilize drug-target MR to evaluated the causal effects of four outcome-related lipid-lowering drug targets on the 28 gut microbiota and 9 inflammatory cytokines identified to be associated with IBD, CD, and UC from the above first step. We found that the four lipid-lowering drug targets were associated with totally 15 gut microbiota and 8 inflammatory cytokines (supplemental Tables S14 and S15, supplemental Figs. S12, and S13). In particular, genetically proxied ANGPTL3 inhibition was associated with increased levels of the genus *Butyrivibrio* (*β* = 0.752; 95% CI: 0.225; 1.278, *P* = 0.005), so as the association between genetically proxied APOC3 inhibition and the genus *Lachnospiraceae FCS020* group (*β* = 0.102; 95% CI: 0.011, 0.193; *P* = 0.028), the association between genetically proxied LPL and the genus *Clostridium sensu stricto 1* (*β* = −0.240; 95% CI: −0.379, −0.100; *P* = 0.001), and the association between genetically proxied LDLR inhibition and the phylum Lentisphaerae (*β* = 0.371; 95% CI: 0.144, 0.599; *P* = 0.001).

Furthermore, genetically proxied APOC3 inhibition was associated with increased levels of interleukin-2 receptor alpha (IL-2ra) (*β* = 0.245; 95% CI: 0.073, 0.416; *P* = 0.005). Genetically proxied LPL enhancement was associated with decreased levels of IL-18 (*β* = −0.331; 95% CI: −0.570, −0.092; *P* = 0.007), so as the association between genetically proxied LDLR inhibition and fibroblast growth factor basic levels (*β* = −0.220; 95% CI: −0.382, −0.058; *P* = 0.008).

### Mediation effect of lipid-lowering drug target on IBD, UC, and CD via gut microbiota and inflammatory cytokines

To evaluate the indirect effect of four lipid-lowering drug targets (ANGPTL3, APOC3, LPL, and LDLR) on IBD, CD, and UC through gut microbiota and inflammatory cytokines, we carried out a mediation analysis utilizing both the effect estimates from two-step MR and the total effect from primary MR analysis.

**Fig. 3:**
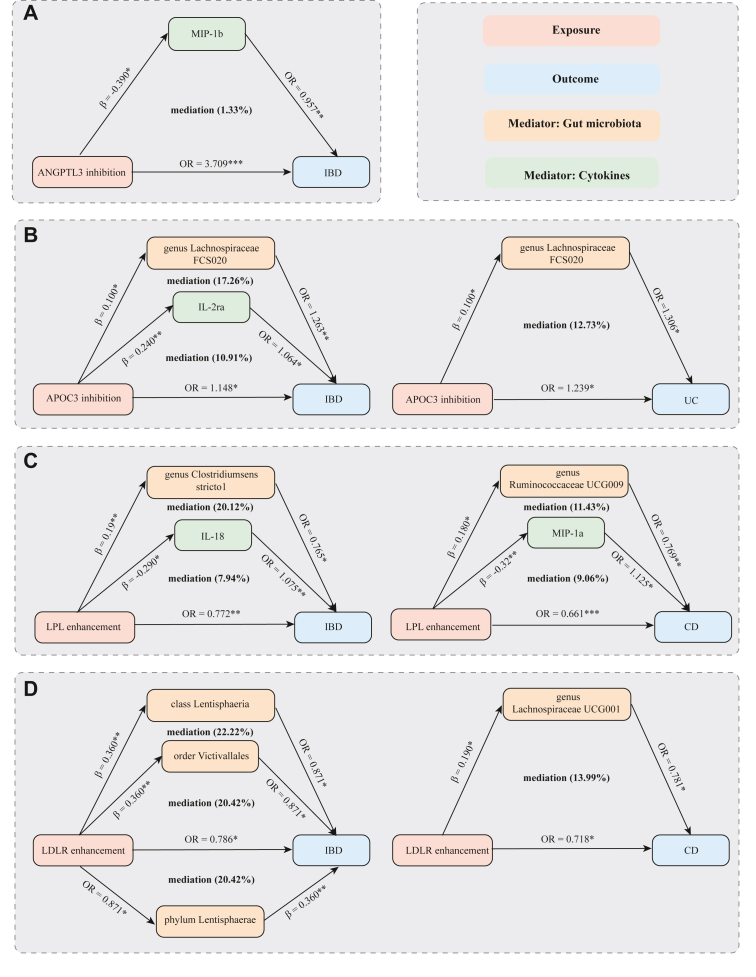
Mediation effects of lipid-lowering drug target on IBD, CD, and UC via gut microbiota and inflammatory cytokines. (A) Mediation effects of ANGPTL3 inhibition via cytokine MIP-1b on IBD. (B) Mediation effects of APOC3 inhibition via gut microbiota (genus Lachnospiraceae FCS020) and cytokine IL-2ra on IBD and UC. (C) Mediation effects of LPL enhancement via gut microbiota (genus Clostridiumsensu stricto1, genus Ruminococcaceae UCG009) and cytokine IL-18/MIP-1a on IBD and CD. (D) Mediation effects of LDLR enhancement via gut microbiota (class Lentisphaeria, order Victivallales, phylum Lentisphaerae, and genus Lachnospiraceae UCG001) on IBD and CD. IBD, inflammatory bowel disease; CD, Crohn’s disease; UC, ulcerative colitis; LDLR, LDL Receptor; ANGPTL3, angiopoietin-like protein 3; APOC3, apolipoprotein C-III; LPL, lipoprotein lipase; Interleukin-2 receptor antagonist, IL-2ra; macrophage inflammatory protein, MIP. ∗*P*-value < 0.05, ∗∗*P*-value < 0.01 and ∗∗∗*P*-value < 0.001.

The findings highlighted seven gut microbiota and four inflammatory cytokines as potential mediators in the association between lipid-lowering drug target and at least one of IBD, UC, and CD (Fig. 3 and supplemental TableS16). Specifically, the mediation effect proportions of APOC3 inhibition on the risk of IBD through *Lachnospiraceae FCS020* and IL-2ra were estimated to be 17.26% and 10.91%, respectively. The mediation effect proportion of APOC3 inhibition on the risk of UC through the genus *Lachnospiraceae FCS020* was estimated to be 12.73%. The mediation effect proportion of ANGPTL3 inhibition on CD through MIP-1b was estimated to be 1.33%.

The mediation effect proportions of LPL enhancement on protection from IBD through *Clostridium sensu stricto 1* and IL-18 were 20.12% and 7.94%, respectively. For CD, the mediation effect proportions of LPL enhancement through *Ruminococcaceae UCG009* and MIP-1α were 11.43% and 9.06%, respectively. The mediation effect proportions of LDLR enhancement on protection from IBD through Lentisphaeria, Victivallales, and Lentisphaerae were estimated to be 22.22%, 20.42%, and 20.42%, respectively. In addition, the mediation effect proportion of LDLR enhancement on protection from CD through *Lachnospiraceae UCG001* was 13.99%.

The mediation effect proportions of LPL enhancement on protection to IBD through *Clostridium sensu stricto 1* and IL-18 were found to be 20.12% and 7.94%, respectively. The mediation effect proportion of LPL enhancement on protection to CD through *Ruminococcaceae UCG009* and MIP-1a was 11.43% and 9.06%, respectively. The mediation effect proportion of LDLR enhancement on protection to IBD through Lentisphaeria, Victivallalesh, and Lentisphaerae was estimated to be 22.22%, 20.42%, and 20.42%, respectively. Furthermore, the mediation effect proportion of LDLR inhibition on protection against CD through *Lachnospiraceae UCG001* was 13.99%.

### Validation analysis

According to the same stringent IV selection criteria as above, we selected independent SNPs that were associated with drug target gene-encoded protein levels as IVs from the decoded pQTL summary data. Among these, only the cis-pQTLs of ANGPTL3 and APOC3 (i.e., genetic variants near the *ANGPTL3* and *APOC3* genes) yielded suitable IVs. Specifically, 24 independent SNPs were selected as IVs for ANGPTL3 and 19 for APOC3. Due to the unavailability of IVs, LPL and LDLR were excluded from the validation analysis. Again, the *F*-statistics for all the IVs were >10, indicating that the analyses were less likely to suffer from weak instrument bias.

The results revealed a causal association between lower level of ANGPTL3 protein and an increased risk of CD (OR = 1.270; 95% CI: 1.092, 1.477; *P* = 0.002). Similarly, lower level of APOC3 protein was causally associated with increased risk of UC (OR = 1.191; 95% CI: 1.011, 1.404; *P* = 0.036) (see supplemental Table S17 for details). The consistent results from the pQTL data in terms of the direction and magnitude of the causal effects further suggest the reliability of the findings (supplemental Table S17).

## Discussion

We conducted a comprehensive drug-target MR analysis to investigate potential causal effects of multiple lipid-lowering drug targets on IBD, CD, and UC, followed by two-step mediation MR analysis to explore the mediating roles of gut microbiota and inflammatory cytokines. We found the significantly causal associations between four lipid-lowering drug targets (ANGPTL3, APOC3, LPL, and LDLR) and the risk of IBD, CD, and UC. Moreover, we identified specific gut microbiota, such as *Lachnospiraceae FCS020* and *Ruminococcaceae UCG009*, and inflammatory cytokines, such as IL-18 and MIP-1b, as potential mediators in the causal effect of these lipid-lowering drug targets on IBD, CD, and UC. These findings not only provided novel insights into the off-target effects of lipid-lowering drugs but also indicated potential mechanisms underlying the impact of these lipid-lowering drug target genes on IBD, CD, and UC.

ANGPTL3, pivotal regulator of lipid transport, is a member of the angiopoietin-like protein family, which also includes ANGPTL4 and ANGPTL8. Although direct links between ANGPTL3 and IBD have not been extensively documented, recent studies have shown that the specific antisense oligonucleotide, vupanorsen, which inhibits ANGPTL3 synthesis, significantly increases hepatic steatosis (fatty liver). Hepatic steatosis has been suggested to be independently associated with an increased risk (48) of clinical relapse in UC and CD patients (49). In addition, another member of the ANGPTL family, ANGPTL4, has shown protective effect on IBD by downregulating CD8+ T-cell activity (50). Animal studies have also demonstrated that mice lacking *Angptl4* exhibited exacerbated colonic inflammation due to increased expression of colon chemokines (51). Our study first provides the genetic evidence supporting a causal association between ANGPTL3 inhibition and an increased risk of IBD and CD. We further found the mediating role of MIP-1b in the causal association between ANGPTL3 inhibition and CD, with the proportion of mediation effect found to be only 1%, suggesting that ANGPTL3 primarily exerts a direct effect on IBD and CD or that there may be additional unknown mediators that require further exploration.

APOC3, a significant lipoprotein synthesized in the liver and intestine, plays a crucial role in the assembly and secretion of TG-rich VLDL particles. A study involving 405 patients reported lower levels of circulating APOC3 in patients with IBD compared with the control group (52). However, due to the limited sample size and the inherent limitations of observational studies, inferring a direct causal association between APOC3 and IBD remains challenging. In our study, using genetic tools, we have uncovered compelling evidence supporting a causal association between APOC3 inhibition and an increased risk of IBD and UC. Furthermore, our investigation identified potential mediators such as *Lachnospiraceae FCS020* and IL-2ra. While studies on the associations between APOC3 and gut microbiota or inflammatory cytokines are insufficient, previous study has identified Lachnospiraceae and IL-2ra as risk factors for IBD and its subtypes (38, 53). A cohort study found that alterations in the Lachnospiraceae family of bacteria were associated with an increased risk of IBD, and disruptions in the microbial network of Lachnospiraceae and Ruminococcaceae were associated with poor treatment response and frequent relapse in IBD (53). In addition, a genome-wide meta-analysis study implicated IL-2ra in the risk of CD, with the IL-2ra-targeting antibody daclizumab demonstrating efficacy in multiple sclerosis, which is an autoimmune disease, and showed promising benefit for CD treatment (38). However, further clinical trials are imperative to validate its efficacy.

LDLR, a glycoprotein situated on the cell surface, takes place primarily in the liver and intestine, where it assumes a pivotal role in the formation and release of triglyceride-rich VLDL particles (54). Using *Ldlr* knockout mice, a study demonstrated that LDLR deficiency inhibited the activation, proliferation, and effector functions of CD8+ T cells. Primary experiments on CD8+ T cell showed that their reliance on LDLR-mediated lipid transport for proliferation and effector functions (55). While CD8+ T cells have been implicated in IBD pathogenesis, their role in IBD is multifaceted, exhibiting both immunosuppressive and proinflammatory effects (56, 57). Despite the insights from existing studies, investigating the underlying mechanisms by which LDLR influences IBD through its impact on inflammatory responses remain challenging. Our study provides causal evidence supporting the association between LDLR enhancement and a reduced risk of IBD. However, our findings only indicated mediation pathways through specific gut microbiota, including Lentisphaeria, Victivallales, and Lentisphaerae, without evidence of mediation effects through inflammatory cytokines. Lentisphaeria encompasses the Lentisphaerae class and Victivallales order. While evidence for impact of Lentisphaeria on IBD is lacking, studies have suggested the potential influence of Lentisphaeria on metabolism, insulin sensitivity, and hepatic fat content (58), all of which have potential implications for IBD (59, 60). Therefore, the mechanisms underlying the impact of LDLR on IBD require further exploration.

LPL plays a role in lipid distribution and metabolism by catalyzing the hydrolysis of TGs in circulating lipoproteins (61). Despite its significance in lipid metabolism, studies exploring the association between LPL and IBD remain limited. An age- and sex-matched case-control study reported elevated levels of circulating LPL in individuals with IBD compared with controls (62). In contrast, our study provides causal evidence supporting the protective effect of increased LPL expression on IBD and CD, and we identified potential mediators, such as genus *Clostridium sens stricto 1*, *Ruminococcaceae UCG009*, IL-18, and MIP-1a. While investigations on the associations between LPL and gut microbiota or inflammatory cytokines are lacking, previous studies have highlighted the role of IL-18 and MIP-1a as potent proinflammatory cytokines crucial in host defense and immune response regulation. Studies have documented increased levels of IL-18 (63) and MIP-1a (64) in serum and colonic tissues of mice with dextran sodium sulfate-induced colitis, further emphasizing their involvement in inflammatory processes. In addition, a study using an innovative multivariate metagenomic analysis approach analyzed the microbiota in colonic tissues and fecal samples from 196 IBD patients (121 CD patients, 75 UC patients) and 27 healthy individuals, revealing a decreased abundance of the Ruminococcaceae in CD patients (65). These findings further support our study and suggest that targeted regulation of LPL activity may be a potential strategy for treating intestinal inflammation and other inflammatory diseases.

Our results showed significant associations between several drug target genes and gut microbiota as well as inflammatory factors, suggesting that these drugs may act through a "microbiota-immune"-mediated pathway. Specifically, we found that lipid-lowering drug targets, such as ANGPTL3, APOC3, LPL, and LDLR, were closely associated with IBD risk, and their effects might be mediated indirectly through the modulation of specific gut microbiota (e.g., *Lachnospiraceae FCS020* genus, *Clostridium sensu stricto 1*, Lentisphaeria class, Victivallales order, Lentisphaerae phylum, and *Ruminococcaceae UCG009* genus) or immune factors (e.g., MIP-1β, IL-18, IL-12ra). In CD, we observed the significant effect of LPL and LDLR, with their potential mediating pathways involving systemic proinflammatory factors (e.g., MIP-1β) and gut microbiota (e.g., *Ruminococcaceae UCG009* genus and *Lachnospiraceae UCG001* genus). In UC, we found the significant effect of APOC3, with its mediating effect primarily driven by *Lachnospiraceae FCS020* genus, rather than inflammatory factors. The different results between IBD and CD may be related to the inflammatory distribution characteristics of CD. CD typically presents with transmural inflammation, which affects the entire intestinal wall (66), thus triggering more systemic immune responses. The immune factors such as MIP-1β were involved in the mediating pathway in CD, suggesting immune regulation may be an important mechanism through which lipid-lowering drug targets exert protective effects in CD. The different results between IBD and UC may be attributed to the localized mucosal inflammation observed in UC, in which the gut microbiota (67), rather than systemic immune factors, may present a more prominent role in disease progression.

This study has several strengths. First, we used the large-scale omics data, paralleled with a cutting-edge analysis pipeline, to investigate the impact of lipid-lowering drug target on IBD and sequentially explore the potential mediating roles of gut microbiota and inflammatory cytokines, providing novel insights into the repurposing of lipid-lowering drugs in treatment of IBD as well as future optimization of lipid-lowering drug therapies. Second, we used drug-target MR approach to mimic drug exposure utilizing genetic instruments, which could mitigate confounding bias, avoid reverse causation, and ascertain the causal association between drug exposure and diseases. Third, in addition to performing various sensitivity analyses to assess the robustness and the consistency of the results, we also used multiple MR methods with different model assumptions to evaluate the robustness of our findings and to avoid the risk of false discoveries due to the model misspecification of single MR method.

Our study is not without limitations. First, the drug-target MR focuses on evaluating the long-term impact of intervention on the drug target, which may differ from the outcome of drug interventions at specific time points or shorter durations. Although these discrepancies may impact the accurate estimation of clinical drug intervention effects, it still provides valuable insight into the presence and direction of causal effects. Second, drug-target MR analysis can offer valuable perspectives on drug effects within the same drug class or with similar targets; however, it may fail to provide specific information on individual drug compounds. Third, the drug effects can vary across different tissues and populations. It is crucial to utilize genetic association data from relevant tissues or diverse populations to further validate the findings. Last, the analysis based on protein expression is constrained by the limited sample size of available pQTL datasets. Furthermore, the analysis based on protein expression is constrained by the limited sample size of available pQTL datasets, and only two lipid-lowering drug targets (ANGPTL3 and APOC3) had suitable IVs in the current analysis. Expanding the sample size and including more drug targets in future pQTL studies would further strengthen the robustness of the results. Finally, although drug target MR analysis could be an ideal drug target screening tool, the wet-bench experimentation is still required for further verifications.

In conclusion, this study presents compelling evidence establishing causal associations between lipid-lowering drug target genes and IBD, shedding light on the mediating roles of gut microbiota and inflammatory cytokines. The results have implications for repurposing lipid-lowering medications in future IBD prevention and treatment strategies and for future clinical trials in IBD with comorbid hyperlipidemic.

## Data availability

The datasets analyzed in the current study can be downloaded from the websites https://mibiogen.gcc.rug.nl/, https://www.ibdgenetics.org/, https://gwas.mrcieu.ac.uk/, and https://www.ebi.ac.uk/gwas/downloads/summary-statistics.

## Conflict of interest

The authors declare that they have no conflicts of interest with the contents of this article.
