# Supplementary material for: Lipid-lowering drug targets influence inflammatory bowel disease through gut microbiota and inflammatory cytokines
Source: J Lipid Res. 2025 Sep 1;66(9):100871. doi: 10.1016/j.jlr.2025.100871 (PMC12423403; doi:10.1016/j.jlr.2025.100871)
Supplement: Supporting Information Figures [file mmc1.docx]

## Supplementary Figures

**
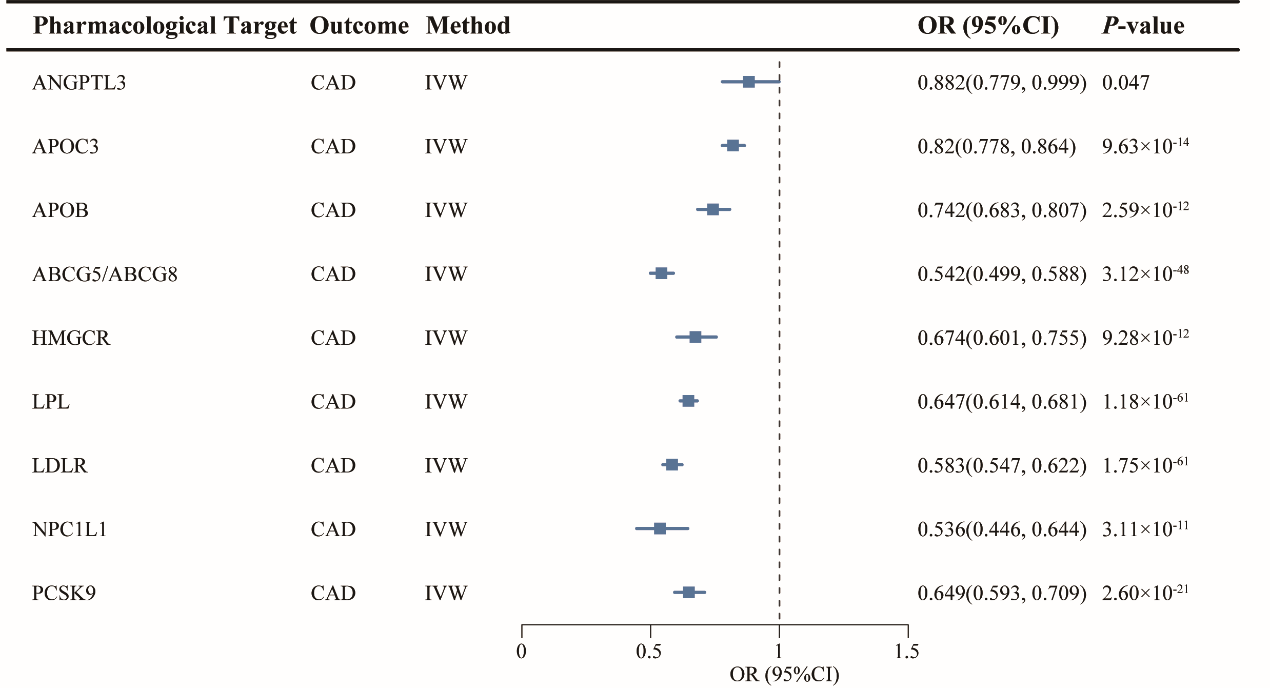
**

**Figure S1. MR estimates for the effect of lipid-lowering drug target on risk of CAD.**

CI, confidence interval; OR, odds ratio; IVW, inverse-variance weighted; MR, Mendelian

randomization; CAD, coronary artery disease.


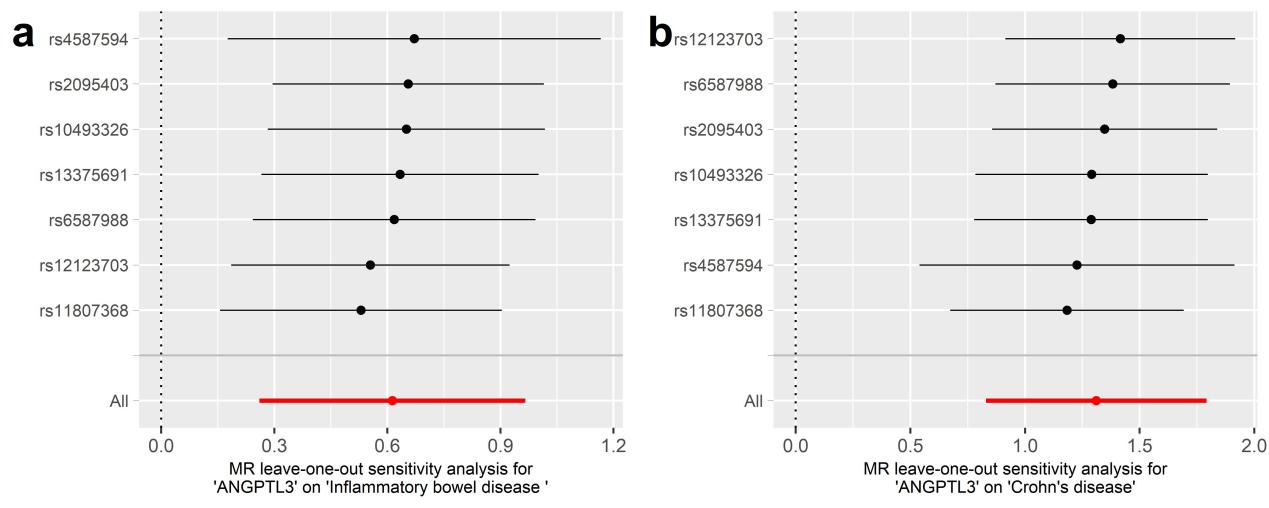


**Figure S2. Leave-one-out analysis to investigate whether the causal association between ANGPTL3 and IBD and CD was driven by a single SNP.** (a) Leave-one-out plot to assess if a single variant is driving the association between ANGPTL3 and IBD. (b) Leave-one-out plot to assess if a single variant is driving the association between ANGPTL3 and CD.


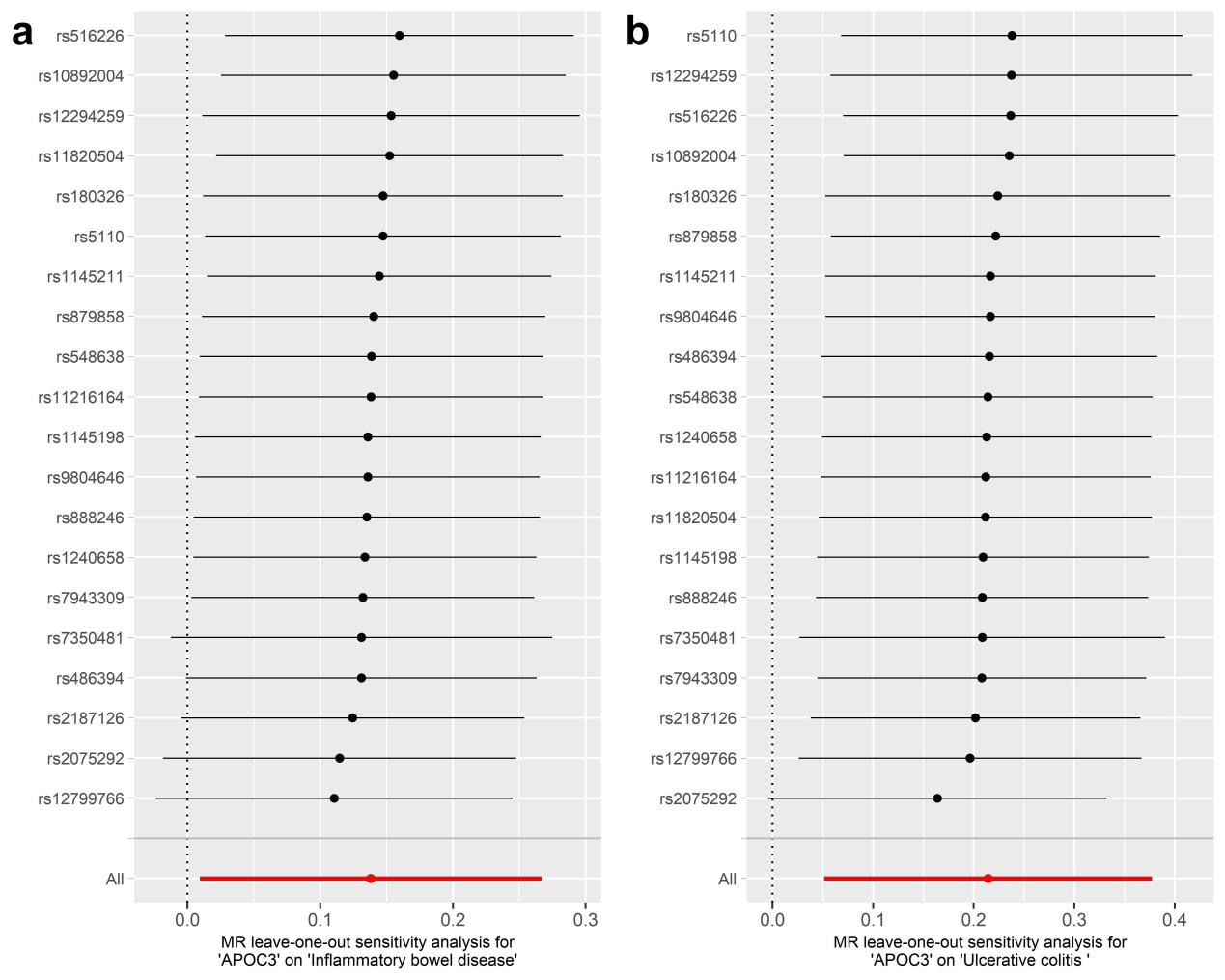


**Figure S3. Leave-one-out plot to analysis to investigate whether the causal association between APOC3 and IBD and UC was driven by a single SNP.** (a) Leave-one-out plot to assess if a single variant is driving the association between APOC3 and IBD. (b) Leave-one-out plot to assess if a single variant is driving the association between APOC3 and UC.


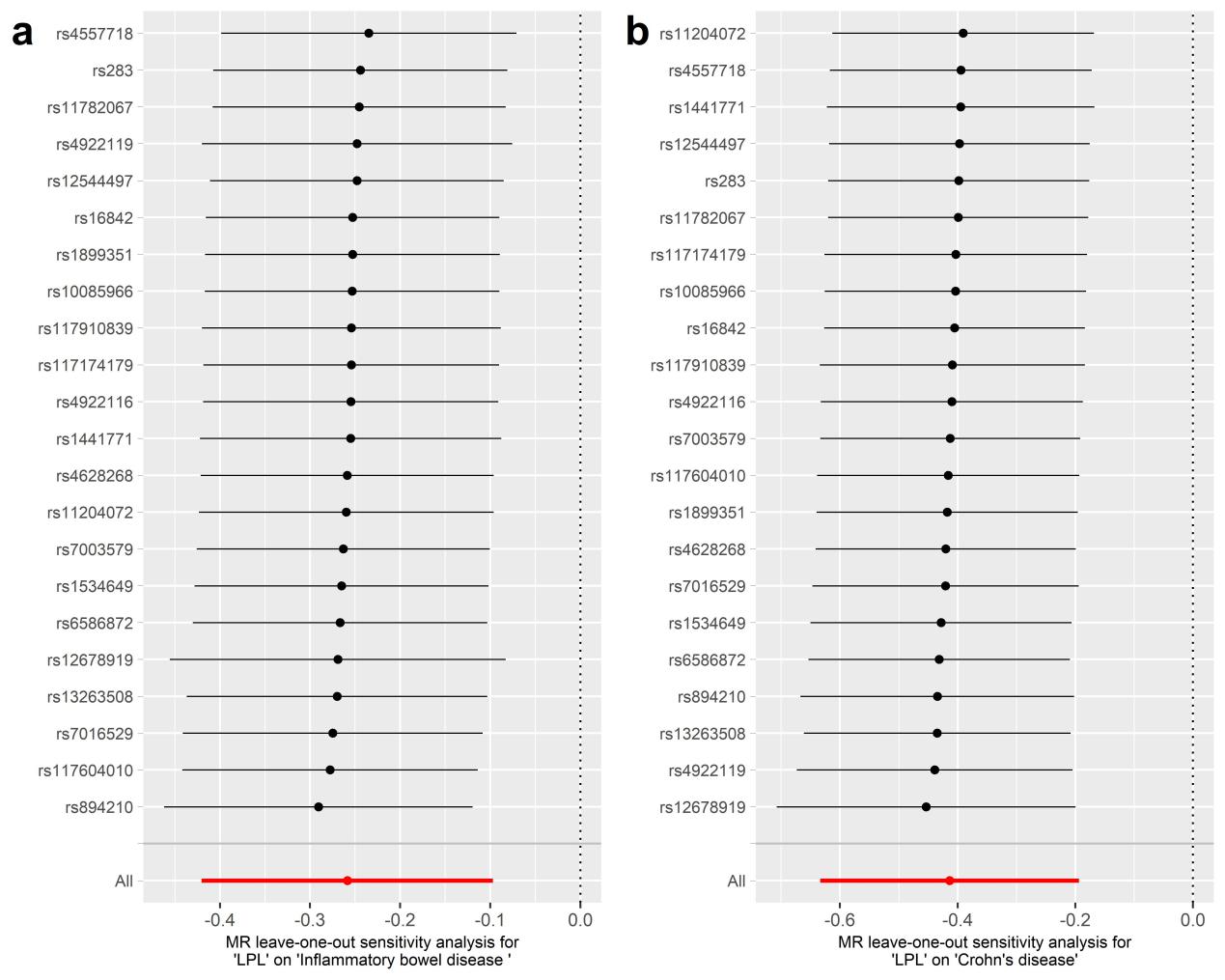


**Figure S4. Leave-one-out plot to analysis to investigate whether the causal association**

**between LPL and IBD and CD was driven by a single SNP.** (a) Leave-one-out plot to assess if a single variant is driving the association between LPL and IBD. (b) Leave-one-out plot to assess if a single variant is driving the association between LPL and CD.


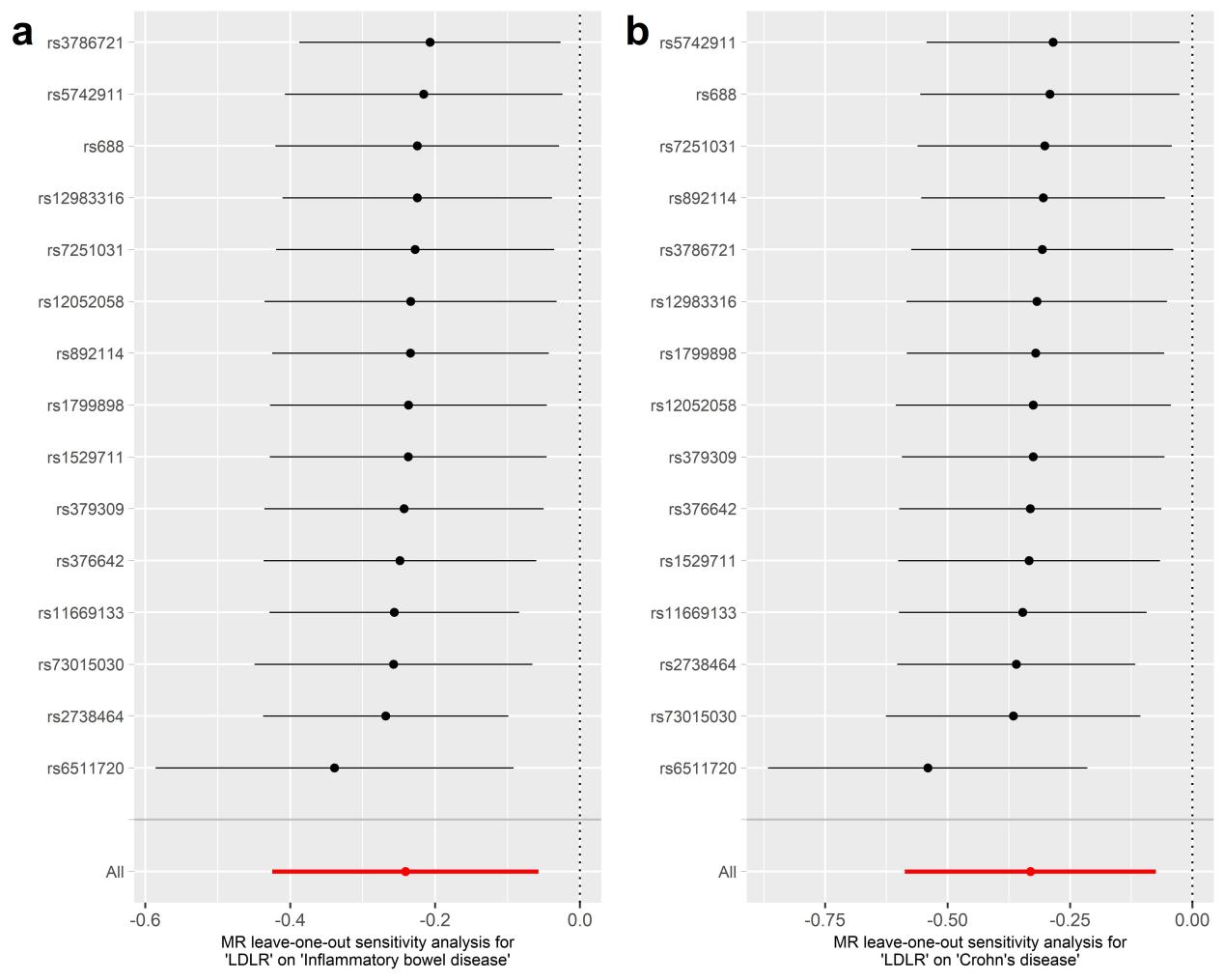


**Figure S5. Leave-one-out plot analysis to investigate whether the causal association between LDLR and IBD and CD was driven by a single SNP.** (a) Leave-one-out plot to assess if a single variant is driving the association between LDLR and IBD. (b) Leave-one-out plot to assess if a single variant is driving the association between LDLR and CD.


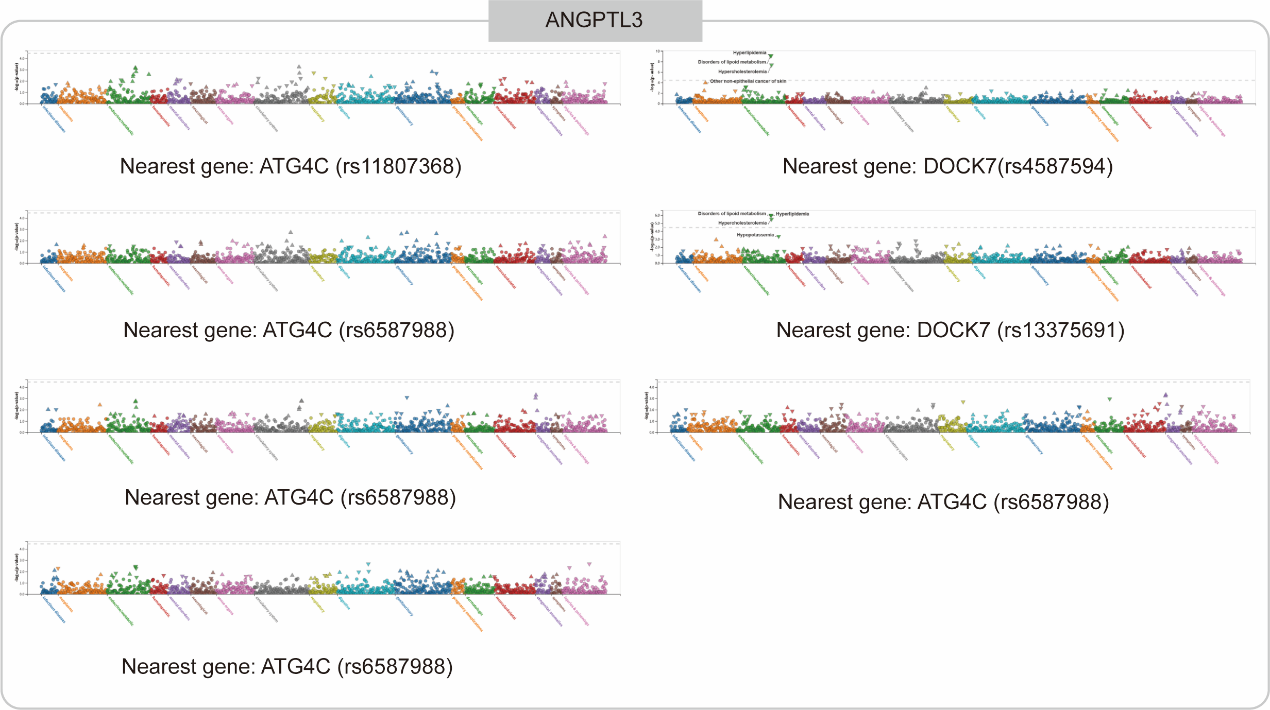


**Figure S6. Phenome-wide association analysis (PheWAS) results for ANGPTL3-related genetic variants.**


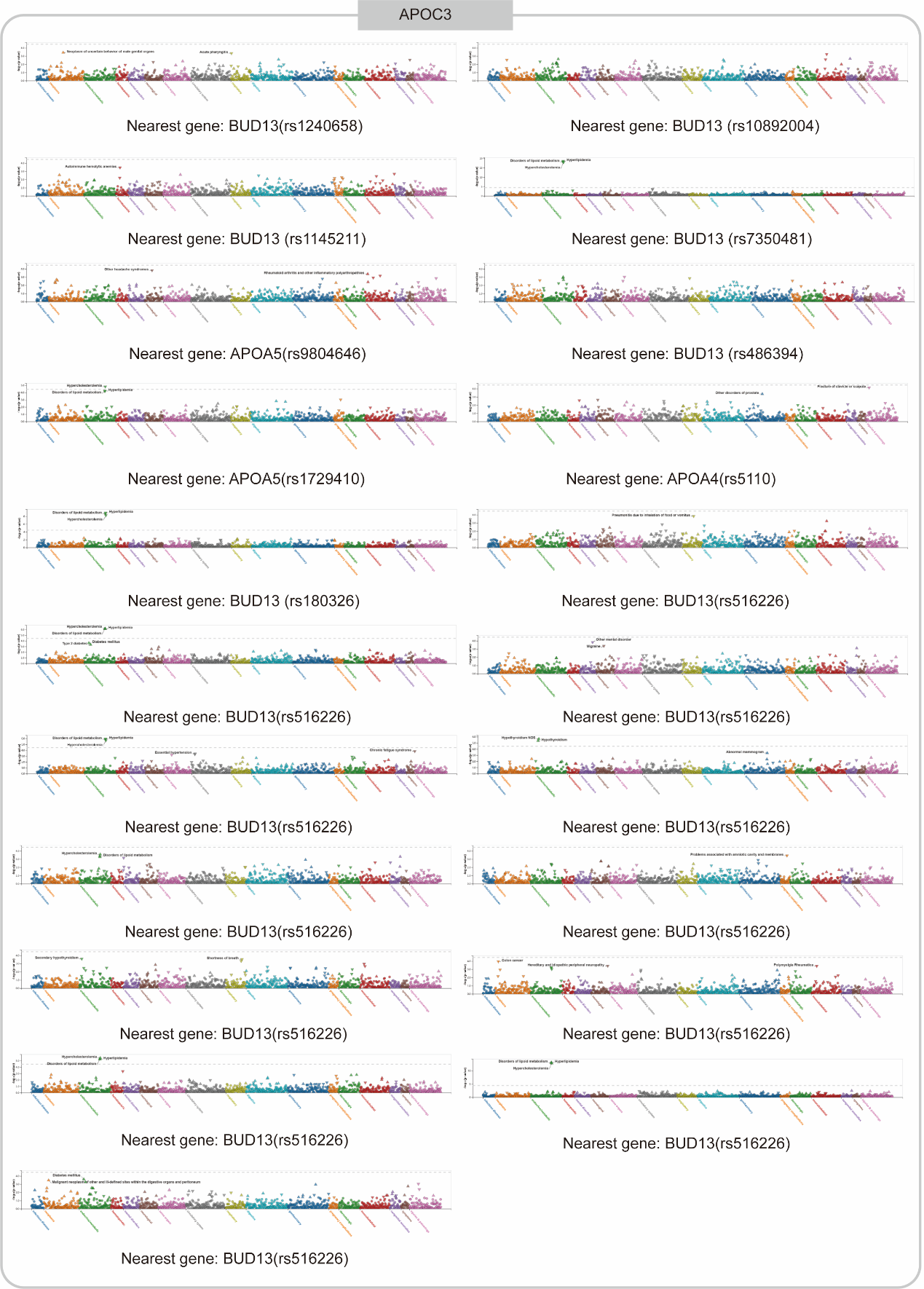


**Figure 7. Phenome-wide association analysis (PheWAS) results for LDLR-related genetic variants.**

**
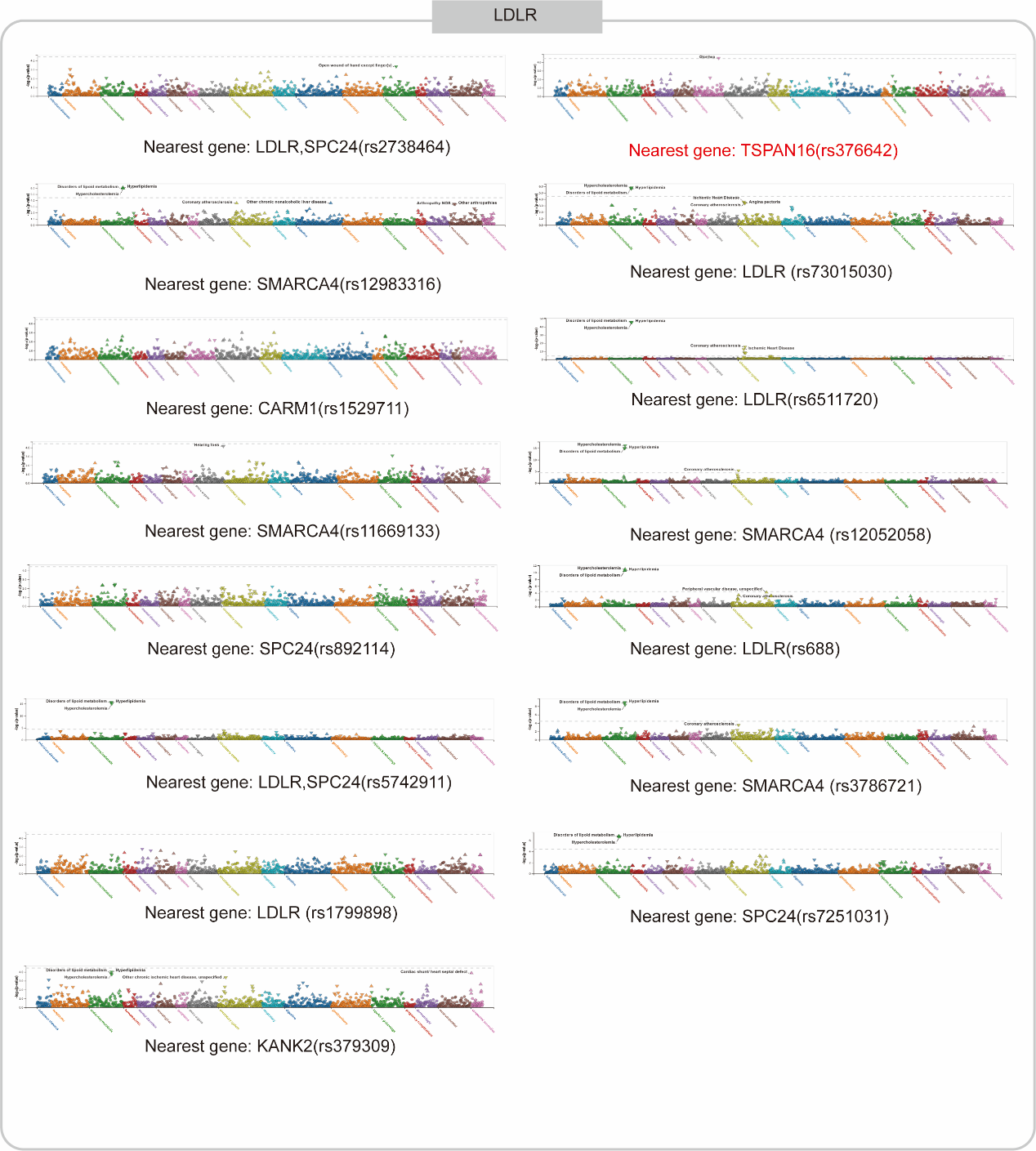
**

**Figure 8. Phenome-wide association analysis (PheWAS) results for LDLR-related genetic variants.**


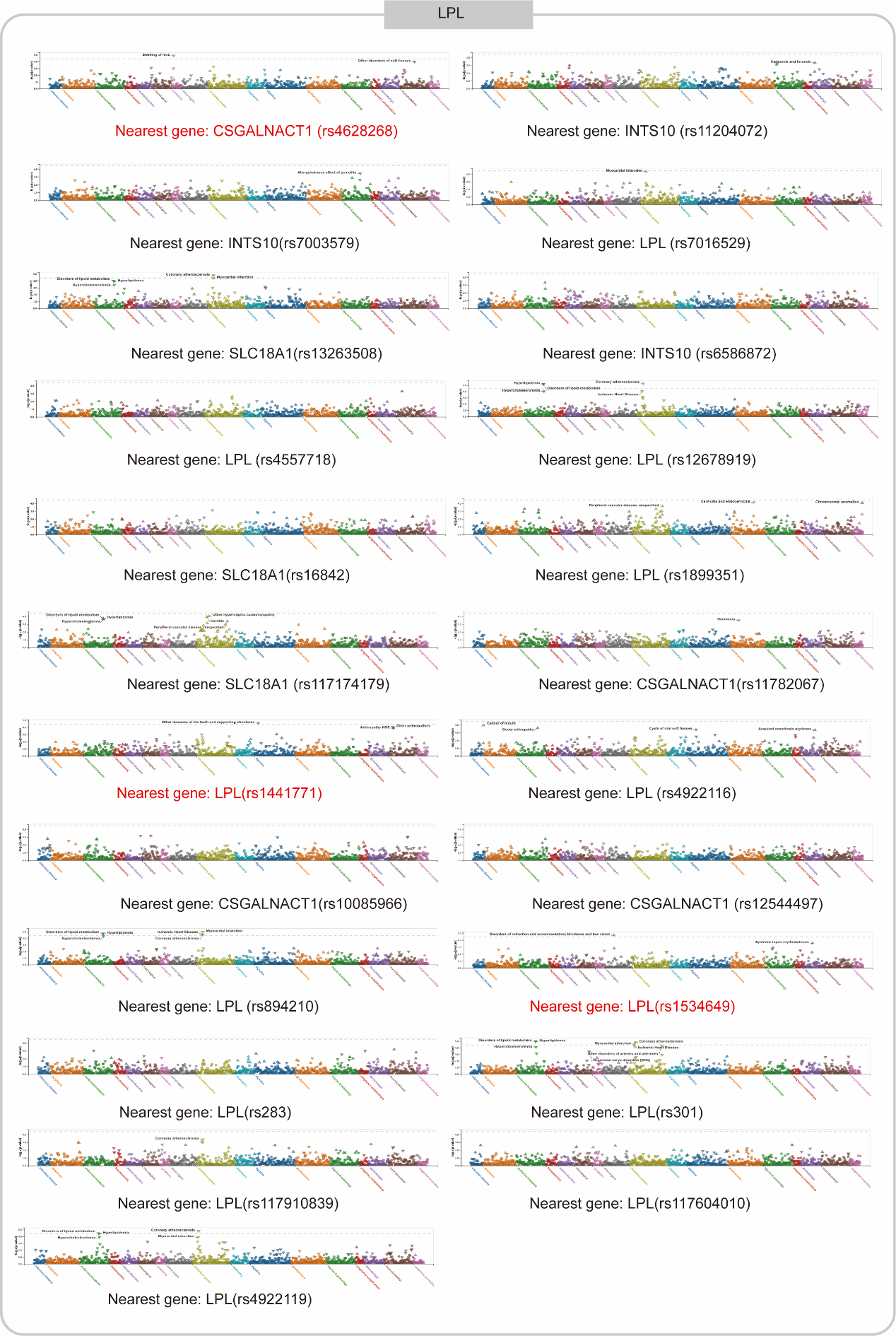


**Figure 9. Phenome-wide association analysis (PheWAS) results for LPL-related genetic variants.**


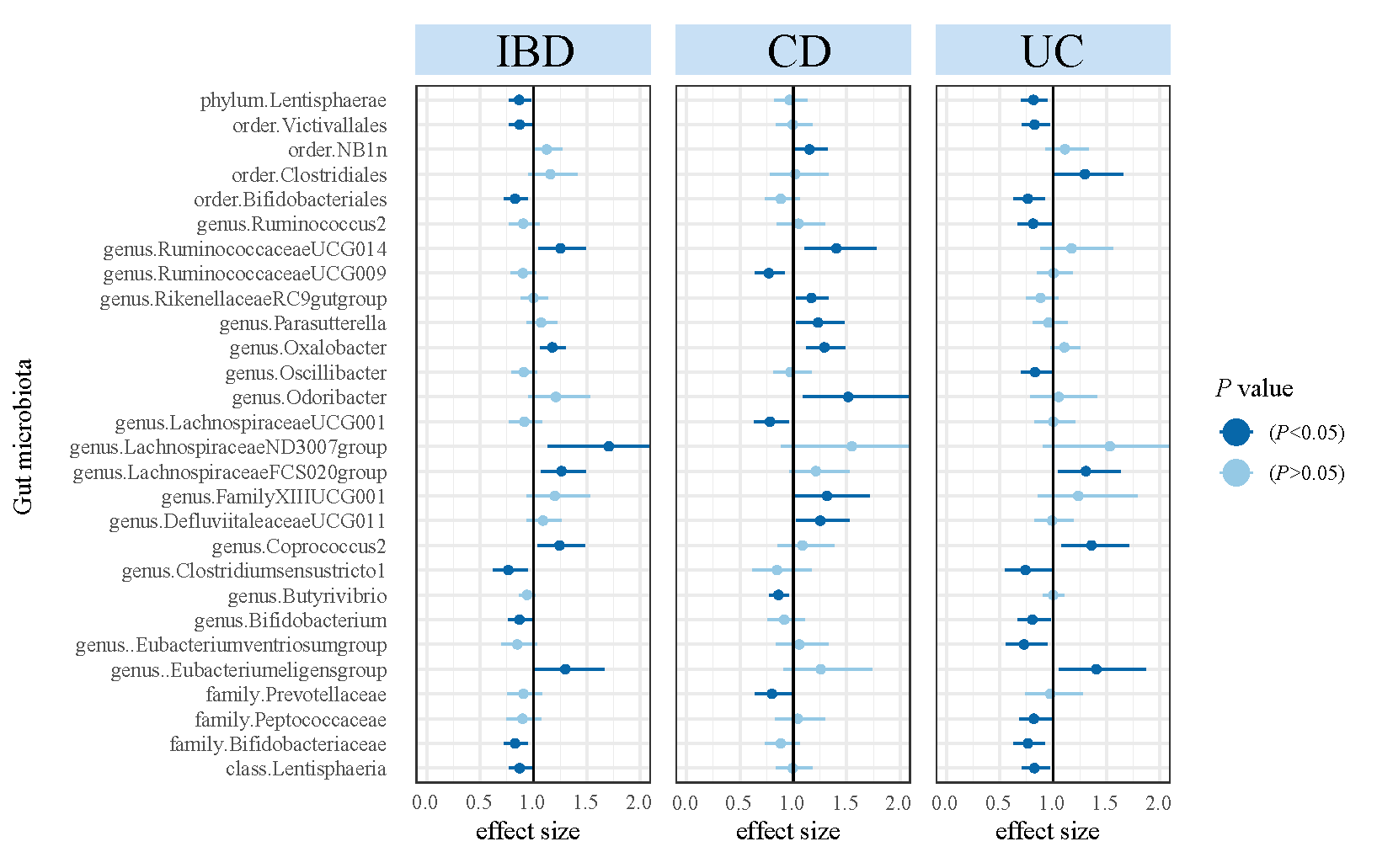


**Figure S10. Causal associations between gut microbiota on IBD, CD and UC.**

Abbreviations: IBD, inflammatory bowel disease; CD, Crohn’s disease; UC, ulcerative colitis.


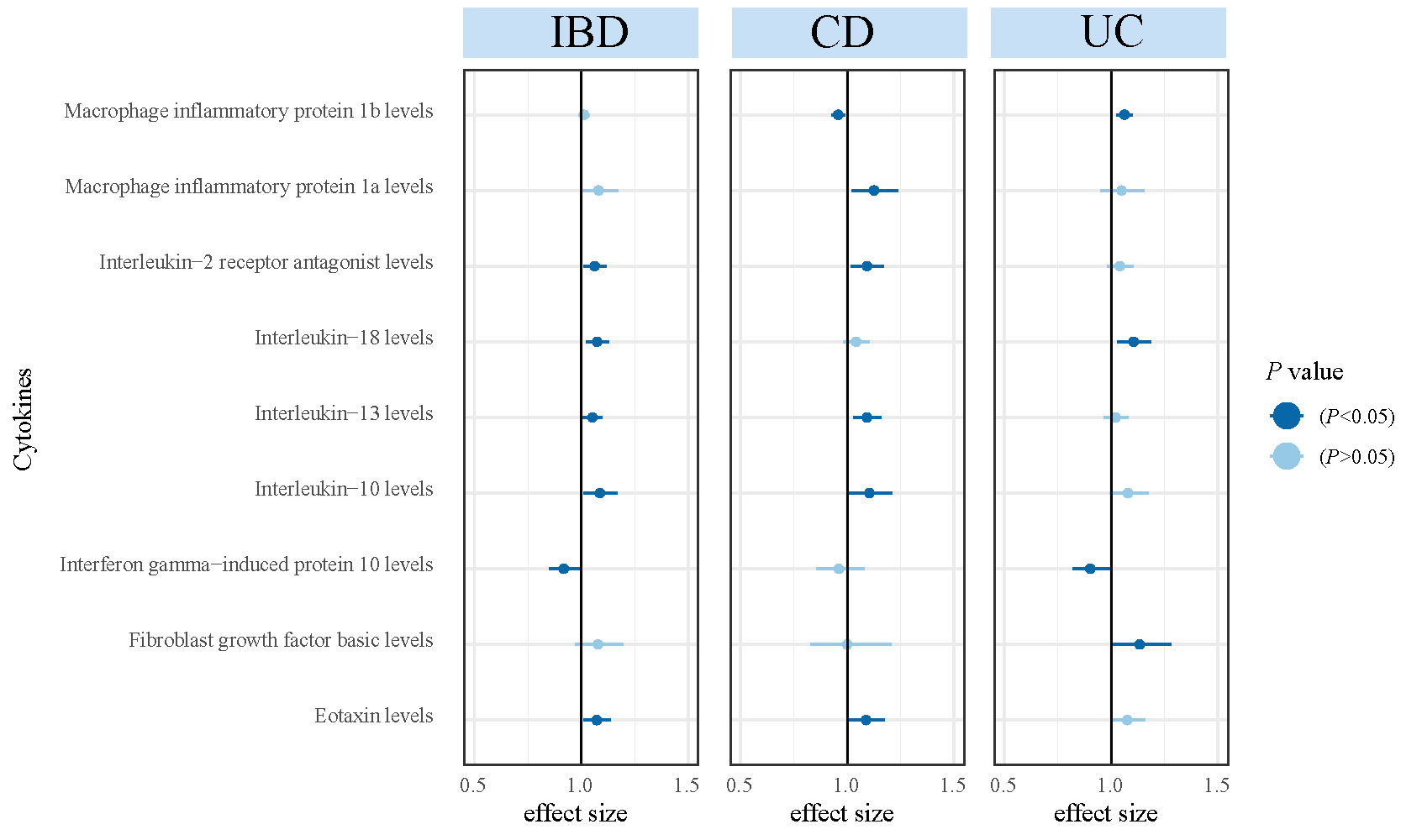


**Figure S11. Causal associations between inflammatory cytokines on IBD, CD and UC.**

Abbreviations: IBD, inflammatory bowel disease; CD, Crohn’s disease; UC, ulcerative colitis.

**
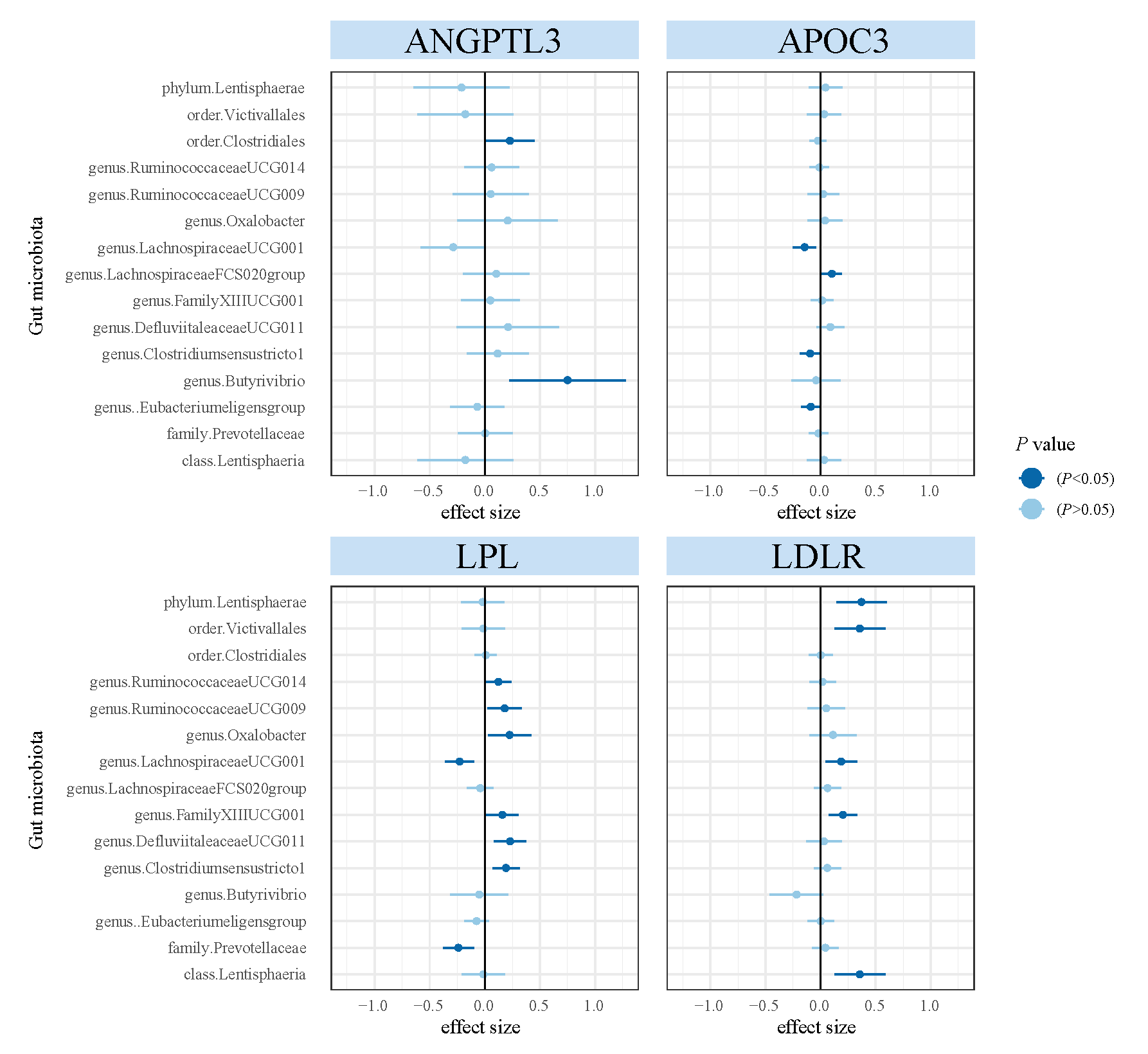
**

**Figure S12. Causal associations between lipid-lowering drug target and gut microbiota.**

Abbreviations: LPL, Lipoprotein Lipase; LDLR, LDL Receptor; ANGPTL3, Angiopoietin-like 3; APOC3, Apolipoprotein C-III.

**
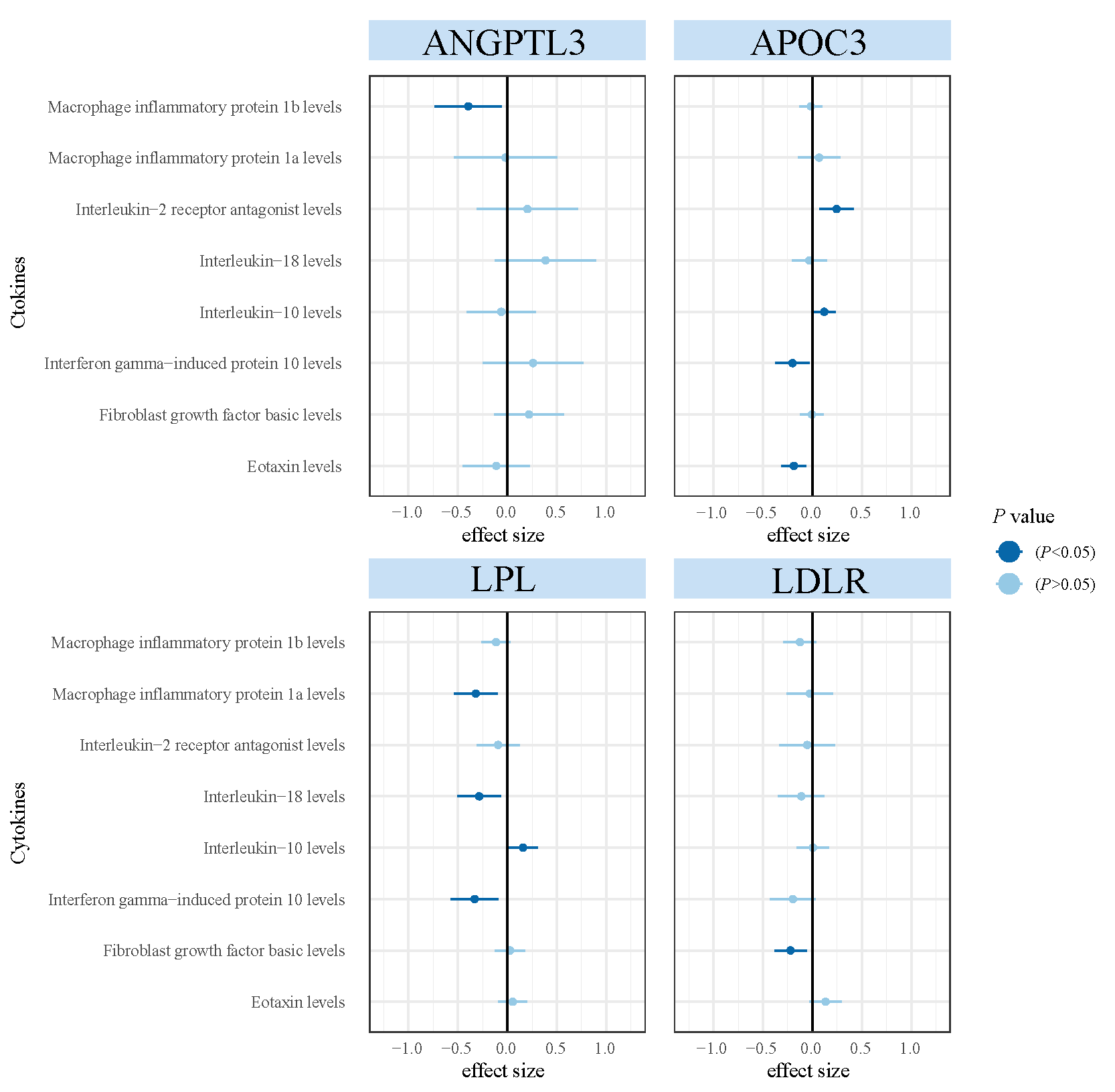
**

**Figure S13. Causal associations between lipid-lowering drug target and inflammatory cytokines.**

Abbreviations: LPL, Lipoprotein Lipase; LDLR, LDL Receptor; ANGPTL3, Angiopoietin-like 3; APOC3, Apolipoprotein C-III.
