# Supplementary material for: Lipid-lowering drug targets influence inflammatory bowel disease through gut microbiota and inflammatory cytokines
Source: J Lipid Res. 2025 Sep 1;66(9):100871. doi: 10.1016/j.jlr.2025.100871 (PMC12423403; doi:10.1016/j.jlr.2025.100871)
Supplement: Supporting Information Tables [file mmc2.docx]

**S1 Table. STROBE-MR checklist of recommended items to address in reports of Mendelian randomisation studies (1).**

| Item No. | Section | Checklist item | Section (paragraph number) |
| --- | --- | --- | --- |
| 1 | TITLE and ABSTRACT | Indicate Mendelian randomisation as the study’s design in the title and/or the abstract if that is a main purpose of the study | Title page & Abstract page |
|  | INTRODUCTION |  |  |
| 2 | Background | Explain the scientific background and rationale for the reported study. What is the exposure? Is a potential causal relationship between exposure and outcome plausible? Justify why MR is a helpful method to address the study question | Introduction (paragraphs 1-4) |
| 3 | Objectives | State specific objectives clearly, including pre-specified causal hypotheses (if any). State that MR is a method that, under specific assumptions, intends to estimate causal effects | Introduction (paragraph 5) |
|  | METHODS |  |  |
| 4 | Study design and data sources | Present key elements of the study design early in the article. Consider including a table listing sources of data for all phases of the study. For each data source contributing to the analysis, describe the following: | Methods (Genetic Variant selection & Mediators & Outcome & Statistical analysis) |
|  | a) | Setting: Describe the study design and the underlying population, if possible. Describe the setting, locations, and relevant dates, including periods of recruitment, exposure, follow-up, and data collection, when available. | Methods (Genetic Variant selection & Mediators & Outcome & Statistical analysis) |
|  | b) | Participants: Give the eligibility criteria, and the sources and methods of selection of participants. Report the sample size, and whether any power or sample size calculations were carried out prior to the main analysis | Methods (Genetic Variant selection & Mediators & Outcome & Statistical analysis & S2 Table & S3 Table) |
|  | c) | Describe measurement, quality control and selection of genetic variants | Methods (Genetic Variant selection & Mediators & Outcome) |
|  | d) | For each exposure, outcome, and other relevant variables, describe methods of assessment and diagnostic criteria for diseases | Methods (Genetic Variant selection & Mediators & Outcome & Statistical analysis) |
|  | e) | Provide details of ethics committee approval and participant informed consent, if relevant | Methods (Paragraph 1) |
| 5 | Assumptions | Explicitly state the three core IV assumptions for the main analysis (relevance, independence and exclusion restriction) as well assumptions for any additional or sensitivity analysis | Methods (Statistical analysis paragraph 2-6) |
| 6 | Statistical methods: main analysis | Describe statistical methods and statistics used |  |
|  | a) | Describe how quantitative variables were handled in the analyses (i.e., scale, units, model) | Methods (Statistical analysis paragraph 1 & S2 Table) |
|  | b) | Describe how genetic variants were handled in the analyses and, if applicable, how their weights were selected | Methods (Genetic Instrument Selection & Statistical analysis) |
|  | c) | Describe the MR estimator (e.g. two-stage least squares, Wald ratio) and related statistics. Detail the included covariates and, in case of two-sample MR, whether the same covariate set was used for adjustment in the two samples | Methods (Statistical analysis paragraph 1) |
|  | d) | Explain how missing data were addressed | N/A |
|  | e) | If applicable, indicate how multiple testing was addressed | Methods (Statistical analysis paragraph 7) |
| 7 | Assessment of assumptions | Describe any methods or prior knowledge used to assess the assumptions or justify their validity | Methods (Statistical analysis) |
| 8 | Sensitivity analyses and additional analyses | Describe any sensitivity analyses or additional analyses performed (e.g. comparison of effect estimates from different approaches, independent replication, bias analytic techniques, validation of instruments, simulations) | Methods (Statistical analysis &Sensitivity analyses) |
| 9 | Software and pre-registration |  |  |
|  | a) | Name statistical software and package(s), including version and settings used | Methods (Statistical analysis) |
|  | b) | State whether the study protocol and details were pre-registered (as well as when and where) | N/A |
|  | RESULTS |  |  |
| 10 | Descriptive data |  |  |
|  | a) | Report the numbers of individuals at each stage of included studies and reasons for exclusion. Consider use of a flow-diagram | Methods (Data source) & Fig 1 |
|  | b) | Report summary statistics for phenotypic exposure(s), outcome(s) and other relevant variables (e.g. means, SDs, proportions) | S2 Table |
|  | c) | If the data sources include meta-analyses of previous studies, provide the assessments of heterogeneity across these studies | N/A |
|  | d) | For two-sample Mendelian randomisation: | N/A |
|  |  | i. Provide justification of the similarity of the genetic variant-exposure associations between the exposure and outcome samples |  |
|  |  | ii. Provide information on the number of individuals who were in both samples for the exposure and for the outcome |  |
| 11 | Main results |  |  |
|  | a) | Report the associations between genetic variant and exposure, and between genetic variant and outcome, preferably on an interpretable scale | Results |
|  | b) | Report MR estimates of the relationship between exposure and outcome, and the measures of uncertainty from the MR analysis, on an interpretable scale, such as odds ratio or relative risk per SD difference | Results |
|  | c) | If relevant, consider translating estimates of relative risk into absolute risk for a meaningful time period | N/A |
|  | d) | Consider plots to visualize results (e.g. forest plot, scatterplot of associations between genetic variants and outcome versus between genetic variants and exposure) | S2-4 Fig |
| 12 | Assessment of assumptions |  |  |
|  | a) | Report the assessment of the validity of the assumptions | Results |
|  | b) | Report any additional statistics (e.g., assessments of heterogeneity across genetic variants, such as I2, Q statistic or E-value) | S5 Table |
| 13 | Sensitivity analyses and additional analyses |  |  |
|  | a) | Report any sensitivity analyses to assess the robustness of the main results to violations of the assumptions | Sensitivity analyses and Mediation analysis |
|  | b) | Report results from other sensitivity analyses or additional analyses |  |
|  | c) | Report any assessment of direction of causal relationship (e.g., bidirectional MR) | N/A |
|  | d) | When relevant, report and compare with estimates from non-MR analyses | N/A |
|  | e) | Consider additional plots to visualize results (e.g., leave-one-out analyses) | S2-5 Fig |
|  | DISCUSSION |  |  |
| 14 | Key results | Summarize key results with reference to study objectives | Discussion (paragraph 1) |
| 15 | Limitations | Discuss limitations of the study, taking into account the validity of the IV assumptions, other sources of potential bias, and imprecision. Discuss both direction and magnitude of any potential bias and any efforts to address them | Discussion (paragraph 5) |
| 16 | Interpretation |  |  |
|  | a) | Meaning: Give a cautious overall interpretation of results in the context of their limitations and in comparison with other studies | Discussion (paragraph 2-3) |
|  | b) | Mechanism: Discuss underlying biological mechanisms that could drive a potential causal relationship between the investigated exposure and the outcome, and whether the gene-environment equivalence assumption is reasonable. Use causal language carefully, clarifying that IV estimates may provide causal effects only under certain assumptions | Discussion (paragraph 2-3) |
|  | c) | Clinical relevance: Discuss whether the results have clinical or public policy relevance, and to what extent they inform effect sizes of possible interventions | Discussion (paragraph 2-3) |
| 17 | Generalizability | Discuss the generalizability of the study results (a) to other populations, (b) across other exposure periods/timings, and (c) across other levels of exposure | Discussion (paragraph 2-3) |
|  | OTHER INFORMATION |  |  |
| 18 | Funding | Describe sources of funding and the role of funders in the present study and, if applicable, sources of funding for the databases and original study or studies on which the present study is based | Funding section |
| 19 | Data and data sharing | Provide the data used to perform all analyses or report where and how the data can be accessed, and reference these sources in the article. Provide the statistical code needed to reproduce the results in the article,or report whether the code is publicly accessible and if so, where | Data share statement & S2 Table |
| 20 | Conflicts of Interest | All authors should declare all potential conflicts of interest | Competing interests section |

**S2 Table. Summary of GWAS datasets included in this study.**

| **Phenotype** | **Sample size (case/control)** | **Consortium or cohort study** | **Population** | **PubMed ID** |
| --- | --- | --- | --- | --- |
| LDL-C | 173,082 | GLGC | European | 24097068 |
| TG | 177,861 | GLGC | European | 24097068 |
| IBD | 34,652 | IIBDGC | European | 26192919 |
| CD | 20,883 | IIBDGC | European | 26192919 |
| UC | 27,432 | IIBDGC | European | 26192919 |
| Gut microbiota | 14,306 | MiBioGen | Europe, North America, and East Asia | 33462485 |
| Inflammatory Cytokine | 8986 | YFS and FINRISK surveys | European | 27989323 |

Abbreviations: LDL-C, low-density lipoprotein cholesterol; TG, triglyceride. IIBDGC, the International Inflammatory Bowel Disease Genetics Consortium; IBD, inflammatory bowel disease; CD, Crohn’s disease; UC, ulcerative colitis.

**S3 Table. Genetic variants that were used as instruments for lipid-lowering drugs in target genes.**

| **Gene** | **SNP** | **Chr** | **Pos** | **EAF** | **A1** | **Beta** | **SE** | **PVE** |
| --- | --- | --- | --- | --- | --- | --- | --- | --- |
| ANGPTL3 | rs11807368 | 1 | 63231401 | 0.4261 | T | 0.0312 | 0.0035 | 4.52E-04 |
| ANGPTL3 | rs4587594 | 1 | 63133930 | 0.31 | A | -0.0694 | 0.0035 | 2.21E-03 |
| ANGPTL3 | rs6587988 | 1 | 63252766 | 0.1372 | T | -0.0432 | 0.0045 | 5.18E-04 |
| ANGPTL3 | rs13375691 | 1 | 62996373 | 0.06728 | T | -0.0485 | 0.0058 | 3.93E-04 |
| ANGPTL3 | rs10493326 | 1 | 62953373 | 0.2467 | A | 0.0311 | 0.0039 | 3.58E-04 |
| ANGPTL3 | rs2095403 | 1 | 62860310 | 0.876 | A | -0.0294 | 0.005 | 1.94E-04 |
| ANGPTL3 | rs12123703 | 1 | 62861261 | 0.05673 | G | -0.0643 | 0.0095 | 5.28E-04 |
| APOC3 | rs1240658 | 11 | 116496360 | 0.1478 | C | 0.0346 | 0.0046 | 3.19E-04 |
| APOC3 | rs10892004 | 11 | 116514417 | 0.1359 | T | 0.0557 | 0.0082 | 5.07E-04 |
| APOC3 | rs1145211 | 11 | 116557216 | 0.6372 | C | 0.0358 | 0.0035 | 5.88E-04 |
| APOC3 | rs7350481 | 11 | 116586283 | 0.90237 | C | -0.2254 | 0.0066 | 6.52E-03 |
| APOC3 | rs9804646 | 11 | 116665079 | 0.08971 | T | -0.0524 | 0.0064 | 3.88E-04 |
| APOC3 | rs486394 | 11 | 116526322 | 0.2942 | C | 0.0665 | 0.0038 | 1.72E-03 |
| APOC3 | rs1729410 | 11 | 116665661 | 0.4301 | C | -0.0362 | 0.0035 | 6.02E-04 |
| APOC3 | rs5110 | 11 | 116691634 | 0.06464 | A | 0.156 | 0.0124 | 1.93E-03 |
| APOC3 | rs180326 | 11 | 116624703 | 0.6372 | T | -0.0839 | 0.0036 | 3.35E-03 |
| APOC3 | rs516226 | 11 | 116519655 | 0.2243 | T | 0.067 | 0.0065 | 1.17E-03 |
| APOC3 | rs879858 | 11 | 116573120 | 0.02375 | A | 0.1008 | 0.015 | 5.03E-04 |
| APOC3 | rs888246 | 11 | 116724232 | 0.08179 | T | 0.0707 | 0.0058 | 9.35E-04 |
| APOC3 | rs2075292 | 11 | 116732512 | 0.8509 | T | -0.1026 | 0.005 | 2.36E-03 |
| APOC3 | rs11216164 | 11 | 116734545 | 0.3602 | A | -0.0335 | 0.0036 | 4.87E-04 |
| APOC3 | rs12799766 | 11 | 116558427 | 0.219 | A | 0.0884 | 0.0039 | 2.88E-03 |
| APOC3 | rs1145198 | 11 | 116574336 | 0.2269 | A | -0.046 | 0.0039 | 7.82E-04 |
| APOC3 | rs2187126 | 11 | 116635784 | 0.05409 | G | -0.0543 | 0.0069 | 3.48E-04 |
| APOC3 | rs548638 | 11 | 116737093 | 0.2084 | G | -0.0332 | 0.0042 | 3.67E-04 |
| APOC3 | rs11820504 | 11 | 116529442 | 0.2032 | C | 0.0604 | 0.0044 | 1.09E-03 |
| APOC3 | rs12294259 | 11 | 116637146 | 0.05937 | T | 0.219 | 0.0069 | 5.81E-03 |
| APOC3 | rs7943309 | 11 | 116773653 | 0.03958 | A | -0.0605 | 0.0087 | 2.80E-04 |
| APOB | rs17398765 | 2 | 21270751 | 0.0686 | G | 0.0916 | 0.0076 | 8.63E-04 |
| APOB | rs6756743 | 2 | 21301892 | 0.04222 | T | 0.0553 | 0.0092 | 2.20E-04 |
| APOB | rs111826230 | 2 | 21378453 | 0.06332 | G | 0.0717 | 0.0099 | 6.32E-04 |
| APOB | rs11685356 | 2 | 21197159 | 0.2296 | T | 0.0517 | 0.0042 | 8.76E-04 |
| APOB | rs12720842 | 2 | 21257927 | 0.02111 | C | 0.0993 | 0.0116 | 4.54E-04 |
| APOB | rs492399 | 2 | 21015763 | 0.96438 | A | -0.0629 | 0.0102 | 2.20E-04 |
| APOB | rs11679386 | 2 | 20985414 | 0.128 | C | 0.0452 | 0.0061 | 3.48E-04 |
| APOB | rs533617 | 2 | 21233972 | 0.04881 | C | -0.1405 | 0.0098 | 1.23E-03 |
| APOB | rs3791981 | 2 | 21245367 | 0.1201 | G | -0.0939 | 0.0067 | 1.21E-03 |
| APOB | rs6729410 | 2 | 21099871 | 0.3549 | G | -0.0389 | 0.004 | 5.48E-04 |
| APOB | rs4665788 | 2 | 21188488 | 0.7744 | C | -0.0667 | 0.0042 | 1.52E-03 |
| APOB | rs6754295 | 2 | 21206183 | 0.2586 | G | -0.0628 | 0.0042 | 1.29E-03 |
| APOB | rs312028 | 2 | 21460827 | 0.7784 | C | -0.039 | 0.0061 | 4.55E-04 |
| APOB | rs1367117 | 2 | 21263900 | 0.2876 | A | 0.1186 | 0.004 | 5.06E-03 |
| APOB | rs6725189 | 2 | 21219001 | 0.2348 | T | -0.0604 | 0.0045 | 1.04E-03 |
| APOB | rs12720796 | 2 | 21261998 | 0.02243 | C | 0.0909 | 0.0141 | 2.70E-04 |
| APOB | rs7567653 | 2 | 21276962 | 0.03694 | A | -0.1145 | 0.0112 | 6.48E-04 |
| APOB | rs12710745 | 2 | 21112689 | 0.3958 | G | -0.0429 | 0.0038 | 7.36E-04 |
| APOB | rs4635554 | 2 | 21389659 | 0.3061 | G | 0.0783 | 0.0041 | 2.32E-03 |
| APOB | rs515135 | 2 | 21286057 | 0.7823 | C | 0.1394 | 0.0048 | 4.85E-03 |
| ABCG5/ABCG8 | rs1025447 | 2 | 44022970 | 0.1583 | C | 0.0418 | 0.0048 | 4.40E-04 |
| ABCG5/ABCG8 | rs10208987 | 2 | 44043135 | 0.08971 | G | -0.0486 | 0.0068 | 2.95E-04 |
| ABCG5/ABCG8 | rs17424122 | 2 | 44075217 | 0.06596 | A | 0.0645 | 0.0091 | 3.30E-04 |
| ABCG5/ABCG8 | rs6544713 | 2 | 44073881 | 0.7058 | C | -0.0806 | 0.0041 | 2.23E-03 |
| ABCG5/ABCG8 | rs4148177 | 2 | 44057102 | 0.4327 | A | -0.0365 | 0.0051 | 6.16E-04 |
| ABCG5/ABCG8 | rs4148218 | 2 | 44099582 | 0.1913 | A | -0.0441 | 0.0047 | 5.09E-04 |
| ABCG5/ABCG8 | rs4953023 | 2 | 44074000 | 0.08443 | A | -0.1313 | 0.0074 | 1.85E-03 |
| HMGCR | rs3857388 | 5 | 74620377 | 0.128 | C | 0.0421 | 0.0059 | 2.94E-04 |
| HMGCR | rs10515198 | 5 | 74641560 | 0.1029 | A | 0.0599 | 0.0061 | 5.57E-04 |
| HMGCR | rs12916 | 5 | 74656539 | 0.4314 | C | 0.0733 | 0.0038 | 2.21E-03 |
| LDLR | rs2738464 | 19 | 11242307 | 0.8747 | C | 0.0422 | 0.0061 | 2.88E-04 |
| LDLR | rs376642 | 19 | 11392431 | 0.285 | G | -0.0233 | 0.004 | 1.98E-04 |
| LDLR | rs12983316 | 19 | 11114352 | 0.1689 | A | 0.0514 | 0.0052 | 5.93E-04 |
| LDLR | rs73015030 | 19 | 11207516 | 0.02507 | T | -0.1517 | 0.0148 | 1.26E-03 |
| LDLR | rs1529711 | 19 | 11023434 | 0.1609 | T | 0.0323 | 0.0051 | 2.34E-04 |
| LDLR | rs6511720 | 19 | 11202306 | 0.09763 | G | -0.2209 | 0.0061 | 7.63E-03 |
| LDLR | rs11669133 | 19 | 11092139 | 0.04222 | T | 0.0501 | 0.0098 | 1.56E-04 |
| LDLR | rs12052058 | 19 | 11159525 | 0.248 | G | -0.075 | 0.0043 | 1.79E-03 |
| LDLR | rs892114 | 19 | 11266584 | 0.7731 | C | -0.0353 | 0.0047 | 3.61E-04 |
| LDLR | rs688 | 19 | 11227602 | 0.4472 | T | 0.054 | 0.0037 | 1.28E-03 |
| LDLR | rs5742911 | 19 | 11243445 | 0.2678 | G | -0.0606 | 0.0057 | 1.45E-03 |
| LDLR | rs3786721 | 19 | 11146499 | 0.5383 | T | -0.0468 | 0.0038 | 9.26E-04 |
| LDLR | rs1799898 | 19 | 11227554 | 0.1504 | G | -0.0333 | 0.0054 | 2.29E-04 |
| LDLR | rs7251031 | 19 | 11266693 | 0.2889 | A | 0.0456 | 0.0046 | 7.14E-04 |
| LDLR | rs379309 | 19 | 11284302 | 0.4974 | A | -0.0313 | 0.0039 | 3.86E-04 |
| LPL | rs4628268 | 8 | 19514934 | 0.4776 | C | -0.0215 | 0.0033 | 2.39E-04 |
| LPL | rs11204072 | 8 | 19650333 | 0.153 | C | -0.0379 | 0.0044 | 4.22E-04 |
| LPL | rs7003579 | 8 | 19728586 | 0.785 | C | -0.0255 | 0.004 | 2.29E-04 |
| LPL | rs7016529 | 8 | 19806631 | 0.01319 | C | 0.1911 | 0.014 | 1.21E-03 |
| LPL | rs13263508 | 8 | 19942181 | 0.5699 | T | -0.0517 | 0.0034 | 1.30E-03 |
| LPL | rs6586872 | 8 | 19715236 | 0.7441 | G | 0.0339 | 0.0038 | 4.48E-04 |
| LPL | rs4557718 | 8 | 19890654 | 0.1266 | C | 0.0578 | 0.0059 | 5.62E-04 |
| LPL | rs12678919 | 8 | 19844222 | 0.1214 | G | -0.1702 | 0.0056 | 5.17E-03 |
| LPL | rs16842 | 8 | 19968929 | 0.2533 | C | 0.0274 | 0.0037 | 3.17E-04 |
| LPL | rs1899351 | 8 | 19863301 | 0.01319 | A | 0.1236 | 0.0156 | 3.96E-04 |
| LPL | rs117174179 | 8 | 19941078 | 0.02507 | T | 0.1181 | 0.0185 | 4.91E-04 |
| LPL | rs11782067 | 8 | 19623129 | 0.6517 | A | -0.0232 | 0.0036 | 2.36E-04 |
| LPL | rs1441771 | 8 | 19757300 | 0.1636 | T | -0.0669 | 0.0043 | 1.36E-03 |
| LPL | rs4922116 | 8 | 19832778 | 0.1478 | A | -0.0435 | 0.0044 | 5.50E-04 |
| LPL | rs10085966 | 8 | 19576448 | 0.4591 | T | -0.03 | 0.0037 | 4.59E-04 |
| LPL | rs12544497 | 8 | 19605966 | 0.1517 | G | -0.0339 | 0.0044 | 3.34E-04 |
| LPL | rs894210 | 8 | 19865843 | 0.5646 | A | -0.0671 | 0.0033 | 2.34E-03 |
| LPL | rs1534649 | 8 | 19799641 | 0.4485 | T | -0.028 | 0.0035 | 3.65E-04 |
| LPL | rs283 | 8 | 19815098 | 0.2348 | T | 0.037 | 0.0044 | 4.16E-04 |
| LPL | rs301 | 8 | 19816934 | 0.4274 | C | -0.1089 | 0.0039 | 4.37E-03 |
| LPL | rs117910839 | 8 | 19822741 | 0.03694 | A | -0.1365 | 0.0141 | 1.08E-03 |
| LPL | rs117604010 | 8 | 19848396 | 0.01979 | A | -0.1371 | 0.017 | 8.00E-04 |
| LPL | rs4922119 | 8 | 19874153 | 0.4499 |  | -0.0708 | 0.0034 | 2.51E-03 |
| NPC1L1 | rs2073547 | 7 | 44582331 | 0.1939 | G | 0.0485 | 0.0049 | 5.76E-04 |
| NPC1L1 | rs217386 | 7 | 44600695 | 0.4077 | A | -0.0363 | 0.0038 | 5.27E-04 |
| PCSK9 | rs6662286 | 1 | 55730327 | 0.90633 | C | 0.0989 | 0.0073 | 1.08E-03 |
| PCSK9 | rs2647281 | 1 | 55724704 | 0.05541 | G | 0.0589 | 0.0095 | 2.42E-04 |
| PCSK9 | rs2479409 | 1 | 55504650 | 0.6675 | A | -0.0642 | 0.0041 | 1.42E-03 |
| PCSK9 | rs11591147 | 1 | 55505647 | 0.01715 | T | -0.497 | 0.018 | 9.75E-03 |
| PCSK9 | rs11583974 | 1 | 55551718 | 0.03034 | A | 0.0646 | 0.0117 | 3.05E-04 |
| PCSK9 | rs2479394 | 1 | 55486064 | 0.715 | A | -0.0386 | 0.0041 | 5.12E-04 |
| PCSK9 | rs11206510 | 1 | 55496039 | 0.1544 | C | -0.0831 | 0.005 | 1.60E-03 |
| PCSK9 | rs572512 | 1 | 55517344 | 0.3456 | T | 0.0478 | 0.0047 | 6.87E-04 |
| PCSK9 | rs4927207 | 1 | 55713628 | 0.1715 | A | -0.0692 | 0.0049 | 1.15E-03 |
| PCSK9 | rs11206514 | 1 | 55516004 | 0.6108 | A | 0.0507 | 0.0041 | 8.83E-04 |
| PCSK9 | rs1874776 | 1 | 55743519 | 0.7823 | C | 0.044 | 0.0044 | 5.78E-04 |
| PCSK9 | rs585131 | 1 | 55524116 | 0.8153 | T | 0.0637 | 0.005 | 9.67E-04 |
| PCSK9 | rs1475701 | 1 | 55638546 | 0.03562 | C | 0.0904 | 0.0092 | 5.60E-04 |

Abbreviations: SNP, single nucleotide polymorphism; CHR, chromosome; A1, effect allele; EAF, effect allele frequency; Beta, beta-coefficient (in standard deviation units); Se, standard error; F, F statistic; ABCG5, ATP Binding Cassette Subfamily G Member 5; LPL, Lipoprotein Lipase; LDLR, LDL Receptor; ANGPTL3, Angiopoietin-like 3; APOC3, Apolipoprotein C-III; HMGCR, HMG-CoA Reductase; NPC1L1, Niemann-Pick C1-like Protein 1; PCSK9, Proprotein Convertase Subtilisin/Kexin Type 9.

**S4 Table.** IBD-associated risk alleles and mapped genes from the GWAS Catalog.

| **Risk Allele** | **Mapped Genes** | **Trait Name** | **Accession Id** | **Pubmed Id** |
| --- | --- | --- | --- | --- |
| rs6596 | SNX20 | IBD | GCST003958 | 28008999 |
| rs13333062 | LINC02128 | IBD | GCST003958 | 28008999 |
| rs1049526 | BRD2 | IBD | GCST003958 | 28008999 |
| rs501916 | SEMA6D | IBD | GCST003958 | 28008999 |
| rs2395128 | DUSP29 | IBD | GCST003958 | 28008999 |
| rs11209026 | IL23R | IBD | GCST003958 | 28008999 |
| rs3024505 | IL10,Y_RNA | IBD | GCST003958 | 28008999 |
| rs4903214 | VSX2 | IBD | GCST003958 | 28008999 |
| rs6596 | SNX20 | IBD | GCST003958 | 28008999 |
| rs13333062 | LINC02128 | IBD | GCST003958 | 28008999 |
| rs1049526 | BRD2 | IBD | GCST003958 | 28008999 |
| rs501916 | SEMA6D | IBD | GCST003958 | 28008999 |
| rs6596 | SNX20 | IBD | GCST003958 | 28008999 |
| rs13333062 | LINC02128 | IBD | GCST003958 | 28008999 |
| rs1049526 | BRD2 | IBD | GCST003958 | 28008999 |
| rs2395128 | DUSP29 | IBD | GCST003958 | 28008999 |
| rs2187668 | HLAQA1 | IBD | GCST003958 | 28008999 |
| rs11209026 | IL23R | IBD | GCST003958 | 28008999 |
| rs59043219 | IRF6 | IBD | GCST004131 | 28067908 |
| rs6740847 | LINC01934,ITGA4 | IBD | GCST004131 | 28067908 |
| rs76527535 | BOKS1 | IBD | GCST004131 | 28067908 |
| rs2593855 | FOXP1 | IBD | GCST004131 | 28067908 |
| rs503734 | IMPG2 | IBD | GCST004131 | 28067908 |
| rs11734570 | LINC02278,KLF3S1 | IBD | GCST004131 | 28067908 |
| rs67289879 | CCND3 | IBD | GCST004131 | 28067908 |
| rs11768365 | GRID2IP | IBD | GCST004131 | 28067908 |
| rs149169037 | ITGB8,EEF1A1P27 | IBD | GCST004131 | 28067908 |
| rs243505 | CUL1 | IBD | GCST004131 | 28067908 |
| rs111456533 | EEF1AKMT2 | IBD | GCST004131 | 28067908 |
| rs11548656 | PLCG2 | IBD | GCST004131 | 28067908 |
| rs4256018 | FERMT1 | IBD | GCST004131 | 28067908 |
| rs4845604 | RORC | IBD | GCST004131 | 28067908 |
| rs11581607 | IL23R | IBD | GCST004131 | 28067908 |
| rs11742570 | RNU150P,TTC33 | IBD | GCST004131 | 28067908 |
| rs2066844 | NOD2 | IBD | GCST004131 | 28067908 |
| rs56167332 | IL12B,LINC01845 | IBD | GCST004131 | 28067908 |
| rs10761659 | ALDH7A1P4,LINC02929 | IBD | GCST004131 | 28067908 |
| rs10781499 | CARD9 | IBD | GCST004131 | 28067908 |
| rs4409764 | LINC01475 | IBD | GCST004131 | 28067908 |
| rs3197999 | MST1 | IBD | GCST004131 | 28067908 |
| rs11236797 | LINC02757,EMSY | IBD | GCST004131 | 28067908 |
| rs6927022 | HLAQA1 | IBD | GCST004131 | 28067908 |
| rs3024505 | IL10,Y_RNA | IBD | GCST004131 | 28067908 |
| rs2836878 | LINC02940,RPL23AP12 | IBD | GCST004131 | 28067908 |
| rs2823286 | LINC02920,CYCSP42 | IBD | GCST004131 | 28067908 |
| rs75900472 | HNRNPA1P41,JAK2 | IBD | GCST004131 | 28067908 |
| rs7608910 | PUS10 | IBD | GCST004131 | 28067908 |
| rs6752107 | ATG16L1 | IBD | GCST004131 | 28067908 |
| rs2188962 | CARINH | IBD | GCST004131 | 28067908 |
| rs12946510 | IKZF3,GRB7 | IBD | GCST004131 | 28067908 |
| rs6062496 | TNFRSF6B,RTEL1NFRSF6B | IBD | GCST004131 | 28067908 |
| rs2413583 | PDGFB,RPL3 | IBD | GCST004131 | 28067908 |
| rs7282490 | GATD3 | IBD | GCST004131 | 28067908 |
| rs11614178 | IFNGS1 | IBD | GCST004131 | 28067908 |
| rs7554511 | INAVA | IBD | GCST004131 | 28067908 |
| rs17293632 | SMAD3 | IBD | GCST004131 | 28067908 |
| rs6426833 | RNF186S1,OTUD3 | IBD | GCST004131 | 28067908 |
| rs6017342 | LINC01620 | IBD | GCST004131 | 28067908 |
| rs12942547 | STAT3 | IBD | GCST004131 | 28067908 |
| rs2024092 | SBNO2 | IBD | GCST004131 | 28067908 |
| rs80262450 | PTPN2 | IBD | GCST004131 | 28067908 |
| rs4380874 | PIGCP2,DLD | IBD | GCST004131 | 28067908 |
| rs148319899 | LRRK2 | IBD | GCST004131 | 28067908 |
| rs11741861 | ZNF300,IRGM | IBD | GCST004131 | 28067908 |
| rs2266959 | UBE2L3 | IBD | GCST004131 | 28067908 |
| rs12568930 | ZBTB40,PPIAP34 | IBD | GCST004131 | 28067908 |
| rs4676408 | GPR35,AQP12B | IBD | GCST004131 | 28067908 |
| rs1819333 | RNASET2,MIR3939 | IBD | GCST004131 | 28067908 |
| rs56116661 | LPP | IBD | GCST004131 | 28067908 |
| rs1847472 | BACH2 | IBD | GCST004131 | 28067908 |
| rs653178 | ATXN2 | IBD | GCST004131 | 28067908 |
| rs11195128 | DUSP5T | IBD | GCST004131 | 28067908 |
| rs259964 | ZNF831 | IBD | GCST004131 | 28067908 |
| rs4976646 | RGS14 | IBD | GCST004131 | 28067908 |
| rs17656349 | CAMK2A | IBD | GCST004131 | 28067908 |
| rs10495903 | THADA | IBD | GCST004131 | 28067908 |
| rs2284553 | IFNGR2 | IBD | GCST004131 | 28067908 |
| rs7517810 | AIMP1P2,SLC25A38P1 | IBD | GCST004131 | 28067908 |
| rs9297145 | KPNA7,SMURF1 | IBD | GCST004131 | 28067908 |
| rs6920220 | LINC03004 | IBD | GCST004131 | 28067908 |
| rs4656958 | ITLN2,ITLN1 | IBD | GCST004131 | 28067908 |
| rs11221332 | ETS1 | IBD | GCST004131 | 28067908 |
| rs3764147 | LACC1 | IBD | GCST004131 | 28067908 |
| rs5763767 | HORMAD2 | IBD | GCST004131 | 28067908 |
| rs7240004 | CTIF,SMAD7 | IBD | GCST004131 | 28067908 |
| rs6586030 | TSPAN14 | IBD | GCST004131 | 28067908 |
| rs3180018 | SCAMP3 | IBD | GCST004131 | 28067908 |
| rs2816958 | NR5A2 | IBD | GCST004131 | 28067908 |
| rs1260326 | GCKR | IBD | GCST004131 | 28067908 |
| rs1479918 | ADAD1,IL2 | IBD | GCST004131 | 28067908 |
| rs798502 | GNA12,AMZ1 | IBD | GCST004131 | 28067908 |
| rs7404095 | PRKCB | IBD | GCST004131 | 28067908 |
| rs2382817 | PNKD,TMBIM1 | IBD | GCST004131 | 28067908 |
| rs1801274 | FCGR2A | IBD | GCST004131 | 28067908 |
| rs7746082 | PRDM1,RN7SKP211 | IBD | GCST004131 | 28067908 |
| rs3766606 | PARK7 | IBD | GCST004131 | 28067908 |
| rs17694108 | CEBPA,SLC7A10 | IBD | GCST004131 | 28067908 |
| rs3091316 | CCL2,CCL7 | IBD | GCST004131 | 28067908 |
| rs78534766 | ADCY7 | IBD | GCST004131 | 28067908 |
| rs28449958 | IL27,NUPR1 | IBD | GCST004131 | 28067908 |
| rs921720 | TRIB1AL | IBD | GCST004131 | 28067908 |
| rs1042058 | MAP3K8 | IBD | GCST004131 | 28067908 |
| rs1250550 | ZMIZ1 | IBD | GCST004131 | 28067908 |
| rs34779708 | CREM | IBD | GCST004131 | 28067908 |
| rs11879191 | CDC37 | IBD | GCST004131 | 28067908 |
| rs16940202 | LINC01082,LINC02132 | IBD | GCST004131 | 28067908 |
| rs8005161 | GPR65 | IBD | GCST004131 | 28067908 |
| rs17780256 | SLC39A11 | IBD | GCST004131 | 28067908 |
| rs2488397 | DENND1B | IBD | GCST004131 | 28067908 |
| rs13001325 | IL18R1,IL1RL1 | IBD | GCST004131 | 28067908 |
| rs1456896 | SPMIP7,IKZF1 | IBD | GCST004131 | 28067908 |
| rs925255 | FOSL2S1 | IBD | GCST004131 | 28067908 |
| rs17119 | RNU693P,RPL6P17 | IBD | GCST004131 | 28067908 |
| rs1363907 | ERAP2 | IBD | GCST004131 | 28067908 |
| rs3851228 | TRAF3IP2S1 | IBD | GCST004131 | 28067908 |
| rs395157 | OSMR | IBD | GCST004131 | 28067908 |
| rs6863411 | NDFIP1 | IBD | GCST004131 | 28067908 |
| rs17085007 | RPS21P8,FGFR1OP2P1 | IBD | GCST004131 | 28067908 |
| rs1811711 | CCL20 | IBD | GCST004131 | 28067908 |
| rs12103 | INTS11 | IBD | GCST004131 | 28067908 |
| rs9358372 | CDKAL1 | IBD | GCST004131 | 28067908 |
| rs7495132 | CRTC3,CRTC3S1 | IBD | GCST004131 | 28067908 |
| rs11681525 | TEX41 | IBD | GCST004131 | 28067908 |
| rs13407913 | ADCY3 | IBD | GCST004131 | 28067908 |
| rs941823 | LINC00598 | IBD | GCST004131 | 28067908 |
| rs913678 | RN7SL636P,LINC01271 | IBD | GCST004131 | 28067908 |
| rs71559680 | CDKAL1,LINC00581 | IBD | GCST004131 | 28067908 |
| rs7911264 | HHEX,EIF2S2P3 | IBD | GCST004131 | 28067908 |
| rs6651252 | LINC00824 | IBD | GCST004131 | 28067908 |
| rs10797432 | PRXL2B,TNFRSF14 | IBD | GCST004131 | 28067908 |
| rs907611 | LSP1 | IBD | GCST004131 | 28067908 |
| rs529866 | RMI2 | IBD | GCST004131 | 28067908 |
| rs1991866 | CCDC26 | IBD | GCST004131 | 28067908 |
| rs9557195 | UBAC2,GPR183 | IBD | GCST004131 | 28067908 |
| rs1819658 | IPMK,MRPS35P3 | IBD | GCST004131 | 28067908 |
| rs200349593 | NXPE1 | IBD | GCST004131 | 28067908 |
| rs254560 | PITX1S1 | IBD | GCST004131 | 28067908 |
| rs11230563 | CD6 | IBD | GCST004131 | 28067908 |
| rs1569723 | RPL13P2,CD40 | IBD | GCST004131 | 28067908 |
| rs564349 | ERGIC1 | IBD | GCST004131 | 28067908 |
| rs7954567 | LTBR | IBD | GCST004131 | 28067908 |
| rs4743820 | LINC00484,LINC02937 | IBD | GCST004131 | 28067908 |
| rs13126505 | BANK1 | IBD | GCST004131 | 28067908 |
| rs38904 | ST7,MTND4P6 | IBD | GCST004131 | 28067908 |
| rs516246 | FUT2 | IBD | GCST004131 | 28067908 |
| rs2930047 | DAP | IBD | GCST004131 | 28067908 |
| rs6716753 | SP140 | IBD | GCST004131 | 28067908 |
| rs7011507 | IDI1P2,UBE2V2 | IBD | GCST004131 | 28067908 |
| rs6142618 | TM9SF4 | IBD | GCST004131 | 28067908 |
| rs80244186 | LINC02341 | IBD | GCST004131 | 28067908 |
| rs11168249 | HDAC7 | IBD | GCST004131 | 28067908 |
| rs138788 | TOM1 | IBD | GCST004131 | 28067908 |
| rs2790216 | IPMK | IBD | GCST001725 | 23128233 |
| rs10761659 | ALDH7A1P4,LINC02929 | IBD | GCST001725 | 23128233 |
| rs2227564 | PLAU,C10orf55 | IBD | GCST001725 | 23128233 |
| rs1250546 | ZMIZ1 | IBD | GCST001725 | 23128233 |
| rs6586030 | TSPAN14 | IBD | GCST001725 | 23128233 |
| rs7911264 | HHEX,EIF2S2P3 | IBD | GCST001725 | 23128233 |
| rs4409764 | LINC01475 | IBD | GCST001725 | 23128233 |
| rs907611 | LSP1 | IBD | GCST001725 | 23128233 |
| rs10896794 | LPXN | IBD | GCST001725 | 23128233 |
| rs11230563 | CD6 | IBD | GCST001725 | 23128233 |
| rs4246215 | FADS2,FEN1 | IBD | GCST001725 | 23128233 |
| rs559928 | RPS6KA4,LINC02723 | IBD | GCST001725 | 23128233 |
| rs2231884 | CCDC85B,FIBP | IBD | GCST001725 | 23128233 |
| rs2155219 | EMSY,LINC02757 | IBD | GCST001725 | 23128233 |
| rs6592362 | RNU6135P,PSMA2P1 | IBD | GCST001725 | 23128233 |
| rs630923 | YRNA,CXCR5 | IBD | GCST001725 | 23128233 |
| rs11612508 | DUSP16 | IBD | GCST001725 | 23128233 |
| rs11168249 | HDAC7 | IBD | GCST001725 | 23128233 |
| rs7134599 | IFNGS1 | IBD | GCST001725 | 23128233 |
| rs941823 | LINC00598 | IBD | GCST001725 | 23128233 |
| rs9557195 | UBAC2,GPR183 | IBD | GCST001725 | 23128233 |
| rs194749 | MAGOH3P,ZFP36L1 | IBD | GCST001725 | 23128233 |
| rs8005161 | GPR65 | IBD | GCST001725 | 23128233 |
| rs17293632 | SMAD3 | IBD | GCST001725 | 23128233 |
| rs7495132 | CRTC3,CRTC3S1 | IBD | GCST001725 | 23128233 |
| rs7404095 | PRKCB | IBD | GCST001725 | 23128233 |
| rs26528 | IL27 | IBD | GCST001725 | 23128233 |
| rs10521318 | LINC01082,LINC02132 | IBD | GCST001725 | 23128233 |
| rs12946510 | IKZF3,GRB7 | IBD | GCST001725 | 23128233 |
| rs12942547 | STAT3 | IBD | GCST001725 | 23128233 |
| rs1292053 | TUBD1 | IBD | GCST001725 | 23128233 |
| rs1893217 | PTPN2 | IBD | GCST001725 | 23128233 |
| rs7240004 | CTIF,SMAD7 | IBD | GCST001725 | 23128233 |
| rs727088 | CD226 | IBD | GCST001725 | 23128233 |
| rs11879191 | CDC37 | IBD | GCST001725 | 23128233 |
| rs17694108 | CEBPA,SLC7A10 | IBD | GCST001725 | 23128233 |
| rs11672983 | RNU622P,FCAR | IBD | GCST001725 | 23128233 |
| rs6142618 | TM9SF4 | IBD | GCST001725 | 23128233 |
| rs4911259 | DNMT3B | IBD | GCST001725 | 23128233 |
| rs1569723 | RPL13P2,CD40 | IBD | GCST001725 | 23128233 |
| rs913678 | RN7SL636P,LINC01271 | IBD | GCST001725 | 23128233 |
| rs259964 | ZNF831 | IBD | GCST001725 | 23128233 |
| rs6062504 | ZGPAT | IBD | GCST001725 | 23128233 |
| rs2823286 | LINC02920,CYCSP42 | IBD | GCST001725 | 23128233 |
| rs1363907 | ERAP2 | IBD | GCST001725 | 23128233 |
| rs11741861 | ZNF300,IRGM | IBD | GCST001725 | 23128233 |
| rs9358372 | CDKAL1 | IBD | GCST001725 | 23128233 |
| rs1517352 | STAT4 | IBD | GCST001725 | 23128233 |
| rs2382817 | PNKD,TMBIM1 | IBD | GCST001725 | 23128233 |
| rs3749171 | GPR35 | IBD | GCST001725 | 23128233 |
| rs4256159 | SATB1S1 | IBD | GCST001725 | 23128233 |
| rs3197999 | MST1 | IBD | GCST001725 | 23128233 |
| rs2472649 | CXCL5,PPBP | IBD | GCST001725 | 23128233 |
| rs7657746 | BLTP1 | IBD | GCST001725 | 23128233 |
| rs2930047 | DAP | IBD | GCST001725 | 23128233 |
| rs11742570 | RNU150P,TTC33 | IBD | GCST001725 | 23128233 |
| rs4836519 | HINT1,ARL2BPP4 | IBD | GCST001725 | 23128233 |
| rs2188962 | CARINH | IBD | GCST001725 | 23128233 |
| rs6863411 | NDFIP1 | IBD | GCST001725 | 23128233 |
| rs6871626 | LINC01845,IL12B | IBD | GCST001725 | 23128233 |
| rs12654812 | RGS14 | IBD | GCST001725 | 23128233 |
| rs17119 | RNU693P,RPL6P17 | IBD | GCST001725 | 23128233 |
| rs1847472 | BACH2 | IBD | GCST001725 | 23128233 |
| rs6568421 | PRDM1,RN7SKP211 | IBD | GCST001725 | 23128233 |
| rs6920220 | LINC03004 | IBD | GCST001725 | 23128233 |
| rs12199775 | PHACTR2 | IBD | GCST001725 | 23128233 |
| rs1819333 | RNASET2,MIR3939 | IBD | GCST001725 | 23128233 |
| rs1456896 | SPMIP7,IKZF1 | IBD | GCST001725 | 23128233 |
| rs9297145 | KPNA7,SMURF1 | IBD | GCST001725 | 23128233 |
| rs1734907 | POP7,EPO | IBD | GCST001725 | 23128233 |
| rs38904 | ST7,MTND4P6 | IBD | GCST001725 | 23128233 |
| rs921720 | TRIB1AL | IBD | GCST001725 | 23128233 |
| rs3851228 | TRAF3IP2S1 | IBD | GCST001725 | 23128233 |
| rs11564258 | MUC19 | IBD | GCST001725 | 23128233 |
| rs17085007 | RPS21P8,FGFR1OP2P1 | IBD | GCST001725 | 23128233 |
| rs4899554 | RNU4ATAC14P,U2 | IBD | GCST001725 | 23128233 |
| rs529866 | RMI2 | IBD | GCST001725 | 23128233 |
| rs3091316 | CCL2,CCL7 | IBD | GCST001725 | 23128233 |
| rs11209026 | IL23R | IBD | GCST001725 | 23128233 |
| rs12103 | INTS11 | IBD | GCST001725 | 23128233 |
| rs35675666 | PARK7 | IBD | GCST001725 | 23128233 |
| rs12568930 | ZBTB40,PPIAP34 | IBD | GCST001725 | 23128233 |
| rs2651244 | CHORDC1P5,CTH | IBD | GCST001725 | 23128233 |
| rs4845604 | RORC | IBD | GCST001725 | 23128233 |
| rs670523 | RIT1 | IBD | GCST001725 | 23128233 |
| rs4656958 | ITLN2,ITLN1 | IBD | GCST001725 | 23128233 |
| rs1801274 | FCGR2A | IBD | GCST001725 | 23128233 |
| rs2488389 | DENND1B | IBD | GCST001725 | 23128233 |
| rs7554511 | INAVA | IBD | GCST001725 | 23128233 |
| rs3024505 | IL10,Y_RNA | IBD | GCST001725 | 23128233 |
| rs6545800 | ADCY3 | IBD | GCST001725 | 23128233 |
| rs925255 | FOSL2S1 | IBD | GCST001725 | 23128233 |
| rs10495903 | THADA | IBD | GCST001725 | 23128233 |
| rs7608910 | PUS10 | IBD | GCST001725 | 23128233 |
| rs6740462 | LINC02934 | IBD | GCST001725 | 23128233 |
| rs917997 | SLC9A4,IL18RAP | IBD | GCST001725 | 23128233 |
| rs2111485 | FAP,IFIH1 | IBD | GCST001725 | 23128233 |
| rs1991866 | CCDC26 | IBD | GCST001725 | 23128233 |
| rs10758669 | JAK2,HNRNPA1P41 | IBD | GCST001725 | 23128233 |
| rs4743820 | LINC00484,LINC02937 | IBD | GCST001725 | 23128233 |
| rs4246905 | TNFSF15 | IBD | GCST001725 | 23128233 |
| rs10781499 | CARD9 | IBD | GCST001725 | 23128233 |
| rs12722515 | IL2RA | IBD | GCST001725 | 23128233 |
| rs1042058 | MAP3K8 | IBD | GCST001725 | 23128233 |
| rs11010067 | CUL2,LINC02635 | IBD | GCST001725 | 23128233 |
| rs2836878 | LINC02940,RPL23AP12 | IBD | GCST001725 | 23128233 |
| rs7282490 | GATD3 | IBD | GCST001725 | 23128233 |
| rs2266959 | UBE2L3 | IBD | GCST001725 | 23128233 |
| rs2412970 | HORMAD2 | IBD | GCST001725 | 23128233 |
| rs2413583 | PDGFB,RPL3 | IBD | GCST001725 | 23128233 |
| rs10798069 | PLA2G4A | IBD | GCST003043 | 26192919 |
| rs2189234 | TET2 | IBD | GCST003043 | 26192919 |
| rs12199775 | PHACTR2 | IBD | GCST003043 | 26192919 |
| rs7165170 | CRTC3,CRTC3S1 | IBD | GCST003043 | 26192919 |
| rs16967103 | LINC02694 | IBD | GCST003043 | 26192919 |
| rs3116494 | CD28 | IBD | GCST003043 | 26192919 |
| rs4728142 | KCP,IRF5 | IBD | GCST003043 | 26192919 |
| rs1077773 | LINC02888 | IBD | GCST003043 | 26192919 |
| rs17229285 | PLCL1,RNU747P | IBD | GCST003043 | 26192919 |
| rs11150589 | Y_RNA | IBD | GCST003043 | 26192919 |
| rs4243971 | POFUT1,KIF3B | IBD | GCST003043 | 26192919 |
| rs7438704 | SLAIN2 | IBD | GCST003043 | 26192919 |
| rs490608 | DAP3 | IBD | GCST003043 | 26192919 |
| rs6856616 | LINC02513 | IBD | GCST003043 | 26192919 |
| rs13204048 | SLC22A23 | IBD | GCST003043 | 26192919 |
| rs6667605 | TNFRSF14,PRXL2B | IBD | GCST003043 | 26192919 |
| rs10061469 | LINC02230,TMEM174 | IBD | GCST003043 | 26192919 |
| rs11583043 | DPH5 | IBD | GCST003043 | 26192919 |
| rs11083840 | CALM3,PTGIR | IBD | GCST003043 | 26192919 |
| rs727563 | ACO2 | IBD | GCST003043 | 26192919 |
| rs732072 | RELA | IBD | GCST003043 | 26192919 |
| rs2641348 | ADAM30 | IBD | GCST003043 | 26192919 |
| rs11054935 | DUSP16 | IBD | GCST003043 | 26192919 |
| rs17771967 | KIR3DL2,RNU622P | IBD | GCST003043 | 26192919 |
| rs9525625 | LINC02341 | IBD | GCST003043 | 26192919 |
| rs13300218 | NOTCH1 | IBD | GCST003043 | 26192919 |
| rs72634258 | ERRFI1T | IBD | GCST003043 | 26192919 |
| rs2024092 | SBNO2 | IBD | GCST003043 | 26192919 |
| rs116392568 | HLA,LINC02571 | IBD | GCST003043 | 26192919 |
| rs12627970 | RPL3,SYNGR1 | IBD | GCST003043 | 26192919 |
| rs4676410 | GPR35 | IBD | GCST003043 | 26192919 |
| rs9868809 | CELSR3 | IBD | GCST003043 | 26192919 |
| rs2179070 | TRAF3IP2S1,TRAF3IP2 | IBD | GCST003043 | 26192919 |
| rs4845604 | RORC | IBD | GCST003043 | 26192919 |
| rs444210 | MIR3939,RNASET2 | IBD | GCST003043 | 26192919 |
| rs55808324 | GALC | IBD | GCST003043 | 26192919 |
| rs11641016 | LINC01082,LINC02132 | IBD | GCST003043 | 26192919 |
| rs10956252 | TRIB1AL | IBD | GCST003043 | 26192919 |
| rs12720356 | TYK2 | IBD | GCST003043 | 26192919 |
| rs2488397 | DENND1B | IBD | GCST003043 | 26192919 |
| rs13300483 | DELEC1 | IBD | GCST003043 | 26192919 |
| rs78487399 | THADA | IBD | GCST003043 | 26192919 |
| rs3764147 | LACC1 | IBD | GCST003043 | 26192919 |
| rs7097656 | TSPAN14 | IBD | GCST003043 | 26192919 |
| rs4380874 | PIGCP2,DLD | IBD | GCST003043 | 26192919 |
| rs4768236 | LRRK2 | IBD | GCST003043 | 26192919 |
| rs11677953 | ARPC2,GPBAR1 | IBD | GCST003043 | 26192919 |
| rs2279990 | FOSL2 | IBD | GCST003043 | 26192919 |
| rs1003342 | HORMAD2 | IBD | GCST003043 | 26192919 |
| rs1842076 | KRT18P56,LINC00604 | IBD | GCST003043 | 26192919 |
| rs1363907 | ERAP2 | IBD | GCST003043 | 26192919 |
| rs6933404 | BTF3L4P3,LINC03004 | IBD | GCST003043 | 26192919 |
| rs2395022 | KPNA7,SMURF1 | IBD | GCST003043 | 26192919 |
| rs1260326 | GCKR | IBD | GCST003043 | 26192919 |
| rs11641184 | LITAF | IBD | GCST003043 | 26192919 |
| rs17694108 | CEBPA,SLC7A10 | IBD | GCST003043 | 26192919 |
| rs6920220 | LINC03004 | IBD | GCST003043 | 26192919 |
| rs11230563 | CD6 | IBD | GCST003043 | 26192919 |
| rs449454 | NDFIP1 | IBD | GCST003043 | 26192919 |
| rs3776414 | DAP | IBD | GCST003043 | 26192919 |
| rs12718244 | SPMIP7 | IBD | GCST003043 | 26192919 |
| rs1517352 | STAT4 | IBD | GCST003043 | 26192919 |
| rs6708413 | IL18RAP | IBD | GCST003043 | 26192919 |
| rs516246 | FUT2 | IBD | GCST003043 | 26192919 |
| rs2328546 | CDKAL1 | IBD | GCST003043 | 26192919 |
| rs7657746 | BLTP1 | IBD | GCST003043 | 26192919 |
| rs2266961 | UBE2L3 | IBD | GCST003043 | 26192919 |
| rs559928 | RPS6KA4,LINC02723 | IBD | GCST003043 | 26192919 |
| rs1456896 | SPMIP7,IKZF1 | IBD | GCST003043 | 26192919 |
| rs9557207 | UBAC2 | IBD | GCST003043 | 26192919 |
| rs941823 | LINC00598 | IBD | GCST003043 | 26192919 |
| rs3742130 | UBAC2,GPR18 | IBD | GCST003043 | 26192919 |
| rs1292053 | TUBD1 | IBD | GCST003043 | 26192919 |
| rs4976646 | RGS14 | IBD | GCST003043 | 26192919 |
| rs12722515 | IL2RA | IBD | GCST003043 | 26192919 |
| rs2816958 | NR5A2 | IBD | GCST003043 | 26192919 |
| rs314313 | EPHB4 | IBD | GCST003043 | 26192919 |
| rs13204742 | PTPRK,THEMIS | IBD | GCST003043 | 26192919 |
| rs1847472 | BACH2 | IBD | GCST003043 | 26192919 |
| rs3853824 | C17orf67 | IBD | GCST003043 | 26192919 |
| rs254560 | PITX1S1 | IBD | GCST003043 | 26192919 |
| rs6651252 | LINC00824 | IBD | GCST003043 | 26192919 |
| rs1182188 | GNA12 | IBD | GCST003043 | 26192919 |
| rs3184504 | ATXN2,SH2B3 | IBD | GCST003043 | 26192919 |
| rs561722 | NXPE2P1,NXPE1 | IBD | GCST003043 | 26192919 |
| rs17207042 | CD226 | IBD | GCST003043 | 26192919 |
| rs11681525 | TEX41 | IBD | GCST003043 | 26192919 |
| rs7555082 | ATP6V1G3,PTPRC | IBD | GCST003043 | 26192919 |
| rs1535 | FADS2 | IBD | GCST003043 | 26192919 |
| rs915286 | LINC00598 | IBD | GCST003043 | 26192919 |
| rs1569328 | FOS,U2 | IBD | GCST003043 | 26192919 |
| rs4743820 | LINC00484,LINC02937 | IBD | GCST003043 | 26192919 |
| rs17061048 | LINC00598 | IBD | GCST003043 | 26192919 |
| rs2284553 | IFNGR2 | IBD | GCST003043 | 26192919 |
| rs7773324 | DUSP22,IRF4 | IBD | GCST003043 | 26192919 |
| rs6740462 | LINC02934 | IBD | GCST003043 | 26192919 |
| rs259964 | ZNF831 | IBD | GCST003043 | 26192919 |
| rs2153283 | IPMK | IBD | GCST003043 | 26192919 |
| rs10142466 | MAGOH3P,ZFP36L1 | IBD | GCST003043 | 26192919 |
| rs4802307 | PPP5C,HIF3A | IBD | GCST003043 | 26192919 |
| rs7011507 | IDI1P2,UBE2V2 | IBD | GCST003043 | 26192919 |
| rs6025 | F5 | IBD | GCST003043 | 26192919 |
| rs4664304 | PLA2R1 | IBD | GCST003043 | 26192919 |
| rs6058869 | COMMD7,DNMT3B | IBD | GCST003043 | 26192919 |
| rs2945412 | KSR1 | IBD | GCST003043 | 26192919 |
| rs7015630 | PARAIL | IBD | GCST003043 | 26192919 |
| rs7404095 | PRKCB | IBD | GCST003043 | 26192919 |
| rs113010081 | CCRL2,LINC02009 | IBD | GCST003043 | 26192919 |
| rs17057051 | PTK2B | IBD | GCST003043 | 26192919 |
| rs11064881 | CIT | IBD | GCST003043 | 26192919 |
| rs483905 | MAML2 | IBD | GCST003043 | 26192919 |
| rs10065637 | ANKRD55 | IBD | GCST003043 | 26192919 |
| rs7517810 | AIMP1P2,SLC25A38P1 | IBD | GCST003043 | 26192919 |
| rs7758080 | TAB2S1,TAB2 | IBD | GCST003043 | 26192919 |
| rs13277237 | CCDC26 | IBD | GCST003043 | 26192919 |
| rs2073505 | HGFAC | IBD | GCST003043 | 26192919 |
| rs564349 | ERGIC1 | IBD | GCST003043 | 26192919 |
| rs212388 | TAGAP,TAGAPS1 | IBD | GCST003043 | 26192919 |
| rs10486483 | SKAP2 | IBD | GCST003043 | 26192919 |
| rs2538470 | RN7SL72P,RPL32P17 | IBD | GCST003043 | 26192919 |
| rs17780256 | SLC39A11 | IBD | GCST003043 | 26192919 |
| rs12103 | INTS11 | IBD | GCST003043 | 26192919 |
| rs12585310 | FGFR1OP2P1,RPS21P8 | IBD | GCST003043 | 26192919 |
| rs913678 | RN7SL636P,LINC01271 | IBD | GCST003043 | 26192919 |
| rs4703855 | PTCD2,YBX1P5 | IBD | GCST003043 | 26192919 |
| rs11708026 | SATB1S1 | IBD | GCST003043 | 26192919 |
| rs6074022 | CD40,RPL13P2 | IBD | GCST003043 | 26192919 |
| rs13126505 | BANK1 | IBD | GCST003043 | 26192919 |
| rs7240004 | CTIF,SMAD7 | IBD | GCST003043 | 26192919 |
| rs6716753 | SP140 | IBD | GCST003043 | 26192919 |
| rs34920465 | ZBTB40,PPIAP34 | IBD | GCST003043 | 26192919 |
| rs423674 | RMI2 | IBD | GCST003043 | 26192919 |
| rs11187157 | EXOC6,Y_RNA | IBD | GCST003043 | 26192919 |
| rs7556897 | SNRPGP8,CCL20 | IBD | GCST003043 | 26192919 |
| rs71559680 | CDKAL1,LINC00581 | IBD | GCST003043 | 26192919 |
| rs2688608 | CAMK2G,C10orf55 | IBD | GCST003043 | 26192919 |
| rs1990760 | IFIH1 | IBD | GCST003043 | 26192919 |
| rs11229555 | GLYAT | IBD | GCST003043 | 26192919 |
| rs9358372 | CDKAL1 | IBD | GCST003043 | 26192919 |
| rs2274351 | SUFU | IBD | GCST003043 | 26192919 |
| rs10051722 | HINT1,ARL2BPP4 | IBD | GCST003043 | 26192919 |
| rs34856868 | BTBD8 | IBD | GCST003043 | 26192919 |
| rs2050392 | NIFKP1,CCND3P1 | IBD | GCST003043 | 26192919 |
| rs2297559 | ITLN1 | IBD | GCST003043 | 26192919 |
| rs17119 | RNU693P,RPL6P17 | IBD | GCST003043 | 26192919 |
| rs653178 | ATXN2 | IBD | GCST003043 | 26192919 |
| rs4692386 | LINC02357,RBPJ | IBD | GCST003043 | 26192919 |
| rs7236492 | NFATC1 | IBD | GCST003043 | 26192919 |
| rs72810983 | CPEB4 | IBD | GCST003043 | 26192919 |
| rs907611 | LSP1 | IBD | GCST003043 | 26192919 |
| rs4812833 | LINC01620 | IBD | GCST003043 | 26192919 |
| rs7547569 | RNU4ATAC4P,IL23R | IBD | GCST003043 | 26192919 |
| rs6880778 | RNU150P,TTC33 | IBD | GCST003043 | 26192919 |
| rs10748781 | LINC01475 | IBD | GCST003043 | 26192919 |
| rs113653754 | HLAQB1,HLAQA1 | IBD | GCST003043 | 26192919 |
| rs4077515 | CARD9 | IBD | GCST003043 | 26192919 |
| rs10761659 | ALDH7A1P4,LINC02929 | IBD | GCST003043 | 26192919 |
| rs9836291 | BSN | IBD | GCST003043 | 26192919 |
| rs11236797 | LINC02757,EMSY | IBD | GCST003043 | 26192919 |
| rs3024493 | IL10 | IBD | GCST003043 | 26192919 |
| rs56167332 | IL12B,LINC01845 | IBD | GCST003043 | 26192919 |
| rs2836883 | RPL23AP12,LINC02940 | IBD | GCST003043 | 26192919 |
| rs75900472 | HNRNPA1P41,JAK2 | IBD | GCST003043 | 26192919 |
| rs35730213 | INAVA | IBD | GCST003043 | 26192919 |
| rs4795397 | ZPBP2,IKZF3 | IBD | GCST003043 | 26192919 |
| rs56399423 | SLC22A4,MIR3936HG | IBD | GCST003043 | 26192919 |
| rs17622378 | CARINH,IRF1 | IBD | GCST003043 | 26192919 |
| rs12994997 | ATG16L1 | IBD | GCST003043 | 26192919 |
| rs12946510 | IKZF3,GRB7 | IBD | GCST003043 | 26192919 |
| rs2143178 | RPL3,PDGFB | IBD | GCST003043 | 26192919 |
| rs10800309 | FCGR2A,RNU681P | IBD | GCST003043 | 26192919 |
| rs7608910 | PUS10 | IBD | GCST003043 | 26192919 |
| rs6426833 | RNF186S1,OTUD3 | IBD | GCST003043 | 26192919 |
| rs1801274 | FCGR2A | IBD | GCST003043 | 26192919 |
| rs7848647 | DELEC1,TNFSF15 | IBD | GCST003043 | 26192919 |
| rs6062496 | TNFRSF6B,RTEL1NFRSF6B | IBD | GCST003043 | 26192919 |
| rs6556412 | IL12B,LINC01845 | IBD | GCST003043 | 26192919 |
| rs1505992 | TTC33,RNU150P | IBD | GCST003043 | 26192919 |
| rs17800987 | ZNF300P1 | IBD | GCST003043 | 26192919 |
| rs1297258 | CYCSP42,LINC02920 | IBD | GCST003043 | 26192919 |
| rs8127691 | GATD3 | IBD | GCST003043 | 26192919 |
| rs2847278 | LINC01882,PTPN2 | IBD | GCST003043 | 26192919 |
| rs1893217 | PTPN2 | IBD | GCST003043 | 26192919 |
| rs12318183 | IFNGS1 | IBD | GCST003043 | 26192919 |
| rs9313808 | LINC01845,IL12B | IBD | GCST003043 | 26192919 |
| rs34779708 | CREM | IBD | GCST003043 | 26192919 |
| rs11010067 | CUL2,LINC02635 | IBD | GCST003043 | 26192919 |
| rs11743851 | CDC42SE2 | IBD | GCST003043 | 26192919 |
| rs744166 | STAT3 | IBD | GCST003043 | 26192919 |
| rs4946717 | PRDM1 | IBD | GCST003043 | 26192919 |
| rs62037363 | SH2B1 | IBD | GCST003043 | 26192919 |
| rs1388585 | SLC2A13,LINC02555 | IBD | GCST003043 | 26192919 |
| rs26528 | IL27 | IBD | GCST003043 | 26192919 |
| rs11554257 | TNFSF15,DELEC1 | IBD | GCST003043 | 26192919 |
| rs9889296 | CCL2,LINC01989 | IBD | GCST003043 | 26192919 |
| rs13407913 | ADCY3 | IBD | GCST003043 | 26192919 |
| rs1420098 | IL18R1 | IBD | GCST003043 | 26192919 |
| rs395157 | OSMR | IBD | GCST003043 | 26192919 |
| rs35164067 | CDC37 | IBD | GCST003043 | 26192919 |
| rs17293632 | SMAD3 | IBD | GCST003043 | 26192919 |
| rs3091315 | CCL7,CCL2 | IBD | GCST003043 | 26192919 |
| rs1250566 | ZMIZ1 | IBD | GCST003043 | 26192919 |
| rs2315008 | ZGPAT | IBD | GCST000225 | 18758464 |
| rs5743289 | NOD2 | IBD | GCST000225 | 18758464 |
| rs477515 | HLAQA1,HLARB1 | IBD | GCST000225 | 18758464 |
| rs6478109 | TNFSF15,DELEC1 | IBD | GCST000225 | 18758464 |
| rs2836878 | LINC02940,RPL23AP12 | IBD | GCST000225 | 18758464 |
| rs11209026 | IL23R | IBD | GCST000225 | 18758464 |
| rs2076756 | NOD2 | IBD | GCST000008 | 17068223 |
| rs6856616 | LINC02513 | IBD | GCST003602 | 27569725 |
| rs1819333 | RNASET2,MIR3939 | IBD | GCST003602 | 27569725 |
| rs3766920 | SHC1,PYGO2,PYGO2S1 | IBD | GCST003602 | 27569725 |
| rs16953946 | CDYL2 | IBD | GCST003602 | 27569725 |
| rs4821558 | NCF4,CSF2RB | IBD | GCST003602 | 27569725 |
| rs11788518 | PCSK5 | IBD | GCST003602 | 27569725 |
| rs3731257 | CDKN2AS1 | IBD | GCST003602 | 27569725 |
| rs1321366 | RFX6 | IBD | GCST003602 | 27569725 |
| rs1816854 | TWF1,TMEM117 | IBD | GCST003602 | 27569725 |
| rs7973572 | SOX5 | IBD | GCST003602 | 27569725 |
| rs6740462 | LINC02934 | IBD | GCST003602 | 27569725 |
| rs13300483 | DELEC1 | IBD | GCST003602 | 27569725 |
| rs12942547 | STAT3 | IBD | GCST003602 | 27569725 |
| rs6677524 | PLXNA2,LINC01735 | IBD | GCST003602 | 27569725 |
| rs3798544 | SPDEF | IBD | GCST003602 | 27569725 |
| rs35990859 | IL12B,LINC01845 | IBD | GCST008731 | 26278503 |
| rs730086 | KAT2A | IBD | GCST008731 | 26278503 |
| rs7224339 | STAT3 | IBD | GCST008731 | 26278503 |
| rs77894461 | IFFO2,UBR4 | IBD | GCST90020070 | 35232999 |
| rs6660226 | DNAJB6P4,IL12RB2 | IBD | GCST90020070 | 35232999 |
| rs12185578 | PSMC1P10,RAD51AP2 | IBD | GCST90020070 | 35232999 |
| rs62266031 | LSAMP | IBD | GCST90020070 | 35232999 |
| rs2030413 | BTNL12P | IBD | GCST90020070 | 35232999 |
| rs3121685 | LINC02229,LINC02065 | IBD | GCST90020070 | 35232999 |
| rs1998136 | TC2N | IBD | GCST90020070 | 35232999 |
| rs6702829 | LINC01648,LINC01756 | IBD | GCST90020070 | 35232999 |
| rs2052483 | NUDT12,NIHCOLE | IBD | GCST90020070 | 35232999 |
| rs10515625 | ABLIM3,AFAP1L1 | IBD | GCST90020070 | 35232999 |
| rs6946352 | BZW2 | IBD | GCST90020070 | 35232999 |
| rs10119004 | JAK2 | IBD | GCST90020070 | 35232999 |
| rs1826333 | HSP90AA2P,CBX3P1 | IBD | GCST90020070 | 35232999 |
| rs2806899 | SIAH3,ZC3H13 | IBD | GCST90020070 | 35232999 |
| rs12568930 | ZBTB40,PPIAP34 | IBD | GCST011501 | 33608531 |
| rs11581607 | IL23R | IBD | GCST011501 | 33608531 |
| rs4551125 | RNU150P,TTC33 | IBD | GCST011501 | 33608531 |
| rs55722650 | P4HA2,PDLIM4 | IBD | GCST011501 | 33608531 |
| rs148844907 | C6orf47S1,C6orf47 | IBD | GCST011501 | 33608531 |
| rs17264332 | LINC03004 | IBD | GCST011501 | 33608531 |
| rs6961243 | DLD,PIGCP2 | IBD | GCST011501 | 33608531 |
| rs142738614 | IRF5 | IBD | GCST011501 | 33608531 |
| rs10761659 | ALDH7A1P4,LINC02929 | IBD | GCST011501 | 33608531 |
| rs73370726 | CH25H,MIR4679 | IBD | GCST011501 | 33608531 |
| rs11403745 | LINC01475,GOT1T | IBD | GCST011501 | 33608531 |
| rs7936312 | EMSY,LINC02757 | IBD | GCST011501 | 33608531 |
| rs35788599 | IFNGS1 | IBD | GCST011501 | 33608531 |
| rs2066847 | CYLDS1,NOD2 | IBD | GCST011501 | 33608531 |
| rs12720356 | TYK2 | IBD | GCST011501 | 33608531 |
| rs6017342 | LINC01620 | IBD | GCST011501 | 33608531 |
| rs1297261 | CYCSP42,LINC02920 | IBD | GCST011501 | 33608531 |
| rs2836878 | LINC02940,RPL23AP12 | IBD | GCST011501 | 33608531 |
| rs59998884 | GATD3 | IBD | GCST011501 | 33608531 |
| rs1521186 | RORC | IBD | GCST011501 | 33608531 |
| rs10737482 | RNF186S1,OTUD3 | IBD | GCST011501 | 33608531 |
| rs1521186 | RORC | IBD | GCST011501 | 33608531 |
| rs6671847 | FCGR2A | IBD | GCST011501 | 33608531 |
| rs905634 | INAVA,MROH3P | IBD | GCST011501 | 33608531 |
| rs3024505 | IL10,Y_RNA | IBD | GCST011501 | 33608531 |
| rs10188217 | PUS10 | IBD | GCST011501 | 33608531 |
| rs13384671 | ITGA4,LINC01934 | IBD | GCST011501 | 33608531 |
| rs73370726 | CH25H,MIR4679 | IBD | GCST011501 | 33608531 |
| rs957100 | TTC33,RNU150P | IBD | GCST90093115 | 33600772 |
| rs1292053 | TUBD1 | IBD | GCST90432182 | 37156999 |
| rs6505765 | PTPN2,LINC01882 | IBD | GCST90432182 | 37156999 |
| rs2112801 | CEBPA,SLC7A10 | IBD | GCST90432182 | 37156999 |
| rs2071699 | FUT1 | IBD | GCST90432182 | 37156999 |
| rs6062496 | TNFRSF6B,RTEL1NFRSF6B | IBD | GCST90432182 | 37156999 |
| rs6517432 | CYCSP42,LINC02920 | IBD | GCST90432182 | 37156999 |
| rs2298428 | YDJC | IBD | GCST90432182 | 37156999 |
| rs2143178 | RPL3,PDGFB | IBD | GCST90432182 | 37156999 |
| rs1535903 | FAM216B,LINC01050 | IBD | GCST90292538 | 37156999 |
| rs1373904 | NRAD1,LACC1 | IBD | GCST90292538 | 37156999 |
| rs2026029 | FNDC3A | IBD | GCST90292538 | 37156999 |
| rs184429843 | NEK5,ALG11 | IBD | GCST90292538 | 37156999 |
| rs9585037 | UBAC2 | IBD | GCST90292538 | 37156999 |
| rs743228 | RPLP0P3,NFKBIA | IBD | GCST90292538 | 37156999 |
| rs4902367 | LINC02324,RNU24P | IBD | GCST90292538 | 37156999 |
| rs194746 | MAGOH3P,ZFP36L1 | IBD | GCST90292538 | 37156999 |
| rs1569328 | FOS,U2 | IBD | GCST90292538 | 37156999 |
| rs4462528 | GALC | IBD | GCST90292538 | 37156999 |
| rs11627111 | ELK2AP,IGHA1 | IBD | GCST90292538 | 37156999 |
| rs11071559 | RORA | IBD | GCST90292538 | 37156999 |
| rs56375023 | SMAD3 | IBD | GCST90292538 | 37156999 |
| rs12440543 | ABHD17C,RNU680P | IBD | GCST90292538 | 37156999 |
| rs71407313 | CRTC3 | IBD | GCST90292538 | 37156999 |
| rs191241541 | PKD1 | IBD | GCST90292538 | 37156999 |
| rs11641184 | LITAF | IBD | GCST90292538 | 37156999 |
| rs59790099 | PRKCB | IBD | GCST90292538 | 37156999 |
| rs201121732 | IL4R,IL21R | IBD | GCST90292538 | 37156999 |
| rs4787458 | IL27,NUPR1 | IBD | GCST90292538 | 37156999 |
| rs5743289 | NOD2 | IBD | GCST90292538 | 37156999 |
| rs6499186 | CDH3,ZFP90 | IBD | GCST90292538 | 37156999 |
| rs8055490 | TMEM170A | IBD | GCST90292538 | 37156999 |
| rs9938250 | WWOX | IBD | GCST90292538 | 37156999 |
| rs187975120 | CDYL2,ARLNC1 | IBD | GCST90292538 | 37156999 |
| rs4256018 | FERMT1 | IBD | GCST90292538 | 37156999 |
| rs875519 | NOL4L | IBD | GCST90292538 | 37156999 |
| rs6426833 | RNF186S1,OTUD3 | IBD | GCST90292538 | 37156999 |
| rs34963268 | ZBTB40,PPIAP34 | IBD | GCST90292538 | 37156999 |
| rs7525903 | MIR4425,RUNX3 | IBD | GCST90292538 | 37156999 |
| rs140466198 | ARL8BP2,RNU676P | IBD | GCST90292538 | 37156999 |
| rs12138864 | PHC2 | IBD | GCST90292538 | 37156999 |
| rs6687307 | PLPP3,LINC01767 | IBD | GCST90292538 | 37156999 |
| rs11581607 | IL23R | IBD | GCST90292538 | 37156999 |
| rs11165441 | TGFBR3 | IBD | GCST90292538 | 37156999 |
| rs3767964 | DR1 | IBD | GCST90292538 | 37156999 |
| rs17572009 | SLC25A24,VAV3S1 | IBD | GCST90292538 | 37156999 |
| rs116062645 | NOTCH2 | IBD | GCST90292538 | 37156999 |
| rs1336900 | HORMAD1 | IBD | GCST90292538 | 37156999 |
| rs11204894 | RORC | IBD | GCST90292538 | 37156999 |
| rs3766920 | SHC1,PYGO2,PYGO2S1 | IBD | GCST90292538 | 37156999 |
| rs34687326 | SLAMF8 | IBD | GCST90292538 | 37156999 |
| rs6658353 | RNU681P,FCGR2A | IBD | GCST90292538 | 37156999 |
| rs6025 | F5 | IBD | GCST90292538 | 37156999 |
| rs10912488 | SLC25A38P1,AIMP1P2 | IBD | GCST90292538 | 37156999 |
| rs72709461 | ABL2 | IBD | GCST90292538 | 37156999 |
| rs2224873 | DENND1B | IBD | GCST90292538 | 37156999 |
| rs74359027 | PTPRC | IBD | GCST90292538 | 37156999 |
| rs2816980 | NR5A2 | IBD | GCST90292538 | 37156999 |
| rs35730213 | INAVA | IBD | GCST90292538 | 37156999 |
| rs11240504 | CDK18 | IBD | GCST90292538 | 37156999 |
| rs3024495 | IL10 | IBD | GCST90292538 | 37156999 |
| rs2073486 | IRF6 | IBD | GCST90292538 | 37156999 |
| rs11582528 | PSEN2,RPS27P5 | IBD | GCST90292538 | 37156999 |
| rs10910476 | IRF2BP2,U8 | IBD | GCST90292538 | 37156999 |
| rs7578575 | DNMT3A | IBD | GCST90292538 | 37156999 |
| rs1260326 | GCKR | IBD | GCST90292538 | 37156999 |
| rs12478126 | PLB1,FOSL2 | IBD | GCST90292538 | 37156999 |
| rs78487399 | THADA | IBD | GCST90292538 | 37156999 |
| rs67927699 | PUS10 | IBD | GCST90292538 | 37156999 |
| rs1370394 | LINC02934 | IBD | GCST90292538 | 37156999 |
| rs1882348 | IL18R1 | IBD | GCST90292538 | 37156999 |
| rs72837826 | MIR4435HG | IBD | GCST90292538 | 37156999 |
| rs55678466 | BIN1 | IBD | GCST90292538 | 37156999 |
| rs56862595 | TEX41 | IBD | GCST90292538 | 37156999 |
| rs2111485 | FAP,IFIH1 | IBD | GCST90292538 | 37156999 |
| rs2124440 | ITGA4 | IBD | GCST90292538 | 37156999 |
| rs2595392 | ITGAV | IBD | GCST90292538 | 37156999 |
| rs142152795 | GLS,NAB1 | IBD | GCST90292538 | 37156999 |
| rs28468423 | CELA3B | IBD | GCST90432182 | 37156999 |
| rs76418789 | IL23R,C1orf141 | IBD | GCST90432182 | 37156999 |
| rs3766920 | SHC1,PYGO2,PYGO2S1 | IBD | GCST90432182 | 37156999 |
| rs147935920 | SPRED2 | IBD | GCST90432182 | 37156999 |
| rs34020101 | IL18RAP | IBD | GCST90432182 | 37156999 |
| rs3769684 | CD28 | IBD | GCST90432182 | 37156999 |
| rs2953155 | GPR35 | IBD | GCST90432182 | 37156999 |
| rs796417 | RDUR | IBD | GCST90432182 | 37156999 |
| rs6071961 | MAFB,LINC01370 | IBD | GCST90292538 | 37156999 |
| rs17181845 | ZHX3 | IBD | GCST90292538 | 37156999 |
| rs6017342 | LINC01620 | IBD | GCST90292538 | 37156999 |
| rs1883832 | CD40 | IBD | GCST90292538 | 37156999 |
| rs913678 | RN7SL636P,LINC01271 | IBD | GCST90292538 | 37156999 |
| rs259964 | ZNF831 | IBD | GCST90292538 | 37156999 |
| rs6062496 | TNFRSF6B,RTEL1NFRSF6B | IBD | GCST90292538 | 37156999 |
| rs7277261 | LINC02920,CYCSP42 | IBD | GCST90292538 | 37156999 |
| rs2284553 | IFNGR2 | IBD | GCST90292538 | 37156999 |
| rs2836882 | LINC02940,RPL23AP12 | IBD | GCST90292538 | 37156999 |
| rs13047030 | TRAPPC10 | IBD | GCST90292538 | 37156999 |
| rs12158299 | YDJC,UBE2L3 | IBD | GCST90292538 | 37156999 |
| rs5761654 | CRYBA4,ISCA2P1 | IBD | GCST90292538 | 37156999 |
| rs1003342 | HORMAD2 | IBD | GCST90292538 | 37156999 |
| rs4821382 | RNU767P,LINC01399 | IBD | GCST90292538 | 37156999 |
| rs2413430 | CSF2RB,NCF4 | IBD | GCST90292538 | 37156999 |
| rs9611131 | PDGFB,RPL3 | IBD | GCST90292538 | 37156999 |
| rs5771192 | IL17REL | IBD | GCST90292538 | 37156999 |
| rs2427870 | Y_RNA,RNU620P | IBD | GCST90292538 | 37156999 |
| rs34119476 | PLCL1,RNU747P | IBD | GCST90292538 | 37156999 |
| rs9726836 | CCNL2 | IBD | GCST90292538 | 37156999 |
| rs1886730 | TNFRSF14 | IBD | GCST90292538 | 37156999 |
| rs225131 | ERRFI1T | IBD | GCST90292538 | 37156999 |
| rs73243351 | LINC02513 | IBD | GCST90432182 | 37156999 |
| rs147909357 | IL2,IL21 | IBD | GCST90432182 | 37156999 |
| rs254560 | PITX1S1 | IBD | GCST90432182 | 37156999 |
| rs11741861 | ZNF300,IRGM | IBD | GCST90432182 | 37156999 |
| rs56167332 | IL12B,LINC01845 | IBD | GCST90432182 | 37156999 |
| rs490928 | ERGIC1 | IBD | GCST90432182 | 37156999 |
| rs6941485 | LINC03066,DUSP22 | IBD | GCST90432182 | 37156999 |
| rs9270984 | HLARB1,HLAQA1 | IBD | GCST90432182 | 37156999 |
| rs77992257 | COX19,ADAP1 | IBD | GCST90432182 | 37156999 |
| rs876036 | SPMIP7,IKZF1 | IBD | GCST90432182 | 37156999 |
| rs10817678 | DELEC1,TNFSF15 | IBD | GCST90432182 | 37156999 |
| rs224136 | ALDH7A1P4,LINC02929 | IBD | GCST90432182 | 37156999 |
| rs1250566 | ZMIZ1 | IBD | GCST90432182 | 37156999 |
| rs10786557 | LINC01475 | IBD | GCST90432182 | 37156999 |
| rs174534 | TMEM258,MYRF | IBD | GCST90432182 | 37156999 |
| rs3176905 | CXCR5 | IBD | GCST90432182 | 37156999 |
| rs17630801 | ELF1 | IBD | GCST90432182 | 37156999 |
| rs2233408 | DNAJC8P1,NFKBIA | IBD | GCST90432182 | 37156999 |
| rs4645854 | FOS | IBD | GCST90432182 | 37156999 |
| rs1988959 | HISLA | IBD | GCST90432182 | 37156999 |
| rs12928665 | CIITA | IBD | GCST90432182 | 37156999 |
| rs9932278 | LITAF | IBD | GCST90432182 | 37156999 |
| rs73583946 | CDYL2 | IBD | GCST90432182 | 37156999 |
| rs16940186 | LINC01082,LINC02132 | IBD | GCST90432182 | 37156999 |
| rs9897389 | STAT3 | IBD | GCST90432182 | 37156999 |
| rs6710043 | PUS10 | IBD | GCST90503485 | 39792054 |
| rs2129944 | CDC37 | IBD | GCST90503485 | 39792054 |
| rs2698193 | PUS10 | IBD | GCST90503485 | 39792054 |
| rs1420101 | IL1RL1,IL18R1 | IBD | GCST90503485 | 39792054 |
| rs10175585 | IL18RAP,SLC9A4 | IBD | GCST90503485 | 39792054 |
| rs6712638 | SLC9A4 | IBD | GCST90503485 | 39792054 |
| rs6431270 | SAG,ATG16L1 | IBD | GCST90503485 | 39792054 |
| rs13005285 | ATG16L1 | IBD | GCST90503485 | 39792054 |
| rs10172939 | SAG | IBD | GCST90503485 | 39792054 |
| rs10412166 | CEBPA,SLC7A10 | IBD | GCST90503485 | 39792054 |
| rs11891546 | SAG | IBD | GCST90503485 | 39792054 |
| rs59930110 | RNF186S1,OTUD3 | IBD | GCST90503485 | 39792054 |
| rs4676410 | GPR35 | IBD | GCST90503485 | 39792054 |
| rs76747262 | IP6K2 | IBD | GCST90503485 | 39792054 |
| rs7631908 | RHOA | IBD | GCST90503485 | 39792054 |
| rs80033912 | RNA5SP130,NICN1 | IBD | GCST90503485 | 39792054 |
| rs59805578 | SLC35D1,C1orf141 | IBD | GCST90503485 | 39792054 |
| rs4855881 | APEH | IBD | GCST90503485 | 39792054 |
| rs56116382 | BSN | IBD | GCST90503485 | 39792054 |
| rs2247036 | TRAIP | IBD | GCST90503485 | 39792054 |
| rs36022378 | ACTL11P | IBD | GCST90503485 | 39792054 |
| rs3796386 | CAMKV | IBD | GCST90503485 | 39792054 |
| rs7428430 | SEMA3FS1 | IBD | GCST90503485 | 39792054 |
| rs77138986 | C1orf141,IL23R | IBD | GCST90503485 | 39792054 |
| rs164765 | KRT18P56,LINC00604 | IBD | GCST90503485 | 39792054 |
| rs7247893 | CDC37 | IBD | GCST90503485 | 39792054 |
| rs12712140 | IL18R1,IL1RL1 | IBD | GCST90503485 | 39792054 |
| rs142770866 | CDC37 | IBD | GCST90503485 | 39792054 |
| rs10416073 | S1PR5 | IBD | GCST90503485 | 39792054 |
| rs62131887 | KEAP1,PDE4A | IBD | GCST90503485 | 39792054 |
| rs11669923 | SLC7A10,CEBPA | IBD | GCST90503485 | 39792054 |
| rs55803162 | SLC7A10,CEBPA | IBD | GCST90503485 | 39792054 |
| rs62126615 | SLC7A10,CEBPA | IBD | GCST90503485 | 39792054 |
| rs13028996 | SAG | IBD | GCST90503485 | 39792054 |
| rs72634258 | ERRFI1T | IBD | GCST90503485 | 39792054 |
| rs4676408 | GPR35,AQP12B | IBD | GCST90503485 | 39792054 |
| rs113985555 | OTUD3 | IBD | GCST90503485 | 39792054 |
| rs115064659 | MIER1 | IBD | GCST90503485 | 39792054 |
| rs182550937 | SLC35D1 | IBD | GCST90503485 | 39792054 |
| rs9850465 | UBA7,TRAIP | IBD | GCST90503485 | 39792054 |
| rs113181267 | C1orf141 | IBD | GCST90503485 | 39792054 |
| rs72676041 | C1orf141 | IBD | GCST90503485 | 39792054 |
| rs137965004 | C1orf141,IL23R | IBD | GCST90503485 | 39792054 |
| rs7540900 | C1orf141 | IBD | GCST90503485 | 39792054 |
| rs45528737 | BLTP1 | IBD | GCST90503485 | 39792054 |
| rs72678531 | IL12RB2 | IBD | GCST90503485 | 39792054 |
| rs11209043 | IL12RB2,DNAJB6P4 | IBD | GCST90503485 | 39792054 |
| rs112149646 | IL23R,C1orf141 | IBD | GCST90503485 | 39792054 |
| rs4655709 | LINC01702,SERBP1 | IBD | GCST90503485 | 39792054 |
| rs11590963 | LINC01702,SERBP1 | IBD | GCST90503485 | 39792054 |
| rs1250573 | ZMIZ1 | IBD | GCST90503485 | 39792054 |
| rs888208 | NKX2 | IBD | GCST90503485 | 39792054 |
| rs117436764 | ADO,ALDH7A1P4 | IBD | GCST90503485 | 39792054 |
| rs4590800 | NKX2,SLC25A28 | IBD | GCST90503485 | 39792054 |
| rs7936070 | LINC02757,EMSY | IBD | GCST90503485 | 39792054 |
| rs7938856 | LINC02757,EMSY | IBD | GCST90503485 | 39792054 |
| rs139518863 | SLC2A13 | IBD | GCST90503485 | 39792054 |
| rs10883374 | SLC25A28,NKX2 | IBD | GCST90503485 | 39792054 |
| rs10786560 | SLC25A28,NKX2 | IBD | GCST90503485 | 39792054 |
| rs11236795 | EMSY,LINC02757 | IBD | GCST90503485 | 39792054 |
| rs10899231 | EMSY,LINC02757 | IBD | GCST90503485 | 39792054 |
| rs17444868 | MUC19 | IBD | GCST90503485 | 39792054 |
| rs74966863 | MUC19 | IBD | GCST90503485 | 39792054 |
| rs112753079 | MUC19 | IBD | GCST90503485 | 39792054 |
| rs117136834 | LINC00598 | IBD | GCST90503485 | 39792054 |
| rs722748 | IFNGS1 | IBD | GCST90503485 | 39792054 |
| rs148947833 | LINC00598 | IBD | GCST90503485 | 39792054 |
| rs3850378 | GALC | IBD | GCST90503485 | 39792054 |
| rs28374519 | CLN3 | IBD | GCST90503485 | 39792054 |
| rs62034318 | NUPR1,IL27 | IBD | GCST90503485 | 39792054 |
| rs2726036 | SBK1,NPIPB6 | IBD | GCST90503485 | 39792054 |
| rs56062135 | SMAD3 | IBD | GCST90503485 | 39792054 |
| rs2650492 | SBK1 | IBD | GCST90503485 | 39792054 |
| rs231977 | IL27,NUPR1 | IBD | GCST90503485 | 39792054 |
| rs750155 | SULT1A1 | IBD | GCST90503485 | 39792054 |
| rs11646242 | CYLDS1 | IBD | GCST90503485 | 39792054 |
| rs78299226 | LINC02128,LINC02127 | IBD | GCST90503485 | 39792054 |
| rs1861762 | CYLDS2,LINC02168 | IBD | GCST90503485 | 39792054 |
| rs3091316 | CCL2,CCL7 | IBD | GCST90503485 | 39792054 |
| rs7186262 | NKD1,SNX20 | IBD | GCST90503485 | 39792054 |
| rs12602782 | CCL11,CCL8 | IBD | GCST90503485 | 39792054 |
| rs3809717 | GRB7,MIEN1 | IBD | GCST90503485 | 39792054 |
| rs4795393 | MIEN1,GRB7 | IBD | GCST90503485 | 39792054 |
| rs2952155 | ERBB2 | IBD | GCST90503485 | 39792054 |
| rs12603481 | LRRC3C | IBD | GCST90503485 | 39792054 |
| rs11078926 | GSDMB | IBD | GCST90503485 | 39792054 |
| rs9908237 | STAT5A | IBD | GCST90503485 | 39792054 |
| rs8072566 | STAT3 | IBD | GCST90503485 | 39792054 |
| rs744166 | STAT3 | IBD | GCST90503485 | 39792054 |
| rs2847266 | LINC01882 | IBD | GCST90503485 | 39792054 |
| rs12966224 | LINC01882 | IBD | GCST90503485 | 39792054 |
| rs3736164 | STAT3 | IBD | GCST90503485 | 39792054 |
| rs11656652 | CAVIN1 | IBD | GCST90503485 | 39792054 |
| rs2186941 | LINC01882 | IBD | GCST90503485 | 39792054 |
| rs2542147 | LINC01882 | IBD | GCST90503485 | 39792054 |
| rs80262450 | PTPN2 | IBD | GCST90503485 | 39792054 |
| rs10412207 | SBNO2 | IBD | GCST90503485 | 39792054 |
| rs547268 | PTPN2 | IBD | GCST90503485 | 39792054 |
| rs35580406 | PTPN2 | IBD | GCST90503485 | 39792054 |
| rs11669443 | SBNO2 | IBD | GCST90503485 | 39792054 |
| rs11582349 | RNU681P,FCGR2A | IBD | GCST90503485 | 39792054 |
| rs10800314 | FCGR2A,RNU681P | IBD | GCST90503485 | 39792054 |
| rs4657041 | FCGR2A | IBD | GCST90503485 | 39792054 |
| rs4656309 | FCGR2A | IBD | GCST90503485 | 39792054 |
| rs11590749 | FCGR2A | IBD | GCST90503485 | 39792054 |
| rs10919347 | RPS23P10,HSPA6 | IBD | GCST90503485 | 39792054 |
| rs296526 | INAVA | IBD | GCST90503485 | 39792054 |
| rs1572789 | MROH3P | IBD | GCST90503485 | 39792054 |
| rs72749142 | KIF21B | IBD | GCST90503485 | 39792054 |
| rs41299637 | INAVA | IBD | GCST90503485 | 39792054 |
| rs296539 | MROH3P | IBD | GCST90503485 | 39792054 |
| rs55705316 | IL10,Y_RNA | IBD | GCST90503485 | 39792054 |
| rs12123181 | IL19 | IBD | GCST90503485 | 39792054 |
| rs11583398 | IL19 | IBD | GCST90503485 | 39792054 |
| rs4845134 | IL19 | IBD | GCST90503485 | 39792054 |
| rs11583204 | IL20,IL19 | IBD | GCST90503485 | 39792054 |
| rs112694524 | ZFP36L2,LINC01126 | IBD | GCST90503485 | 39792054 |
| rs10186441 | THADA,Y_RNA | IBD | GCST90503485 | 39792054 |
| rs55946629 | PLEKHH2,Y_RNA | IBD | GCST90503485 | 39792054 |
| rs2115592 | REL | IBD | GCST90503485 | 39792054 |
| rs6706689 | PUS10 | IBD | GCST90503485 | 39792054 |
| rs10174032 | PUS10 | IBD | GCST90503485 | 39792054 |
| rs4643526 | PUS10 | IBD | GCST90503485 | 39792054 |
| rs112401990 | PUS10 | IBD | GCST90503485 | 39792054 |
| rs2310173 | IL1R1,IL1R2 | IBD | GCST90503485 | 39792054 |
| rs62358228 | RNU150P,TTC33 | IBD | GCST90503485 | 39792054 |
| rs112848340 | TTC33,RNU150P | IBD | GCST90503485 | 39792054 |
| rs78951062 | RNU150P,TTC33 | IBD | GCST90503485 | 39792054 |
| rs4957129 | TTC33,RNU150P | IBD | GCST90503485 | 39792054 |
| rs10055349 | RNU150P,TTC33 | IBD | GCST90503485 | 39792054 |
| rs12515934 | RNU150P,TTC33 | IBD | GCST90503485 | 39792054 |
| rs56249542 | TTC33,RNU150P | IBD | GCST90503485 | 39792054 |
| rs76523431 | TTC33,RNU150P | IBD | GCST90503485 | 39792054 |
| rs114152040 | TTC33,RNU150P | IBD | GCST90503485 | 39792054 |
| rs111293474 | RNU150P,TTC33 | IBD | GCST90503485 | 39792054 |
| rs10077544 | RNU150P,TTC33 | IBD | GCST90503485 | 39792054 |
| rs4452593 | RNU150P,TTC33 | IBD | GCST90503485 | 39792054 |
| rs4587119 | RNU150P,TTC33 | IBD | GCST90503485 | 39792054 |
| rs10041894 | TTC33,RNU150P | IBD | GCST90503485 | 39792054 |
| rs77097031 | TTC33 | IBD | GCST90503485 | 39792054 |
| rs2036597 | TTC33 | IBD | GCST90503485 | 39792054 |
| rs10941518 | TTC33 | IBD | GCST90503485 | 39792054 |
| rs10408351 | SLC7A10,CEBPA | IBD | GCST90503485 | 39792054 |
| rs6090460 | GMEB2,HELZ2 | IBD | GCST90503485 | 39792054 |
| rs310674 | GMEB2,HELZ2 | IBD | GCST90503485 | 39792054 |
| rs6089945 | MHENCR,STMN3 | IBD | GCST90503485 | 39792054 |
| rs6089946 | MHENCR,STMN3 | IBD | GCST90503485 | 39792054 |
| rs2738774 | STMN3,MHENCR | IBD | GCST90503485 | 39792054 |
| rs909334 | MHENCR,STMN3 | IBD | GCST90503485 | 39792054 |
| rs2738776 | STMN3 | IBD | GCST90503485 | 39792054 |
| rs444210 | MIR3939,RNASET2 | IBD | GCST90503485 | 39792054 |
| rs6911490 | ATG5,PRDM1 | IBD | GCST90503485 | 39792054 |
| rs11152949 | PRDM1 | IBD | GCST90503485 | 39792054 |
| rs7752873 | ATG5 | IBD | GCST90503485 | 39792054 |
| rs28701841 | ATG5,PRDM1 | IBD | GCST90503485 | 39792054 |
| rs13199692 | CEP43,CCR6 | IBD | GCST90503485 | 39792054 |
| rs4730272 | PIGCP2,DLD | IBD | GCST90503485 | 39792054 |
| rs740287 | LAMB1 | IBD | GCST90503485 | 39792054 |
| rs6955771 | PIGCP2,DLD | IBD | GCST90503485 | 39792054 |
| rs4871611 | TRIB1AL | IBD | GCST90503485 | 39792054 |
| rs7028112 | JAK2 | IBD | GCST90503485 | 39792054 |
| rs11999802 | INSL6,INSL4 | IBD | GCST90503485 | 39792054 |
| rs1327500 | JAK2,HNRNPA1P41 | IBD | GCST90503485 | 39792054 |
| rs7849191 | JAK2 | IBD | GCST90503485 | 39792054 |
| rs10815147 | JAK2 | IBD | GCST90503485 | 39792054 |
| rs6478108 | TNFSF15 | IBD | GCST90503485 | 39792054 |
| rs4246905 | TNFSF15 | IBD | GCST90503485 | 39792054 |
| rs28578070 | GPSM1 | IBD | GCST90503485 | 39792054 |
| rs149619730 | RPL37 | IBD | GCST90503485 | 39792054 |
| rs3852206 | CSF2,P4HA2S1 | IBD | GCST90503485 | 39792054 |
| rs25890 | CSF2,P4HA2S1 | IBD | GCST90503485 | 39792054 |
| rs156109 | P4HA2 | IBD | GCST90503485 | 39792054 |
| rs4551060 | SLC22A4,MIR3936HG | IBD | GCST90503485 | 39792054 |
| rs4705940 | CARINH,SLC22A5 | IBD | GCST90503485 | 39792054 |
| rs17622208 | SLC22A5 | IBD | GCST90503485 | 39792054 |
| rs10071051 | CARINH | IBD | GCST90503485 | 39792054 |
| rs11242110 | SLC22A5,CARINH | IBD | GCST90503485 | 39792054 |
| rs6897597 | CARINH,SLC22A5 | IBD | GCST90503485 | 39792054 |
| rs11745587 | CARINH,IRF1 | IBD | GCST90503485 | 39792054 |
| rs2522051 | IRF1,CARINH | IBD | GCST90503485 | 39792054 |
| rs2548993 | IRF1,CARINH | IBD | GCST90503485 | 39792054 |
| rs2548998 | IRF1 | IBD | GCST90503485 | 39792054 |
| rs66536655 | SMIM3,IRGM | IBD | GCST90503485 | 39792054 |
| rs1428552 | IRGM | IBD | GCST90503485 | 39792054 |
| rs183636467 | GPX3,ZNF300P1 | IBD | GCST90503485 | 39792054 |
| rs1157509 | IL12B,LINC01845 | IBD | GCST90503485 | 39792054 |
| rs9968642 | LINC01845,IL12B | IBD | GCST90503485 | 39792054 |
| rs11745241 | ZNF300,ZNF300P1 | IBD | GCST90503485 | 39792054 |
| rs138384115 | LINC01845,IL12B | IBD | GCST90503485 | 39792054 |
| rs918521 | IL12B,LINC01845 | IBD | GCST90503485 | 39792054 |
| rs10476295 | IL12B,LINC01845 | IBD | GCST90503485 | 39792054 |
| rs9370774 | RPL6P17,RNU693P | IBD | GCST90503485 | 39792054 |
| rs3865523 | STMN3 | IBD | GCST90503485 | 39792054 |
| rs11696198 | RTEL1,RTEL1NFRSF6B | IBD | GCST90503485 | 39792054 |
| rs6089763 | RTEL1NFRSF6B,RTEL1 | IBD | GCST90503485 | 39792054 |
| rs2150910 | STMN3 | IBD | GCST90503485 | 39792054 |
| rs6062294 | STMN3 | IBD | GCST90503485 | 39792054 |
| rs34538116 | RTEL1,RTEL1NFRSF6B | IBD | GCST90503485 | 39792054 |
| rs6011026 | RTEL1,RTEL1NFRSF6B | IBD | GCST90503485 | 39792054 |
| rs4809330 | ZGPAT | IBD | GCST90503485 | 39792054 |
| rs6089970 | ZBTB46 | IBD | GCST90503485 | 39792054 |
| rs2297441 | RTEL1,RTEL1NFRSF6B | IBD | GCST90503485 | 39792054 |
| rs13052469 | LINC02920,CYCSP42 | IBD | GCST90503485 | 39792054 |
| rs1297271 | LINC02920,CYCSP42 | IBD | GCST90503485 | 39792054 |
| rs7283479 | GATD3 | IBD | GCST90503485 | 39792054 |
| rs57171270 | CYCSP42,LINC02920 | IBD | GCST90503485 | 39792054 |
| rs443669 | LINC02940,RPL23AP12 | IBD | GCST90503485 | 39792054 |
| rs3804032 | GATD3 | IBD | GCST90503485 | 39792054 |
| rs5754100 | UBE2L3 | IBD | GCST90503485 | 39792054 |
| rs2838527 | ICOSLG,GATD3 | IBD | GCST90503485 | 39792054 |
| rs5752968 | ASCC2 | IBD | GCST90503485 | 39792054 |
| rs1003342 | HORMAD2 | IBD | GCST90503485 | 39792054 |
| rs73165237 | RPL3,PDGFB | IBD | GCST90503485 | 39792054 |
| rs7285952 | SYNGR1,RPL3 | IBD | GCST90503485 | 39792054 |
| rs80250173 | TTC33 | IBD | GCST90503485 | 39792054 |
| rs39897 | CSF2,P4HA2S1 | IBD | GCST90503485 | 39792054 |
| rs6873866 | ERAP2 | IBD | GCST90503485 | 39792054 |
| rs4921492 | LINC01845,IL12B | IBD | GCST90503485 | 39792054 |
| rs12653520 | IL12B,LINC01845 | IBD | GCST90503485 | 39792054 |
| rs11738617 | LINC01845 | IBD | GCST90503485 | 39792054 |
| rs1012636 | CDKAL1 | IBD | GCST90503485 | 39792054 |
| rs6568421 | PRDM1,RN7SKP211 | IBD | GCST90503485 | 39792054 |
| rs76504641 | TTC33 | IBD | GCST90503485 | 39792054 |
| rs10870202 | DNLZ | IBD | GCST90503485 | 39792054 |
| rs12237626 | TNFSF15,DELEC1 | IBD | GCST90503485 | 39792054 |
| rs3829109 | DNLZ | IBD | GCST90503485 | 39792054 |
| rs4073153 | CARD9 | IBD | GCST90503485 | 39792054 |
| rs3812555 | CARD9 | IBD | GCST90503485 | 39792054 |
| rs138408105 | ENTR1 | IBD | GCST90503485 | 39792054 |
| rs3124994 | SNAPC4,ENTR1 | IBD | GCST90503485 | 39792054 |
| rs11145917 | ENTR1 | IBD | GCST90503485 | 39792054 |
| rs10870139 | ENTR1 | IBD | GCST90503485 | 39792054 |
| rs35288226 | SEC16A | IBD | GCST90503485 | 39792054 |
| rs11145988 | PMPCA | IBD | GCST90503485 | 39792054 |
| rs1139057 | C9orf163 | IBD | GCST90503485 | 39792054 |
| rs10781543 | INPP5E | IBD | GCST90503485 | 39792054 |
| rs73566945 | INPP5E | IBD | GCST90503485 | 39792054 |
| rs7870145 | NOTCH1 | IBD | GCST90503485 | 39792054 |
| rs11574896 | NOTCH1 | IBD | GCST90503485 | 39792054 |
| rs4880099 | NOTCH1 | IBD | GCST90503485 | 39792054 |
| rs2229975 | NOTCH1 | IBD | GCST90503485 | 39792054 |
| rs10826797 | CCND3P1,NIFKP1 | IBD | GCST90503485 | 39792054 |
| rs17500653 | CCNYS1 | IBD | GCST90503485 | 39792054 |
| rs12764283 | CCNYS1 | IBD | GCST90503485 | 39792054 |
| rs10995258 | LINC02929 | IBD | GCST90503485 | 39792054 |
| rs4746522 | LINC02929,ALDH7A1P4 | IBD | GCST90503485 | 39792054 |
| rs78439216 | CCNY | IBD | GCST90503485 | 39792054 |
| rs12254043 | CCNYS1,CCNY | IBD | GCST90503485 | 39792054 |
| rs224136 | ALDH7A1P4,LINC02929 | IBD | GCST90503485 | 39792054 |
| rs224123 | LINC02929,ALDH7A1P4 | IBD | GCST90503485 | 39792054 |
| rs13334400 | BRD7 | IBD | GCST90503485 | 39792054 |
| rs11644238 | LINC02178,BRD7 | IBD | GCST90503485 | 39792054 |
| rs62036658 | TUFM,ATXN2L | IBD | GCST90503485 | 39792054 |
| rs9934775 | BRD7 | IBD | GCST90503485 | 39792054 |
| rs62029974 | BRD7 | IBD | GCST90503485 | 39792054 |
| rs4785220 | NKD1 | IBD | GCST90503485 | 39792054 |
| rs9480634 | PRDM1 | IBD | GCST90292538 | 37156999 |
| rs1991866 | CCDC26 | IBD | GCST90292538 | 37156999 |
| rs210648 | DCBLD1 | IBD | GCST90292538 | 37156999 |
| rs36051895 | JAK2,HNRNPA1P41 | IBD | GCST90292538 | 37156999 |
| rs4743820 | LINC00484,LINC02937 | IBD | GCST90292538 | 37156999 |
| rs10992396 | IPPK,RPL21P86 | IBD | GCST90292538 | 37156999 |
| rs56211063 | DELEC1,TNFSF15 | IBD | GCST90292538 | 37156999 |
| rs6939196 | RNASET2,MIR3939 | IBD | GCST90292538 | 37156999 |
| rs2688607 | C10orf55,CAMK2G | IBD | GCST90292538 | 37156999 |
| rs2260230 | GNA12 | IBD | GCST90292538 | 37156999 |
| rs1870148 | TSPAN14 | IBD | GCST90292538 | 37156999 |
| rs76380568 | ITGB8,EEF1A1P27 | IBD | GCST90292538 | 37156999 |
| rs7918084 | EIF2S2P3 | IBD | GCST90292538 | 37156999 |
| rs1990134 | GLI3,INHBAS1 | IBD | GCST90292538 | 37156999 |
| rs4917129 | IKZF1,SPMIP7 | IBD | GCST90292538 | 37156999 |
| rs117026326 | GTF2I,GTF2IS1 | IBD | GCST90292538 | 37156999 |
| rs144341967 | KPNA7,SMURF1 | IBD | GCST90292538 | 37156999 |
| rs1734907 | POP7,EPO | IBD | GCST90292538 | 37156999 |
| rs181826 | NDFIP1 | IBD | GCST90292538 | 37156999 |
| rs59672579 | ARHGAP26S1,ARHGAP26 | IBD | GCST90292538 | 37156999 |
| rs3757387 | IRF5,KCP | IBD | GCST90292538 | 37156999 |
| rs243500 | CUL1 | IBD | GCST90292538 | 37156999 |
| rs17057051 | PTK2B | IBD | GCST90292538 | 37156999 |
| rs77535993 | UBE2V2,IDI1P2 | IBD | GCST90292538 | 37156999 |
| rs2721933 | TRPS1 | IBD | GCST90292538 | 37156999 |
| rs10051765 | SLC34A1,RGS14 | IBD | GCST90292538 | 37156999 |
| rs77688705 | DUSP22,LINC03066 | IBD | GCST90292538 | 37156999 |
| rs17119 | RNU693P,RPL6P17 | IBD | GCST90292538 | 37156999 |
| rs2328546 | CDKAL1 | IBD | GCST90292538 | 37156999 |
| rs4710973 | LINC00581,CDKAL1 | IBD | GCST90292538 | 37156999 |
| rs9271511 | HLAQA1,HLARB1 | IBD | GCST90292538 | 37156999 |
| rs67289879 | CCND3 | IBD | GCST90292538 | 37156999 |
| rs1044690 | TMEM151B | IBD | GCST90292538 | 37156999 |
| rs943689 | BACH2 | IBD | GCST90292538 | 37156999 |
| rs3769684 | CD28 | IBD | GCST90292538 | 37156999 |
| rs11690316 | CXCR2,CXCR1 | IBD | GCST90292538 | 37156999 |
| rs4973341 | CCL20,SNRPGP8 | IBD | GCST90292538 | 37156999 |
| rs13385151 | SP140 | IBD | GCST90292538 | 37156999 |
| rs12692254 | ATG16L1 | IBD | GCST90292538 | 37156999 |
| rs34236350 | GPR35 | IBD | GCST90292538 | 37156999 |
| rs115622046 | BOKS1 | IBD | GCST90292538 | 37156999 |
| rs73180609 | SATB1S1 | IBD | GCST90292538 | 37156999 |
| rs111456533 | EEF1AKMT2 | IBD | GCST90292538 | 37156999 |
| rs16940202 | LINC01082,LINC02132 | IBD | GCST90292538 | 37156999 |
| rs16946807 | CRK | IBD | GCST90292538 | 37156999 |
| rs10896794 | LPXN | IBD | GCST90292538 | 37156999 |
| rs11230563 | CD6 | IBD | GCST90292538 | 37156999 |
| rs174564 | FADS1,FADS2 | IBD | GCST90292538 | 37156999 |
| rs1783521 | PRDX5,CCDC88B | IBD | GCST90292538 | 37156999 |
| rs11658993 | IKZF3 | IBD | GCST90292538 | 37156999 |
| rs9911533 | CCR7,SMARCE1 | IBD | GCST90292538 | 37156999 |
| rs12601611 | STAT3 | IBD | GCST90292538 | 37156999 |
| rs1470831 | SKAP1 | IBD | GCST90292538 | 37156999 |
| rs1292053 | TUBD1 | IBD | GCST90292538 | 37156999 |
| rs8072795 | SLC39A11,LINC00511 | IBD | GCST90292538 | 37156999 |
| rs116885423 | PVALEF,CEP131 | IBD | GCST90292538 | 37156999 |
| rs199762200 | RPS26,ERBB3 | IBD | GCST90292538 | 37156999 |
| rs547268 | PTPN2 | IBD | GCST90292538 | 37156999 |
| rs61576454 | LYZ | IBD | GCST90292538 | 37156999 |
| rs138863186 | CTIF,SMAD7 | IBD | GCST90292538 | 37156999 |
| rs9635956 | ELAC1 | IBD | GCST90292538 | 37156999 |
| rs11669443 | SBNO2 | IBD | GCST90292538 | 37156999 |
| rs11064881 | CIT | IBD | GCST90292538 | 37156999 |
| rs138636798 | SLC7A10,CEBPA | IBD | GCST90292538 | 37156999 |
| rs1644682 | ZNF568 | IBD | GCST90292538 | 37156999 |
| rs9525562 | RPS28P8,DGKH | IBD | GCST90292538 | 37156999 |
| rs55665939 | PPP5C,HIF3A | IBD | GCST90292538 | 37156999 |
| rs646327 | FUT2,MAMSTR | IBD | GCST90292538 | 37156999 |
| rs11672983 | RNU622P,FCAR | IBD | GCST90292538 | 37156999 |
| rs938649 | LINC00824 | IBD | GCST90292538 | 37156999 |
| rs12722489 | IL2RA | IBD | GCST90292538 | 37156999 |
| rs6870798 | TMEM174,LINC02230 | IBD | GCST90292538 | 37156999 |
| rs10041920 | SERINC5 | IBD | GCST90292538 | 37156999 |
| rs6873866 | ERAP2 | IBD | GCST90292538 | 37156999 |
| rs769009 | FRMPD2 | IBD | GCST90292538 | 37156999 |
| rs2188962 | CARINH | IBD | GCST90292538 | 37156999 |
| rs10761659 | ALDH7A1P4,LINC02929 | IBD | GCST90292538 | 37156999 |
| rs74572369 | ADAP1,COX19 | IBD | GCST90292538 | 37156999 |
| rs1250566 | ZMIZ1 | IBD | GCST90292538 | 37156999 |
| rs748670681 | TNRC18 | IBD | GCST90292538 | 37156999 |
| rs11203002 | CH25H,MIR4679 | IBD | GCST90292538 | 37156999 |
| rs73120731 | ELMO1 | IBD | GCST90292538 | 37156999 |
| rs4409764 | LINC01475 | IBD | GCST90292538 | 37156999 |
| rs113369211 | BTRC | IBD | GCST90292538 | 37156999 |
| rs11591780 | MFSD13A,ACTR1A | IBD | GCST90292538 | 37156999 |
| rs10884966 | DUSP5T | IBD | GCST90292538 | 37156999 |
| rs4380874 | PIGCP2,DLD | IBD | GCST90292538 | 37156999 |
| rs1000113 | IRGM | IBD | GCST90292538 | 37156999 |
| rs1363232 | TIMD4 | IBD | GCST90292538 | 37156999 |
| rs755374 | IL12B,LINC01845 | IBD | GCST90292538 | 37156999 |
| rs2101594 | STK10 | IBD | GCST90292538 | 37156999 |
| rs564349 | ERGIC1 | IBD | GCST90292538 | 37156999 |
| rs359457 | CPEB4,LINC01485 | IBD | GCST90292538 | 37156999 |
| rs4380956 | TRIB1AL | IBD | GCST90292538 | 37156999 |
| rs11548656 | PLCG2 | IBD | GCST90292538 | 37156999 |
| rs907613 | LSP1 | IBD | GCST90292538 | 37156999 |
| rs9645621 | NUCB2 | IBD | GCST90292538 | 37156999 |
| rs77531520 | PRR5L | IBD | GCST90292538 | 37156999 |
| rs181068528 | TMEM230P2,OR2AH1P | IBD | GCST90292538 | 37156999 |
| rs415704 | CLEC10A,SLC16A11 | IBD | GCST90292538 | 37156999 |
| rs4795894 | CCL7,CCL2 | IBD | GCST90292538 | 37156999 |
| rs71040008 | EMSY,LINC02757 | IBD | GCST90292538 | 37156999 |
| rs561722 | NXPE2P1,NXPE1 | IBD | GCST90292538 | 37156999 |
| rs11221332 | ETS1 | IBD | GCST90292538 | 37156999 |
| rs4020660 | LTBR,RPL31P10 | IBD | GCST90292538 | 37156999 |
| rs181198871 | ZNF970P,AK6P2 | IBD | GCST90292538 | 37156999 |
| rs75913487 | MUC19 | IBD | GCST90292538 | 37156999 |
| rs11168249 | HDAC7 | IBD | GCST90292538 | 37156999 |
| rs663411 | SMCHD1 | IBD | GCST90292538 | 37156999 |
| rs1558746 | IFNGS1 | IBD | GCST90292538 | 37156999 |
| rs16978179 | SETBP1 | IBD | GCST90292538 | 37156999 |
| rs117121174 | ATP2A2,IFT81 | IBD | GCST90292538 | 37156999 |
| rs11066188 | HECTD4 | IBD | GCST90292538 | 37156999 |
| rs12720356 | TYK2 | IBD | GCST90292538 | 37156999 |
| rs17085007 | RPS21P8,FGFR1OP2P1 | IBD | GCST90292538 | 37156999 |
| rs17630801 | ELF1 | IBD | GCST90292538 | 37156999 |
| rs2604913 | COQ8B | IBD | GCST90292538 | 37156999 |
| rs6770038 | TGFBR2 | IBD | GCST90292538 | 37156999 |
| rs113010081 | CCRL2,LINC02009 | IBD | GCST90292538 | 37156999 |
| rs1131095 | APEH | IBD | GCST90292538 | 37156999 |
| rs138317798 | DUSP7,RPL29 | IBD | GCST90292538 | 37156999 |
| rs2593855 | FOXP1 | IBD | GCST90292538 | 37156999 |
| rs503734 | IMPG2 | IBD | GCST90292538 | 37156999 |
| rs616597 | NFKBIZ | IBD | GCST90292538 | 37156999 |
| rs6763931 | ZBTB38 | IBD | GCST90292538 | 37156999 |
| rs56116661 | LPP | IBD | GCST90292538 | 37156999 |
| rs16844401 | HGFAC | IBD | GCST90292538 | 37156999 |
| rs61797472 | ARAP2 | IBD | GCST90292538 | 37156999 |
| rs73243351 | LINC02513 | IBD | GCST90292538 | 37156999 |
| rs13107325 | SLC39A8 | IBD | GCST90292538 | 37156999 |
| rs2903386 | TET2,TET2S1 | IBD | GCST90292538 | 37156999 |
| rs147909357 | IL2,IL21 | IBD | GCST90292538 | 37156999 |
| rs3776414 | DAP | IBD | GCST90292538 | 37156999 |
| rs395157 | OSMR | IBD | GCST90292538 | 37156999 |
| rs7725052 | TTC33,RNU150P | IBD | GCST90292538 | 37156999 |
| rs73534586 | TRAF3IP2S1,TRAF3IP2 | IBD | GCST90292538 | 37156999 |
| rs682251 | DOCK8 | IBD | GCST90292538 | 37156999 |
| rs6933404 | BTF3L4P3,LINC03004 | IBD | GCST90292538 | 37156999 |
| rs12191421 | PHACTR2 | IBD | GCST90292538 | 37156999 |
| rs7758080 | TAB2S1,TAB2 | IBD | GCST90292538 | 37156999 |
| rs212408 | TAGAP,TAGAPS1 | IBD | GCST90292538 | 37156999 |
| rs8176719 | ABO | IBD | GCST90292538 | 37156999 |
| rs3812565 | SNAPC4 | IBD | GCST90292538 | 37156999 |
| rs10065637 | ANKRD55 | IBD | GCST90292538 | 37156999 |
| rs4748198 | PRKCQS1 | IBD | GCST90292538 | 37156999 |
| rs7898978 | CCND3P1,MAP3K8 | IBD | GCST90292538 | 37156999 |
| rs12764283 | CCNYS1 | IBD | GCST90292538 | 37156999 |
| rs341295 | TMEM183AP6,HMGB3P16 | IBD | GCST90292538 | 37156999 |
| rs2790211 | IPMK | IBD | GCST90292538 | 37156999 |
| rs254560 | PITX1S1 | IBD | GCST90292538 | 37156999 |
| rs8179252 | SPATA31H1,GCKR | IBD | GCST90301319 | 37262302 |
| rs35716097 | SLC34A1 | IBD | GCST90301318 | 37262302 |
| rs887464 | POU5F1,PSORS1C3 | IBD | GCST90301318 | 37262302 |
| rs2844498 | MICB | IBD | GCST90301318 | 37262302 |
| rs3130623 | PRRC2A | IBD | GCST90301318 | 37262302 |
| rs36110630 | LINC02929 | IBD | GCST90301318 | 37262302 |
| rs2915866 | SPRY4,NDFIP1 | IBD | GCST90301319 | 37262302 |
| rs35716097 | SLC34A1 | IBD | GCST90301319 | 37262302 |
| rs2256266 | MOG | IBD | GCST90301319 | 37262302 |
| rs887464 | POU5F1,PSORS1C3 | IBD | GCST90301319 | 37262302 |
| rs2523554 | HLA | IBD | GCST90301319 | 37262302 |
| rs2844498 | MICB | IBD | GCST90301319 | 37262302 |
| rs1150755 | TNXB | IBD | GCST90301318 | 37262302 |
| rs116630553 | RAD50 | IBD | GCST90301319 | 37262302 |
| rs61839660 | IL2RA | IBD | GCST90301318 | 37262302 |
| rs1265061 | C6orf15,RNU6133P | IBD | GCST90301319 | 37262302 |
| rs3128987 | HCP5 | IBD | GCST90301319 | 37262302 |
| rs10489972 | LINC01127,MAP4K4 | IBD | GCST90301318 | 37262302 |
| rs10932775 | CATIPS2,PNKD | IBD | GCST90301318 | 37262302 |
| rs3806156 | TSBP1S1,BTNL2 | IBD | GCST90301319 | 37262302 |
| rs36110630 | LINC02929 | IBD | GCST90301319 | 37262302 |
| rs12931474 | LITAF,RMI2 | IBD | GCST90301319 | 37262302 |
| rs8084006 | PTPN2 | IBD | GCST90301319 | 37262302 |
| rs2690110 | CMAHP,CARMIL1 | IBD | GCST90301317 | 37262302 |
| rs35518316 | LINC02934 | IBD | GCST90301319 | 37262302 |
| rs10489972 | LINC01127,MAP4K4 | IBD | GCST90301319 | 37262302 |
| rs10932775 | CATIPS2,PNKD | IBD | GCST90301319 | 37262302 |
| rs34804116 | LINC02230,TMEM174 | IBD | GCST90301319 | 37262302 |
| rs1893592 | UBASH3A | IBD | GCST90301317 | 37262302 |
| rs2915866 | SPRY4,NDFIP1 | IBD | GCST90301317 | 37262302 |
| rs2072633 | CFB | IBD | GCST90301317 | 37262302 |
| rs3096691 | TSBP1S1,NOTCH4 | IBD | GCST90301317 | 37262302 |
| rs3806156 | TSBP1S1,BTNL2 | IBD | GCST90301317 | 37262302 |
| rs12931474 | LITAF,RMI2 | IBD | GCST90301317 | 37262302 |
| rs7171171 | LINC02694 | IBD | GCST90301318 | 37262302 |
| rs8084006 | PTPN2 | IBD | GCST90301318 | 37262302 |
| rs1990760 | IFIH1 | IBD | GCST90301318 | 37262302 |
| rs116630553 | RAD50 | IBD | GCST90301318 | 37262302 |
| rs11754821 | C6orf15,RNU6133P | IBD | GCST90301318 | 37262302 |
| rs2690110 | CMAHP,CARMIL1 | IBD | GCST90301319 | 37262302 |
| rs3128987 | HCP5 | IBD | GCST90301318 | 37262302 |
| rs1990760 | IFIH1 | IBD | GCST90301319 | 37262302 |
| rs2395158 | BTNL2,TSBP1S1 | IBD | GCST90301318 | 37262302 |
| rs4917014 | SPMIP7,IKZF1 | IBD | GCST90301318 | 37262302 |
| rs11754821 | C6orf15,RNU6133P | IBD | GCST90301319 | 37262302 |
| rs76458677 | CCNYS1,CCNY | IBD | GCST90301318 | 37262302 |
| rs12268645 | SLC25A28,NKX2 | IBD | GCST90301318 | 37262302 |
| rs8179252 | SPATA31H1,GCKR | IBD | GCST90301318 | 37262302 |
| rs35518316 | LINC02934 | IBD | GCST90301318 | 37262302 |
| rs2395158 | BTNL2,TSBP1S1 | IBD | GCST90301319 | 37262302 |
| rs4917014 | SPMIP7,IKZF1 | IBD | GCST90301319 | 37262302 |
| rs61839660 | IL2RA | IBD | GCST90301319 | 37262302 |
| rs76458677 | CCNYS1,CCNY | IBD | GCST90301319 | 37262302 |
| rs12268645 | SLC25A28,NKX2 | IBD | GCST90301319 | 37262302 |
| rs2256266 | MOG | IBD | GCST90301318 | 37262302 |
| rs2523554 | HLA | IBD | GCST90301318 | 37262302 |
| rs6592651 | LINC02757,EMSY | IBD | GCST90301318 | 37262302 |
| rs11788118 | NAMA | IBD | GCST90301318 | 37262302 |
| rs7752195 | CARMIL1,CMAHP | IBD | GCST90301318 | 37262302 |
| rs35812145 | C2orf74S1,C2orf74 | IBD | GCST90301318 | 37262302 |
| rs11788118 | NAMA | IBD | GCST90301319 | 37262302 |
| rs7752195 | CARMIL1,CMAHP | IBD | GCST90301319 | 37262302 |
| rs1265061 | C6orf15,RNU6133P | IBD | GCST90301318 | 37262302 |
| rs1893592 | UBASH3A | IBD | GCST90301319 | 37262302 |
| rs3130623 | PRRC2A | IBD | GCST90301319 | 37262302 |
| rs2072633 | CFB | IBD | GCST90301319 | 37262302 |
| rs3096691 | TSBP1S1,NOTCH4 | IBD | GCST90301319 | 37262302 |
| rs6592651 | LINC02757,EMSY | IBD | GCST90301319 | 37262302 |
| rs7171171 | LINC02694 | IBD | GCST90301319 | 37262302 |
| rs34804116 | LINC02230,TMEM174 | IBD | GCST90301318 | 37262302 |

**S5 Table. Association of genetically proxied lipid-lowering drug targets with risk of IBD, CD and UC.**

| **Exposure** | **Outcome** | **Method** | **OR (95CI%)** | ***P*-value** |
| --- | --- | --- | --- | --- |
| ANGPTL3 | CD | IVW | 3.709(2.293,5.998) | **9.17E-08** |
|  |  | Weighted median | 4.069(2.220,7.458) | **5.61E-06** |
|  |  | MR-Lasso | 3.709(2.293,5.998) | **9.17E-08** |
|  |  | MR-Robust | 3.712(2.647,5.205) | **2.88E-14** |
|  |  | MR-Egger | 2.434(0.594,9.967) | 0.216 |
|  |  | MRRAPS | 3.746(2.254,6.225) | **3.49E-07** |
|  |  | MRPRESSO | 3.709(2.367,5.810) | **0.001** |
|  | IBD | IVW | 1.846(1.298,2.627) | **0.001** |
|  |  | Weighted median | 1.746(1.128,2.701) | **0.012** |
|  |  | MR-Lasso | 1.846(1.298,2.627) | **0.001** |
|  |  | MR-Robust | 1.831(1.462,2.293) | **1.37E-07** |
|  |  | MR-Egger | 1.981(0.706,5.558) | 0.194 |
|  |  | MRRAPS | 1.852(1.282,2.677) | **0.001** |
|  |  | MRPRESSO | 1.846(1.361,2.505) | **0.008** |
|  | UC | IVW | 1.015(0.610,1.688) | 0.954 |
|  |  | Weighted median | 0.951(0.549,1.649) | 0.859 |
|  |  | MR-Lasso | 1.015(0.610,1.688) | 0.954 |
|  |  | MR-Robust | 0.986(0.683,1.423) | 0.940 |
|  |  | MR-Egger | 1.448(0.294,7.132) | 0.649 |
|  |  | MRRAPS | 1.108(0.446,2.754) | 0.825 |
|  |  | MRPRESSO | 1.015(0.610,1.688) | 0.956 |
| APOC3 | CD | IVW | 0.906(0.761,1.078) | 0.265 |
|  |  | Weighted median | 0.968(0.768,1.220) | 0.781 |
|  |  | MR-Lasso | 0.906(0.761,1.078) | 0.265 |
|  |  | MR-Robust | 0.920(0.758,1.117) | 0.398 |
|  |  | MR-Egger | 1.036(0.748,1.434) | 0.833 |
|  |  | MRRAPS | 0.908(0.759,1.086) | 0.292 |
|  |  | MRPRESSO | 0.906(0.790,1.039) | 0.174 |
|  | IBD | IVW | 1.148(1.009,1.306) | **0.036** |
|  |  | Weighted median | 1.133(0.951,1.349) | 0.162 |
|  |  | MR-Lasso | 1.239(1.053,1.458) | **0.010** |
|  |  | MR-Robust | 1.146(1.024,1.283) | **0.018** |
|  |  | MR-Egger | 1.163(0.914,1.480) | 0.220 |
|  |  | MRRAPS | 1.151(1.005,1.319) | **0.043** |
|  |  | MRPRESSO | 1.148(1.013,1.301) | **0.043** |
|  | UC | IVW | 1.239(1.053,1.458) | **0.010** |
|  |  | Weighted median | 1.227(0.984,1.529) | 0.069 |
|  |  | MR-Lasso | 1.197(0.940,1.524) | 0.146 |
|  |  | MR-Robust | 1.208(1.041,1.401) | **0.013** |
|  |  | MR-Egger | 1.199(0.883,1.628) | 0.244 |
|  |  | MRRAPS | 1.219(1.031,1.443) | **0.021** |
|  |  | MRPRESSO | 1.239(1.069,1.436) | **0.010** |
| APOB | CD | IVW | 0.865(0.711,1.053) | 0.148 |
|  |  | Weighted median | 0.857(0.661,1.111) | 0.245 |
|  |  | MR-Lasso | 0.865(0.711,1.053) | 0.148 |
|  |  | MR-Robust | 0.868(0.737,1.022) | 0.089 |
|  |  | MR-Egger | 1.046(0.635,1.723) | 0.860 |
|  |  | MRRAPS | 0.871(0.713,1.064) | 0.176 |
|  |  | MRPRESSO | 0.865(0.711,1.053) | 0.165 |
|  | IBD | IVW | 0.947(0.823,1.089) | 0.442 |
|  |  | Weighted median | 0.859(0.709,1.040) | 0.118 |
|  |  | MR-Lasso | 0.879(0.759,1.017) | 0.082 |
|  |  | MR-Robust | 0.924(0.784,1.089) | 0.347 |
|  |  | MR-Egger | 0.967(0.674,1.388) | 0.856 |
|  |  | MRRAPS | 0.925(0.801,1.068) | 0.290 |
|  |  | MRPRESSO | 0.947(0.823,1.089) | 0.451 |
|  | UC | IVW | 1.046(0.878,1.246) | 0.615 |
|  |  | Weighted median | 1.067(0.836,1.362) | 0.600 |
|  |  | MR-Lasso | 1.046(0.878,1.246) | 0.615 |
|  |  | MR-Robust | 1.033(0.890,1.200) | 0.666 |
|  |  | MR-Egger | 1.079(0.686,1.699) | 0.741 |
|  |  | MRRAPS | 1.022(0.855,1.222) | 0.811 |
|  |  | MRPRESSO | 1.046(0.878,1.246) | 0.621 |
| ABCG5/ABCG8b | CD | IVW | 0.996(0.741,1.337) | 0.976 |
|  |  | Weighted median | 1.096(0.739,1.625) | 0.648 |
|  |  | MR-Lasso | 0.996(0.741,1.337) | 0.976 |
|  |  | MR-Robust | 1.003(0.712,1.411) | 0.988 |
|  |  | MR-Egger | 0.679(0.333,1.387) | 0.288 |
|  |  | MRRAPS | 0.932(0.665,1.305) | 0.680 |
|  |  | MRPRESSO | 0.996(0.741,1.337) | 0.977 |
|  | IBD | IVW | 0.904(0.744,1.097) | 0.307 |
|  |  | Weighted median | 0.797(0.615,1.033) | 0.086 |
|  |  | MR-Lasso | 0.904(0.744,1.097) | 0.307 |
|  |  | MR-Robust | 0.792(0.188,3.333) | 0.750 |
|  |  | MR-Egger | 0.695(0.432,1.117) | 0.133 |
|  |  | MRRAPS | 0.892(0.730,1.091) | 0.267 |
|  |  | MRPRESSO | 0.904(0.766,1.066) | 0.250 |
|  | UC | IVW | 0.850(0.666,1.084) | 0.191 |
|  |  | Weighted median | 0.889(0.653,1.210) | 0.454 |
|  |  | MR-Lasso | 0.850(0.666,1.084) | 0.191 |
|  |  | MR-Robust | 0.897(0.684,1.178) | 0.434 |
|  |  | MR-Egger | 0.791(0.437,1.434) | 0.440 |
|  |  | MRRAPS | 0.853(0.663,1.098) | 0.218 |
|  |  | MRPRESSO | 0.850(0.729,0.991) | 0.059 |
| HMGCR | CD | IVW | 1.007(0.596,1.702) | 0.979 |
|  |  | Weighted median | 0.914(0.523,1.595) | 0.750 |
|  |  | MR-Lasso | 1.007(0.596,1.702) | 0.979 |
|  |  | MR-Robust | 0.996(0.714,1.389) | 0.980 |
|  |  | MR-Egger | 0.221(0.016,3.073) | 0.261 |
|  |  | MRRAPS | 1.007(0.585,1.733) | 0.980 |
|  | IBD | IVW | 0.974(0.660,1.435) | 0.892 |
|  |  | Weighted median | 1.005(0.669,1.510) | 0.982 |
|  |  | MR-Lasso | 0.974(0.660,1.435) | 0.892 |
|  |  | MR-Robust | 0.976(0.798,1.195) | 0.815 |
|  |  | MR-Egger | 1.881(0.273,12.951) | 0.521 |
|  |  | MRRAPS | 0.973(0.651,1.455) | 0.896 |
|  | UC | IVW | 0.934(0.571,1.527) | 0.785 |
|  |  | Weighted median | 1.097(0.649,1.856) | 0.729 |
|  |  | MR-Lasso | 0.934(0.476,1.833) | 0.843 |
|  |  | MR-Robust | 0.953(0.483,1.879) | 0.890 |
|  |  | MR-Egger | 8.668(0.765,98.202) | 0.081 |
|  |  | MRRAPS | 0.901(0.444,1.829) | 0.773 |
| LPL | CD | IVW | 0.661(0.531,0.824) | **2.27E-04** |
|  |  | Weighted median | 0.744(0.551,1.005) | 0.054 |
|  |  | MR-Lasso | 0.661(0.531,0.824) | **2.27E-04** |
|  |  | MR-Robust | 0.667(0.564,0.789) | **2.21E-06** |
|  |  | MR-Egger | 0.769(0.518,1.141) | 0.192 |
|  |  | MRRAPS | 0.660(0.526,0.829) | **3.60E-04** |
|  |  | MRPRESSO | 0.661(0.560,0.781) | **8.20E-05** |
|  | IBD | IVW | 0.772(0.657,0.908) | **0.002** |
|  |  | Weighted median | 0.791(0.630,0.993) | **0.043** |
|  |  | MR-Lasso | 0.772(0.657,0.908) | **0.002** |
|  |  | MR-Robust | 0.777(0.685,0.882) | **0.000** |
|  |  | MR-Egger | 0.876(0.655,1.173) | 0.375 |
|  |  | MRRAPS | 0.776(0.657,0.918) | **0.003** |
|  |  | MRPRESSO | 0.772(0.677,0.881) | **0.001** |
|  | UC | IVW | 0.836(0.682,1.026) | 0.087 |
|  |  | Weighted median | 0.814(0.605,1.097) | 0.176 |
|  |  | MR-Lasso | 0.836(0.682,1.026) | 0.087 |
|  |  | MR-Robust | 0.827(0.699,0.979) | 0.027 |
|  |  | MR-Egger | 0.887(0.613,1.282) | 0.523 |
|  |  | MRRAPS | 0.827(0.669,1.022) | 0.078 |
|  |  | MRPRESSO | 0.836(0.691,1.012) | 0.081 |
| LDLR | CD | IVW | 0.718(0.556,0.928) | **0.011** |
|  |  | Weighted median | 0.891(0.656,1.211) | 0.462 |
|  |  | MR-Lasso | 0.718(0.556,0.928) | **0.011** |
|  |  | MR-Robust | 0.707(0.461,1.082) | 0.110 |
|  |  | MR-Egger | 1.008(0.686,1.482) | 0.966 |
|  |  | MRRAPS | 0.769(0.567,1.045) | 0.093 |
|  |  | MRPRESSO | 0.718(0.556,0.928) | **0.024** |
|  | IBD | IVW | 0.786(0.654,0.944) | **0.010** |
|  |  | Weighted median | 0.866(0.695,1.080) | 0.201 |
|  |  | MR-Lasso | 0.786(0.654,0.944) | **0.010** |
|  |  | MR-Robust | 0.778(0.640,0.945) | **0.011** |
|  |  | MR-Egger | 0.887(0.648,1.215) | 0.456 |
|  |  | MRRAPS | 0.789(0.651,0.958) | **0.017** |
|  |  | MRPRESSO | 0.786(0.654,0.944) | **0.022** |
|  | UC | IVW | 0.909(0.734,1.124) | 0.378 |
|  |  | Weighted median | 0.922(0.700,1.214) | 0.563 |
|  |  | MR-Lasso | 0.909(0.734,1.124) | 0.378 |
|  |  | MR-Robust | 0.908(0.813,1.014) | 0.087 |
|  |  | MR-Egger | 0.830(0.575,1.197) | 0.318 |
|  |  | MRRAPS | 0.910(0.730,1.134) | 0.401 |
|  |  | MRPRESSO | 0.909(0.755,1.094) | 0.328 |
| NPC1L1 | CD | IVW | 0.676(0.280,1.631) | 0.384 |
|  |  | MR-Robust | 0.676(0.367,1.247) | 0.210 |
|  |  | MRRAPS | 0.675(0.269,1.690) | 0.401 |
|  | IBD | IVW | 0.769(0.401,1.477) | 0.431 |
|  |  | MR-Robust | 0.769(0.560,1.057) | 0.106 |
|  |  | MRRAPS | 0.769(0.389,1.520) | 0.450 |
|  | UC | IVW | 0.898(0.393,2.054) | 0.799 |
|  |  | MR-Robust | 0.898(0.600,1.345) | 0.602 |
|  |  | MRRAPS | 0.898(0.379,2.128) | 0.807 |
| PCSK9 | CD | IVW | 0.832(0.642,1.078) | 0.165 |
|  |  | Weighted median | 0.832(0.587,1.180) | 0.303 |
|  |  | MR-Lasso | 0.832(0.642,1.078) | 0.165 |
|  |  | MR-Robust | 0.840(0.640,1.103) | 0.210 |
|  |  | MR-Egger | 0.991(0.587,1.674) | 0.973 |
|  |  | MRRAPS | 0.839(0.642,1.096) | 0.197 |
|  |  | MRPRESSO | 0.832(0.686,1.009) | 0.087 |
|  | IBD | IVW | 0.890(0.738,1.073) | 0.222 |
|  |  | Weighted median | 0.822(0.635,1.063) | 0.136 |
|  |  | MR-Lasso | 0.890(0.738,1.073) | 0.222 |
|  |  | MR-Robust | 0.883(0.745,1.046) | 0.149 |
|  |  | MR-Egger | 0.776(0.532,1.130) | 0.185 |
|  |  | MRRAPS | 0.879(0.723,1.069) | 0.197 |
|  |  | MRPRESSO | 0.890(0.739,1.072) | 0.242 |
|  | UC | IVW | 0.989(0.783,1.250) | 0.927 |
|  |  | Weighted median | 0.910(0.666,1.243) | 0.553 |
|  |  | MR-Lasso | 0.989(0.783,1.250) | 0.927 |
|  |  | MR-Robust | 0.983(0.830,1.164) | 0.840 |
|  |  | MR-Egger | 0.863(0.543,1.372) | 0.535 |
|  |  | MRRAPS | 0.979(0.769,1.247) | 0.864 |
|  |  | MRPRESSO | 0.989(0.793,1.234) | 0.924 |

Abbreviations: IVW, inverse-variance weighted; MR, Mendelian randomization; MR-RAPS, Robust Adjusted Profile Score; MR-PRESSO, Mendelian Randomization Pleiotropy RESidual Sum and Outlier; IBD, inflammatory bowel disease; CD, Crohn’s disease; UC, ulcerative colitis; ABCG5, ATP Binding Cassette Subfamily G Member 5; LPL, Lipoprotein Lipase; LDLR, LDL Receptor; ANGPTL3, Angiopoietin-like 3; APOC3, Apolipoprotein C-III; HMGCR, HMG-CoA Reductase; NPC1L1, Niemann-Pick C1-like Protein 1; PCSK9, Proprotein Convertase Subtilisin/Kexin Type 9.

**S6 Table. Heterogeneity and pleiotropy tests of instrument effects.**

| **Exposure** | **Outcome** | **Method** | **Heterogeneity analysis** | | | **Pleiotropy analysis** | | |
| --- | --- | --- | --- | --- | --- | --- | --- | --- |
|  |  |  | **Q** | **Q_df** | **Q_pval** | **Egger intercept** | **SE** | ***P*-value** |
| ANGPTL3 | CD | MR Egger | 4.841 | 5 | 0.436 | -0.021 | 0.034 | 0.533 |
| ANGPTL3 | CD | IVW | 5.229 | 6 | 0.515 |  |  |  |
| ANGPTL3 | IBD | MR Egger | 4.468 | 5 | 0.484 | 0.003 | 0.025 | 0.887 |
| ANGPTL3 | IBD | IVW | 4.488 | 6 | 0.611 |  |  |  |
| ANGPTL3 | UC | MR Egger | 7.556 | 5 | 0.182 | 0.018 | 0.038 | 0.642 |
| ANGPTL3 | UC | IVW | 7.883 | 6 | 0.247 |  |  |  |
| APOC3 | CD | MR Egger | 10.932 | 18 | 0.897 | 0.013 | 0.014 | 0.339 |
| APOC3 | CD | IVW | 11.845 | 19 | 0.892 |  |  |  |
| APOC3 | IBD | MR Egger | 17.912 | 18 | 0.461 | 0.001 | 0.010 | 0.903 |
| APOC3 | IBD | IVW | 17.927 | 19 | 0.527 |  |  |  |
| APOC3 | UC | MR Egger | 15.525 | 18 | 0.626 | -0.003 | 0.013 | 0.804 |
| APOC3 | UC | IVW | 15.586 | 19 | 0.685 |  |  |  |
| APOB | CD | MR Egger | 20.066 | 18 | 0.329 | 0.016 | 0.020 | 0.417 |
| APOB | CD | IVW | 20.8 | 19 | 0.348 |  |  |  |
| APOB | IBD | MR Egger | 19.547 | 18 | 0.359 | 0.002 | 0.014 | 0.899 |
| APOB | IBD | IVW | 19.564 | 19 | 0.421 |  |  |  |
| APOB | UC | MR Egger | 19.392 | 18 | 0.368 | 0.003 | 0.018 | 0.882 |
| APOB | UC | IVW | 19.416 | 19 | 0.430 |  |  |  |
| ABCG5/ABCG8 | CD | MR Egger | 14.482 | 12 | 0.271 | -0.026 | 0.023 | 0.250 |
| ABCG5/ABCG8 | CD | IVW | 16.077 | 13 | 0.245 |  |  |  |
| ABCG5/ABCG8 | IBD | MR Egger | 7.96 | 12 | 0.788 | -0.018 | 0.015 | 0.234 |
| ABCG5/ABCG8 | IBD | IVW | 9.374 | 13 | 0.744 |  |  |  |
| ABCG5/ABCG8 | UC | MR Egger | 5.113 | 12 | 0.954 | -0.005 | 0.019 | 0.796 |
| ABCG5/ABCG8 | UC | IVW | 5.18 | 13 | 0.971 |  |  |  |
| HMGCR | CD | MR Egger | 0.108 | 1 | 0.743 | -0.099 | 0.086 | 0.249 |
| HMGCR | CD | IVW | 1.435 | 2 | 0.488 |  |  |  |
| HMGCR | IBD | MR Egger | 0.002 | 1 | 0.961 | 0.043 | 0.063 | 0.494 |
| HMGCR | IBD | IVW | 0.469 | 2 | 0.791 |  |  |  |
| HMGCR | UC | MR Egger | 0.387 | 1 | 0.534 | 0.146 | 0.079 | 0.066 |
| HMGCR | UC | IVW | 3.762 | 2 | 0.152 |  |  |  |
| LPL | CD | MR Egger | 11.235 | 20 | 0.940 | 0.010 | 0.011 | 0.367 |
| LPL | CD | IVW | 12.051 | 21 | 0.938 |  |  |  |
| LPL | IBD | MR Egger | 12.93 | 20 | 0.880 | 0.009 | 0.008 | 0.305 |
| LPL | IBD | IVW | 13.984 | 21 | 0.870 |  |  |  |
| LPL | UC | MR Egger | 18.172 | 20 | 0.576 | 0.004 | 0.011 | 0.709 |
| LPL | UC | IVW | 18.311 | 21 | 0.629 |  |  |  |
| LDLR | CD | MR Egger | 13.222 | 13 | 0.431 | 0.028 | 0.013 | 0.132 |
| LDLR | CD | IVW | 17.913 | 14 | 0.211 |  |  |  |
| LDLR | IBD | MR Egger | 15.787 | 13 | 0.261 | 0.010 | 0.011 | 0.350 |
| LDLR | IBD | IVW | 16.848 | 14 | 0.264 |  |  |  |
| LDLR | UC | MR Egger | 10.225 | 13 | 0.675 | -0.007 | 0.012 | 0.551 |
| LDLR | UC | IVW | 10.581 | 14 | 0.719 |  |  |  |
| PCSK9 | CD | MR Egger | 6.103 | 11 | 0.866 | 0.014 | 0.019 | 0.453 |
| PCSK9 | CD | IVW | 6.667 | 12 | 0.879 |  |  |  |
| PCSK9 | IBD | MR Egger | 11.148 | 11 | 0.431 | -0.011 | 0.014 | 0.408 |
| PCSK9 | IBD | IVW | 11.841 | 12 | 0.459 |  |  |  |
| PCSK9 | UC | MR Egger | 10.255 | 11 | 0.508 | -0.011 | 0.017 | 0.506 |
| PCSK9 | UC | IVW | 10.698 | 12 | 0.555 |  |  |  |

Abbreviations: IVW, inverse-variance weighted; MR, Mendelian randomization; MR-RAPS, Robust Adjusted Profile Score; MR-PRESSO, Mendelian Randomization Pleiotropy RESidual Sum and Outlier; IBD, inflammatory bowel disease; CD, Crohn’s disease; UC, ulcerative colitis; ABCG5, ATP Binding Cassette Subfamily G Member 5; LPL, Lipoprotein Lipase; LDLR, LDL Receptor; ANGPTL3, Angiopoietin-like 3; APOC3, Apolipoprotein C-III; HMGCR, HMG-CoA Reductase; NPC1L1, Niemann-Pick C1-like Protein 1; PCSK9, Proprotein Convertase Subtilisin/Kexin Type 9.

**S7 Table. Association Between Genetically Predicted Lipid-Lowering Drug Targets and the Risk of IBD, CD, and UC After Excluding Pleiotropic SNPs.**

| **Exposure** | **Outcome** | **Method** | **OR (95CI%)** | ***P*-value** |
| --- | --- | --- | --- | --- |
| LDLR | CD | IVW | 0.722(0.545,0.956) | 0.023 |
|  |  | Weighted median | 0.893(0.659,1.211) | 0.466 |
|  |  | MR-Lasso | 0.722(0.545,0.956) | 0.023 |
|  |  | MR-Robust | 0.717(0.498,1.032) | 0.073 |
|  |  | MR-Egger | 1.084(0.71,1.656) | 0.708 |
|  |  | MRRAPS | 0.714(0.563,0.906) | 0.006 |
|  |  | MRPRESSO | 0.722(0.545,0.956) | 0.042 |
|  | IBD | IVW | 0.778(0.638,0.949) | 0.013 |
|  |  | Weighted median | 0.792(0.635,0.987) | 0.038 |
|  |  | MR-Lasso | 0.778(0.638,0.949) | 0.013 |
|  |  | MR-Robust | 0.764(0.577,1.012) | 0.061 |
|  |  | MR-Egger | 0.955(0.683,1.336) | 0.788 |
|  |  | MRRAPS | 0.768(0.644,0.916) | 0.003 |
|  |  | MRPRESSO | 0.778(0.638,0.949) | 0.029 |
|  | UC | IVW | 0.891(0.717,1.107) | 0.296 |
|  |  | Weighted median | 0.913(0.692,1.203) | 0.517 |
|  |  | MR-Lasso | 0.891(0.717,1.107) | 0.296 |
|  |  | MR-Robust | 0.891(0.796,0.997) | 0.045 |
|  |  | MR-Egger | 0.886(0.601,1.306) | 0.541 |
|  |  | MRRAPS | 0.891(0.712,1.114) | 0.311 |
|  |  | MRPRESSO | 0.891(0.739,1.074) | 0.248 |
| LPL | CD | IVW | 0.656(0.519,0.828) | 3.98E-04 |
|  |  | Weighted median | 0.744(0.545,1.016) | 0.063 |
|  |  | MR-Lasso | 0.656(0.519,0.828) | 3.98E-04 |
|  |  | MR-Robust | 0.662(0.554,0.791) | 5.92E-06 |
|  |  | MR-Egger | 0.861(0.564,1.314) | 0.489 |
|  |  | MRRAPS | 0.655(0.514,0.834) | 0.001 |
|  |  | MRPRESSO | 0.656(0.546,0.787) | 2.96E-04 |
|  | IBD | IVW | 0.774(0.652,0.919) | 0.004 |
|  |  | Weighted median | 0.790(0.625,1.000) | 0.050 |
|  |  | MR-Lasso | 0.774(0.652,0.919) | 0.004 |
|  |  | MR-Robust | 0.779(0.690,0.880) | 5.62E-05 |
|  |  | MR-Egger | 0.932(0.682,1.273) | 0.658 |
|  |  | MRRAPS | 0.779(0.652,0.931) | 0.006 |
|  |  | MRPRESSO | 0.774(0.665,0.902) | 0.004 |
|  | UC | IVW | 0.848(0.683,1.055) | 0.139 |
|  |  | Weighted median | 0.808(0.595,1.096) | 0.171 |
|  |  | MR-Lasso | 0.848(0.683,1.055) | 0.139 |
|  |  | MR-Robust | 0.833(0.681,1.017) | 0.073 |
|  |  | MR-Egger | 0.911(0.614,1.354) | 0.646 |
|  |  | MRRAPS | 0.838(0.669,1.049) | 0.124 |
|  |  | MRPRESSO | 0.848(0.693,1.039) | 0.131 |

**S8 Table. Effect estimates of the associations between 196 bacterial traits and risk of inflammatory bowel disease in MR analyses.**

| **Exposure** | **Outcome** | **Method** | **OR (95CI%)** | ***P*-value** |
| --- | --- | --- | --- | --- |
| class.Actinobacteria.id.419 | IBD | IVW | 0.882(0.748,1.041) | 0.138 |
| class.Actinobacteria.id.419 | IBD | Weighted median | 0.925(0.755,1.133) | 0.449 |
| class.Actinobacteria.id.419 | IBD | Weighted mode | 1.016(0.758,1.361) | 0.918 |
| class.Actinobacteria.id.419 | IBD | MR-Robust | 0.878(0.750,1.028) | 0.105 |
| class.Actinobacteria.id.419 | IBD | MR-Egger | 0.810(0.466,1.406) | 0.453 |
| class.Actinobacteria.id.419 | IBD | MRRAPS | 0.857(0.720,1.020) | 0.083 |
| class.Actinobacteria.id.419 | IBD | MRPRESSO | 0.882(0.748,1.041) | 0.153 |
| class.Alphaproteobacteria.id.2379 | IBD | IVW | 1.039(0.857,1.260) | 0.694 |
| class.Alphaproteobacteria.id.2379 | IBD | Weighted median | 1.111(0.872,1.415) | 0.393 |
| class.Alphaproteobacteria.id.2379 | IBD | Weighted mode | 1.156(0.819,1.630) | 0.410 |
| class.Alphaproteobacteria.id.2379 | IBD | MR-Robust | 1.043(0.895,1.215) | 0.588 |
| class.Alphaproteobacteria.id.2379 | IBD | MR-Egger | 1.444(0.695,2.999) | 0.325 |
| class.Alphaproteobacteria.id.2379 | IBD | MRRAPS | 1.040(0.843,1.284) | 0.714 |
| class.Alphaproteobacteria.id.2379 | IBD | MRPRESSO | 1.039(0.908,1.189) | 0.594 |
| class.Bacilli.id.1673 | IBD | IVW | 0.985(0.826,1.174) | 0.867 |
| class.Bacilli.id.1673 | IBD | Weighted median | 0.980(0.784,1.223) | 0.855 |
| class.Bacilli.id.1673 | IBD | Weighted mode | 0.973(0.703,1.346) | 0.868 |
| class.Bacilli.id.1673 | IBD | MR-Robust | 0.996(0.844,1.175) | 0.961 |
| class.Bacilli.id.1673 | IBD | MR-Egger | 1.018(0.621,1.670) | 0.942 |
| class.Bacilli.id.1673 | IBD | MRRAPS | 0.996(0.841,1.179) | 0.960 |
| class.Bacilli.id.1673 | IBD | MRPRESSO | 0.985(0.826,1.174) | 0.869 |
| class.Bacteroidia.id.912 | IBD | IVW | 0.976(0.810,1.175) | 0.796 |
| class.Bacteroidia.id.912 | IBD | Weighted median | 0.980(0.765,1.254) | 0.872 |
| class.Bacteroidia.id.912 | IBD | Weighted mode | 1.047(0.733,1.495) | 0.800 |
| class.Bacteroidia.id.912 | IBD | MR-Robust | 0.956(0.745,1.228) | 0.725 |
| class.Bacteroidia.id.912 | IBD | MR-Egger | 1.352(0.925,1.977) | 0.119 |
| class.Bacteroidia.id.912 | IBD | MRRAPS | 0.977(0.793,1.204) | 0.828 |
| class.Bacteroidia.id.912 | IBD | MRPRESSO | 0.976(0.810,1.175) | 0.800 |
| class.Betaproteobacteria.id.2867 | IBD | IVW | 0.922(0.762,1.114) | 0.398 |
| class.Betaproteobacteria.id.2867 | IBD | Weighted median | 0.989(0.768,1.272) | 0.929 |
| class.Betaproteobacteria.id.2867 | IBD | Weighted mode | 0.992(0.665,1.478) | 0.967 |
| class.Betaproteobacteria.id.2867 | IBD | MR-Robust | 0.948(0.748,1.201) | 0.657 |
| class.Betaproteobacteria.id.2867 | IBD | MR-Egger | 0.710(0.358,1.410) | 0.328 |
| class.Betaproteobacteria.id.2867 | IBD | MRRAPS | 0.940(0.766,1.153) | 0.551 |
| class.Betaproteobacteria.id.2867 | IBD | MRPRESSO | 0.922(0.770,1.103) | 0.392 |
| class.Clostridia.id.1859 | IBD | IVW | 1.135(0.928,1.388) | 0.217 |
| class.Clostridia.id.1859 | IBD | Weighted median | 1.117(0.855,1.459) | 0.418 |
| class.Clostridia.id.1859 | IBD | Weighted mode | 1.014(0.638,1.612) | 0.953 |
| class.Clostridia.id.1859 | IBD | MR-Robust | 1.131(0.949,1.349) | 0.168 |
| class.Clostridia.id.1859 | IBD | MR-Egger | 1.290(0.451,3.693) | 0.635 |
| class.Clostridia.id.1859 | IBD | MRRAPS | 1.139(0.916,1.418) | 0.242 |
| class.Clostridia.id.1859 | IBD | MRPRESSO | 1.135(0.957,1.345) | 0.173 |
| class.Coriobacteriia.id.809 | IBD | IVW | 1.055(0.892,1.248) | 0.531 |
| class.Coriobacteriia.id.809 | IBD | Weighted median | 1.110(0.879,1.403) | 0.379 |
| class.Coriobacteriia.id.809 | IBD | Weighted mode | 1.157(0.731,1.832) | 0.533 |
| class.Coriobacteriia.id.809 | IBD | MR-Robust | 1.059(0.889,1.261) | 0.520 |
| class.Coriobacteriia.id.809 | IBD | MR-Egger | 1.173(0.591,2.325) | 0.648 |
| class.Coriobacteriia.id.809 | IBD | MRRAPS | 1.067(0.887,1.282) | 0.491 |
| class.Coriobacteriia.id.809 | IBD | MRPRESSO | 1.055(0.899,1.239) | 0.521 |
| class.Deltaproteobacteria.id.3087 | IBD | IVW | 1.067(0.907,1.256) | 0.434 |
| class.Deltaproteobacteria.id.3087 | IBD | Weighted median | 1.095(0.875,1.371) | 0.428 |
| class.Deltaproteobacteria.id.3087 | IBD | Weighted mode | 1.125(0.804,1.574) | 0.493 |
| class.Deltaproteobacteria.id.3087 | IBD | MR-Robust | 1.064(0.919,1.232) | 0.408 |
| class.Deltaproteobacteria.id.3087 | IBD | MR-Egger | 1.183(0.750,1.868) | 0.470 |
| class.Deltaproteobacteria.id.3087 | IBD | MRRAPS | 1.067(0.893,1.274) | 0.476 |
| class.Deltaproteobacteria.id.3087 | IBD | MRPRESSO | 1.067(0.934,1.22) | 0.359 |
| class.Erysipelotrichia.id.2147 | IBD | IVW | 1.030(0.850,1.248) | 0.765 |
| class.Erysipelotrichia.id.2147 | IBD | Weighted median | 1.036(0.802,1.338) | 0.787 |
| class.Erysipelotrichia.id.2147 | IBD | Weighted mode | 0.989(0.645,1.516) | 0.959 |
| class.Erysipelotrichia.id.2147 | IBD | MR-Robust | 1.013(0.818,1.254) | 0.908 |
| class.Erysipelotrichia.id.2147 | IBD | MR-Egger | 1.389(0.593,3.25) | 0.449 |
| class.Erysipelotrichia.id.2147 | IBD | MRRAPS | 1.026(0.833,1.265) | 0.807 |
| class.Erysipelotrichia.id.2147 | IBD | MRPRESSO | 1.030(0.887,1.195) | 0.706 |
| class.Gammaproteobacteria.id.3303 | IBD | IVW | 0.885(0.703,1.114) | 0.297 |
| class.Gammaproteobacteria.id.3303 | IBD | Weighted median | 0.878(0.662,1.164) | 0.365 |
| class.Gammaproteobacteria.id.3303 | IBD | Weighted mode | 0.884(0.601,1.298) | 0.528 |
| class.Gammaproteobacteria.id.3303 | IBD | MR-Robust | 0.886(0.740,1.060) | 0.186 |
| class.Gammaproteobacteria.id.3303 | IBD | MR-Egger | 0.858(0.383,1.923) | 0.710 |
| class.Gammaproteobacteria.id.3303 | IBD | MRRAPS | 0.884(0.685,1.141) | 0.344 |
| class.Gammaproteobacteria.id.3303 | IBD | MRPRESSO | 0.885(0.828,0.946) | 0.011 |
| class.Lentisphaeria.id.2250 | IBD | IVW | 0.871(0.770,0.986) | 0.028 |
| class.Lentisphaeria.id.2250 | IBD | Weighted median | 0.845(0.718,0.996) | 0.045 |
| class.Lentisphaeria.id.2250 | IBD | Weighted mode | 0.780(0.591,1.028) | 0.078 |
| class.Lentisphaeria.id.2250 | IBD | MR-Robust | 0.870(0.779,0.973) | 0.014 |
| class.Lentisphaeria.id.2250 | IBD | MR-Egger | 1.040(0.678,1.594) | 0.858 |
| class.Lentisphaeria.id.2250 | IBD | MRRAPS | 0.867(0.757,0.994) | 0.040 |
| class.Lentisphaeria.id.2250 | IBD | MRPRESSO | 0.871(0.779,0.973) | 0.044 |
| class.Melainabacteria.id.1589 | IBD | IVW | 0.921(0.815,1.040) | 0.182 |
| class.Melainabacteria.id.1589 | IBD | Weighted median | 0.973(0.832,1.136) | 0.726 |
| class.Melainabacteria.id.1589 | IBD | Weighted mode | 0.999(0.793,1.257) | 0.992 |
| class.Melainabacteria.id.1589 | IBD | MR-Robust | 0.945(0.796,1.122) | 0.517 |
| class.Melainabacteria.id.1589 | IBD | MR-Egger | 0.968(0.671,1.395) | 0.860 |
| class.Melainabacteria.id.1589 | IBD | MRRAPS | 0.935(0.823,1.062) | 0.299 |
| class.Melainabacteria.id.1589 | IBD | MRPRESSO | 0.921(0.815,1.040) | 0.215 |
| class.Methanobacteria.id.119 | IBD | IVW | 0.935(0.853,1.025) | 0.152 |
| class.Methanobacteria.id.119 | IBD | Weighted median | 0.952(0.837,1.082) | 0.448 |
| class.Methanobacteria.id.119 | IBD | Weighted mode | 0.990(0.796,1.231) | 0.927 |
| class.Methanobacteria.id.119 | IBD | MR-Robust | 0.935(0.853,1.026) | 0.156 |
| class.Methanobacteria.id.119 | IBD | MR-Egger | 1.044(0.702,1.554) | 0.830 |
| class.Methanobacteria.id.119 | IBD | MRRAPS | 0.933(0.842,1.035) | 0.191 |
| class.Methanobacteria.id.119 | IBD | MRPRESSO | 0.935(0.853,1.025) | 0.182 |
| class.Mollicutes.id.3920 | IBD | IVW | 1.018(0.869,1.192) | 0.829 |
| class.Mollicutes.id.3920 | IBD | Weighted median | 0.907(0.735,1.121) | 0.367 |
| class.Mollicutes.id.3920 | IBD | Weighted mode | 0.847(0.604,1.189) | 0.337 |
| class.Mollicutes.id.3920 | IBD | MR-Robust | 1.005(0.851,1.187) | 0.952 |
| class.Mollicutes.id.3920 | IBD | MR-Egger | 0.954(0.562,1.620) | 0.862 |
| class.Mollicutes.id.3920 | IBD | MRRAPS | 0.998(0.834,1.193) | 0.979 |
| class.Mollicutes.id.3920 | IBD | MRPRESSO | 1.018(0.869,1.192) | 0.833 |
| class.Negativicutes.id.2164 | IBD | IVW | 0.907(0.735,1.119) | 0.361 |
| class.Negativicutes.id.2164 | IBD | Weighted median | 0.892(0.679,1.170) | 0.408 |
| class.Negativicutes.id.2164 | IBD | Weighted mode | 0.980(0.637,1.509) | 0.928 |
| class.Negativicutes.id.2164 | IBD | MR-Robust | 0.903(0.740,1.101) | 0.312 |
| class.Negativicutes.id.2164 | IBD | MR-Egger | 0.710(0.354,1.424) | 0.334 |
| class.Negativicutes.id.2164 | IBD | MRRAPS | 0.896(0.726,1.105) | 0.303 |
| class.Negativicutes.id.2164 | IBD | MRPRESSO | 0.907(0.735,1.119) | 0.380 |
| class.Verrucomicrobiae.id.4029 | IBD | IVW | 0.971(0.809,1.166) | 0.756 |
| class.Verrucomicrobiae.id.4029 | IBD | Weighted median | 0.851(0.679,1.067) | 0.162 |
| class.Verrucomicrobiae.id.4029 | IBD | Weighted mode | 0.793(0.538,1.168) | 0.240 |
| class.Verrucomicrobiae.id.4029 | IBD | MR-Robust | 0.946(0.739,1.212) | 0.662 |
| class.Verrucomicrobiae.id.4029 | IBD | MR-Egger | 0.576(0.305,1.086) | 0.088 |
| class.Verrucomicrobiae.id.4029 | IBD | MRRAPS | 0.926(0.756,1.134) | 0.458 |
| class.Verrucomicrobiae.id.4029 | IBD | MRPRESSO | 0.971(0.809,1.166) | 0.762 |
| family.Acidaminococcaceae.id.2166 | IBD | IVW | 0.946(0.765,1.172) | 0.613 |
| family.Acidaminococcaceae.id.2166 | IBD | Weighted median | 0.959(0.732,1.256) | 0.762 |
| family.Acidaminococcaceae.id.2166 | IBD | Weighted mode | 1.072(0.709,1.622) | 0.741 |
| family.Acidaminococcaceae.id.2166 | IBD | MR-Robust | 0.944(0.791,1.128) | 0.528 |
| family.Acidaminococcaceae.id.2166 | IBD | MR-Egger | 0.985(0.520,1.866) | 0.962 |
| family.Acidaminococcaceae.id.2166 | IBD | MRRAPS | 0.945(0.749,1.193) | 0.635 |
| family.Acidaminococcaceae.id.2166 | IBD | MRPRESSO | 0.946(0.812,1.104) | 0.509 |
| family.Actinomycetaceae.id.421 | IBD | IVW | 1.049(0.857,1.284) | 0.643 |
| family.Actinomycetaceae.id.421 | IBD | Weighted median | 1.144(0.884,1.480) | 0.306 |
| family.Actinomycetaceae.id.421 | IBD | Weighted mode | 1.195(0.865,1.651) | 0.281 |
| family.Actinomycetaceae.id.421 | IBD | MR-Robust | 1.113(0.702,1.764) | 0.650 |
| family.Actinomycetaceae.id.421 | IBD | MR-Egger | 1.009(0.524,1.941) | 0.979 |
| family.Actinomycetaceae.id.421 | IBD | MRRAPS | 1.074(0.867,1.331) | 0.512 |
| family.Actinomycetaceae.id.421 | IBD | MRPRESSO | 1.049(0.857,1.284) | 0.667 |
| family.Alcaligenaceae.id.2875 | IBD | IVW | 0.989(0.833,1.174) | 0.896 |
| family.Alcaligenaceae.id.2875 | IBD | Weighted median | 1.008(0.808,1.258) | 0.943 |
| family.Alcaligenaceae.id.2875 | IBD | Weighted mode | 0.991(0.683,1.437) | 0.961 |
| family.Alcaligenaceae.id.2875 | IBD | MR-Robust | 0.989(0.848,1.154) | 0.888 |
| family.Alcaligenaceae.id.2875 | IBD | MR-Egger | 0.686(0.327,1.438) | 0.318 |
| family.Alcaligenaceae.id.2875 | IBD | MRRAPS | 0.988(0.819,1.193) | 0.903 |
| family.Alcaligenaceae.id.2875 | IBD | MRPRESSO | 0.989(0.897,1.089) | 0.820 |
| family.Bacteroidaceae.id.917 | IBD | IVW | 1.082(0.849,1.379) | 0.526 |
| family.Bacteroidaceae.id.917 | IBD | Weighted median | 1.150(0.836,1.583) | 0.391 |
| family.Bacteroidaceae.id.917 | IBD | Weighted mode | 1.177(0.685,2.022) | 0.555 |
| family.Bacteroidaceae.id.917 | IBD | MR-Robust | 1.085(0.764,1.541) | 0.649 |
| family.Bacteroidaceae.id.917 | IBD | MR-Egger | 1.628(0.398,6.666) | 0.498 |
| family.Bacteroidaceae.id.917 | IBD | MRRAPS | 1.074(0.827,1.396) | 0.592 |
| family.Bacteroidaceae.id.917 | IBD | MRPRESSO | 1.082(0.867,1.350) | 0.509 |
| family.BacteroidalesS24.7group.id.11173 | IBD | IVW | 0.984(0.839,1.155) | 0.844 |
| family.BacteroidalesS24.7group.id.11173 | IBD | Weighted median | 0.871(0.708,1.070) | 0.189 |
| family.BacteroidalesS24.7group.id.11173 | IBD | Weighted mode | 0.842(0.594,1.194) | 0.335 |
| family.BacteroidalesS24.7group.id.11173 | IBD | MR-Robust | 0.947(0.448,2.005) | 0.887 |
| family.BacteroidalesS24.7group.id.11173 | IBD | MR-Egger | 0.640(0.332,1.235) | 0.183 |
| family.BacteroidalesS24.7group.id.11173 | IBD | MRRAPS | 0.974(0.822,1.155) | 0.764 |
| family.BacteroidalesS24.7group.id.11173 | IBD | MRPRESSO | 0.984(0.839,1.155) | 0.849 |
| family.Bifidobacteriaceae.id.433 | IBD | IVW | 0.828(0.727,0.943) | 0.004 |
| family.Bifidobacteriaceae.id.433 | IBD | Weighted median | 0.841(0.705,1.004) | 0.055 |
| family.Bifidobacteriaceae.id.433 | IBD | Weighted mode | 0.860(0.637,1.162) | 0.326 |
| family.Bifidobacteriaceae.id.433 | IBD | MR-Robust | 0.823(0.717,0.945) | 0.006 |
| family.Bifidobacteriaceae.id.433 | IBD | MR-Egger | 0.850(0.510,1.417) | 0.534 |
| family.Bifidobacteriaceae.id.433 | IBD | MRRAPS | 0.808(0.699,0.935) | 0.004 |
| family.Bifidobacteriaceae.id.433 | IBD | MRPRESSO | 0.828(0.727,0.943) | 0.009 |
| family.Christensenellaceae.id.1866 | IBD | IVW | 0.886(0.738,1.064) | 0.194 |
| family.Christensenellaceae.id.1866 | IBD | Weighted median | 0.858(0.673,1.095) | 0.219 |
| family.Christensenellaceae.id.1866 | IBD | Weighted mode | 0.877(0.610,1.262) | 0.480 |
| family.Christensenellaceae.id.1866 | IBD | MR-Robust | 0.878(0.739,1.042) | 0.137 |
| family.Christensenellaceae.id.1866 | IBD | MR-Egger | 0.909(0.602,1.372) | 0.649 |
| family.Christensenellaceae.id.1866 | IBD | MRRAPS | 0.864(0.713,1.048) | 0.137 |
| family.Christensenellaceae.id.1866 | IBD | MRPRESSO | 0.886(0.738,1.064) | 0.224 |
| family.Clostridiaceae1.id.1869 | IBD | IVW | 0.856(0.683,1.073) | 0.178 |
| family.Clostridiaceae1.id.1869 | IBD | Weighted median | 0.900(0.686,1.180) | 0.444 |
| family.Clostridiaceae1.id.1869 | IBD | Weighted mode | 0.915(0.629,1.332) | 0.644 |
| family.Clostridiaceae1.id.1869 | IBD | MR-Robust | 0.870(0.708,1.070) | 0.187 |
| family.Clostridiaceae1.id.1869 | IBD | MR-Egger | 0.842(0.410,1.728) | 0.638 |
| family.Clostridiaceae1.id.1869 | IBD | MRRAPS | 0.870(0.703,1.078) | 0.202 |
| family.Clostridiaceae1.id.1869 | IBD | MRPRESSO | 0.856(0.683,1.073) | 0.211 |
| family.ClostridialesvadinBB60group.id.11286 | IBD | IVW | 0.927(0.816,1.054) | 0.249 |
| family.ClostridialesvadinBB60group.id.11286 | IBD | Weighted median | 0.972(0.812,1.163) | 0.757 |
| family.ClostridialesvadinBB60group.id.11286 | IBD | Weighted mode | 1.008(0.746,1.363) | 0.957 |
| family.ClostridialesvadinBB60group.id.11286 | IBD | MR-Robust | 0.928(0.816,1.055) | 0.254 |
| family.ClostridialesvadinBB60group.id.11286 | IBD | MR-Egger | 1.143(0.794,1.647) | 0.472 |
| family.ClostridialesvadinBB60group.id.11286 | IBD | MRRAPS | 0.923(0.804,1.061) | 0.259 |
| family.ClostridialesvadinBB60group.id.11286 | IBD | MRPRESSO | 0.927(0.818,1.052) | 0.261 |
| family.Coriobacteriaceae.id.811 | IBD | IVW | 1.055(0.892,1.248) | 0.531 |
| family.Coriobacteriaceae.id.811 | IBD | Weighted median | 1.110(0.879,1.403) | 0.379 |
| family.Coriobacteriaceae.id.811 | IBD | Weighted mode | 1.157(0.731,1.832) | 0.533 |
| family.Coriobacteriaceae.id.811 | IBD | MR-Robust | 1.059(0.889,1.261) | 0.520 |
| family.Coriobacteriaceae.id.811 | IBD | MR-Egger | 1.173(0.591,2.325) | 0.648 |
| family.Coriobacteriaceae.id.811 | IBD | MRRAPS | 1.067(0.887,1.282) | 0.491 |
| family.Coriobacteriaceae.id.811 | IBD | MRPRESSO | 1.055(0.899,1.239) | 0.521 |
| family.Defluviitaleaceae.id.1924 | IBD | IVW | 0.987(0.849,1.147) | 0.860 |
| family.Defluviitaleaceae.id.1924 | IBD | Weighted median | 0.998(0.829,1.200) | 0.979 |
| family.Defluviitaleaceae.id.1924 | IBD | Weighted mode | 1.014(0.759,1.356) | 0.923 |
| family.Defluviitaleaceae.id.1924 | IBD | MR-Robust | 0.975(0.834,1.141) | 0.753 |
| family.Defluviitaleaceae.id.1924 | IBD | MR-Egger | 1.038(0.609,1.770) | 0.891 |
| family.Defluviitaleaceae.id.1924 | IBD | MRRAPS | 0.978(0.844,1.133) | 0.765 |
| family.Defluviitaleaceae.id.1924 | IBD | MRPRESSO | 0.987(0.849,1.147) | 0.863 |
| family.Desulfovibrionaceae.id.3169 | IBD | IVW | 1.119(0.934,1.341) | 0.221 |
| family.Desulfovibrionaceae.id.3169 | IBD | Weighted median | 1.162(0.910,1.484) | 0.227 |
| family.Desulfovibrionaceae.id.3169 | IBD | Weighted mode | 1.162(0.829,1.628) | 0.385 |
| family.Desulfovibrionaceae.id.3169 | IBD | MR-Robust | 1.119(0.972,1.288) | 0.118 |
| family.Desulfovibrionaceae.id.3169 | IBD | MR-Egger | 1.173(0.745,1.848) | 0.490 |
| family.Desulfovibrionaceae.id.3169 | IBD | MRRAPS | 1.122(0.922,1.366) | 0.251 |
| family.Desulfovibrionaceae.id.3169 | IBD | MRPRESSO | 1.119(0.965,1.298) | 0.170 |
| family.Enterobacteriaceae.id.3469 | IBD | IVW | 0.914(0.737,1.133) | 0.413 |
| family.Enterobacteriaceae.id.3469 | IBD | Weighted median | 0.945(0.711,1.256) | 0.696 |
| family.Enterobacteriaceae.id.3469 | IBD | Weighted mode | 1.014(0.646,1.590) | 0.953 |
| family.Enterobacteriaceae.id.3469 | IBD | MR-Robust | 0.923(0.755,1.129) | 0.435 |
| family.Enterobacteriaceae.id.3469 | IBD | MR-Egger | 1.534(0.387,6.085) | 0.543 |
| family.Enterobacteriaceae.id.3469 | IBD | MRRAPS | 0.926(0.734,1.169) | 0.519 |
| family.Enterobacteriaceae.id.3469 | IBD | MRPRESSO | 0.914(0.747,1.118) | 0.412 |
| family.Erysipelotrichaceae.id.2149 | IBD | IVW | 1.030(0.850,1.248) | 0.765 |
| family.Erysipelotrichaceae.id.2149 | IBD | Weighted median | 1.036(0.802,1.338) | 0.787 |
| family.Erysipelotrichaceae.id.2149 | IBD | Weighted mode | 0.989(0.645,1.516) | 0.959 |
| family.Erysipelotrichaceae.id.2149 | IBD | MR-Robust | 1.013(0.818,1.254) | 0.908 |
| family.Erysipelotrichaceae.id.2149 | IBD | MR-Egger | 1.389(0.593,3.250) | 0.449 |
| family.Erysipelotrichaceae.id.2149 | IBD | MRRAPS | 1.026(0.833,1.265) | 0.807 |
| family.Erysipelotrichaceae.id.2149 | IBD | MRPRESSO | 1.030(0.887,1.195) | 0.706 |
| family.FamilyXI.id.1936 | IBD | IVW | 0.974(0.882,1.076) | 0.609 |
| family.FamilyXI.id.1936 | IBD | Weighted median | 0.973(0.855,1.107) | 0.675 |
| family.FamilyXI.id.1936 | IBD | Weighted mode | 0.979(0.799,1.198) | 0.834 |
| family.FamilyXI.id.1936 | IBD | MR-Robust | 0.976(0.892,1.067) | 0.589 |
| family.FamilyXI.id.1936 | IBD | MR-Egger | 0.931(0.483,1.797) | 0.832 |
| family.FamilyXI.id.1936 | IBD | MRRAPS | 0.977(0.877,1.087) | 0.667 |
| family.FamilyXI.id.1936 | IBD | MRPRESSO | 0.974(0.887,1.070) | 0.604 |
| family.FamilyXIII.id.1957 | IBD | IVW | 0.977(0.800,1.194) | 0.823 |
| family.FamilyXIII.id.1957 | IBD | Weighted median | 0.944(0.724,1.232) | 0.671 |
| family.FamilyXIII.id.1957 | IBD | Weighted mode | 0.907(0.593,1.387) | 0.654 |
| family.FamilyXIII.id.1957 | IBD | MR-Robust | 0.968(0.746,1.257) | 0.808 |
| family.FamilyXIII.id.1957 | IBD | MR-Egger | 0.827(0.360,1.900) | 0.654 |
| family.FamilyXIII.id.1957 | IBD | MRRAPS | 0.977(0.785,1.216) | 0.834 |
| family.FamilyXIII.id.1957 | IBD | MRPRESSO | 0.977(0.846,1.129) | 0.763 |
| family.Lachnospiraceae.id.1987 | IBD | IVW | 1.143(0.979,1.334) | 0.092 |
| family.Lachnospiraceae.id.1987 | IBD | Weighted median | 1.196(0.968,1.477) | 0.096 |
| family.Lachnospiraceae.id.1987 | IBD | Weighted mode | 1.241(0.914,1.685) | 0.166 |
| family.Lachnospiraceae.id.1987 | IBD | MR-Robust | 1.159(0.999,1.344) | 0.051 |
| family.Lachnospiraceae.id.1987 | IBD | MR-Egger | 1.243(0.847,1.823) | 0.266 |
| family.Lachnospiraceae.id.1987 | IBD | MRRAPS | 1.159(0.978,1.374) | 0.089 |
| family.Lachnospiraceae.id.1987 | IBD | MRPRESSO | 1.143(1.005,1.300) | 0.059 |
| family.Lactobacillaceae.id.1836 | IBD | IVW | 0.877(0.767,1.004) | 0.056 |
| family.Lactobacillaceae.id.1836 | IBD | Weighted median | 0.883(0.736,1.058) | 0.176 |
| family.Lactobacillaceae.id.1836 | IBD | Weighted mode | 0.882(0.657,1.184) | 0.404 |
| family.Lactobacillaceae.id.1836 | IBD | MR-Robust | 0.869(0.733,1.029) | 0.104 |
| family.Lactobacillaceae.id.1836 | IBD | MR-Egger | 0.896(0.605,1.327) | 0.584 |
| family.Lactobacillaceae.id.1836 | IBD | MRRAPS | 0.865(0.747,1.001) | 0.052 |
| family.Lactobacillaceae.id.1836 | IBD | MRPRESSO | 0.877(0.767,1.004) | 0.089 |
| family.Methanobacteriaceae.id.121 | IBD | IVW | 0.935(0.853,1.025) | 0.152 |
| family.Methanobacteriaceae.id.121 | IBD | Weighted median | 0.952(0.837,1.082) | 0.448 |
| family.Methanobacteriaceae.id.121 | IBD | Weighted mode | 0.990(0.796,1.231) | 0.927 |
| family.Methanobacteriaceae.id.121 | IBD | MR-Robust | 0.935(0.853,1.026) | 0.156 |
| family.Methanobacteriaceae.id.121 | IBD | MR-Egger | 1.044(0.702,1.554) | 0.830 |
| family.Methanobacteriaceae.id.121 | IBD | MRRAPS | 0.933(0.842,1.035) | 0.191 |
| family.Methanobacteriaceae.id.121 | IBD | MRPRESSO | 0.935(0.853,1.025) | 0.182 |
| family.Oxalobacteraceae.id.2966 | IBD | IVW | 1.103(0.984,1.236) | 0.091 |
| family.Oxalobacteraceae.id.2966 | IBD | Weighted median | 1.169(1.021,1.339) | 0.024 |
| family.Oxalobacteraceae.id.2966 | IBD | Weighted mode | 1.185(0.952,1.476) | 0.129 |
| family.Oxalobacteraceae.id.2966 | IBD | MR-Robust | 1.116(0.984,1.265) | 0.087 |
| family.Oxalobacteraceae.id.2966 | IBD | MR-Egger | 0.974(0.609,1.558) | 0.914 |
| family.Oxalobacteraceae.id.2966 | IBD | MRRAPS | 1.118(0.997,1.253) | 0.056 |
| family.Oxalobacteraceae.id.2966 | IBD | MRPRESSO | 1.103(0.984,1.236) | 0.115 |
| family.Pasteurellaceae.id.3689 | IBD | IVW | 0.993(0.867,1.138) | 0.922 |
| family.Pasteurellaceae.id.3689 | IBD | Weighted median | 0.915(0.771,1.086) | 0.311 |
| family.Pasteurellaceae.id.3689 | IBD | Weighted mode | 0.869(0.649,1.165) | 0.348 |
| family.Pasteurellaceae.id.3689 | IBD | MR-Robust | 0.962(0.763,1.214) | 0.746 |
| family.Pasteurellaceae.id.3689 | IBD | MR-Egger | 0.918(0.669,1.261) | 0.599 |
| family.Pasteurellaceae.id.3689 | IBD | MRRAPS | 0.961(0.825,1.118) | 0.605 |
| family.Pasteurellaceae.id.3689 | IBD | MRPRESSO | 0.993(0.867,1.138) | 0.924 |
| family.Peptococcaceae.id.2024 | IBD | IVW | 0.898(0.751,1.073) | 0.235 |
| family.Peptococcaceae.id.2024 | IBD | Weighted median | 0.817(0.665,1.004) | 0.055 |
| family.Peptococcaceae.id.2024 | IBD | Weighted mode | 0.824(0.64,1.061) | 0.134 |
| family.Peptococcaceae.id.2024 | IBD | MR-Robust | 0.848(0.697,1.033) | 0.102 |
| family.Peptococcaceae.id.2024 | IBD | MR-Egger | 0.991(0.632,1.554) | 0.969 |
| family.Peptococcaceae.id.2024 | IBD | MRRAPS | 0.866(0.739,1.016) | 0.077 |
| family.Peptococcaceae.id.2024 | IBD | MRPRESSO | 0.898(0.751,1.073) | 0.265 |
| family.Peptostreptococcaceae.id.2042 | IBD | IVW | 1.014(0.871,1.182) | 0.853 |
| family.Peptostreptococcaceae.id.2042 | IBD | Weighted median | 1.032(0.825,1.290) | 0.784 |
| family.Peptostreptococcaceae.id.2042 | IBD | Weighted mode | 1.084(0.722,1.628) | 0.697 |
| family.Peptostreptococcaceae.id.2042 | IBD | MR-Robust | 1.054(0.849,1.308) | 0.634 |
| family.Peptostreptococcaceae.id.2042 | IBD | MR-Egger | 0.787(0.554,1.118) | 0.181 |
| family.Peptostreptococcaceae.id.2042 | IBD | MRRAPS | 1.021(0.843,1.238) | 0.829 |
| family.Peptostreptococcaceae.id.2042 | IBD | MRPRESSO | 1.014(0.871,1.182) | 0.856 |
| family.Porphyromonadaceae.id.943 | IBD | IVW | 1.001(0.741,1.351) | 0.997 |
| family.Porphyromonadaceae.id.943 | IBD | Weighted median | 1.147(0.838,1.57) | 0.392 |
| family.Porphyromonadaceae.id.943 | IBD | Weighted mode | 1.238(0.774,1.982) | 0.373 |
| family.Porphyromonadaceae.id.943 | IBD | MR-Robust | 1.032(0.709,1.501) | 0.871 |
| family.Porphyromonadaceae.id.943 | IBD | MR-Egger | 2.577(0.927,7.16) | 0.069 |
| family.Porphyromonadaceae.id.943 | IBD | MRRAPS | 1.075(0.794,1.456) | 0.641 |
| family.Porphyromonadaceae.id.943 | IBD | MRPRESSO | 1.001(0.741,1.351) | 0.997 |
| family.Prevotellaceae.id.960 | IBD | IVW | 0.906(0.758,1.082) | 0.274 |
| family.Prevotellaceae.id.960 | IBD | Weighted median | 0.845(0.677,1.054) | 0.135 |
| family.Prevotellaceae.id.960 | IBD | Weighted mode | 0.718(0.464,1.11) | 0.136 |
| family.Prevotellaceae.id.960 | IBD | MR-Robust | 0.894(0.722,1.106) | 0.302 |
| family.Prevotellaceae.id.960 | IBD | MR-Egger | 1.214(0.645,2.285) | 0.549 |
| family.Prevotellaceae.id.960 | IBD | MRRAPS | 0.919(0.748,1.128) | 0.417 |
| family.Prevotellaceae.id.960 | IBD | MRPRESSO | 0.906(0.758,1.082) | 0.292 |
| family.Rhodospirillaceae.id.2717 | IBD | IVW | 1.072(0.947,1.213) | 0.271 |
| family.Rhodospirillaceae.id.2717 | IBD | Weighted median | 1.048(0.884,1.244) | 0.587 |
| family.Rhodospirillaceae.id.2717 | IBD | Weighted mode | 1.079(0.808,1.44) | 0.608 |
| family.Rhodospirillaceae.id.2717 | IBD | MR-Robust | 1.079(0.938,1.242) | 0.285 |
| family.Rhodospirillaceae.id.2717 | IBD | MR-Egger | 0.749(0.371,1.513) | 0.421 |
| family.Rhodospirillaceae.id.2717 | IBD | MRRAPS | 1.083(0.947,1.238) | 0.244 |
| family.Rhodospirillaceae.id.2717 | IBD | MRPRESSO | 1.072(0.953,1.206) | 0.267 |
| family.Rikenellaceae.id.967 | IBD | IVW | 1.107(0.959,1.279) | 0.165 |
| family.Rikenellaceae.id.967 | IBD | Weighted median | 1.112(0.908,1.362) | 0.305 |
| family.Rikenellaceae.id.967 | IBD | Weighted mode | 0.864(0.595,1.255) | 0.442 |
| family.Rikenellaceae.id.967 | IBD | MR-Robust | 1.104(0.958,1.273) | 0.173 |
| family.Rikenellaceae.id.967 | IBD | MR-Egger | 1.144(0.716,1.828) | 0.573 |
| family.Rikenellaceae.id.967 | IBD | MRRAPS | 1.103(0.944,1.288) | 0.218 |
| family.Rikenellaceae.id.967 | IBD | MRPRESSO | 1.107(0.959,1.279) | 0.181 |
| family.Ruminococcaceae.id.2050 | IBD | IVW | 0.905(0.749,1.093) | 0.300 |
| family.Ruminococcaceae.id.2050 | IBD | Weighted median | 0.897(0.686,1.171) | 0.424 |
| family.Ruminococcaceae.id.2050 | IBD | Weighted mode | 0.848(0.573,1.255) | 0.409 |
| family.Ruminococcaceae.id.2050 | IBD | MR-Robust | 0.906(0.747,1.099) | 0.317 |
| family.Ruminococcaceae.id.2050 | IBD | MR-Egger | 0.83(0.553,1.247) | 0.370 |
| family.Ruminococcaceae.id.2050 | IBD | MRRAPS | 0.912(0.744,1.118) | 0.375 |
| family.Ruminococcaceae.id.2050 | IBD | MRPRESSO | 0.905(0.755,1.085) | 0.308 |
| family.Streptococcaceae.id.1850 | IBD | IVW | 1.055(0.898,1.238) | 0.517 |
| family.Streptococcaceae.id.1850 | IBD | Weighted median | 1.089(0.873,1.358) | 0.451 |
| family.Streptococcaceae.id.1850 | IBD | Weighted mode | 1.162(0.783,1.726) | 0.456 |
| family.Streptococcaceae.id.1850 | IBD | MR-Robust | 1.078(0.904,1.284) | 0.402 |
| family.Streptococcaceae.id.1850 | IBD | MR-Egger | 0.994(0.531,1.861) | 0.984 |
| family.Streptococcaceae.id.1850 | IBD | MRRAPS | 1.088(0.92,1.288) | 0.325 |
| family.Streptococcaceae.id.1850 | IBD | MRPRESSO | 1.055(0.898,1.238) | 0.526 |
| family.unknownfamily.id.1000001214 | IBD | IVW | 0.968(0.837,1.12) | 0.660 |
| family.unknownfamily.id.1000001214 | IBD | Weighted median | 0.979(0.828,1.158) | 0.803 |
| family.unknownfamily.id.1000001214 | IBD | Weighted mode | 1.006(0.803,1.261) | 0.956 |
| family.unknownfamily.id.1000001214 | IBD | MR-Robust | 0.971(0.853,1.104) | 0.650 |
| family.unknownfamily.id.1000001214 | IBD | MR-Egger | 0.871(0.566,1.342) | 0.531 |
| family.unknownfamily.id.1000001214 | IBD | MRRAPS | 0.967(0.848,1.103) | 0.618 |
| family.unknownfamily.id.1000001214 | IBD | MRPRESSO | 0.968(0.837,1.12) | 0.672 |
| family.unknownfamily.id.1000005471 | IBD | IVW | 0.974(0.851,1.115) | 0.703 |
| family.unknownfamily.id.1000005471 | IBD | Weighted median | 0.948(0.793,1.132) | 0.552 |
| family.unknownfamily.id.1000005471 | IBD | Weighted mode | 0.843(0.622,1.143) | 0.272 |
| family.unknownfamily.id.1000005471 | IBD | MR-Robust | 0.975(0.867,1.096) | 0.671 |
| family.unknownfamily.id.1000005471 | IBD | MR-Egger | 0.805(0.543,1.192) | 0.278 |
| family.unknownfamily.id.1000005471 | IBD | MRRAPS | 0.973(0.84,1.128) | 0.720 |
| family.unknownfamily.id.1000005471 | IBD | MRPRESSO | 0.974(0.885,1.073) | 0.603 |
| family.unknownfamily.id.1000006161 | IBD | IVW | 1.124(0.995,1.27) | 0.061 |
| family.unknownfamily.id.1000006161 | IBD | Weighted median | 1.078(0.941,1.236) | 0.277 |
| family.unknownfamily.id.1000006161 | IBD | Weighted mode | 1.084(0.871,1.35) | 0.470 |
| family.unknownfamily.id.1000006161 | IBD | MR-Robust | 1.122(1,1.258) | 0.049 |
| family.unknownfamily.id.1000006161 | IBD | MR-Egger | 0.81(0.499,1.316) | 0.396 |
| family.unknownfamily.id.1000006161 | IBD | MRRAPS | 1.121(0.991,1.268) | 0.068 |
| family.unknownfamily.id.1000006161 | IBD | MRPRESSO | 1.124(0.995,1.27) | 0.083 |
| family.Veillonellaceae.id.2172 | IBD | IVW | 0.966(0.848,1.101) | 0.606 |
| family.Veillonellaceae.id.2172 | IBD | Weighted median | 1.017(0.847,1.221) | 0.854 |
| family.Veillonellaceae.id.2172 | IBD | Weighted mode | 1.055(0.808,1.376) | 0.695 |
| family.Veillonellaceae.id.2172 | IBD | MR-Robust | 0.965(0.861,1.081) | 0.534 |
| family.Veillonellaceae.id.2172 | IBD | MR-Egger | 0.977(0.733,1.302) | 0.875 |
| family.Veillonellaceae.id.2172 | IBD | MRRAPS | 0.959(0.833,1.105) | 0.564 |
| family.Veillonellaceae.id.2172 | IBD | MRPRESSO | 0.966(0.856,1.09) | 0.585 |
| family.Verrucomicrobiaceae.id.4036 | IBD | IVW | 0.971(0.809,1.166) | 0.755 |
| family.Verrucomicrobiaceae.id.4036 | IBD | Weighted median | 0.851(0.679,1.066) | 0.161 |
| family.Verrucomicrobiaceae.id.4036 | IBD | Weighted mode | 0.793(0.538,1.168) | 0.240 |
| family.Verrucomicrobiaceae.id.4036 | IBD | MR-Robust | 0.946(0.739,1.212) | 0.662 |
| family.Verrucomicrobiaceae.id.4036 | IBD | MR-Egger | 0.575(0.305,1.085) | 0.088 |
| family.Verrucomicrobiaceae.id.4036 | IBD | MRRAPS | 0.926(0.756,1.134) | 0.458 |
| family.Verrucomicrobiaceae.id.4036 | IBD | MRPRESSO | 0.971(0.809,1.166) | 0.761 |
| family.Victivallaceae.id.2255 | IBD | IVW | 1.025(0.937,1.122) | 0.586 |
| family.Victivallaceae.id.2255 | IBD | Weighted median | 1.017(0.901,1.148) | 0.788 |
| family.Victivallaceae.id.2255 | IBD | Weighted mode | 1.011(0.828,1.235) | 0.912 |
| family.Victivallaceae.id.2255 | IBD | MR-Robust | 1.031(0.943,1.127) | 0.499 |
| family.Victivallaceae.id.2255 | IBD | MR-Egger | 0.796(0.528,1.198) | 0.274 |
| family.Victivallaceae.id.2255 | IBD | MRRAPS | 1.038(0.944,1.141) | 0.441 |
| family.Victivallaceae.id.2255 | IBD | MRPRESSO | 1.025(0.937,1.122) | 0.596 |
| genus Clostridiuminnocuumgroup.id.14397 | IBD | IVW | 0.938(0.839,1.049) | 0.263 |
| genus Clostridiuminnocuumgroup.id.14397 | IBD | Weighted median | 0.918(0.792,1.064) | 0.257 |
| genus Clostridiuminnocuumgroup.id.14397 | IBD | Weighted mode | 0.914(0.728,1.148) | 0.439 |
| genus Clostridiuminnocuumgroup.id.14397 | IBD | MR-Robust | 0.933(0.835,1.043) | 0.221 |
| genus Clostridiuminnocuumgroup.id.14397 | IBD | MR-Egger | 1.212(0.687,2.137) | 0.507 |
| genus Clostridiuminnocuumgroup.id.14397 | IBD | MRRAPS | 0.937(0.828,1.059) | 0.296 |
| genus Clostridiuminnocuumgroup.id.14397 | IBD | MRPRESSO | 0.938(0.865,1.017) | 0.161 |
| genus Eubacteriumbrachygroup.id.11296 | IBD | IVW | 0.952(0.852,1.064) | 0.385 |
| genus Eubacteriumbrachygroup.id.11296 | IBD | Weighted median | 0.939(0.807,1.093) | 0.418 |
| genus Eubacteriumbrachygroup.id.11296 | IBD | Weighted mode | 0.917(0.731,1.149) | 0.450 |
| genus Eubacteriumbrachygroup.id.11296 | IBD | MR-Robust | 0.922(0.774,1.099) | 0.364 |
| genus Eubacteriumbrachygroup.id.11296 | IBD | MR-Egger | 0.752(0.458,1.234) | 0.259 |
| genus Eubacteriumbrachygroup.id.11296 | IBD | MRRAPS | 0.938(0.831,1.059) | 0.303 |
| genus Eubacteriumbrachygroup.id.11296 | IBD | MRPRESSO | 0.952(0.863,1.05) | 0.353 |
| genus Eubacteriumcoprostanoligenesgroup.id.11375 | IBD | IVW | 0.947(0.788,1.138) | 0.564 |
| genus Eubacteriumcoprostanoligenesgroup.id.11375 | IBD | Weighted median | 1.029(0.806,1.315) | 0.817 |
| genus Eubacteriumcoprostanoligenesgroup.id.11375 | IBD | Weighted mode | 1.108(0.723,1.7) | 0.637 |
| genus Eubacteriumcoprostanoligenesgroup.id.11375 | IBD | MR-Robust | 0.952(0.794,1.141) | 0.595 |
| genus Eubacteriumcoprostanoligenesgroup.id.11375 | IBD | MR-Egger | 1.547(0.754,3.174) | 0.234 |
| genus Eubacteriumcoprostanoligenesgroup.id.11375 | IBD | MRRAPS | 0.946(0.775,1.155) | 0.584 |
| genus Eubacteriumcoprostanoligenesgroup.id.11375 | IBD | MRPRESSO | 0.947(0.815,1.101) | 0.494 |
| genus Eubacteriumeligensgroup.id.14372 | IBD | IVW | 1.298(1.014,1.662) | 0.038 |
| genus Eubacteriumeligensgroup.id.14372 | IBD | Weighted median | 1.251(0.915,1.712) | 0.161 |
| genus Eubacteriumeligensgroup.id.14372 | IBD | Weighted mode | 1.266(0.795,2.017) | 0.321 |
| genus Eubacteriumeligensgroup.id.14372 | IBD | MR-Robust | 1.236(0.954,1.601) | 0.108 |
| genus Eubacteriumeligensgroup.id.14372 | IBD | MR-Egger | 1.138(0.416,3.111) | 0.801 |
| genus Eubacteriumeligensgroup.id.14372 | IBD | MRRAPS | 1.248(0.975,1.597) | 0.078 |
| genus Eubacteriumeligensgroup.id.14372 | IBD | MRPRESSO | 1.298(1.014,1.662) | 0.077 |
| genus Eubacteriumfissicatenagroup.id.14373 | IBD | IVW | 1.04(0.934,1.158) | 0.472 |
| genus Eubacteriumfissicatenagroup.id.14373 | IBD | Weighted median | 1.049(0.903,1.219) | 0.532 |
| genus Eubacteriumfissicatenagroup.id.14373 | IBD | Weighted mode | 1.062(0.829,1.36) | 0.636 |
| genus Eubacteriumfissicatenagroup.id.14373 | IBD | MR-Robust | 1.043(0.935,1.164) | 0.452 |
| genus Eubacteriumfissicatenagroup.id.14373 | IBD | MR-Egger | 1.255(0.696,2.263) | 0.450 |
| genus Eubacteriumfissicatenagroup.id.14373 | IBD | MRRAPS | 1.049(0.934,1.179) | 0.421 |
| genus Eubacteriumfissicatenagroup.id.14373 | IBD | MRPRESSO | 1.04(0.936,1.156) | 0.484 |
| genus Eubacteriumhalliigroup.id.11338 | IBD | IVW | 0.834(0.649,1.071) | 0.155 |
| genus Eubacteriumhalliigroup.id.11338 | IBD | Weighted median | 0.826(0.65,1.051) | 0.119 |
| genus Eubacteriumhalliigroup.id.11338 | IBD | Weighted mode | 0.871(0.616,1.231) | 0.433 |
| genus Eubacteriumhalliigroup.id.11338 | IBD | MR-Robust | 0.845(0.668,1.069) | 0.161 |
| genus Eubacteriumhalliigroup.id.11338 | IBD | MR-Egger | 1.529(0.971,2.409) | 0.067 |
| genus Eubacteriumhalliigroup.id.11338 | IBD | MRRAPS | 0.925(0.739,1.158) | 0.497 |
| genus Eubacteriumhalliigroup.id.11338 | IBD | MRPRESSO | 0.834(0.649,1.071) | 0.175 |
| genus Eubacteriumnodatumgroup.id.11297 | IBD | IVW | 0.965(0.884,1.054) | 0.426 |
| genus Eubacteriumnodatumgroup.id.11297 | IBD | Weighted median | 0.99(0.881,1.113) | 0.867 |
| genus Eubacteriumnodatumgroup.id.11297 | IBD | Weighted mode | 0.99(0.831,1.18) | 0.913 |
| genus Eubacteriumnodatumgroup.id.11297 | IBD | MR-Robust | 0.974(0.893,1.062) | 0.549 |
| genus Eubacteriumnodatumgroup.id.11297 | IBD | MR-Egger | 0.968(0.656,1.427) | 0.868 |
| genus Eubacteriumnodatumgroup.id.11297 | IBD | MRRAPS | 0.971(0.882,1.068) | 0.544 |
| genus Eubacteriumnodatumgroup.id.11297 | IBD | MRPRESSO | 0.965(0.898,1.037) | 0.352 |
| genus Eubacteriumoxidoreducensgroup.id.11339 | IBD | IVW | 0.863(0.682,1.091) | 0.217 |
| genus Eubacteriumoxidoreducensgroup.id.11339 | IBD | Weighted median | 0.865(0.677,1.106) | 0.248 |
| genus Eubacteriumoxidoreducensgroup.id.11339 | IBD | Weighted mode | 1.013(0.676,1.518) | 0.950 |
| genus Eubacteriumoxidoreducensgroup.id.11339 | IBD | MR-Robust | 0.865(0.688,1.088) | 0.216 |
| genus Eubacteriumoxidoreducensgroup.id.11339 | IBD | MR-Egger | 0.611(0.299,1.251) | 0.178 |
| genus Eubacteriumoxidoreducensgroup.id.11339 | IBD | MRRAPS | 0.832(0.628,1.1) | 0.197 |
| genus Eubacteriumoxidoreducensgroup.id.11339 | IBD | MRPRESSO | 0.863(0.682,1.091) | 0.305 |
| genus Eubacteriumrectalegroup.id.14374 | IBD | IVW | 1.176(0.944,1.464) | 0.148 |
| genus Eubacteriumrectalegroup.id.14374 | IBD | Weighted median | 1.21(0.906,1.616) | 0.196 |
| genus Eubacteriumrectalegroup.id.14374 | IBD | Weighted mode | 1.271(0.828,1.952) | 0.273 |
| genus Eubacteriumrectalegroup.id.14374 | IBD | MR-Robust | 1.201(0.957,1.508) | 0.114 |
| genus Eubacteriumrectalegroup.id.14374 | IBD | MR-Egger | 0.903(0.407,2.005) | 0.803 |
| genus Eubacteriumrectalegroup.id.14374 | IBD | MRRAPS | 1.199(0.943,1.526) | 0.139 |
| genus Eubacteriumrectalegroup.id.14374 | IBD | MRPRESSO | 1.176(0.976,1.417) | 0.127 |
| genus Eubacteriumruminantiumgroup.id.11340 | IBD | IVW | 1.074(0.966,1.193) | 0.188 |
| genus Eubacteriumruminantiumgroup.id.11340 | IBD | Weighted median | 1.103(0.961,1.267) | 0.163 |
| genus Eubacteriumruminantiumgroup.id.11340 | IBD | Weighted mode | 1.121(0.873,1.438) | 0.370 |
| genus Eubacteriumruminantiumgroup.id.11340 | IBD | MR-Robust | 1.082(0.973,1.203) | 0.148 |
| genus Eubacteriumruminantiumgroup.id.11340 | IBD | MR-Egger | 1.108(0.746,1.645) | 0.612 |
| genus Eubacteriumruminantiumgroup.id.11340 | IBD | MRRAPS | 1.08(0.962,1.213) | 0.192 |
| genus Eubacteriumruminantiumgroup.id.11340 | IBD | MRPRESSO | 1.074(0.998,1.155) | 0.073 |
| genus Eubacteriumventriosumgroup.id.11341 | IBD | IVW | 0.849(0.7,1.03) | 0.097 |
| genus Eubacteriumventriosumgroup.id.11341 | IBD | Weighted median | 0.807(0.632,1.031) | 0.086 |
| genus Eubacteriumventriosumgroup.id.11341 | IBD | Weighted mode | 0.698(0.444,1.098) | 0.120 |
| genus Eubacteriumventriosumgroup.id.11341 | IBD | MR-Robust | 0.845(0.695,1.028) | 0.092 |
| genus Eubacteriumventriosumgroup.id.11341 | IBD | MR-Egger | 0.596(0.248,1.429) | 0.246 |
| genus Eubacteriumventriosumgroup.id.11341 | IBD | MRRAPS | 0.833(0.678,1.025) | 0.084 |
| genus Eubacteriumventriosumgroup.id.11341 | IBD | MRPRESSO | 0.849(0.7,1.03) | 0.119 |
| genus Eubacteriumxylanophilumgroup.id.14375 | IBD | IVW | 1.084(0.892,1.318) | 0.415 |
| genus Eubacteriumxylanophilumgroup.id.14375 | IBD | Weighted median | 1.171(0.904,1.517) | 0.232 |
| genus Eubacteriumxylanophilumgroup.id.14375 | IBD | Weighted mode | 1.232(0.832,1.826) | 0.298 |
| genus Eubacteriumxylanophilumgroup.id.14375 | IBD | MR-Robust | 1.094(0.919,1.301) | 0.312 |
| genus Eubacteriumxylanophilumgroup.id.14375 | IBD | MR-Egger | 0.841(0.455,1.552) | 0.579 |
| genus Eubacteriumxylanophilumgroup.id.14375 | IBD | MRRAPS | 1.101(0.891,1.36) | 0.373 |
| genus Eubacteriumxylanophilumgroup.id.14375 | IBD | MRPRESSO | 1.084(0.907,1.296) | 0.399 |
| genus Ruminococcusgauvreauiigroup.id.11342 | IBD | IVW | 0.927(0.736,1.168) | 0.521 |
| genus Ruminococcusgauvreauiigroup.id.11342 | IBD | Weighted median | 0.975(0.768,1.239) | 0.837 |
| genus Ruminococcusgauvreauiigroup.id.11342 | IBD | Weighted mode | 1.018(0.713,1.452) | 0.923 |
| genus Ruminococcusgauvreauiigroup.id.11342 | IBD | MR-Robust | 0.968(0.809,1.158) | 0.721 |
| genus Ruminococcusgauvreauiigroup.id.11342 | IBD | MR-Egger | 0.789(0.256,2.428) | 0.679 |
| genus Ruminococcusgauvreauiigroup.id.11342 | IBD | MRRAPS | 0.946(0.776,1.153) | 0.584 |
| genus Ruminococcusgauvreauiigroup.id.11342 | IBD | MRPRESSO | 0.927(0.736,1.168) | 0.534 |
| genus Ruminococcusgnavusgroup.id.14376 | IBD | IVW | 0.997(0.875,1.135) | 0.959 |
| genus Ruminococcusgnavusgroup.id.14376 | IBD | Weighted median | 0.969(0.831,1.129) | 0.683 |
| genus Ruminococcusgnavusgroup.id.14376 | IBD | Weighted mode | 0.956(0.764,1.197) | 0.697 |
| genus Ruminococcusgnavusgroup.id.14376 | IBD | MR-Robust | 0.935(0.852,1.026) | 0.158 |
| genus Ruminococcusgnavusgroup.id.14376 | IBD | MR-Egger | 0.61(0.354,1.052) | 0.075 |
| genus Ruminococcusgnavusgroup.id.14376 | IBD | MRRAPS | 0.96(0.846,1.089) | 0.526 |
| genus Ruminococcusgnavusgroup.id.14376 | IBD | MRPRESSO | 0.997(0.875,1.135) | 0.960 |
| genus Ruminococcustorquesgroup.id.14377 | IBD | IVW | 0.943(0.67,1.328) | 0.738 |
| genus Ruminococcustorquesgroup.id.14377 | IBD | Weighted median | 0.858(0.628,1.174) | 0.339 |
| genus Ruminococcustorquesgroup.id.14377 | IBD | Weighted mode | 0.862(0.542,1.372) | 0.532 |
| genus Ruminococcustorquesgroup.id.14377 | IBD | MR-Robust | 0.93(0.643,1.346) | 0.701 |
| genus Ruminococcustorquesgroup.id.14377 | IBD | MR-Egger | 0.542(0.227,1.297) | 0.169 |
| genus Ruminococcustorquesgroup.id.14377 | IBD | MRRAPS | 0.874(0.646,1.183) | 0.384 |
| genus Ruminococcustorquesgroup.id.14377 | IBD | MRPRESSO | 0.943(0.67,1.328) | 0.746 |
| genus.Actinomyces.id.423 | IBD | IVW | 0.988(0.833,1.172) | 0.889 |
| genus.Actinomyces.id.423 | IBD | Weighted median | 1.099(0.895,1.35) | 0.369 |
| genus.Actinomyces.id.423 | IBD | Weighted mode | 1.15(0.867,1.525) | 0.333 |
| genus.Actinomyces.id.423 | IBD | MR-Robust | 0.993(0.824,1.196) | 0.940 |
| genus.Actinomyces.id.423 | IBD | MR-Egger | 1.246(0.781,1.989) | 0.357 |
| genus.Actinomyces.id.423 | IBD | MRRAPS | 0.998(0.831,1.199) | 0.986 |
| genus.Actinomyces.id.423 | IBD | MRPRESSO | 0.988(0.833,1.172) | 0.893 |
| genus.Adlercreutzia.id.812 | IBD | IVW | 1.024(0.862,1.216) | 0.785 |
| genus.Adlercreutzia.id.812 | IBD | Weighted median | 1.001(0.801,1.25) | 0.995 |
| genus.Adlercreutzia.id.812 | IBD | Weighted mode | 0.985(0.681,1.424) | 0.936 |
| genus.Adlercreutzia.id.812 | IBD | MR-Robust | 1.031(0.868,1.226) | 0.726 |
| genus.Adlercreutzia.id.812 | IBD | MR-Egger | 1.479(0.666,3.283) | 0.337 |
| genus.Adlercreutzia.id.812 | IBD | MRRAPS | 1.047(0.875,1.254) | 0.615 |
| genus.Adlercreutzia.id.812 | IBD | MRPRESSO | 1.024(0.862,1.216) | 0.792 |
| genus.Akkermansia.id.4037 | IBD | IVW | 0.971(0.809,1.166) | 0.754 |
| genus.Akkermansia.id.4037 | IBD | Weighted median | 0.85(0.678,1.065) | 0.159 |
| genus.Akkermansia.id.4037 | IBD | Weighted mode | 0.793(0.539,1.167) | 0.239 |
| genus.Akkermansia.id.4037 | IBD | MR-Robust | 0.946(0.738,1.212) | 0.661 |
| genus.Akkermansia.id.4037 | IBD | MR-Egger | 0.575(0.305,1.084) | 0.087 |
| genus.Akkermansia.id.4037 | IBD | MRRAPS | 0.926(0.756,1.134) | 0.457 |
| genus.Akkermansia.id.4037 | IBD | MRPRESSO | 0.971(0.809,1.166) | 0.760 |
| genus.Alistipes.id.968 | IBD | IVW | 1.064(0.858,1.32) | 0.573 |
| genus.Alistipes.id.968 | IBD | Weighted median | 1.154(0.885,1.506) | 0.291 |
| genus.Alistipes.id.968 | IBD | Weighted mode | 1.323(0.802,2.18) | 0.273 |
| genus.Alistipes.id.968 | IBD | MR-Robust | 1.075(0.85,1.359) | 0.547 |
| genus.Alistipes.id.968 | IBD | MR-Egger | 2.635(1.015,6.845) | 0.047 |
| genus.Alistipes.id.968 | IBD | MRRAPS | 1.104(0.876,1.392) | 0.403 |
| genus.Alistipes.id.968 | IBD | MRPRESSO | 1.064(0.858,1.32) | 0.582 |
| genus.Allisonella.id.2174 | IBD | IVW | 1.047(0.939,1.167) | 0.411 |
| genus.Allisonella.id.2174 | IBD | Weighted median | 1.041(0.91,1.192) | 0.556 |
| genus.Allisonella.id.2174 | IBD | Weighted mode | 1.039(0.868,1.245) | 0.674 |
| genus.Allisonella.id.2174 | IBD | MR-Robust | 1.018(0.86,1.204) | 0.838 |
| genus.Allisonella.id.2174 | IBD | MR-Egger | 0.548(0.28,1.07) | 0.078 |
| genus.Allisonella.id.2174 | IBD | MRRAPS | 1.032(0.926,1.151) | 0.570 |
| genus.Allisonella.id.2174 | IBD | MRPRESSO | 1.047(0.939,1.167) | 0.438 |
| genus.Alloprevotella.id.961 | IBD | IVW | 1.041(0.928,1.168) | 0.491 |
| genus.Alloprevotella.id.961 | IBD | Weighted median | 1.087(0.94,1.257) | 0.259 |
| genus.Alloprevotella.id.961 | IBD | Weighted mode | 1.106(0.907,1.35) | 0.319 |
| genus.Alloprevotella.id.961 | IBD | MR-Robust | 1.098(0.992,1.216) | 0.071 |
| genus.Alloprevotella.id.961 | IBD | MR-Egger | 1.128(0.339,3.75) | 0.844 |
| genus.Alloprevotella.id.961 | IBD | MRRAPS | 1.061(0.936,1.202) | 0.357 |
| genus.Alloprevotella.id.961 | IBD | MRPRESSO | 1.041(0.929,1.167) | 0.519 |
| genus.Anaerofilum.id.2053 | IBD | IVW | 0.965(0.849,1.098) | 0.590 |
| genus.Anaerofilum.id.2053 | IBD | Weighted median | 0.966(0.829,1.125) | 0.654 |
| genus.Anaerofilum.id.2053 | IBD | Weighted mode | 0.966(0.757,1.232) | 0.780 |
| genus.Anaerofilum.id.2053 | IBD | MR-Robust | 0.974(0.862,1.101) | 0.676 |
| genus.Anaerofilum.id.2053 | IBD | MR-Egger | 1.01(0.502,2.034) | 0.977 |
| genus.Anaerofilum.id.2053 | IBD | MRRAPS | 0.973(0.857,1.103) | 0.666 |
| genus.Anaerofilum.id.2053 | IBD | MRPRESSO | 0.965(0.849,1.098) | 0.602 |
| genus.Anaerostipes.id.1991 | IBD | IVW | 1.107(0.92,1.332) | 0.281 |
| genus.Anaerostipes.id.1991 | IBD | Weighted median | 1.033(0.813,1.312) | 0.792 |
| genus.Anaerostipes.id.1991 | IBD | Weighted mode | 1.006(0.678,1.493) | 0.977 |
| genus.Anaerostipes.id.1991 | IBD | MR-Robust | 1.06(0.715,1.57) | 0.773 |
| genus.Anaerostipes.id.1991 | IBD | MR-Egger | 1.183(0.607,2.305) | 0.622 |
| genus.Anaerostipes.id.1991 | IBD | MRRAPS | 1.1(0.899,1.346) | 0.355 |
| genus.Anaerostipes.id.1991 | IBD | MRPRESSO | 1.107(0.975,1.257) | 0.143 |
| genus.Anaerotruncus.id.2054 | IBD | IVW | 0.834(0.675,1.031) | 0.094 |
| genus.Anaerotruncus.id.2054 | IBD | Weighted median | 0.809(0.621,1.055) | 0.118 |
| genus.Anaerotruncus.id.2054 | IBD | Weighted mode | 0.707(0.443,1.129) | 0.146 |
| genus.Anaerotruncus.id.2054 | IBD | MR-Robust | 0.827(0.661,1.035) | 0.098 |
| genus.Anaerotruncus.id.2054 | IBD | MR-Egger | 0.859(0.422,1.746) | 0.674 |
| genus.Anaerotruncus.id.2054 | IBD | MRRAPS | 0.822(0.647,1.044) | 0.108 |
| genus.Anaerotruncus.id.2054 | IBD | MRPRESSO | 0.834(0.675,1.031) | 0.120 |
| genus.Bacteroides.id.918 | IBD | IVW | 1.082(0.849,1.379) | 0.526 |
| genus.Bacteroides.id.918 | IBD | Weighted median | 1.15(0.836,1.583) | 0.391 |
| genus.Bacteroides.id.918 | IBD | Weighted mode | 1.177(0.685,2.022) | 0.555 |
| genus.Bacteroides.id.918 | IBD | MR-Robust | 1.085(0.764,1.541) | 0.649 |
| genus.Bacteroides.id.918 | IBD | MR-Egger | 1.628(0.398,6.666) | 0.498 |
| genus.Bacteroides.id.918 | IBD | MRRAPS | 1.074(0.827,1.396) | 0.592 |
| genus.Bacteroides.id.918 | IBD | MRPRESSO | 1.082(0.867,1.35) | 0.509 |
| genus.Barnesiella.id.944 | IBD | IVW | 1.113(0.943,1.314) | 0.204 |
| genus.Barnesiella.id.944 | IBD | Weighted median | 1.101(0.88,1.379) | 0.400 |
| genus.Barnesiella.id.944 | IBD | Weighted mode | 1.046(0.734,1.491) | 0.804 |
| genus.Barnesiella.id.944 | IBD | MR-Robust | 1.101(0.922,1.316) | 0.288 |
| genus.Barnesiella.id.944 | IBD | MR-Egger | 1.415(0.807,2.479) | 0.225 |
| genus.Barnesiella.id.944 | IBD | MRRAPS | 1.11(0.93,1.325) | 0.246 |
| genus.Barnesiella.id.944 | IBD | MRPRESSO | 1.113(0.943,1.314) | 0.226 |
| genus.Bifidobacterium.id.436 | IBD | IVW | 0.869(0.762,0.991) | 0.036 |
| genus.Bifidobacterium.id.436 | IBD | Weighted median | 0.884(0.744,1.05) | 0.160 |
| genus.Bifidobacterium.id.436 | IBD | Weighted mode | 0.909(0.703,1.175) | 0.466 |
| genus.Bifidobacterium.id.436 | IBD | MR-Robust | 0.861(0.764,0.969) | 0.013 |
| genus.Bifidobacterium.id.436 | IBD | MR-Egger | 0.775(0.512,1.171) | 0.225 |
| genus.Bifidobacterium.id.436 | IBD | MRRAPS | 0.845(0.74,0.967) | 0.014 |
| genus.Bifidobacterium.id.436 | IBD | MRPRESSO | 0.869(0.762,0.991) | 0.048 |
| genus.Bilophila.id.3170 | IBD | IVW | 1.175(0.997,1.383) | 0.054 |
| genus.Bilophila.id.3170 | IBD | Weighted median | 1.248(0.993,1.567) | 0.057 |
| genus.Bilophila.id.3170 | IBD | Weighted mode | 1.402(0.935,2.103) | 0.102 |
| genus.Bilophila.id.3170 | IBD | MR-Robust | 1.201(0.949,1.518) | 0.127 |
| genus.Bilophila.id.3170 | IBD | MR-Egger | 1.18(0.54,2.577) | 0.677 |
| genus.Bilophila.id.3170 | IBD | MRRAPS | 1.202(1.006,1.438) | 0.043 |
| genus.Bilophila.id.3170 | IBD | MRPRESSO | 1.175(0.998,1.383) | 0.077 |
| genus.Blautia.id.1992 | IBD | IVW | 1.108(0.933,1.317) | 0.243 |
| genus.Blautia.id.1992 | IBD | Weighted median | 1.133(0.896,1.434) | 0.296 |
| genus.Blautia.id.1992 | IBD | Weighted mode | 1.074(0.758,1.522) | 0.689 |
| genus.Blautia.id.1992 | IBD | MR-Robust | 1.111(0.956,1.293) | 0.171 |
| genus.Blautia.id.1992 | IBD | MR-Egger | 1.231(0.84,1.806) | 0.287 |
| genus.Blautia.id.1992 | IBD | MRRAPS | 1.115(0.923,1.347) | 0.257 |
| genus.Blautia.id.1992 | IBD | MRPRESSO | 1.108(0.968,1.269) | 0.162 |
| genus.Butyricicoccus.id.2055 | IBD | IVW | 1.056(0.754,1.478) | 0.752 |
| genus.Butyricicoccus.id.2055 | IBD | Weighted median | 0.92(0.711,1.19) | 0.524 |
| genus.Butyricicoccus.id.2055 | IBD | Weighted mode | 0.89(0.688,1.152) | 0.377 |
| genus.Butyricicoccus.id.2055 | IBD | MR-Robust | 1.007(0.744,1.362) | 0.964 |
| genus.Butyricicoccus.id.2055 | IBD | MR-Egger | 0.932(0.486,1.786) | 0.832 |
| genus.Butyricicoccus.id.2055 | IBD | MRRAPS | 0.975(0.784,1.213) | 0.821 |
| genus.Butyricicoccus.id.2055 | IBD | MRPRESSO | 1.056(0.754,1.478) | 0.761 |
| genus.Butyricimonas.id.945 | IBD | IVW | 0.996(0.853,1.162) | 0.956 |
| genus.Butyricimonas.id.945 | IBD | Weighted median | 1.108(0.909,1.35) | 0.310 |
| genus.Butyricimonas.id.945 | IBD | Weighted mode | 1.148(0.828,1.593) | 0.407 |
| genus.Butyricimonas.id.945 | IBD | MR-Robust | 1.133(0.724,1.774) | 0.584 |
| genus.Butyricimonas.id.945 | IBD | MR-Egger | 0.868(0.511,1.474) | 0.600 |
| genus.Butyricimonas.id.945 | IBD | MRRAPS | 1.028(0.883,1.198) | 0.718 |
| genus.Butyricimonas.id.945 | IBD | MRPRESSO | 0.996(0.853,1.162) | 0.957 |
| genus.Butyrivibrio.id.1993 | IBD | IVW | 0.94(0.867,1.019) | 0.132 |
| genus.Butyrivibrio.id.1993 | IBD | Weighted median | 0.927(0.83,1.034) | 0.173 |
| genus.Butyrivibrio.id.1993 | IBD | Weighted mode | 0.936(0.77,1.136) | 0.503 |
| genus.Butyrivibrio.id.1993 | IBD | MR-Robust | 0.939(0.861,1.024) | 0.157 |
| genus.Butyrivibrio.id.1993 | IBD | MR-Egger | 1.206(0.834,1.744) | 0.318 |
| genus.Butyrivibrio.id.1993 | IBD | MRRAPS | 0.935(0.859,1.019) | 0.124 |
| genus.Butyrivibrio.id.1993 | IBD | MRPRESSO | 0.94(0.867,1.019) | 0.154 |
| genus.CandidatusSoleaferrea.id.11350 | IBD | IVW | 0.957(0.839,1.091) | 0.509 |
| genus.CandidatusSoleaferrea.id.11350 | IBD | Weighted median | 0.898(0.754,1.07) | 0.230 |
| genus.CandidatusSoleaferrea.id.11350 | IBD | Weighted mode | 0.856(0.653,1.123) | 0.263 |
| genus.CandidatusSoleaferrea.id.11350 | IBD | MR-Robust | 0.945(0.828,1.079) | 0.404 |
| genus.CandidatusSoleaferrea.id.11350 | IBD | MR-Egger | 0.976(0.244,3.904) | 0.972 |
| genus.CandidatusSoleaferrea.id.11350 | IBD | MRRAPS | 0.942(0.817,1.086) | 0.411 |
| genus.CandidatusSoleaferrea.id.11350 | IBD | MRPRESSO | 0.957(0.846,1.082) | 0.500 |
| genus.Catenibacterium.id.2153 | IBD | IVW | 0.889(0.759,1.041) | 0.145 |
| genus.Catenibacterium.id.2153 | IBD | Weighted median | 0.824(0.688,0.986) | 0.035 |
| genus.Catenibacterium.id.2153 | IBD | Weighted mode | 0.799(0.605,1.055) | 0.113 |
| genus.Catenibacterium.id.2153 | IBD | MR-Robust | 0.886(0.755,1.039) | 0.137 |
| genus.Catenibacterium.id.2153 | IBD | MR-Egger | 2.441(0.719,8.292) | 0.153 |
| genus.Catenibacterium.id.2153 | IBD | MRRAPS | 0.893(0.751,1.061) | 0.198 |
| genus.Catenibacterium.id.2153 | IBD | MRPRESSO | 0.889(0.759,1.041) | 0.218 |
| genus.ChristensenellaceaeR.7group.id.11283 | IBD | IVW | 1.016(0.82,1.258) | 0.885 |
| genus.ChristensenellaceaeR.7group.id.11283 | IBD | Weighted median | 1.114(0.835,1.488) | 0.462 |
| genus.ChristensenellaceaeR.7group.id.11283 | IBD | Weighted mode | 1.165(0.748,1.816) | 0.499 |
| genus.ChristensenellaceaeR.7group.id.11283 | IBD | MR-Robust | 1.039(0.839,1.285) | 0.727 |
| genus.ChristensenellaceaeR.7group.id.11283 | IBD | MR-Egger | 1.049(0.563,1.955) | 0.879 |
| genus.ChristensenellaceaeR.7group.id.11283 | IBD | MRRAPS | 1.044(0.828,1.316) | 0.716 |
| genus.ChristensenellaceaeR.7group.id.11283 | IBD | MRPRESSO | 1.016(0.832,1.24) | 0.880 |
| genus.Clostridiumsensustricto1.id.1873 | IBD | IVW | 0.765(0.62,0.942) | 0.012 |
| genus.Clostridiumsensustricto1.id.1873 | IBD | Weighted median | 0.815(0.627,1.061) | 0.129 |
| genus.Clostridiumsensustricto1.id.1873 | IBD | Weighted mode | 0.876(0.642,1.196) | 0.404 |
| genus.Clostridiumsensustricto1.id.1873 | IBD | MR-Robust | 0.775(0.639,0.941) | 0.010 |
| genus.Clostridiumsensustricto1.id.1873 | IBD | MR-Egger | 0.968(0.617,1.517) | 0.886 |
| genus.Clostridiumsensustricto1.id.1873 | IBD | MRRAPS | 0.772(0.622,0.959) | 0.019 |
| genus.Clostridiumsensustricto1.id.1873 | IBD | MRPRESSO | 0.765(0.62,0.942) | 0.046 |
| genus.Collinsella.id.815 | IBD | IVW | 0.978(0.806,1.186) | 0.820 |
| genus.Collinsella.id.815 | IBD | Weighted median | 1.032(0.804,1.324) | 0.806 |
| genus.Collinsella.id.815 | IBD | Weighted mode | 1.058(0.722,1.549) | 0.773 |
| genus.Collinsella.id.815 | IBD | MR-Robust | 1.011(0.722,1.416) | 0.948 |
| genus.Collinsella.id.815 | IBD | MR-Egger | 0.878(0.414,1.862) | 0.734 |
| genus.Collinsella.id.815 | IBD | MRRAPS | 0.981(0.795,1.211) | 0.861 |
| genus.Collinsella.id.815 | IBD | MRPRESSO | 0.978(0.835,1.146) | 0.788 |
| genus.Coprobacter.id.949 | IBD | IVW | 0.94(0.828,1.067) | 0.335 |
| genus.Coprobacter.id.949 | IBD | Weighted median | 0.934(0.787,1.108) | 0.432 |
| genus.Coprobacter.id.949 | IBD | Weighted mode | 0.944(0.734,1.213) | 0.652 |
| genus.Coprobacter.id.949 | IBD | MR-Robust | 0.937(0.832,1.055) | 0.285 |
| genus.Coprobacter.id.949 | IBD | MR-Egger | 1.077(0.691,1.677) | 0.744 |
| genus.Coprobacter.id.949 | IBD | MRRAPS | 0.931(0.814,1.064) | 0.295 |
| genus.Coprobacter.id.949 | IBD | MRPRESSO | 0.94(0.828,1.067) | 0.358 |
| genus.Coprococcus1.id.11301 | IBD | IVW | 1.021(0.852,1.224) | 0.821 |
| genus.Coprococcus1.id.11301 | IBD | Weighted median | 0.956(0.756,1.21) | 0.707 |
| genus.Coprococcus1.id.11301 | IBD | Weighted mode | 0.961(0.713,1.295) | 0.794 |
| genus.Coprococcus1.id.11301 | IBD | MR-Robust | 1.017(0.876,1.181) | 0.827 |
| genus.Coprococcus1.id.11301 | IBD | MR-Egger | 0.993(0.63,1.566) | 0.977 |
| genus.Coprococcus1.id.11301 | IBD | MRRAPS | 1.019(0.846,1.227) | 0.846 |
| genus.Coprococcus1.id.11301 | IBD | MRPRESSO | 1.021(0.852,1.224) | 0.825 |
| genus.Coprococcus2.id.11302 | IBD | IVW | 1.244(1.042,1.485) | 0.016 |
| genus.Coprococcus2.id.11302 | IBD | Weighted median | 1.335(1.058,1.684) | 0.015 |
| genus.Coprococcus2.id.11302 | IBD | Weighted mode | 1.359(0.922,2.005) | 0.122 |
| genus.Coprococcus2.id.11302 | IBD | MR-Robust | 1.243(1.076,1.435) | 0.003 |
| genus.Coprococcus2.id.11302 | IBD | MR-Egger | 1.179(0.394,3.529) | 0.769 |
| genus.Coprococcus2.id.11302 | IBD | MRRAPS | 1.251(1.028,1.522) | 0.025 |
| genus.Coprococcus2.id.11302 | IBD | MRPRESSO | 1.244(1.08,1.432) | 0.014 |
| genus.Coprococcus3.id.11303 | IBD | IVW | 1.107(0.864,1.418) | 0.423 |
| genus.Coprococcus3.id.11303 | IBD | Weighted median | 1.112(0.836,1.48) | 0.464 |
| genus.Coprococcus3.id.11303 | IBD | Weighted mode | 1.102(0.676,1.796) | 0.696 |
| genus.Coprococcus3.id.11303 | IBD | MR-Robust | 1.1(0.854,1.418) | 0.460 |
| genus.Coprococcus3.id.11303 | IBD | MR-Egger | 2.123(0.778,5.793) | 0.141 |
| genus.Coprococcus3.id.11303 | IBD | MRRAPS | 1.121(0.796,1.58) | 0.513 |
| genus.Coprococcus3.id.11303 | IBD | MRPRESSO | 1.107(0.864,1.418) | 0.441 |
| genus.DefluviitaleaceaeUCG011.id.11287 | IBD | IVW | 1.088(0.941,1.259) | 0.254 |
| genus.DefluviitaleaceaeUCG011.id.11287 | IBD | Weighted median | 1.06(0.879,1.277) | 0.542 |
| genus.DefluviitaleaceaeUCG011.id.11287 | IBD | Weighted mode | 1.04(0.784,1.379) | 0.785 |
| genus.DefluviitaleaceaeUCG011.id.11287 | IBD | MR-Robust | 1.037(0.893,1.204) | 0.638 |
| genus.DefluviitaleaceaeUCG011.id.11287 | IBD | MR-Egger | 1.266(0.752,2.13) | 0.375 |
| genus.DefluviitaleaceaeUCG011.id.11287 | IBD | MRRAPS | 1.073(0.916,1.257) | 0.382 |
| genus.DefluviitaleaceaeUCG011.id.11287 | IBD | MRPRESSO | 1.088(0.968,1.224) | 0.190 |
| genus.Desulfovibrio.id.3173 | IBD | IVW | 1.015(0.878,1.173) | 0.838 |
| genus.Desulfovibrio.id.3173 | IBD | Weighted median | 1.058(0.874,1.281) | 0.562 |
| genus.Desulfovibrio.id.3173 | IBD | Weighted mode | 1.149(0.853,1.548) | 0.360 |
| genus.Desulfovibrio.id.3173 | IBD | MR-Robust | 1.018(0.906,1.144) | 0.766 |
| genus.Desulfovibrio.id.3173 | IBD | MR-Egger | 1.14(0.748,1.739) | 0.542 |
| genus.Desulfovibrio.id.3173 | IBD | MRRAPS | 1.016(0.867,1.19) | 0.848 |
| genus.Desulfovibrio.id.3173 | IBD | MRPRESSO | 1.015(0.914,1.128) | 0.784 |
| genus.Dialister.id.2183 | IBD | IVW | 0.976(0.812,1.173) | 0.794 |
| genus.Dialister.id.2183 | IBD | Weighted median | 1.075(0.846,1.367) | 0.554 |
| genus.Dialister.id.2183 | IBD | Weighted mode | 1.112(0.757,1.632) | 0.588 |
| genus.Dialister.id.2183 | IBD | MR-Robust | 0.987(0.816,1.195) | 0.896 |
| genus.Dialister.id.2183 | IBD | MR-Egger | 1.32(0.579,3.007) | 0.509 |
| genus.Dialister.id.2183 | IBD | MRRAPS | 0.995(0.823,1.203) | 0.958 |
| genus.Dialister.id.2183 | IBD | MRPRESSO | 0.976(0.812,1.173) | 0.799 |
| genus.Dorea.id.1997 | IBD | IVW | 1.022(0.844,1.237) | 0.827 |
| genus.Dorea.id.1997 | IBD | Weighted median | 1.108(0.859,1.431) | 0.430 |
| genus.Dorea.id.1997 | IBD | Weighted mode | 1.218(0.8,1.855) | 0.358 |
| genus.Dorea.id.1997 | IBD | MR-Robust | 1.024(0.872,1.202) | 0.772 |
| genus.Dorea.id.1997 | IBD | MR-Egger | 1.1(0.669,1.809) | 0.706 |
| genus.Dorea.id.1997 | IBD | MRRAPS | 1.022(0.83,1.259) | 0.837 |
| genus.Dorea.id.1997 | IBD | MRPRESSO | 1.022(0.876,1.191) | 0.791 |
| genus.Eggerthella.id.819 | IBD | IVW | 0.975(0.864,1.1) | 0.680 |
| genus.Eggerthella.id.819 | IBD | Weighted median | 0.93(0.793,1.091) | 0.375 |
| genus.Eggerthella.id.819 | IBD | Weighted mode | 0.922(0.711,1.196) | 0.541 |
| genus.Eggerthella.id.819 | IBD | MR-Robust | 0.968(0.854,1.097) | 0.609 |
| genus.Eggerthella.id.819 | IBD | MR-Egger | 1.088(0.608,1.946) | 0.776 |
| genus.Eggerthella.id.819 | IBD | MRRAPS | 0.966(0.847,1.102) | 0.607 |
| genus.Eggerthella.id.819 | IBD | MRPRESSO | 0.975(0.876,1.085) | 0.652 |
| genus.Eisenbergiella.id.11304 | IBD | IVW | 1.017(0.873,1.186) | 0.827 |
| genus.Eisenbergiella.id.11304 | IBD | Weighted median | 1.054(0.888,1.252) | 0.549 |
| genus.Eisenbergiella.id.11304 | IBD | Weighted mode | 1.065(0.799,1.419) | 0.666 |
| genus.Eisenbergiella.id.11304 | IBD | MR-Robust | 1.023(0.869,1.204) | 0.783 |
| genus.Eisenbergiella.id.11304 | IBD | MR-Egger | 1.674(0.492,5.694) | 0.410 |
| genus.Eisenbergiella.id.11304 | IBD | MRRAPS | 1.028(0.883,1.196) | 0.721 |
| genus.Eisenbergiella.id.11304 | IBD | MRPRESSO | 1.017(0.873,1.186) | 0.831 |
| genus.Enterorhabdus.id.820 | IBD | IVW | 0.922(0.778,1.093) | 0.351 |
| genus.Enterorhabdus.id.820 | IBD | Weighted median | 0.971(0.777,1.213) | 0.794 |
| genus.Enterorhabdus.id.820 | IBD | Weighted mode | 1.017(0.774,1.337) | 0.901 |
| genus.Enterorhabdus.id.820 | IBD | MR-Robust | 0.932(0.783,1.11) | 0.429 |
| genus.Enterorhabdus.id.820 | IBD | MR-Egger | 1.166(0.753,1.804) | 0.491 |
| genus.Enterorhabdus.id.820 | IBD | MRRAPS | 0.934(0.782,1.117) | 0.455 |
| genus.Enterorhabdus.id.820 | IBD | MRPRESSO | 0.922(0.778,1.093) | 0.394 |
| genus.Erysipelatoclostridium.id.11381 | IBD | IVW | 0.999(0.867,1.15) | 0.985 |
| genus.Erysipelatoclostridium.id.11381 | IBD | Weighted median | 1.034(0.867,1.234) | 0.711 |
| genus.Erysipelatoclostridium.id.11381 | IBD | Weighted mode | 0.992(0.743,1.325) | 0.959 |
| genus.Erysipelatoclostridium.id.11381 | IBD | MR-Robust | 1.05(0.918,1.202) | 0.474 |
| genus.Erysipelatoclostridium.id.11381 | IBD | MR-Egger | 0.645(0.374,1.111) | 0.114 |
| genus.Erysipelatoclostridium.id.11381 | IBD | MRRAPS | 1.036(0.905,1.185) | 0.612 |
| genus.Erysipelatoclostridium.id.11381 | IBD | MRPRESSO | 0.999(0.867,1.15) | 0.985 |
| genus.ErysipelotrichaceaeUCG003.id.11384 | IBD | IVW | 1.058(0.918,1.219) | 0.438 |
| genus.ErysipelotrichaceaeUCG003.id.11384 | IBD | Weighted median | 1.058(0.87,1.288) | 0.572 |
| genus.ErysipelotrichaceaeUCG003.id.11384 | IBD | Weighted mode | 1.009(0.726,1.403) | 0.957 |
| genus.ErysipelotrichaceaeUCG003.id.11384 | IBD | MR-Robust | 1.047(0.912,1.202) | 0.513 |
| genus.ErysipelotrichaceaeUCG003.id.11384 | IBD | MR-Egger | 1.048(0.71,1.547) | 0.814 |
| genus.ErysipelotrichaceaeUCG003.id.11384 | IBD | MRRAPS | 1.045(0.897,1.219) | 0.572 |
| genus.ErysipelotrichaceaeUCG003.id.11384 | IBD | MRPRESSO | 1.058(0.924,1.211) | 0.429 |
| genus.Escherichia.Shigella.id.3504 | IBD | IVW | 1.082(0.902,1.298) | 0.396 |
| genus.Escherichia.Shigella.id.3504 | IBD | Weighted median | 1.037(0.823,1.307) | 0.757 |
| genus.Escherichia.Shigella.id.3504 | IBD | Weighted mode | 1.028(0.707,1.493) | 0.886 |
| genus.Escherichia.Shigella.id.3504 | IBD | MR-Robust | 1.077(0.821,1.413) | 0.594 |
| genus.Escherichia.Shigella.id.3504 | IBD | MR-Egger | 1.223(0.695,2.152) | 0.485 |
| genus.Escherichia.Shigella.id.3504 | IBD | MRRAPS | 1.084(0.888,1.323) | 0.429 |
| genus.Escherichia.Shigella.id.3504 | IBD | MRPRESSO | 1.082(0.953,1.228) | 0.255 |
| genus.Faecalibacterium.id.2057 | IBD | IVW | 1.103(0.925,1.316) | 0.275 |
| genus.Faecalibacterium.id.2057 | IBD | Weighted median | 0.967(0.753,1.244) | 0.796 |
| genus.Faecalibacterium.id.2057 | IBD | Weighted mode | 0.963(0.672,1.381) | 0.839 |
| genus.Faecalibacterium.id.2057 | IBD | MR-Robust | 1.1(0.904,1.34) | 0.340 |
| genus.Faecalibacterium.id.2057 | IBD | MR-Egger | 1.117(0.79,1.58) | 0.531 |
| genus.Faecalibacterium.id.2057 | IBD | MRRAPS | 1.111(0.883,1.397) | 0.368 |
| genus.Faecalibacterium.id.2057 | IBD | MRPRESSO | 1.103(0.935,1.301) | 0.274 |
| genus.FamilyXIIIAD3011group.id.11293 | IBD | IVW | 1.005(0.826,1.224) | 0.958 |
| genus.FamilyXIIIAD3011group.id.11293 | IBD | Weighted median | 0.903(0.705,1.156) | 0.418 |
| genus.FamilyXIIIAD3011group.id.11293 | IBD | Weighted mode | 0.835(0.559,1.248) | 0.379 |
| genus.FamilyXIIIAD3011group.id.11293 | IBD | MR-Robust | 0.983(0.768,1.258) | 0.890 |
| genus.FamilyXIIIAD3011group.id.11293 | IBD | MR-Egger | 1.025(0.401,2.624) | 0.959 |
| genus.FamilyXIIIAD3011group.id.11293 | IBD | MRRAPS | 0.988(0.806,1.211) | 0.907 |
| genus.FamilyXIIIAD3011group.id.11293 | IBD | MRPRESSO | 1.005(0.826,1.224) | 0.959 |
| genus.FamilyXIIIUCG001.id.11294 | IBD | IVW | 1.2(0.94,1.532) | 0.143 |
| genus.FamilyXIIIUCG001.id.11294 | IBD | Weighted median | 1.203(0.908,1.595) | 0.198 |
| genus.FamilyXIIIUCG001.id.11294 | IBD | Weighted mode | 1.198(0.809,1.774) | 0.368 |
| genus.FamilyXIIIUCG001.id.11294 | IBD | MR-Robust | 1.204(0.962,1.508) | 0.105 |
| genus.FamilyXIIIUCG001.id.11294 | IBD | MR-Egger | 1.421(0.697,2.897) | 0.333 |
| genus.FamilyXIIIUCG001.id.11294 | IBD | MRRAPS | 1.23(0.979,1.544) | 0.075 |
| genus.FamilyXIIIUCG001.id.11294 | IBD | MRPRESSO | 1.2(0.94,1.532) | 0.186 |
| genus.Flavonifractor.id.2059 | IBD | IVW | 0.944(0.727,1.225) | 0.665 |
| genus.Flavonifractor.id.2059 | IBD | Weighted median | 0.961(0.721,1.28) | 0.784 |
| genus.Flavonifractor.id.2059 | IBD | Weighted mode | 1.004(0.68,1.481) | 0.985 |
| genus.Flavonifractor.id.2059 | IBD | MR-Robust | 0.945(0.751,1.19) | 0.632 |
| genus.Flavonifractor.id.2059 | IBD | MR-Egger | 2.623(0.978,7.034) | 0.055 |
| genus.Flavonifractor.id.2059 | IBD | MRRAPS | 0.957(0.745,1.229) | 0.729 |
| genus.Flavonifractor.id.2059 | IBD | MRPRESSO | 0.944(0.727,1.225) | 0.683 |
| genus.Fusicatenibacter.id.11305 | IBD | IVW | 0.946(0.807,1.109) | 0.495 |
| genus.Fusicatenibacter.id.11305 | IBD | Weighted median | 0.916(0.737,1.137) | 0.425 |
| genus.Fusicatenibacter.id.11305 | IBD | Weighted mode | 0.887(0.612,1.286) | 0.528 |
| genus.Fusicatenibacter.id.11305 | IBD | MR-Robust | 0.96(0.825,1.118) | 0.602 |
| genus.Fusicatenibacter.id.11305 | IBD | MR-Egger | 0.943(0.497,1.792) | 0.859 |
| genus.Fusicatenibacter.id.11305 | IBD | MRRAPS | 0.962(0.815,1.136) | 0.647 |
| genus.Fusicatenibacter.id.11305 | IBD | MRPRESSO | 0.946(0.807,1.109) | 0.503 |
| genus.Gordonibacter.id.821 | IBD | IVW | 1.025(0.92,1.143) | 0.652 |
| genus.Gordonibacter.id.821 | IBD | Weighted median | 1.003(0.881,1.141) | 0.969 |
| genus.Gordonibacter.id.821 | IBD | Weighted mode | 0.982(0.79,1.221) | 0.873 |
| genus.Gordonibacter.id.821 | IBD | MR-Robust | 1.023(0.916,1.143) | 0.688 |
| genus.Gordonibacter.id.821 | IBD | MR-Egger | 0.97(0.594,1.584) | 0.902 |
| genus.Gordonibacter.id.821 | IBD | MRRAPS | 1.024(0.905,1.158) | 0.708 |
| genus.Gordonibacter.id.821 | IBD | MRPRESSO | 1.025(0.92,1.143) | 0.660 |
| genus.Haemophilus.id.3698 | IBD | IVW | 1.048(0.892,1.233) | 0.567 |
| genus.Haemophilus.id.3698 | IBD | Weighted median | 0.919(0.747,1.132) | 0.428 |
| genus.Haemophilus.id.3698 | IBD | Weighted mode | 0.877(0.602,1.277) | 0.494 |
| genus.Haemophilus.id.3698 | IBD | MR-Robust | 1.041(0.853,1.27) | 0.693 |
| genus.Haemophilus.id.3698 | IBD | MR-Egger | 0.987(0.662,1.474) | 0.950 |
| genus.Haemophilus.id.3698 | IBD | MRRAPS | 1.02(0.826,1.261) | 0.853 |
| genus.Haemophilus.id.3698 | IBD | MRPRESSO | 1.048(0.892,1.233) | 0.583 |
| genus.Holdemanella.id.11393 | IBD | IVW | 0.887(0.762,1.031) | 0.119 |
| genus.Holdemanella.id.11393 | IBD | Weighted median | 0.933(0.775,1.124) | 0.466 |
| genus.Holdemanella.id.11393 | IBD | Weighted mode | 1.001(0.736,1.361) | 0.994 |
| genus.Holdemanella.id.11393 | IBD | MR-Robust | 0.886(0.766,1.026) | 0.107 |
| genus.Holdemanella.id.11393 | IBD | MR-Egger | 0.989(0.616,1.587) | 0.964 |
| genus.Holdemanella.id.11393 | IBD | MRRAPS | 0.891(0.758,1.048) | 0.163 |
| genus.Holdemanella.id.11393 | IBD | MRPRESSO | 0.887(0.762,1.031) | 0.150 |
| genus.Holdemania.id.2157 | IBD | IVW | 0.961(0.845,1.092) | 0.541 |
| genus.Holdemania.id.2157 | IBD | Weighted median | 0.931(0.781,1.109) | 0.421 |
| genus.Holdemania.id.2157 | IBD | Weighted mode | 0.854(0.597,1.223) | 0.389 |
| genus.Holdemania.id.2157 | IBD | MR-Robust | 0.954(0.817,1.114) | 0.552 |
| genus.Holdemania.id.2157 | IBD | MR-Egger | 1.278(0.899,1.815) | 0.171 |
| genus.Holdemania.id.2157 | IBD | MRRAPS | 0.972(0.825,1.146) | 0.738 |
| genus.Holdemania.id.2157 | IBD | MRPRESSO | 0.961(0.845,1.092) | 0.551 |
| genus.Howardella.id.2000 | IBD | IVW | 0.961(0.869,1.064) | 0.445 |
| genus.Howardella.id.2000 | IBD | Weighted median | 0.95(0.837,1.078) | 0.425 |
| genus.Howardella.id.2000 | IBD | Weighted mode | 0.959(0.789,1.167) | 0.677 |
| genus.Howardella.id.2000 | IBD | MR-Robust | 0.949(0.859,1.049) | 0.305 |
| genus.Howardella.id.2000 | IBD | MR-Egger | 0.874(0.557,1.373) | 0.560 |
| genus.Howardella.id.2000 | IBD | MRRAPS | 0.961(0.86,1.074) | 0.481 |
| genus.Howardella.id.2000 | IBD | MRPRESSO | 0.961(0.909,1.016) | 0.197 |
| genus.Hungatella.id.11306 | IBD | IVW | 0.937(0.804,1.092) | 0.403 |
| genus.Hungatella.id.11306 | IBD | Weighted median | 0.898(0.737,1.094) | 0.286 |
| genus.Hungatella.id.11306 | IBD | Weighted mode | 0.853(0.646,1.126) | 0.261 |
| genus.Hungatella.id.11306 | IBD | MR-Robust | 0.934(0.814,1.071) | 0.326 |
| genus.Hungatella.id.11306 | IBD | MR-Egger | 1.198(0.475,3.018) | 0.702 |
| genus.Hungatella.id.11306 | IBD | MRRAPS | 0.935(0.791,1.107) | 0.436 |
| genus.Hungatella.id.11306 | IBD | MRPRESSO | 0.937(0.832,1.054) | 0.340 |
| genus.Intestinibacter.id.11345 | IBD | IVW | 0.978(0.85,1.125) | 0.753 |
| genus.Intestinibacter.id.11345 | IBD | Weighted median | 1.018(0.844,1.229) | 0.849 |
| genus.Intestinibacter.id.11345 | IBD | Weighted mode | 1.056(0.783,1.424) | 0.721 |
| genus.Intestinibacter.id.11345 | IBD | MR-Robust | 1.012(0.889,1.153) | 0.852 |
| genus.Intestinibacter.id.11345 | IBD | MR-Egger | 0.666(0.42,1.054) | 0.083 |
| genus.Intestinibacter.id.11345 | IBD | MRRAPS | 1.001(0.859,1.165) | 0.995 |
| genus.Intestinibacter.id.11345 | IBD | MRPRESSO | 0.978(0.866,1.104) | 0.722 |
| genus.Intestinimonas.id.2062 | IBD | IVW | 0.947(0.832,1.077) | 0.404 |
| genus.Intestinimonas.id.2062 | IBD | Weighted median | 0.929(0.782,1.104) | 0.403 |
| genus.Intestinimonas.id.2062 | IBD | Weighted mode | 0.901(0.672,1.207) | 0.484 |
| genus.Intestinimonas.id.2062 | IBD | MR-Robust | 0.954(0.855,1.064) | 0.395 |
| genus.Intestinimonas.id.2062 | IBD | MR-Egger | 0.973(0.698,1.355) | 0.871 |
| genus.Intestinimonas.id.2062 | IBD | MRRAPS | 0.954(0.829,1.097) | 0.507 |
| genus.Intestinimonas.id.2062 | IBD | MRPRESSO | 0.947(0.851,1.053) | 0.329 |
| genus.Lachnoclostridium.id.11308 | IBD | IVW | 0.913(0.707,1.181) | 0.489 |
| genus.Lachnoclostridium.id.11308 | IBD | Weighted median | 0.974(0.732,1.296) | 0.856 |
| genus.Lachnoclostridium.id.11308 | IBD | Weighted mode | 1.176(0.721,1.916) | 0.516 |
| genus.Lachnoclostridium.id.11308 | IBD | MR-Robust | 0.926(0.704,1.217) | 0.581 |
| genus.Lachnoclostridium.id.11308 | IBD | MR-Egger | 0.373(0.168,0.829) | 0.016 |
| genus.Lachnoclostridium.id.11308 | IBD | MRRAPS | 0.872(0.65,1.17) | 0.360 |
| genus.Lachnoclostridium.id.11308 | IBD | MRPRESSO | 0.913(0.707,1.181) | 0.502 |
| genus.Lachnospira.id.2004 | IBD | IVW | 0.866(0.598,1.255) | 0.447 |
| genus.Lachnospira.id.2004 | IBD | Weighted median | 0.923(0.646,1.319) | 0.660 |
| genus.Lachnospira.id.2004 | IBD | Weighted mode | 0.973(0.612,1.548) | 0.909 |
| genus.Lachnospira.id.2004 | IBD | MR-Robust | 0.909(0.636,1.3) | 0.603 |
| genus.Lachnospira.id.2004 | IBD | MR-Egger | 0.314(0.046,2.131) | 0.236 |
| genus.Lachnospira.id.2004 | IBD | MRRAPS | 0.901(0.64,1.268) | 0.549 |
| genus.Lachnospira.id.2004 | IBD | MRPRESSO | 0.866(0.598,1.255) | 0.476 |
| genus.LachnospiraceaeFCS020group.id.11314 | IBD | IVW | 1.263(1.07,1.49) | 0.006 |
| genus.LachnospiraceaeFCS020group.id.11314 | IBD | Weighted median | 1.226(0.983,1.53) | 0.071 |
| genus.LachnospiraceaeFCS020group.id.11314 | IBD | Weighted mode | 1.173(0.846,1.626) | 0.338 |
| genus.LachnospiraceaeFCS020group.id.11314 | IBD | MR-Robust | 1.254(1.064,1.478) | 0.007 |
| genus.LachnospiraceaeFCS020group.id.11314 | IBD | MR-Egger | 1.076(0.698,1.661) | 0.739 |
| genus.LachnospiraceaeFCS020group.id.11314 | IBD | MRRAPS | 1.26(1.065,1.491) | 0.007 |
| genus.LachnospiraceaeFCS020group.id.11314 | IBD | MRPRESSO | 1.263(1.07,1.49) | 0.017 |
| genus.LachnospiraceaeNC2004group.id.11316 | IBD | IVW | 1.058(0.914,1.223) | 0.451 |
| genus.LachnospiraceaeNC2004group.id.11316 | IBD | Weighted median | 1.097(0.911,1.322) | 0.328 |
| genus.LachnospiraceaeNC2004group.id.11316 | IBD | Weighted mode | 1.118(0.83,1.505) | 0.462 |
| genus.LachnospiraceaeNC2004group.id.11316 | IBD | MR-Robust | 1.066(0.918,1.238) | 0.403 |
| genus.LachnospiraceaeNC2004group.id.11316 | IBD | MR-Egger | 0.791(0.424,1.476) | 0.461 |
| genus.LachnospiraceaeNC2004group.id.11316 | IBD | MRRAPS | 1.074(0.918,1.256) | 0.374 |
| genus.LachnospiraceaeNC2004group.id.11316 | IBD | MRPRESSO | 1.058(0.914,1.223) | 0.472 |
| genus.LachnospiraceaeND3007group.id.11317 | IBD | IVW | 1.706(1.131,2.574) | 0.011 |
| genus.LachnospiraceaeND3007group.id.11317 | IBD | Weighted median | 1.495(0.852,2.625) | 0.161 |
| genus.LachnospiraceaeND3007group.id.11317 | IBD | Weighted mode | 1.388(0.736,2.618) | 0.311 |
| genus.LachnospiraceaeND3007group.id.11317 | IBD | MR-Robust | 1.694(1.146,2.504) | 0.008 |
| genus.LachnospiraceaeND3007group.id.11317 | IBD | MR-Egger | 252.62(0.248,257068.547) | 0.117 |
| genus.LachnospiraceaeND3007group.id.11317 | IBD | MRRAPS | 1.74(1.085,2.79) | 0.022 |
| genus.LachnospiraceaeNK4A136group.id.11319 | IBD | IVW | 0.964(0.83,1.121) | 0.638 |
| genus.LachnospiraceaeNK4A136group.id.11319 | IBD | Weighted median | 1.018(0.824,1.257) | 0.869 |
| genus.LachnospiraceaeNK4A136group.id.11319 | IBD | Weighted mode | 1.039(0.795,1.356) | 0.781 |
| genus.LachnospiraceaeNK4A136group.id.11319 | IBD | MR-Robust | 0.969(0.849,1.105) | 0.635 |
| genus.LachnospiraceaeNK4A136group.id.11319 | IBD | MR-Egger | 1.128(0.825,1.542) | 0.450 |
| genus.LachnospiraceaeNK4A136group.id.11319 | IBD | MRRAPS | 0.964(0.817,1.136) | 0.660 |
| genus.LachnospiraceaeNK4A136group.id.11319 | IBD | MRPRESSO | 0.964(0.868,1.071) | 0.510 |
| genus.LachnospiraceaeUCG001.id.11321 | IBD | IVW | 0.915(0.774,1.082) | 0.298 |
| genus.LachnospiraceaeUCG001.id.11321 | IBD | Weighted median | 0.966(0.785,1.189) | 0.744 |
| genus.LachnospiraceaeUCG001.id.11321 | IBD | Weighted mode | 1.038(0.758,1.42) | 0.818 |
| genus.LachnospiraceaeUCG001.id.11321 | IBD | MR-Robust | 0.935(0.775,1.129) | 0.486 |
| genus.LachnospiraceaeUCG001.id.11321 | IBD | MR-Egger | 1.163(0.506,2.673) | 0.722 |
| genus.LachnospiraceaeUCG001.id.11321 | IBD | MRRAPS | 0.934(0.784,1.112) | 0.441 |
| genus.LachnospiraceaeUCG001.id.11321 | IBD | MRPRESSO | 0.915(0.774,1.082) | 0.320 |
| genus.LachnospiraceaeUCG004.id.11324 | IBD | IVW | 0.932(0.792,1.096) | 0.395 |
| genus.LachnospiraceaeUCG004.id.11324 | IBD | Weighted median | 0.935(0.757,1.154) | 0.531 |
| genus.LachnospiraceaeUCG004.id.11324 | IBD | Weighted mode | 0.941(0.669,1.322) | 0.724 |
| genus.LachnospiraceaeUCG004.id.11324 | IBD | MR-Robust | 0.969(0.842,1.115) | 0.660 |
| genus.LachnospiraceaeUCG004.id.11324 | IBD | MR-Egger | 1.094(0.571,2.095) | 0.786 |
| genus.LachnospiraceaeUCG004.id.11324 | IBD | MRRAPS | 0.949(0.795,1.134) | 0.567 |
| genus.LachnospiraceaeUCG004.id.11324 | IBD | MRPRESSO | 0.932(0.819,1.06) | 0.302 |
| genus.LachnospiraceaeUCG008.id.11328 | IBD | IVW | 1.099(0.982,1.231) | 0.101 |
| genus.LachnospiraceaeUCG008.id.11328 | IBD | Weighted median | 1.085(0.931,1.266) | 0.296 |
| genus.LachnospiraceaeUCG008.id.11328 | IBD | Weighted mode | 1.087(0.826,1.432) | 0.551 |
| genus.LachnospiraceaeUCG008.id.11328 | IBD | MR-Robust | 1.103(0.992,1.226) | 0.070 |
| genus.LachnospiraceaeUCG008.id.11328 | IBD | MR-Egger | 1.187(0.653,2.158) | 0.574 |
| genus.LachnospiraceaeUCG008.id.11328 | IBD | MRRAPS | 1.109(0.981,1.255) | 0.099 |
| genus.LachnospiraceaeUCG008.id.11328 | IBD | MRPRESSO | 1.099(0.994,1.216) | 0.089 |
| genus.LachnospiraceaeUCG010.id.11330 | IBD | IVW | 1.221(0.972,1.533) | 0.086 |
| genus.LachnospiraceaeUCG010.id.11330 | IBD | Weighted median | 1.357(1.06,1.736) | 0.015 |
| genus.LachnospiraceaeUCG010.id.11330 | IBD | Weighted mode | 1.381(1.009,1.89) | 0.044 |
| genus.LachnospiraceaeUCG010.id.11330 | IBD | MR-Robust | 1.291(0.961,1.736) | 0.090 |
| genus.LachnospiraceaeUCG010.id.11330 | IBD | MR-Egger | 1.407(0.759,2.609) | 0.279 |
| genus.LachnospiraceaeUCG010.id.11330 | IBD | MRRAPS | 1.287(1.035,1.601) | 0.023 |
| genus.LachnospiraceaeUCG010.id.11330 | IBD | MRPRESSO | 1.221(0.972,1.533) | 0.120 |
| genus.Lactobacillus.id.1837 | IBD | IVW | 0.862(0.743,1.001) | 0.051 |
| genus.Lactobacillus.id.1837 | IBD | Weighted median | 0.865(0.72,1.039) | 0.121 |
| genus.Lactobacillus.id.1837 | IBD | Weighted mode | 0.878(0.644,1.197) | 0.411 |
| genus.Lactobacillus.id.1837 | IBD | MR-Robust | 0.859(0.742,0.994) | 0.042 |
| genus.Lactobacillus.id.1837 | IBD | MR-Egger | 0.935(0.576,1.515) | 0.784 |
| genus.Lactobacillus.id.1837 | IBD | MRRAPS | 0.847(0.711,1.01) | 0.064 |
| genus.Lactobacillus.id.1837 | IBD | MRPRESSO | 0.862(0.743,1.001) | 0.083 |
| genus.Lactococcus.id.1851 | IBD | IVW | 1.013(0.891,1.152) | 0.839 |
| genus.Lactococcus.id.1851 | IBD | Weighted median | 0.996(0.855,1.161) | 0.960 |
| genus.Lactococcus.id.1851 | IBD | Weighted mode | 0.977(0.765,1.248) | 0.853 |
| genus.Lactococcus.id.1851 | IBD | MR-Robust | 1.014(0.893,1.151) | 0.834 |
| genus.Lactococcus.id.1851 | IBD | MR-Egger | 1.101(0.603,2.011) | 0.753 |
| genus.Lactococcus.id.1851 | IBD | MRRAPS | 1.017(0.889,1.163) | 0.811 |
| genus.Lactococcus.id.1851 | IBD | MRPRESSO | 1.013(0.891,1.152) | 0.844 |
| genus.Marvinbryantia.id.2005 | IBD | IVW | 1.034(0.875,1.221) | 0.697 |
| genus.Marvinbryantia.id.2005 | IBD | Weighted median | 1.03(0.827,1.282) | 0.792 |
| genus.Marvinbryantia.id.2005 | IBD | Weighted mode | 1.009(0.705,1.443) | 0.962 |
| genus.Marvinbryantia.id.2005 | IBD | MR-Robust | 1.032(0.895,1.19) | 0.666 |
| genus.Marvinbryantia.id.2005 | IBD | MR-Egger | 1.013(0.542,1.893) | 0.968 |
| genus.Marvinbryantia.id.2005 | IBD | MRRAPS | 1.034(0.862,1.241) | 0.716 |
| genus.Marvinbryantia.id.2005 | IBD | MRPRESSO | 1.034(0.918,1.165) | 0.598 |
| genus.Methanobrevibacter.id.123 | IBD | IVW | 1.004(0.827,1.219) | 0.967 |
| genus.Methanobrevibacter.id.123 | IBD | Weighted median | 0.977(0.814,1.172) | 0.802 |
| genus.Methanobrevibacter.id.123 | IBD | Weighted mode | 0.882(0.666,1.168) | 0.379 |
| genus.Methanobrevibacter.id.123 | IBD | MR-Robust | 1.001(0.83,1.207) | 0.989 |
| genus.Methanobrevibacter.id.123 | IBD | MR-Egger | 0.733(0.341,1.575) | 0.426 |
| genus.Methanobrevibacter.id.123 | IBD | MRRAPS | 0.983(0.801,1.206) | 0.868 |
| genus.Methanobrevibacter.id.123 | IBD | MRPRESSO | 1.004(0.827,1.219) | 0.969 |
| genus.Odoribacter.id.952 | IBD | IVW | 1.21(0.956,1.531) | 0.113 |
| genus.Odoribacter.id.952 | IBD | Weighted median | 1.186(0.871,1.614) | 0.279 |
| genus.Odoribacter.id.952 | IBD | Weighted mode | 1.162(0.764,1.767) | 0.482 |
| genus.Odoribacter.id.952 | IBD | MR-Robust | 1.18(0.91,1.53) | 0.212 |
| genus.Odoribacter.id.952 | IBD | MR-Egger | 0.975(0.457,2.078) | 0.948 |
| genus.Odoribacter.id.952 | IBD | MRRAPS | 1.205(0.93,1.562) | 0.157 |
| genus.Odoribacter.id.952 | IBD | MRPRESSO | 1.21(1,1.464) | 0.098 |
| genus.Olsenella.id.822 | IBD | IVW | 0.915(0.832,1.007) | 0.070 |
| genus.Olsenella.id.822 | IBD | Weighted median | 0.911(0.797,1.04) | 0.167 |
| genus.Olsenella.id.822 | IBD | Weighted mode | 0.93(0.752,1.148) | 0.499 |
| genus.Olsenella.id.822 | IBD | MR-Robust | 0.919(0.83,1.018) | 0.107 |
| genus.Olsenella.id.822 | IBD | MR-Egger | 0.899(0.616,1.312) | 0.580 |
| genus.Olsenella.id.822 | IBD | MRRAPS | 0.915(0.824,1.016) | 0.097 |
| genus.Olsenella.id.822 | IBD | MRPRESSO | 0.915(0.836,1.002) | 0.083 |
| genus.Oscillibacter.id.2063 | IBD | IVW | 0.909(0.798,1.035) | 0.150 |
| genus.Oscillibacter.id.2063 | IBD | Weighted median | 0.9(0.751,1.079) | 0.254 |
| genus.Oscillibacter.id.2063 | IBD | Weighted mode | 0.916(0.666,1.26) | 0.589 |
| genus.Oscillibacter.id.2063 | IBD | MR-Robust | 0.907(0.794,1.037) | 0.155 |
| genus.Oscillibacter.id.2063 | IBD | MR-Egger | 0.998(0.6,1.659) | 0.993 |
| genus.Oscillibacter.id.2063 | IBD | MRRAPS | 0.904(0.785,1.041) | 0.160 |
| genus.Oscillibacter.id.2063 | IBD | MRPRESSO | 0.909(0.799,1.034) | 0.172 |
| genus.Oscillospira.id.2064 | IBD | IVW | 0.864(0.706,1.057) | 0.156 |
| genus.Oscillospira.id.2064 | IBD | Weighted median | 0.915(0.71,1.179) | 0.494 |
| genus.Oscillospira.id.2064 | IBD | Weighted mode | 0.962(0.648,1.427) | 0.847 |
| genus.Oscillospira.id.2064 | IBD | MR-Robust | 0.869(0.711,1.062) | 0.171 |
| genus.Oscillospira.id.2064 | IBD | MR-Egger | 1.022(0.409,2.553) | 0.963 |
| genus.Oscillospira.id.2064 | IBD | MRRAPS | 0.871(0.702,1.079) | 0.207 |
| genus.Oscillospira.id.2064 | IBD | MRPRESSO | 0.864(0.706,1.057) | 0.194 |
| genus.Oxalobacter.id.2978 | IBD | IVW | 1.177(1.064,1.301) | 0.001 |
| genus.Oxalobacter.id.2978 | IBD | Weighted median | 1.19(1.043,1.357) | 0.010 |
| genus.Oxalobacter.id.2978 | IBD | Weighted mode | 1.18(0.965,1.442) | 0.106 |
| genus.Oxalobacter.id.2978 | IBD | MR-Robust | 1.168(1.067,1.28) | 0.001 |
| genus.Oxalobacter.id.2978 | IBD | MR-Egger | 0.999(0.627,1.592) | 0.997 |
| genus.Oxalobacter.id.2978 | IBD | MRRAPS | 1.179(1.055,1.318) | 0.004 |
| genus.Oxalobacter.id.2978 | IBD | MRPRESSO | 1.177(1.108,1.249) | 0.000 |
| genus.Parabacteroides.id.954 | IBD | IVW | 1.097(0.858,1.404) | 0.460 |
| genus.Parabacteroides.id.954 | IBD | Weighted median | 1.119(0.825,1.518) | 0.469 |
| genus.Parabacteroides.id.954 | IBD | Weighted mode | 1.177(0.793,1.747) | 0.418 |
| genus.Parabacteroides.id.954 | IBD | MR-Robust | 1.124(0.688,1.836) | 0.640 |
| genus.Parabacteroides.id.954 | IBD | MR-Egger | 1.019(0.468,2.216) | 0.962 |
| genus.Parabacteroides.id.954 | IBD | MRRAPS | 1.098(0.837,1.44) | 0.500 |
| genus.Parabacteroides.id.954 | IBD | MRPRESSO | 1.097(0.984,1.224) | 0.157 |
| genus.Paraprevotella.id.962 | IBD | IVW | 0.978(0.869,1.101) | 0.711 |
| genus.Paraprevotella.id.962 | IBD | Weighted median | 1.002(0.853,1.177) | 0.980 |
| genus.Paraprevotella.id.962 | IBD | Weighted mode | 1.123(0.82,1.539) | 0.469 |
| genus.Paraprevotella.id.962 | IBD | MR-Robust | 0.983(0.858,1.125) | 0.800 |
| genus.Paraprevotella.id.962 | IBD | MR-Egger | 0.89(0.585,1.354) | 0.587 |
| genus.Paraprevotella.id.962 | IBD | MRRAPS | 0.976(0.843,1.13) | 0.748 |
| genus.Paraprevotella.id.962 | IBD | MRPRESSO | 0.978(0.869,1.101) | 0.718 |
| genus.Parasutterella.id.2892 | IBD | IVW | 1.072(0.939,1.225) | 0.303 |
| genus.Parasutterella.id.2892 | IBD | Weighted median | 1.034(0.862,1.241) | 0.716 |
| genus.Parasutterella.id.2892 | IBD | Weighted mode | 0.953(0.725,1.254) | 0.733 |
| genus.Parasutterella.id.2892 | IBD | MR-Robust | 1.057(0.92,1.215) | 0.433 |
| genus.Parasutterella.id.2892 | IBD | MR-Egger | 0.91(0.607,1.364) | 0.646 |
| genus.Parasutterella.id.2892 | IBD | MRRAPS | 1.061(0.918,1.225) | 0.423 |
| genus.Parasutterella.id.2892 | IBD | MRPRESSO | 1.072(0.947,1.215) | 0.291 |
| genus.Peptococcus.id.2037 | IBD | IVW | 1.071(0.966,1.188) | 0.194 |
| genus.Peptococcus.id.2037 | IBD | Weighted median | 1.115(0.973,1.278) | 0.119 |
| genus.Peptococcus.id.2037 | IBD | Weighted mode | 1.171(0.93,1.476) | 0.179 |
| genus.Peptococcus.id.2037 | IBD | MR-Robust | 1.073(0.981,1.174) | 0.123 |
| genus.Peptococcus.id.2037 | IBD | MR-Egger | 1.042(0.7,1.552) | 0.839 |
| genus.Peptococcus.id.2037 | IBD | MRRAPS | 1.073(0.958,1.201) | 0.224 |
| genus.Peptococcus.id.2037 | IBD | MRPRESSO | 1.071(0.995,1.153) | 0.092 |
| genus.Phascolarctobacterium.id.2168 | IBD | IVW | 1.093(0.89,1.343) | 0.396 |
| genus.Phascolarctobacterium.id.2168 | IBD | Weighted median | 1.044(0.823,1.323) | 0.725 |
| genus.Phascolarctobacterium.id.2168 | IBD | Weighted mode | 0.994(0.654,1.511) | 0.979 |
| genus.Phascolarctobacterium.id.2168 | IBD | MR-Robust | 1.101(0.899,1.349) | 0.353 |
| genus.Phascolarctobacterium.id.2168 | IBD | MR-Egger | 2.228(0.951,5.223) | 0.065 |
| genus.Phascolarctobacterium.id.2168 | IBD | MRRAPS | 1.137(0.916,1.41) | 0.244 |
| genus.Phascolarctobacterium.id.2168 | IBD | MRPRESSO | 1.093(0.89,1.343) | 0.416 |
| genus.Prevotella7.id.11182 | IBD | IVW | 0.981(0.872,1.102) | 0.743 |
| genus.Prevotella7.id.11182 | IBD | Weighted median | 0.968(0.839,1.117) | 0.658 |
| genus.Prevotella7.id.11182 | IBD | Weighted mode | 0.838(0.634,1.107) | 0.213 |
| genus.Prevotella7.id.11182 | IBD | MR-Robust | 0.978(0.861,1.112) | 0.736 |
| genus.Prevotella7.id.11182 | IBD | MR-Egger | 1.125(0.545,2.32) | 0.750 |
| genus.Prevotella7.id.11182 | IBD | MRRAPS | 0.98(0.855,1.124) | 0.774 |
| genus.Prevotella7.id.11182 | IBD | MRPRESSO | 0.981(0.872,1.102) | 0.750 |
| genus.Prevotella9.id.11183 | IBD | IVW | 1.058(0.927,1.207) | 0.405 |
| genus.Prevotella9.id.11183 | IBD | Weighted median | 1.051(0.873,1.264) | 0.601 |
| genus.Prevotella9.id.11183 | IBD | Weighted mode | 1.062(0.795,1.419) | 0.685 |
| genus.Prevotella9.id.11183 | IBD | MR-Robust | 1.055(0.933,1.192) | 0.393 |
| genus.Prevotella9.id.11183 | IBD | MR-Egger | 0.841(0.55,1.286) | 0.424 |
| genus.Prevotella9.id.11183 | IBD | MRRAPS | 1.053(0.912,1.215) | 0.483 |
| genus.Prevotella9.id.11183 | IBD | MRPRESSO | 1.058(0.935,1.197) | 0.388 |
| genus.RikenellaceaeRC9gutgroup.id.11191 | IBD | IVW | 0.999(0.879,1.136) | 0.991 |
| genus.RikenellaceaeRC9gutgroup.id.11191 | IBD | Weighted median | 0.965(0.841,1.107) | 0.614 |
| genus.RikenellaceaeRC9gutgroup.id.11191 | IBD | Weighted mode | 1.203(0.879,1.646) | 0.250 |
| genus.RikenellaceaeRC9gutgroup.id.11191 | IBD | MR-Robust | 1(0.875,1.143) | 1.000 |
| genus.RikenellaceaeRC9gutgroup.id.11191 | IBD | MR-Egger | 2.015(1.003,4.048) | 0.049 |
| genus.RikenellaceaeRC9gutgroup.id.11191 | IBD | MRRAPS | 1.022(0.888,1.175) | 0.764 |
| genus.RikenellaceaeRC9gutgroup.id.11191 | IBD | MRPRESSO | 0.999(0.879,1.136) | 0.991 |
| genus.Romboutsia.id.11347 | IBD | IVW | 0.972(0.807,1.17) | 0.761 |
| genus.Romboutsia.id.11347 | IBD | Weighted median | 1.028(0.826,1.279) | 0.804 |
| genus.Romboutsia.id.11347 | IBD | Weighted mode | 1.021(0.755,1.381) | 0.891 |
| genus.Romboutsia.id.11347 | IBD | MR-Robust | 0.975(0.824,1.155) | 0.772 |
| genus.Romboutsia.id.11347 | IBD | MR-Egger | 1.023(0.616,1.701) | 0.929 |
| genus.Romboutsia.id.11347 | IBD | MRRAPS | 0.976(0.821,1.16) | 0.779 |
| genus.Romboutsia.id.11347 | IBD | MRPRESSO | 0.972(0.807,1.17) | 0.766 |
| genus.Roseburia.id.2012 | IBD | IVW | 1.098(0.922,1.308) | 0.294 |
| genus.Roseburia.id.2012 | IBD | Weighted median | 1.201(0.945,1.526) | 0.135 |
| genus.Roseburia.id.2012 | IBD | Weighted mode | 1.302(0.877,1.934) | 0.190 |
| genus.Roseburia.id.2012 | IBD | MR-Robust | 1.133(0.856,1.499) | 0.383 |
| genus.Roseburia.id.2012 | IBD | MR-Egger | 0.84(0.497,1.421) | 0.515 |
| genus.Roseburia.id.2012 | IBD | MRRAPS | 1.119(0.924,1.355) | 0.249 |
| genus.Roseburia.id.2012 | IBD | MRPRESSO | 1.098(0.943,1.279) | 0.250 |
| genus.Ruminiclostridium5.id.11355 | IBD | IVW | 0.762(0.554,1.048) | 0.094 |
| genus.Ruminiclostridium5.id.11355 | IBD | Weighted median | 0.904(0.66,1.237) | 0.526 |
| genus.Ruminiclostridium5.id.11355 | IBD | Weighted mode | 1.057(0.669,1.671) | 0.812 |
| genus.Ruminiclostridium5.id.11355 | IBD | MR-Robust | 0.799(0.547,1.165) | 0.244 |
| genus.Ruminiclostridium5.id.11355 | IBD | MR-Egger | 1.637(0.453,5.922) | 0.452 |
| genus.Ruminiclostridium5.id.11355 | IBD | MRRAPS | 0.828(0.613,1.119) | 0.220 |
| genus.Ruminiclostridium5.id.11355 | IBD | MRPRESSO | 0.762(0.554,1.048) | 0.125 |
| genus.Ruminiclostridium6.id.11356 | IBD | IVW | 0.957(0.787,1.164) | 0.659 |
| genus.Ruminiclostridium6.id.11356 | IBD | Weighted median | 0.862(0.697,1.065) | 0.169 |
| genus.Ruminiclostridium6.id.11356 | IBD | Weighted mode | 0.773(0.503,1.188) | 0.241 |
| genus.Ruminiclostridium6.id.11356 | IBD | MR-Robust | 0.856(0.638,1.148) | 0.299 |
| genus.Ruminiclostridium6.id.11356 | IBD | MR-Egger | 0.713(0.44,1.155) | 0.169 |
| genus.Ruminiclostridium6.id.11356 | IBD | MRRAPS | 0.869(0.712,1.061) | 0.168 |
| genus.Ruminiclostridium6.id.11356 | IBD | MRPRESSO | 0.957(0.787,1.164) | 0.666 |
| genus.Ruminiclostridium9.id.11357 | IBD | IVW | 1.108(0.791,1.551) | 0.551 |
| genus.Ruminiclostridium9.id.11357 | IBD | Weighted median | 1.156(0.839,1.592) | 0.376 |
| genus.Ruminiclostridium9.id.11357 | IBD | Weighted mode | 1.165(0.622,2.182) | 0.633 |
| genus.Ruminiclostridium9.id.11357 | IBD | MR-Robust | 1.096(0.766,1.567) | 0.615 |
| genus.Ruminiclostridium9.id.11357 | IBD | MR-Egger | 1.179(0.245,5.672) | 0.837 |
| genus.Ruminiclostridium9.id.11357 | IBD | MRRAPS | 1.064(0.719,1.573) | 0.757 |
| genus.Ruminiclostridium9.id.11357 | IBD | MRPRESSO | 1.108(0.791,1.551) | 0.565 |
| genus.RuminococcaceaeNK4A214group.id.11358 | IBD | IVW | 0.894(0.702,1.139) | 0.364 |
| genus.RuminococcaceaeNK4A214group.id.11358 | IBD | Weighted median | 0.988(0.76,1.284) | 0.927 |
| genus.RuminococcaceaeNK4A214group.id.11358 | IBD | Weighted mode | 1.094(0.664,1.804) | 0.724 |
| genus.RuminococcaceaeNK4A214group.id.11358 | IBD | MR-Robust | 0.895(0.698,1.148) | 0.382 |
| genus.RuminococcaceaeNK4A214group.id.11358 | IBD | MR-Egger | 0.927(0.37,2.32) | 0.872 |
| genus.RuminococcaceaeNK4A214group.id.11358 | IBD | MRRAPS | 0.906(0.676,1.215) | 0.510 |
| genus.RuminococcaceaeNK4A214group.id.11358 | IBD | MRPRESSO | 0.894(0.702,1.139) | 0.379 |
| genus.RuminococcaceaeUCG002.id.11360 | IBD | IVW | 0.933(0.816,1.066) | 0.307 |
| genus.RuminococcaceaeUCG002.id.11360 | IBD | Weighted median | 0.911(0.758,1.095) | 0.321 |
| genus.RuminococcaceaeUCG002.id.11360 | IBD | Weighted mode | 0.888(0.666,1.183) | 0.417 |
| genus.RuminococcaceaeUCG002.id.11360 | IBD | MR-Robust | 0.952(0.818,1.107) | 0.519 |
| genus.RuminococcaceaeUCG002.id.11360 | IBD | MR-Egger | 0.907(0.623,1.318) | 0.608 |
| genus.RuminococcaceaeUCG002.id.11360 | IBD | MRRAPS | 0.947(0.819,1.094) | 0.459 |
| genus.RuminococcaceaeUCG002.id.11360 | IBD | MRPRESSO | 0.933(0.821,1.06) | 0.297 |
| genus.RuminococcaceaeUCG003.id.11361 | IBD | IVW | 0.891(0.684,1.162) | 0.395 |
| genus.RuminococcaceaeUCG003.id.11361 | IBD | Weighted median | 0.958(0.751,1.221) | 0.728 |
| genus.RuminococcaceaeUCG003.id.11361 | IBD | Weighted mode | 0.952(0.665,1.361) | 0.786 |
| genus.RuminococcaceaeUCG003.id.11361 | IBD | MR-Robust | 0.847(0.684,1.048) | 0.127 |
| genus.RuminococcaceaeUCG003.id.11361 | IBD | MR-Egger | 0.59(0.246,1.417) | 0.238 |
| genus.RuminococcaceaeUCG003.id.11361 | IBD | MRRAPS | 0.818(0.65,1.029) | 0.086 |
| genus.RuminococcaceaeUCG003.id.11361 | IBD | MRPRESSO | 0.891(0.684,1.162) | 0.411 |
| genus.RuminococcaceaeUCG004.id.11362 | IBD | IVW | 1.088(0.939,1.26) | 0.262 |
| genus.RuminococcaceaeUCG004.id.11362 | IBD | Weighted median | 1.151(0.943,1.404) | 0.167 |
| genus.RuminococcaceaeUCG004.id.11362 | IBD | Weighted mode | 1.183(0.833,1.681) | 0.348 |
| genus.RuminococcaceaeUCG004.id.11362 | IBD | MR-Robust | 1.096(0.934,1.287) | 0.260 |
| genus.RuminococcaceaeUCG004.id.11362 | IBD | MR-Egger | 0.709(0.318,1.577) | 0.399 |
| genus.RuminococcaceaeUCG004.id.11362 | IBD | MRRAPS | 1.101(0.939,1.291) | 0.236 |
| genus.RuminococcaceaeUCG004.id.11362 | IBD | MRPRESSO | 1.088(0.941,1.257) | 0.283 |
| genus.RuminococcaceaeUCG005.id.11363 | IBD | IVW | 0.938(0.775,1.135) | 0.511 |
| genus.RuminococcaceaeUCG005.id.11363 | IBD | Weighted median | 0.969(0.772,1.216) | 0.785 |
| genus.RuminococcaceaeUCG005.id.11363 | IBD | Weighted mode | 1.004(0.716,1.408) | 0.980 |
| genus.RuminococcaceaeUCG005.id.11363 | IBD | MR-Robust | 0.902(0.772,1.053) | 0.192 |
| genus.RuminococcaceaeUCG005.id.11363 | IBD | MR-Egger | 0.984(0.554,1.748) | 0.957 |
| genus.RuminococcaceaeUCG005.id.11363 | IBD | MRRAPS | 0.908(0.756,1.089) | 0.297 |
| genus.RuminococcaceaeUCG005.id.11363 | IBD | MRPRESSO | 0.938(0.775,1.135) | 0.522 |
| genus.RuminococcaceaeUCG009.id.11366 | IBD | IVW | 0.9(0.791,1.025) | 0.111 |
| genus.RuminococcaceaeUCG009.id.11366 | IBD | Weighted median | 0.896(0.757,1.061) | 0.204 |
| genus.RuminococcaceaeUCG009.id.11366 | IBD | Weighted mode | 0.901(0.694,1.171) | 0.436 |
| genus.RuminococcaceaeUCG009.id.11366 | IBD | MR-Robust | 0.92(0.814,1.04) | 0.184 |
| genus.RuminococcaceaeUCG009.id.11366 | IBD | MR-Egger | 0.754(0.427,1.33) | 0.329 |
| genus.RuminococcaceaeUCG009.id.11366 | IBD | MRRAPS | 0.912(0.792,1.05) | 0.198 |
| genus.RuminococcaceaeUCG009.id.11366 | IBD | MRPRESSO | 0.9(0.804,1.008) | 0.096 |
| genus.RuminococcaceaeUCG010.id.11367 | IBD | IVW | 1.05(0.806,1.369) | 0.717 |
| genus.RuminococcaceaeUCG010.id.11367 | IBD | Weighted median | 1.109(0.82,1.498) | 0.503 |
| genus.RuminococcaceaeUCG010.id.11367 | IBD | Weighted mode | 1.173(0.713,1.93) | 0.530 |
| genus.RuminococcaceaeUCG010.id.11367 | IBD | MR-Robust | 1.051(0.812,1.361) | 0.704 |
| genus.RuminococcaceaeUCG010.id.11367 | IBD | MR-Egger | 1.316(0.512,3.381) | 0.569 |
| genus.RuminococcaceaeUCG010.id.11367 | IBD | MRRAPS | 1.069(0.811,1.409) | 0.637 |
| genus.RuminococcaceaeUCG010.id.11367 | IBD | MRPRESSO | 1.05(0.806,1.369) | 0.732 |
| genus.RuminococcaceaeUCG011.id.11368 | IBD | IVW | 0.964(0.871,1.067) | 0.479 |
| genus.RuminococcaceaeUCG011.id.11368 | IBD | Weighted median | 0.988(0.863,1.131) | 0.857 |
| genus.RuminococcaceaeUCG011.id.11368 | IBD | Weighted mode | 0.928(0.749,1.15) | 0.495 |
| genus.RuminococcaceaeUCG011.id.11368 | IBD | MR-Robust | 0.963(0.88,1.054) | 0.411 |
| genus.RuminococcaceaeUCG011.id.11368 | IBD | MR-Egger | 0.993(0.589,1.675) | 0.979 |
| genus.RuminococcaceaeUCG011.id.11368 | IBD | MRRAPS | 0.963(0.862,1.075) | 0.499 |
| genus.RuminococcaceaeUCG011.id.11368 | IBD | MRPRESSO | 0.964(0.879,1.057) | 0.457 |
| genus.RuminococcaceaeUCG013.id.11370 | IBD | IVW | 0.98(0.82,1.17) | 0.820 |
| genus.RuminococcaceaeUCG013.id.11370 | IBD | Weighted median | 0.93(0.732,1.183) | 0.557 |
| genus.RuminococcaceaeUCG013.id.11370 | IBD | Weighted mode | 0.873(0.622,1.225) | 0.431 |
| genus.RuminococcaceaeUCG013.id.11370 | IBD | MR-Robust | 0.969(0.82,1.145) | 0.713 |
| genus.RuminococcaceaeUCG013.id.11370 | IBD | MR-Egger | 0.867(0.534,1.407) | 0.563 |
| genus.RuminococcaceaeUCG013.id.11370 | IBD | MRRAPS | 0.965(0.796,1.169) | 0.712 |
| genus.RuminococcaceaeUCG013.id.11370 | IBD | MRPRESSO | 0.98(0.828,1.159) | 0.814 |
| genus.RuminococcaceaeUCG014.id.11371 | IBD | IVW | 1.252(1.052,1.49) | 0.012 |
| genus.RuminococcaceaeUCG014.id.11371 | IBD | Weighted median | 1.215(0.956,1.543) | 0.112 |
| genus.RuminococcaceaeUCG014.id.11371 | IBD | Weighted mode | 1.165(0.816,1.665) | 0.401 |
| genus.RuminococcaceaeUCG014.id.11371 | IBD | MR-Robust | 1.229(0.978,1.543) | 0.076 |
| genus.RuminococcaceaeUCG014.id.11371 | IBD | MR-Egger | 1.294(0.786,2.131) | 0.311 |
| genus.RuminococcaceaeUCG014.id.11371 | IBD | MRRAPS | 1.251(1.033,1.515) | 0.022 |
| genus.RuminococcaceaeUCG014.id.11371 | IBD | MRPRESSO | 1.252(1.073,1.461) | 0.017 |
| genus.Ruminococcus1.id.11373 | IBD | IVW | 1.11(0.853,1.443) | 0.439 |
| genus.Ruminococcus1.id.11373 | IBD | Weighted median | 1.012(0.757,1.353) | 0.937 |
| genus.Ruminococcus1.id.11373 | IBD | Weighted mode | 0.993(0.631,1.562) | 0.976 |
| genus.Ruminococcus1.id.11373 | IBD | MR-Robust | 1.089(0.81,1.465) | 0.572 |
| genus.Ruminococcus1.id.11373 | IBD | MR-Egger | 1.328(0.601,2.931) | 0.483 |
| genus.Ruminococcus1.id.11373 | IBD | MRRAPS | 1.102(0.863,1.408) | 0.436 |
| genus.Ruminococcus1.id.11373 | IBD | MRPRESSO | 1.11(0.853,1.443) | 0.459 |
| genus.Ruminococcus2.id.11374 | IBD | IVW | 0.904(0.773,1.058) | 0.209 |
| genus.Ruminococcus2.id.11374 | IBD | Weighted median | 0.854(0.689,1.06) | 0.153 |
| genus.Ruminococcus2.id.11374 | IBD | Weighted mode | 1.048(0.726,1.513) | 0.803 |
| genus.Ruminococcus2.id.11374 | IBD | MR-Robust | 0.906(0.782,1.05) | 0.190 |
| genus.Ruminococcus2.id.11374 | IBD | MR-Egger | 0.695(0.454,1.065) | 0.095 |
| genus.Ruminococcus2.id.11374 | IBD | MRRAPS | 0.899(0.758,1.067) | 0.224 |
| genus.Ruminococcus2.id.11374 | IBD | MRPRESSO | 0.904(0.795,1.028) | 0.147 |
| genus.Sellimonas.id.14369 | IBD | IVW | 0.949(0.869,1.035) | 0.239 |
| genus.Sellimonas.id.14369 | IBD | Weighted median | 0.929(0.825,1.045) | 0.220 |
| genus.Sellimonas.id.14369 | IBD | Weighted mode | 0.895(0.737,1.087) | 0.262 |
| genus.Sellimonas.id.14369 | IBD | MR-Robust | 0.944(0.86,1.036) | 0.223 |
| genus.Sellimonas.id.14369 | IBD | MR-Egger | 0.914(0.604,1.382) | 0.670 |
| genus.Sellimonas.id.14369 | IBD | MRRAPS | 0.944(0.859,1.038) | 0.234 |
| genus.Sellimonas.id.14369 | IBD | MRPRESSO | 0.949(0.874,1.03) | 0.238 |
| genus.Senegalimassilia.id.11160 | IBD | IVW | 0.966(0.779,1.199) | 0.756 |
| genus.Senegalimassilia.id.11160 | IBD | Weighted median | 0.892(0.693,1.149) | 0.376 |
| genus.Senegalimassilia.id.11160 | IBD | Weighted mode | 0.849(0.6,1.2) | 0.353 |
| genus.Senegalimassilia.id.11160 | IBD | MR-Robust | 0.964(0.791,1.175) | 0.717 |
| genus.Senegalimassilia.id.11160 | IBD | MR-Egger | 0.67(0.319,1.408) | 0.291 |
| genus.Senegalimassilia.id.11160 | IBD | MRRAPS | 0.955(0.769,1.187) | 0.679 |
| genus.Senegalimassilia.id.11160 | IBD | MRPRESSO | 0.966(0.779,1.199) | 0.771 |
| genus.Slackia.id.825 | IBD | IVW | 0.92(0.783,1.081) | 0.308 |
| genus.Slackia.id.825 | IBD | Weighted median | 0.915(0.744,1.126) | 0.402 |
| genus.Slackia.id.825 | IBD | Weighted mode | 1.071(0.75,1.529) | 0.706 |
| genus.Slackia.id.825 | IBD | MR-Robust | 0.919(0.787,1.074) | 0.288 |
| genus.Slackia.id.825 | IBD | MR-Egger | 0.769(0.284,2.082) | 0.605 |
| genus.Slackia.id.825 | IBD | MRRAPS | 0.917(0.769,1.092) | 0.331 |
| genus.Slackia.id.825 | IBD | MRPRESSO | 0.92(0.788,1.074) | 0.337 |
| genus.Streptococcus.id.1853 | IBD | IVW | 1.013(0.858,1.197) | 0.876 |
| genus.Streptococcus.id.1853 | IBD | Weighted median | 1.055(0.853,1.304) | 0.622 |
| genus.Streptococcus.id.1853 | IBD | Weighted mode | 1.136(0.773,1.669) | 0.516 |
| genus.Streptococcus.id.1853 | IBD | MR-Robust | 1.038(0.874,1.233) | 0.668 |
| genus.Streptococcus.id.1853 | IBD | MR-Egger | 0.97(0.517,1.820) | 0.924 |
| genus.Streptococcus.id.1853 | IBD | MRRAPS | 1.048(0.878,1.25) | 0.606 |
| genus.Streptococcus.id.1853 | IBD | MRPRESSO | 1.013(0.858,1.197) | 0.878 |
| genus.Subdoligranulum.id.2070 | IBD | IVW | 1.127(0.906,1.402) | 0.283 |
| genus.Subdoligranulum.id.2070 | IBD | Weighted median | 1.112(0.856,1.445) | 0.427 |
| genus.Subdoligranulum.id.2070 | IBD | Weighted mode | 1.149(0.769,1.717) | 0.497 |
| genus.Subdoligranulum.id.2070 | IBD | MR-Robust | 1.120(0.924,1.357) | 0.249 |
| genus.Subdoligranulum.id.2070 | IBD | MR-Egger | 1.325(0.731,2.400) | 0.353 |
| genus.Subdoligranulum.id.2070 | IBD | MRRAPS | 1.124(0.912,1.385) | 0.274 |
| genus.Subdoligranulum.id.2070 | IBD | MRPRESSO | 1.127(0.906,1.402) | 0.309 |
| genus.Sutterella.id.2896 | IBD | IVW | 0.898(0.760,1.061) | 0.205 |
| genus.Sutterella.id.2896 | IBD | Weighted median | 0.897(0.72,1.118) | 0.334 |
| genus.Sutterella.id.2896 | IBD | Weighted mode | 0.927(0.667,1.287) | 0.649 |
| genus.Sutterella.id.2896 | IBD | MR-Robust | 0.888(0.755,1.045) | 0.153 |
| genus.Sutterella.id.2896 | IBD | MR-Egger | 0.735(0.325,1.663) | 0.459 |
| genus.Sutterella.id.2896 | IBD | MRRAPS | 0.890(0.742,1.066) | 0.206 |
| genus.Sutterella.id.2896 | IBD | MRPRESSO | 0.898(0.764,1.056) | 0.218 |
| genus.Terrisporobacter.id.11348 | IBD | IVW | 0.950(0.782,1.153) | 0.602 |
| genus.Terrisporobacter.id.11348 | IBD | Weighted median | 0.905(0.718,1.141) | 0.398 |
| genus.Terrisporobacter.id.11348 | IBD | Weighted mode | 0.883(0.648,1.202) | 0.428 |
| genus.Terrisporobacter.id.11348 | IBD | MR-Robust | 0.947(0.795,1.128) | 0.542 |
| genus.Terrisporobacter.id.11348 | IBD | MR-Egger | 0.903(0.489,1.668) | 0.745 |
| genus.Terrisporobacter.id.11348 | IBD | MRRAPS | 0.941(0.776,1.140) | 0.533 |
| genus.Terrisporobacter.id.11348 | IBD | MRPRESSO | 0.950(0.782,1.153) | 0.629 |
| genus.Turicibacter.id.2162 | IBD | IVW | 1.150(0.988,1.339) | 0.071 |
| genus.Turicibacter.id.2162 | IBD | Weighted median | 1.148(0.943,1.398) | 0.168 |
| genus.Turicibacter.id.2162 | IBD | Weighted mode | 1.096(0.805,1.491) | 0.561 |
| genus.Turicibacter.id.2162 | IBD | MR-Robust | 1.142(0.991,1.316) | 0.067 |
| genus.Turicibacter.id.2162 | IBD | MR-Egger | 0.697(0.367,1.326) | 0.271 |
| genus.Turicibacter.id.2162 | IBD | MRRAPS | 1.153(0.976,1.362) | 0.095 |
| genus.Turicibacter.id.2162 | IBD | MRPRESSO | 1.150(1.028,1.287) | 0.037 |
| genus.Tyzzerella3.id.11335 | IBD | IVW | 1.045(0.944,1.156) | 0.398 |
| genus.Tyzzerella3.id.11335 | IBD | Weighted median | 1.018(0.889,1.165) | 0.799 |
| genus.Tyzzerella3.id.11335 | IBD | Weighted mode | 0.948(0.751,1.197) | 0.653 |
| genus.Tyzzerella3.id.11335 | IBD | MR-Robust | 1.043(0.954,1.139) | 0.355 |
| genus.Tyzzerella3.id.11335 | IBD | MR-Egger | 1.551(0.890,2.704) | 0.121 |
| genus.Tyzzerella3.id.11335 | IBD | MRRAPS | 1.046(0.937,1.167) | 0.425 |
| genus.Tyzzerella3.id.11335 | IBD | MRPRESSO | 1.045(0.965,1.131) | 0.299 |
| genus.unknowngenus.id.1000000073 | IBD | IVW | 0.927(0.816,1.054) | 0.249 |
| genus.unknowngenus.id.1000000073 | IBD | Weighted median | 0.972(0.812,1.163) | 0.757 |
| genus.unknowngenus.id.1000000073 | IBD | Weighted mode | 1.008(0.746,1.363) | 0.957 |
| genus.unknowngenus.id.1000000073 | IBD | MR-Robust | 0.928(0.816,1.055) | 0.254 |
| genus.unknowngenus.id.1000000073 | IBD | MR-Egger | 1.143(0.794,1.647) | 0.472 |
| genus.unknowngenus.id.1000000073 | IBD | MRRAPS | 0.923(0.804,1.061) | 0.259 |
| genus.unknowngenus.id.1000000073 | IBD | MRPRESSO | 0.927(0.818,1.052) | 0.261 |
| genus.unknowngenus.id.1000001215 | IBD | IVW | 0.968(0.837,1.120) | 0.660 |
| genus.unknowngenus.id.1000001215 | IBD | Weighted median | 0.979(0.828,1.158) | 0.803 |
| genus.unknowngenus.id.1000001215 | IBD | Weighted mode | 1.006(0.803,1.261) | 0.956 |
| genus.unknowngenus.id.1000001215 | IBD | MR-Robust | 0.971(0.853,1.104) | 0.650 |
| genus.unknowngenus.id.1000001215 | IBD | MR-Egger | 0.871(0.566,1.342) | 0.531 |
| genus.unknowngenus.id.1000001215 | IBD | MRRAPS | 0.967(0.848,1.103) | 0.618 |
| genus.unknowngenus.id.1000001215 | IBD | MRPRESSO | 0.968(0.837,1.120) | 0.672 |
| genus.unknowngenus.id.1000005472 | IBD | IVW | 0.974(0.851,1.115) | 0.703 |
| genus.unknowngenus.id.1000005472 | IBD | Weighted median | 0.948(0.793,1.132) | 0.552 |
| genus.unknowngenus.id.1000005472 | IBD | Weighted mode | 0.843(0.622,1.143) | 0.272 |
| genus.unknowngenus.id.1000005472 | IBD | MR-Robust | 0.975(0.867,1.096) | 0.671 |
| genus.unknowngenus.id.1000005472 | IBD | MR-Egger | 0.805(0.543,1.192) | 0.278 |
| genus.unknowngenus.id.1000005472 | IBD | MRRAPS | 0.973(0.840,1.128) | 0.720 |
| genus.unknowngenus.id.1000005472 | IBD | MRPRESSO | 0.974(0.885,1.073) | 0.603 |
| genus.unknowngenus.id.1000005479 | IBD | IVW | 0.984(0.839,1.155) | 0.844 |
| genus.unknowngenus.id.1000005479 | IBD | Weighted median | 0.871(0.708,1.070) | 0.189 |
| genus.unknowngenus.id.1000005479 | IBD | Weighted mode | 0.842(0.594,1.194) | 0.335 |
| genus.unknowngenus.id.1000005479 | IBD | MR-Robust | 0.947(0.448,2.005) | 0.887 |
| genus.unknowngenus.id.1000005479 | IBD | MR-Egger | 0.640(0.332,1.235) | 0.183 |
| genus.unknowngenus.id.1000005479 | IBD | MRRAPS | 0.974(0.822,1.155) | 0.764 |
| genus.unknowngenus.id.1000005479 | IBD | MRPRESSO | 0.984(0.839,1.155) | 0.849 |
| genus.unknowngenus.id.1000006162 | IBD | IVW | 1.124(0.995,1.270) | 0.061 |
| genus.unknowngenus.id.1000006162 | IBD | Weighted median | 1.078(0.941,1.236) | 0.277 |
| genus.unknowngenus.id.1000006162 | IBD | Weighted mode | 1.084(0.871,1.350) | 0.470 |
| genus.unknowngenus.id.1000006162 | IBD | MR-Robust | 1.122(1.000,1.258) | 0.049 |
| genus.unknowngenus.id.1000006162 | IBD | MR-Egger | 0.810(0.499,1.316) | 0.396 |
| genus.unknowngenus.id.1000006162 | IBD | MRRAPS | 1.121(0.991,1.268) | 0.068 |
| genus.unknowngenus.id.1000006162 | IBD | MRPRESSO | 1.124(0.995,1.270) | 0.083 |
| genus.unknowngenus.id.1868 | IBD | IVW | 0.998(0.864,1.152) | 0.976 |
| genus.unknowngenus.id.1868 | IBD | Weighted median | 1.035(0.855,1.252) | 0.724 |
| genus.unknowngenus.id.1868 | IBD | Weighted mode | 1.036(0.789,1.361) | 0.797 |
| genus.unknowngenus.id.1868 | IBD | MR-Robust | 1.030(0.871,1.219) | 0.729 |
| genus.unknowngenus.id.1868 | IBD | MR-Egger | 1.146(0.706,1.861) | 0.581 |
| genus.unknowngenus.id.1868 | IBD | MRRAPS | 1.025(0.882,1.191) | 0.747 |
| genus.unknowngenus.id.1868 | IBD | MRPRESSO | 0.998(0.864,1.152) | 0.977 |
| genus.unknowngenus.id.2001 | IBD | IVW | 0.990(0.838,1.169) | 0.905 |
| genus.unknowngenus.id.2001 | IBD | Weighted median | 1.078(0.871,1.335) | 0.489 |
| genus.unknowngenus.id.2001 | IBD | Weighted mode | 1.083(0.779,1.506) | 0.634 |
| genus.unknowngenus.id.2001 | IBD | MR-Robust | 0.999(0.798,1.250) | 0.990 |
| genus.unknowngenus.id.2001 | IBD | MR-Egger | 0.843(0.476,1.492) | 0.557 |
| genus.unknowngenus.id.2001 | IBD | MRRAPS | 0.990(0.825,1.187) | 0.912 |
| genus.unknowngenus.id.2001 | IBD | MRPRESSO | 0.990(0.889,1.102) | 0.858 |
| genus.unknowngenus.id.2041 | IBD | IVW | 0.897(0.790,1.018) | 0.093 |
| genus.unknowngenus.id.2041 | IBD | Weighted median | 0.889(0.750,1.054) | 0.177 |
| genus.unknowngenus.id.2041 | IBD | Weighted mode | 0.917(0.685,1.227) | 0.561 |
| genus.unknowngenus.id.2041 | IBD | MR-Robust | 0.894(0.798,1.000) | 0.050 |
| genus.unknowngenus.id.2041 | IBD | MR-Egger | 0.811(0.553,1.189) | 0.283 |
| genus.unknowngenus.id.2041 | IBD | MRRAPS | 0.889(0.773,1.021) | 0.096 |
| genus.unknowngenus.id.2041 | IBD | MRPRESSO | 0.897(0.806,0.997) | 0.069 |
| genus.unknowngenus.id.2071 | IBD | IVW | 1.079(0.937,1.243) | 0.288 |
| genus.unknowngenus.id.2071 | IBD | Weighted median | 1.081(0.891,1.312) | 0.431 |
| genus.unknowngenus.id.2071 | IBD | Weighted mode | 1.055(0.766,1.453) | 0.744 |
| genus.unknowngenus.id.2071 | IBD | MR-Robust | 1.099(0.968,1.247) | 0.146 |
| genus.unknowngenus.id.2071 | IBD | MR-Egger | 1.095(0.559,2.145) | 0.791 |
| genus.unknowngenus.id.2071 | IBD | MRRAPS | 1.100(0.944,1.282) | 0.221 |
| genus.unknowngenus.id.2071 | IBD | MRPRESSO | 1.079(0.941,1.239) | 0.292 |
| genus.unknowngenus.id.2755 | IBD | IVW | 1.122(0.984,1.279) | 0.087 |
| genus.unknowngenus.id.2755 | IBD | Weighted median | 1.080(0.899,1.298) | 0.409 |
| genus.unknowngenus.id.2755 | IBD | Weighted mode | 1.06(0.776,1.447) | 0.715 |
| genus.unknowngenus.id.2755 | IBD | MR-Robust | 1.120(0.975,1.286) | 0.109 |
| genus.unknowngenus.id.2755 | IBD | MR-Egger | 0.952(0.531,1.706) | 0.869 |
| genus.unknowngenus.id.2755 | IBD | MRRAPS | 1.131(0.980,1.304) | 0.093 |
| genus.unknowngenus.id.2755 | IBD | MRPRESSO | 1.122(0.989,1.272) | 0.099 |
| genus.unknowngenus.id.826 | IBD | IVW | 1.130(0.974,1.311) | 0.106 |
| genus.unknowngenus.id.826 | IBD | Weighted median | 1.112(0.908,1.362) | 0.306 |
| genus.unknowngenus.id.826 | IBD | Weighted mode | 1.084(0.814,1.443) | 0.582 |
| genus.unknowngenus.id.826 | IBD | MR-Robust | 1.146(0.965,1.362) | 0.120 |
| genus.unknowngenus.id.826 | IBD | MR-Egger | 1.236(0.821,1.861) | 0.309 |
| genus.unknowngenus.id.826 | IBD | MRRAPS | 1.146(0.981,1.34) | 0.087 |
| genus.unknowngenus.id.826 | IBD | MRPRESSO | 1.130(0.974,1.311) | 0.128 |
| genus.unknowngenus.id.959 | IBD | IVW | 1.022(0.925,1.129) | 0.671 |
| genus.unknowngenus.id.959 | IBD | Weighted median | 0.973(0.845,1.120) | 0.701 |
| genus.unknowngenus.id.959 | IBD | Weighted mode | 0.918(0.726,1.160) | 0.472 |
| genus.unknowngenus.id.959 | IBD | MR-Robust | 1.014(0.907,1.133) | 0.809 |
| genus.unknowngenus.id.959 | IBD | MR-Egger | 0.951(0.517,1.747) | 0.871 |
| genus.unknowngenus.id.959 | IBD | MRRAPS | 1.012(0.908,1.128) | 0.827 |
| genus.unknowngenus.id.959 | IBD | MRPRESSO | 1.022(0.926,1.127) | 0.673 |
| genus.Veillonella.id.2198 | IBD | IVW | 0.965(0.791,1.176) | 0.722 |
| genus.Veillonella.id.2198 | IBD | Weighted median | 1.061(0.837,1.345) | 0.622 |
| genus.Veillonella.id.2198 | IBD | Weighted mode | 1.158(0.762,1.758) | 0.492 |
| genus.Veillonella.id.2198 | IBD | MR-Robust | 0.969(0.770,1.219) | 0.789 |
| genus.Veillonella.id.2198 | IBD | MR-Egger | 0.683(0.239,1.950) | 0.476 |
| genus.Veillonella.id.2198 | IBD | MRRAPS | 0.953(0.762,1.193) | 0.677 |
| genus.Veillonella.id.2198 | IBD | MRPRESSO | 0.965(0.791,1.176) | 0.732 |
| genus.Victivallis.id.2256 | IBD | IVW | 1.080(0.988,1.181) | 0.092 |
| genus.Victivallis.id.2256 | IBD | Weighted median | 1.062(0.944,1.195) | 0.319 |
| genus.Victivallis.id.2256 | IBD | Weighted mode | 1.009(0.832,1.223) | 0.931 |
| genus.Victivallis.id.2256 | IBD | MR-Robust | 1.079(1.003,1.162) | 0.042 |
| genus.Victivallis.id.2256 | IBD | MR-Egger | 0.956(0.533,1.714) | 0.881 |
| genus.Victivallis.id.2256 | IBD | MRRAPS | 1.082(0.980,1.193) | 0.117 |
| genus.Victivallis.id.2256 | IBD | MRPRESSO | 1.080(1.014,1.150) | 0.037 |
| order.Actinomycetales.id.420 | IBD | IVW | 1.049(0.857,1.284) | 0.641 |
| order.Actinomycetales.id.420 | IBD | Weighted median | 1.145(0.884,1.482) | 0.305 |
| order.Actinomycetales.id.420 | IBD | Weighted mode | 1.194(0.865,1.649) | 0.280 |
| order.Actinomycetales.id.420 | IBD | MR-Robust | 1.113(0.705,1.757) | 0.646 |
| order.Actinomycetales.id.420 | IBD | MR-Egger | 1.011(0.525,1.947) | 0.974 |
| order.Actinomycetales.id.420 | IBD | MRRAPS | 1.075(0.867,1.332) | 0.511 |
| order.Actinomycetales.id.420 | IBD | MRPRESSO | 1.049(0.857,1.284) | 0.665 |
| order.Bacillales.id.1674 | IBD | IVW | 0.933(0.846,1.029) | 0.164 |
| order.Bacillales.id.1674 | IBD | Weighted median | 0.941(0.825,1.073) | 0.364 |
| order.Bacillales.id.1674 | IBD | Weighted mode | 0.948(0.772,1.163) | 0.608 |
| order.Bacillales.id.1674 | IBD | MR-Robust | 0.931(0.855,1.013) | 0.097 |
| order.Bacillales.id.1674 | IBD | MR-Egger | 0.768(0.486,1.214) | 0.259 |
| order.Bacillales.id.1674 | IBD | MRRAPS | 0.931(0.836,1.037) | 0.194 |
| order.Bacillales.id.1674 | IBD | MRPRESSO | 0.933(0.867,1.003) | 0.098 |
| order.Bacteroidales.id.913 | IBD | IVW | 0.976(0.810,1.175) | 0.796 |
| order.Bacteroidales.id.913 | IBD | Weighted median | 0.980(0.765,1.254) | 0.872 |
| order.Bacteroidales.id.913 | IBD | Weighted mode | 1.047(0.733,1.495) | 0.800 |
| order.Bacteroidales.id.913 | IBD | MR-Robust | 0.956(0.745,1.228) | 0.725 |
| order.Bacteroidales.id.913 | IBD | MR-Egger | 1.352(0.925,1.977) | 0.119 |
| order.Bacteroidales.id.913 | IBD | MRRAPS | 0.977(0.793,1.204) | 0.828 |
| order.Bacteroidales.id.913 | IBD | MRPRESSO | 0.976(0.81,1.175) | 0.800 |
| order.Bifidobacteriales.id.432 | IBD | IVW | 0.828(0.727,0.943) | 0.004 |
| order.Bifidobacteriales.id.432 | IBD | Weighted median | 0.841(0.705,1.004) | 0.055 |
| order.Bifidobacteriales.id.432 | IBD | Weighted mode | 0.860(0.637,1.162) | 0.326 |
| order.Bifidobacteriales.id.432 | IBD | MR-Robust | 0.823(0.717,0.945) | 0.006 |
| order.Bifidobacteriales.id.432 | IBD | MR-Egger | 0.850(0.510,1.417) | 0.534 |
| order.Bifidobacteriales.id.432 | IBD | MRRAPS | 0.808(0.699,0.935) | 0.004 |
| order.Bifidobacteriales.id.432 | IBD | MRPRESSO | 0.828(0.727,0.943) | 0.009 |
| order.Burkholderiales.id.2874 | IBD | IVW | 0.962(0.791,1.171) | 0.702 |
| order.Burkholderiales.id.2874 | IBD | Weighted median | 1.000(0.769,1.299) | 0.997 |
| order.Burkholderiales.id.2874 | IBD | Weighted mode | 0.969(0.66,1.423) | 0.873 |
| order.Burkholderiales.id.2874 | IBD | MR-Robust | 0.984(0.811,1.193) | 0.867 |
| order.Burkholderiales.id.2874 | IBD | MR-Egger | 0.701(0.358,1.370) | 0.298 |
| order.Burkholderiales.id.2874 | IBD | MRRAPS | 0.977(0.790,1.209) | 0.831 |
| order.Burkholderiales.id.2874 | IBD | MRPRESSO | 0.962(0.817,1.133) | 0.655 |
| order.Clostridiales.id.1863 | IBD | IVW | 1.159(0.954,1.407) | 0.138 |
| order.Clostridiales.id.1863 | IBD | Weighted median | 1.136(0.868,1.486) | 0.352 |
| order.Clostridiales.id.1863 | IBD | Weighted mode | 1.063(0.667,1.695) | 0.796 |
| order.Clostridiales.id.1863 | IBD | MR-Robust | 1.161(0.974,1.383) | 0.096 |
| order.Clostridiales.id.1863 | IBD | MR-Egger | 1.626(0.621,4.255) | 0.322 |
| order.Clostridiales.id.1863 | IBD | MRRAPS | 1.171(0.948,1.447) | 0.143 |
| order.Clostridiales.id.1863 | IBD | MRPRESSO | 1.159(0.973,1.379) | 0.123 |
| order.Coriobacteriales.id.810 | IBD | IVW | 1.055(0.892,1.248) | 0.531 |
| order.Coriobacteriales.id.810 | IBD | Weighted median | 1.110(0.879,1.403) | 0.379 |
| order.Coriobacteriales.id.810 | IBD | Weighted mode | 1.157(0.731,1.832) | 0.533 |
| order.Coriobacteriales.id.810 | IBD | MR-Robust | 1.059(0.889,1.261) | 0.520 |
| order.Coriobacteriales.id.810 | IBD | MR-Egger | 1.173(0.591,2.325) | 0.648 |
| order.Coriobacteriales.id.810 | IBD | MRRAPS | 1.067(0.887,1.282) | 0.491 |
| order.Coriobacteriales.id.810 | IBD | MRPRESSO | 1.055(0.899,1.239) | 0.521 |
| order.Desulfovibrionales.id.3156 | IBD | IVW | 1.113(0.941,1.316) | 0.213 |
| order.Desulfovibrionales.id.3156 | IBD | Weighted median | 1.111(0.886,1.393) | 0.362 |
| order.Desulfovibrionales.id.3156 | IBD | Weighted mode | 1.138(0.819,1.582) | 0.441 |
| order.Desulfovibrionales.id.3156 | IBD | MR-Robust | 1.110(0.952,1.295) | 0.181 |
| order.Desulfovibrionales.id.3156 | IBD | MR-Egger | 1.179(0.755,1.841) | 0.468 |
| order.Desulfovibrionales.id.3156 | IBD | MRRAPS | 1.115(0.928,1.340) | 0.245 |
| order.Desulfovibrionales.id.3156 | IBD | MRPRESSO | 1.113(0.982,1.261) | 0.122 |
| order.Enterobacteriales.id.3468 | IBD | IVW | 0.914(0.737,1.133) | 0.413 |
| order.Enterobacteriales.id.3468 | IBD | Weighted median | 0.945(0.711,1.256) | 0.696 |
| order.Enterobacteriales.id.3468 | IBD | Weighted mode | 1.014(0.646,1.590) | 0.953 |
| order.Enterobacteriales.id.3468 | IBD | MR-Robust | 0.923(0.755,1.129) | 0.435 |
| order.Enterobacteriales.id.3468 | IBD | MR-Egger | 1.534(0.387,6.085) | 0.543 |
| order.Enterobacteriales.id.3468 | IBD | MRRAPS | 0.926(0.734,1.169) | 0.519 |
| order.Enterobacteriales.id.3468 | IBD | MRPRESSO | 0.914(0.747,1.118) | 0.412 |
| order.Erysipelotrichales.id.2148 | IBD | IVW | 1.030(0.850,1.248) | 0.765 |
| order.Erysipelotrichales.id.2148 | IBD | Weighted median | 1.036(0.802,1.338) | 0.787 |
| order.Erysipelotrichales.id.2148 | IBD | Weighted mode | 0.989(0.645,1.516) | 0.959 |
| order.Erysipelotrichales.id.2148 | IBD | MR-Robust | 1.013(0.818,1.254) | 0.908 |
| order.Erysipelotrichales.id.2148 | IBD | MR-Egger | 1.389(0.593,3.250) | 0.449 |
| order.Erysipelotrichales.id.2148 | IBD | MRRAPS | 1.026(0.833,1.265) | 0.807 |
| order.Erysipelotrichales.id.2148 | IBD | MRPRESSO | 1.030(0.887,1.195) | 0.706 |
| order.Gastranaerophilales.id.1591 | IBD | IVW | 0.968(0.837,1.120) | 0.660 |
| order.Gastranaerophilales.id.1591 | IBD | Weighted median | 0.979(0.828,1.158) | 0.803 |
| order.Gastranaerophilales.id.1591 | IBD | Weighted mode | 1.006(0.803,1.261) | 0.956 |
| order.Gastranaerophilales.id.1591 | IBD | MR-Robust | 0.971(0.853,1.104) | 0.650 |
| order.Gastranaerophilales.id.1591 | IBD | MR-Egger | 0.871(0.566,1.342) | 0.531 |
| order.Gastranaerophilales.id.1591 | IBD | MRRAPS | 0.967(0.848,1.103) | 0.618 |
| order.Gastranaerophilales.id.1591 | IBD | MRPRESSO | 0.968(0.837,1.120) | 0.672 |
| order.Lactobacillales.id.1800 | IBD | IVW | 0.992(0.812,1.213) | 0.938 |
| order.Lactobacillales.id.1800 | IBD | Weighted median | 1.003(0.789,1.276) | 0.980 |
| order.Lactobacillales.id.1800 | IBD | Weighted mode | 1.019(0.727,1.430) | 0.911 |
| order.Lactobacillales.id.1800 | IBD | MR-Robust | 1.005(0.835,1.211) | 0.954 |
| order.Lactobacillales.id.1800 | IBD | MR-Egger | 1.090(0.639,1.861) | 0.751 |
| order.Lactobacillales.id.1800 | IBD | MRRAPS | 1.01(0.836,1.219) | 0.921 |
| order.Lactobacillales.id.1800 | IBD | MRPRESSO | 0.992(0.812,1.213) | 0.939 |
| order.Methanobacteriales.id.120 | IBD | IVW | 0.935(0.853,1.025) | 0.152 |
| order.Methanobacteriales.id.120 | IBD | Weighted median | 0.952(0.837,1.082) | 0.448 |
| order.Methanobacteriales.id.120 | IBD | Weighted mode | 0.990(0.796,1.231) | 0.927 |
| order.Methanobacteriales.id.120 | IBD | MR-Robust | 0.935(0.853,1.026) | 0.156 |
| order.Methanobacteriales.id.120 | IBD | MR-Egger | 1.044(0.702,1.554) | 0.830 |
| order.Methanobacteriales.id.120 | IBD | MRRAPS | 0.933(0.842,1.035) | 0.191 |
| order.Methanobacteriales.id.120 | IBD | MRPRESSO | 0.935(0.853,1.025) | 0.182 |
| order.MollicutesRF9.id.11579 | IBD | IVW | 0.974(0.851,1.115) | 0.703 |
| order.MollicutesRF9.id.11579 | IBD | Weighted median | 0.948(0.793,1.132) | 0.552 |
| order.MollicutesRF9.id.11579 | IBD | Weighted mode | 0.843(0.622,1.143) | 0.272 |
| order.MollicutesRF9.id.11579 | IBD | MR-Robust | 0.975(0.867,1.096) | 0.671 |
| order.MollicutesRF9.id.11579 | IBD | MR-Egger | 0.805(0.543,1.192) | 0.278 |
| order.MollicutesRF9.id.11579 | IBD | MRRAPS | 0.973(0.840,1.128) | 0.720 |
| order.MollicutesRF9.id.11579 | IBD | MRPRESSO | 0.974(0.885,1.073) | 0.603 |
| order.NB1n.id.3953 | IBD | IVW | 1.124(0.995,1.270) | 0.061 |
| order.NB1n.id.3953 | IBD | Weighted median | 1.078(0.941,1.236) | 0.277 |
| order.NB1n.id.3953 | IBD | Weighted mode | 1.084(0.871,1.350) | 0.470 |
| order.NB1n.id.3953 | IBD | MR-Robust | 1.122(1.000,1.258) | 0.049 |
| order.NB1n.id.3953 | IBD | MR-Egger | 0.81(0.499,1.316) | 0.396 |
| order.NB1n.id.3953 | IBD | MRRAPS | 1.121(0.991,1.268) | 0.068 |
| order.NB1n.id.3953 | IBD | MRPRESSO | 1.124(0.995,1.270) | 0.083 |
| order.Pasteurellales.id.3688 | IBD | IVW | 0.993(0.867,1.138) | 0.922 |
| order.Pasteurellales.id.3688 | IBD | Weighted median | 0.915(0.771,1.086) | 0.311 |
| order.Pasteurellales.id.3688 | IBD | Weighted mode | 0.869(0.649,1.165) | 0.348 |
| order.Pasteurellales.id.3688 | IBD | MR-Robust | 0.962(0.763,1.214) | 0.746 |
| order.Pasteurellales.id.3688 | IBD | MR-Egger | 0.918(0.669,1.261) | 0.599 |
| order.Pasteurellales.id.3688 | IBD | MRRAPS | 0.961(0.825,1.118) | 0.605 |
| order.Pasteurellales.id.3688 | IBD | MRPRESSO | 0.993(0.867,1.138) | 0.924 |
| order.Rhodospirillales.id.2667 | IBD | IVW | 1.086(0.931,1.266) | 0.294 |
| order.Rhodospirillales.id.2667 | IBD | Weighted median | 1.182(0.975,1.432) | 0.089 |
| order.Rhodospirillales.id.2667 | IBD | Weighted mode | 1.173(0.829,1.660) | 0.367 |
| order.Rhodospirillales.id.2667 | IBD | MR-Robust | 1.094(0.929,1.289) | 0.280 |
| order.Rhodospirillales.id.2667 | IBD | MR-Egger | 0.771(0.320,1.856) | 0.561 |
| order.Rhodospirillales.id.2667 | IBD | MRRAPS | 1.098(0.929,1.299) | 0.273 |
| order.Rhodospirillales.id.2667 | IBD | MRPRESSO | 1.086(0.931,1.266) | 0.315 |
| order.Selenomonadales.id.2165 | IBD | IVW | 0.907(0.735,1.119) | 0.361 |
| order.Selenomonadales.id.2165 | IBD | Weighted median | 0.892(0.679,1.170) | 0.408 |
| order.Selenomonadales.id.2165 | IBD | Weighted mode | 0.980(0.637,1.509) | 0.928 |
| order.Selenomonadales.id.2165 | IBD | MR-Robust | 0.903(0.740,1.101) | 0.312 |
| order.Selenomonadales.id.2165 | IBD | MR-Egger | 0.710(0.354,1.424) | 0.334 |
| order.Selenomonadales.id.2165 | IBD | MRRAPS | 0.896(0.726,1.105) | 0.303 |
| order.Selenomonadales.id.2165 | IBD | MRPRESSO | 0.907(0.735,1.119) | 0.380 |
| order.Verrucomicrobiales.id.4030 | IBD | IVW | 0.971(0.809,1.166) | 0.756 |
| order.Verrucomicrobiales.id.4030 | IBD | Weighted median | 0.851(0.679,1.067) | 0.162 |
| order.Verrucomicrobiales.id.4030 | IBD | Weighted mode | 0.793(0.538,1.168) | 0.240 |
| order.Verrucomicrobiales.id.4030 | IBD | MR-Robust | 0.946(0.739,1.212) | 0.662 |
| order.Verrucomicrobiales.id.4030 | IBD | MR-Egger | 0.576(0.305,1.086) | 0.088 |
| order.Verrucomicrobiales.id.4030 | IBD | MRRAPS | 0.926(0.756,1.134) | 0.458 |
| order.Verrucomicrobiales.id.4030 | IBD | MRPRESSO | 0.971(0.809,1.166) | 0.762 |
| order.Victivallales.id.2254 | IBD | IVW | 0.871(0.770,0.986) | 0.028 |
| order.Victivallales.id.2254 | IBD | Weighted median | 0.845(0.718,0.996) | 0.045 |
| order.Victivallales.id.2254 | IBD | Weighted mode | 0.780(0.591,1.028) | 0.078 |
| order.Victivallales.id.2254 | IBD | MR-Robust | 0.870(0.779,0.973) | 0.014 |
| order.Victivallales.id.2254 | IBD | MR-Egger | 1.040(0.678,1.594) | 0.858 |
| order.Victivallales.id.2254 | IBD | MRRAPS | 0.867(0.757,0.994) | 0.040 |
| order.Victivallales.id.2254 | IBD | MRPRESSO | 0.871(0.779,0.973) | 0.044 |
| phylum.Actinobacteria.id.400 | IBD | IVW | 0.934(0.781,1.115) | 0.449 |
| phylum.Actinobacteria.id.400 | IBD | Weighted median | 0.848(0.683,1.054) | 0.138 |
| phylum.Actinobacteria.id.400 | IBD | Weighted mode | 0.788(0.566,1.098) | 0.160 |
| phylum.Actinobacteria.id.400 | IBD | MR-Robust | 0.894(0.736,1.084) | 0.255 |
| phylum.Actinobacteria.id.400 | IBD | MR-Egger | 0.732(0.360,1.487) | 0.389 |
| phylum.Actinobacteria.id.400 | IBD | MRRAPS | 0.895(0.751,1.067) | 0.216 |
| phylum.Actinobacteria.id.400 | IBD | MRPRESSO | 0.934(0.781,1.115) | 0.459 |
| phylum.Bacteroidetes.id.905 | IBD | IVW | 0.994(0.814,1.213) | 0.952 |
| phylum.Bacteroidetes.id.905 | IBD | Weighted median | 1.016(0.786,1.314) | 0.903 |
| phylum.Bacteroidetes.id.905 | IBD | Weighted mode | 1.057(0.748,1.495) | 0.753 |
| phylum.Bacteroidetes.id.905 | IBD | MR-Robust | 0.989(0.796,1.229) | 0.922 |
| phylum.Bacteroidetes.id.905 | IBD | MR-Egger | 1.386(0.947,2.028) | 0.093 |
| phylum.Bacteroidetes.id.905 | IBD | MRRAPS | 1.006(0.814,1.243) | 0.956 |
| phylum.Bacteroidetes.id.905 | IBD | MRPRESSO | 0.994(0.814,1.213) | 0.953 |
| phylum.Cyanobacteria.id.1500 | IBD | IVW | 0.865(0.748,1.002) | 0.053 |
| phylum.Cyanobacteria.id.1500 | IBD | Weighted median | 0.818(0.679,0.987) | 0.036 |
| phylum.Cyanobacteria.id.1500 | IBD | Weighted mode | 0.811(0.612,1.075) | 0.145 |
| phylum.Cyanobacteria.id.1500 | IBD | MR-Robust | 0.815(0.527,1.261) | 0.358 |
| phylum.Cyanobacteria.id.1500 | IBD | MR-Egger | 0.914(0.552,1.513) | 0.725 |
| phylum.Cyanobacteria.id.1500 | IBD | MRRAPS | 0.864(0.735,1.015) | 0.076 |
| phylum.Cyanobacteria.id.1500 | IBD | MRPRESSO | 0.865(0.794,0.943) | 0.013 |
| phylum.Euryarchaeota.id.55 | IBD | IVW | 0.970(0.873,1.077) | 0.564 |
| phylum.Euryarchaeota.id.55 | IBD | Weighted median | 1.018(0.893,1.161) | 0.791 |
| phylum.Euryarchaeota.id.55 | IBD | Weighted mode | 1.108(0.885,1.388) | 0.370 |
| phylum.Euryarchaeota.id.55 | IBD | MR-Robust | 0.979(0.852,1.126) | 0.767 |
| phylum.Euryarchaeota.id.55 | IBD | MR-Egger | 0.746(0.466,1.196) | 0.223 |
| phylum.Euryarchaeota.id.55 | IBD | MRRAPS | 0.964(0.853,1.090) | 0.558 |
| phylum.Euryarchaeota.id.55 | IBD | MRPRESSO | 0.970(0.873,1.077) | 0.575 |
| phylum.Firmicutes.id.1672 | IBD | IVW | 0.999(0.824,1.212) | 0.993 |
| phylum.Firmicutes.id.1672 | IBD | Weighted median | 1.001(0.778,1.286) | 0.996 |
| phylum.Firmicutes.id.1672 | IBD | Weighted mode | 0.825(0.528,1.288) | 0.397 |
| phylum.Firmicutes.id.1672 | IBD | MR-Robust | 1.004(0.807,1.250) | 0.969 |
| phylum.Firmicutes.id.1672 | IBD | MR-Egger | 0.834(0.489,1.425) | 0.507 |
| phylum.Firmicutes.id.1672 | IBD | MRRAPS | 1.001(0.795,1.260) | 0.993 |
| phylum.Firmicutes.id.1672 | IBD | MRPRESSO | 0.999(0.824,1.212) | 0.993 |
| phylum.Lentisphaerae.id.2238 | IBD | IVW | 0.866(0.770,0.973) | 0.016 |
| phylum.Lentisphaerae.id.2238 | IBD | Weighted median | 0.828(0.708,0.970) | 0.019 |
| phylum.Lentisphaerae.id.2238 | IBD | Weighted mode | 0.793(0.610,1.031) | 0.083 |
| phylum.Lentisphaerae.id.2238 | IBD | MR-Robust | 0.864(0.780,0.958) | 0.005 |
| phylum.Lentisphaerae.id.2238 | IBD | MR-Egger | 1.016(0.661,1.561) | 0.943 |
| phylum.Lentisphaerae.id.2238 | IBD | MRRAPS | 0.862(0.758,0.981) | 0.025 |
| phylum.Lentisphaerae.id.2238 | IBD | MRPRESSO | 0.866(0.785,0.955) | 0.020 |
| phylum.Proteobacteria.id.2375 | IBD | IVW | 1.083(0.784,1.494) | 0.629 |
| phylum.Proteobacteria.id.2375 | IBD | Weighted median | 1.137(0.855,1.511) | 0.377 |
| phylum.Proteobacteria.id.2375 | IBD | Weighted mode | 1.199(0.768,1.872) | 0.424 |
| phylum.Proteobacteria.id.2375 | IBD | MR-Robust | 1.094(0.822,1.454) | 0.538 |
| phylum.Proteobacteria.id.2375 | IBD | MR-Egger | 0.632(0.226,1.771) | 0.383 |
| phylum.Proteobacteria.id.2375 | IBD | MRRAPS | 1.051(0.749,1.475) | 0.775 |
| phylum.Proteobacteria.id.2375 | IBD | MRPRESSO | 1.083(0.784,1.494) | 0.638 |
| phylum.Tenericutes.id.3919 | IBD | IVW | 1.018(0.869,1.192) | 0.829 |
| phylum.Tenericutes.id.3919 | IBD | Weighted median | 0.907(0.735,1.121) | 0.367 |
| phylum.Tenericutes.id.3919 | IBD | Weighted mode | 0.847(0.604,1.189) | 0.337 |
| phylum.Tenericutes.id.3919 | IBD | MR-Robust | 1.005(0.851,1.187) | 0.952 |
| phylum.Tenericutes.id.3919 | IBD | MR-Egger | 0.954(0.562,1.620) | 0.862 |
| phylum.Tenericutes.id.3919 | IBD | MRRAPS | 0.998(0.834,1.193) | 0.979 |
| phylum.Tenericutes.id.3919 | IBD | MRPRESSO | 1.018(0.869,1.192) | 0.833 |
| phylum.Verrucomicrobia.id.3982 | IBD | IVW | 1.000(0.842,1.189) | 0.996 |
| phylum.Verrucomicrobia.id.3982 | IBD | Weighted median | 1.053(0.847,1.308) | 0.643 |
| phylum.Verrucomicrobia.id.3982 | IBD | Weighted mode | 1.117(0.746,1.672) | 0.592 |
| phylum.Verrucomicrobia.id.3982 | IBD | MR-Robust | 0.993(0.829,1.188) | 0.935 |
| phylum.Verrucomicrobia.id.3982 | IBD | MR-Egger | 0.847(0.515,1.392) | 0.512 |
| phylum.Verrucomicrobia.id.3982 | IBD | MRRAPS | 0.979(0.806,1.189) | 0.832 |
| phylum.Verrucomicrobia.id.3982 | IBD | MRPRESSO | 1.000(0.842,1.189) | 0.996 |

Abbreviations: IVW, inverse-variance weighted; MR, Mendelian randomization; MR-RAPS, Robust Adjusted Profile Score; MR-PRESSO, Mendelian Randomization Pleiotropy RESidual Sum and Outlier; IBD, inflammatory bowel disease.

**S9 Table. Effect estimates of the associations between 196 bacterial traits and risk of Crohn's disease in MR analyses.**

| **Exposure** | **Outcome** | **Method** | **OR (95CI%)** | ***P*-value** |
| --- | --- | --- | --- | --- |
| class.Actinobacteria.id.419 | CD | IVW | 0.888(0.718,1.099) | 0.276 |
| class.Actinobacteria.id.419 | CD | Weighted median | 0.904(0.698,1.171) | 0.446 |
| class.Actinobacteria.id.419 | CD | Weighted mode | 1.021(0.706,1.476) | 0.913 |
| class.Actinobacteria.id.419 | CD | MR-Robust | 0.928(0.754,1.142) | 0.479 |
| class.Actinobacteria.id.419 | CD | MR-Egger | 0.998(0.494,2.018) | 0.996 |
| class.Actinobacteria.id.419 | CD | MRRAPS | 0.907(0.74,1.112) | 0.347 |
| class.Actinobacteria.id.419 | CD | MRPRESSO | 0.888(0.718,1.099) | 0.288 |
| class.Alphaproteobacteria.id.2379 | CD | IVW | 1.032(0.792,1.344) | 0.816 |
| class.Alphaproteobacteria.id.2379 | CD | Weighted median | 1.05(0.751,1.468) | 0.775 |
| class.Alphaproteobacteria.id.2379 | CD | Weighted mode | 1.121(0.674,1.862) | 0.660 |
| class.Alphaproteobacteria.id.2379 | CD | MR-Robust | 1.032(0.83,1.285) | 0.775 |
| class.Alphaproteobacteria.id.2379 | CD | MR-Egger | 1.13(0.412,3.095) | 0.812 |
| class.Alphaproteobacteria.id.2379 | CD | MRRAPS | 1.032(0.773,1.379) | 0.829 |
| class.Alphaproteobacteria.id.2379 | CD | MRPRESSO | 1.032(0.88,1.21) | 0.712 |
| class.Bacilli.id.1673 | CD | IVW | 0.994(0.8,1.233) | 0.953 |
| class.Bacilli.id.1673 | CD | Weighted median | 1.047(0.771,1.422) | 0.769 |
| class.Bacilli.id.1673 | CD | Weighted mode | 1.137(0.703,1.838) | 0.600 |
| class.Bacilli.id.1673 | CD | MR-Robust | 0.995(0.802,1.235) | 0.966 |
| class.Bacilli.id.1673 | CD | MR-Egger | 0.76(0.422,1.368) | 0.360 |
| class.Bacilli.id.1673 | CD | MRRAPS | 0.989(0.782,1.251) | 0.928 |
| class.Bacilli.id.1673 | CD | MRPRESSO | 0.994(0.8,1.233) | 0.954 |
| class.Bacteroidia.id.912 | CD | IVW | 1.058(0.834,1.341) | 0.643 |
| class.Bacteroidia.id.912 | CD | Weighted median | 1.097(0.789,1.524) | 0.582 |
| class.Bacteroidia.id.912 | CD | Weighted mode | 1.11(0.718,1.718) | 0.638 |
| class.Bacteroidia.id.912 | CD | MR-Robust | 1.018(0.814,1.274) | 0.873 |
| class.Bacteroidia.id.912 | CD | MR-Egger | 1.257(0.747,2.114) | 0.389 |
| class.Bacteroidia.id.912 | CD | MRRAPS | 1.02(0.789,1.318) | 0.880 |
| class.Bacteroidia.id.912 | CD | MRPRESSO | 1.058(0.84,1.331) | 0.641 |
| class.Betaproteobacteria.id.2867 | CD | IVW | 0.796(0.605,1.049) | 0.105 |
| class.Betaproteobacteria.id.2867 | CD | Weighted median | 0.928(0.651,1.323) | 0.680 |
| class.Betaproteobacteria.id.2867 | CD | Weighted mode | 1.094(0.599,1.996) | 0.771 |
| class.Betaproteobacteria.id.2867 | CD | MR-Robust | 0.833(0.534,1.299) | 0.420 |
| class.Betaproteobacteria.id.2867 | CD | MR-Egger | 0.4(0.155,1.034) | 0.059 |
| class.Betaproteobacteria.id.2867 | CD | MRRAPS | 0.805(0.597,1.085) | 0.154 |
| class.Betaproteobacteria.id.2867 | CD | MRPRESSO | 0.796(0.605,1.049) | 0.133 |
| class.Clostridia.id.1859 | CD | IVW | 1.019(0.774,1.342) | 0.892 |
| class.Clostridia.id.1859 | CD | Weighted median | 0.982(0.686,1.406) | 0.922 |
| class.Clostridia.id.1859 | CD | Weighted mode | 1.034(0.593,1.804) | 0.905 |
| class.Clostridia.id.1859 | CD | MR-Robust | 0.979(0.758,1.264) | 0.869 |
| class.Clostridia.id.1859 | CD | MR-Egger | 0.557(0.132,2.344) | 0.425 |
| class.Clostridia.id.1859 | CD | MRRAPS | 0.987(0.733,1.331) | 0.934 |
| class.Clostridia.id.1859 | CD | MRPRESSO | 1.019(0.799,1.3) | 0.881 |
| class.Coriobacteriia.id.809 | CD | IVW | 0.961(0.765,1.208) | 0.736 |
| class.Coriobacteriia.id.809 | CD | Weighted median | 1.04(0.76,1.423) | 0.808 |
| class.Coriobacteriia.id.809 | CD | Weighted mode | 1.146(0.636,2.065) | 0.649 |
| class.Coriobacteriia.id.809 | CD | MR-Robust | 0.965(0.777,1.198) | 0.745 |
| class.Coriobacteriia.id.809 | CD | MR-Egger | 0.827(0.328,2.086) | 0.688 |
| class.Coriobacteriia.id.809 | CD | MRRAPS | 0.968(0.755,1.24) | 0.795 |
| class.Coriobacteriia.id.809 | CD | MRPRESSO | 0.961(0.78,1.186) | 0.718 |
| class.Deltaproteobacteria.id.3087 | CD | IVW | 1.111(0.865,1.426) | 0.410 |
| class.Deltaproteobacteria.id.3087 | CD | Weighted median | 1.242(0.909,1.697) | 0.174 |
| class.Deltaproteobacteria.id.3087 | CD | Weighted mode | 1.232(0.799,1.899) | 0.344 |
| class.Deltaproteobacteria.id.3087 | CD | MR-Robust | 1.104(0.892,1.367) | 0.362 |
| class.Deltaproteobacteria.id.3087 | CD | MR-Egger | 1.143(0.549,2.38) | 0.721 |
| class.Deltaproteobacteria.id.3087 | CD | MRRAPS | 1.105(0.871,1.402) | 0.412 |
| class.Deltaproteobacteria.id.3087 | CD | MRPRESSO | 1.111(0.865,1.426) | 0.426 |
| class.Erysipelotrichia.id.2147 | CD | IVW | 1.044(0.802,1.36) | 0.747 |
| class.Erysipelotrichia.id.2147 | CD | Weighted median | 1.169(0.816,1.673) | 0.395 |
| class.Erysipelotrichia.id.2147 | CD | Weighted mode | 1.196(0.675,2.12) | 0.540 |
| class.Erysipelotrichia.id.2147 | CD | MR-Robust | 1.135(0.699,1.844) | 0.609 |
| class.Erysipelotrichia.id.2147 | CD | MR-Egger | 2.271(0.701,7.351) | 0.171 |
| class.Erysipelotrichia.id.2147 | CD | MRRAPS | 1.066(0.802,1.416) | 0.660 |
| class.Erysipelotrichia.id.2147 | CD | MRPRESSO | 1.044(0.803,1.359) | 0.752 |
| class.Gammaproteobacteria.id.3303 | CD | IVW | 0.938(0.685,1.284) | 0.691 |
| class.Gammaproteobacteria.id.3303 | CD | Weighted median | 0.852(0.567,1.278) | 0.439 |
| class.Gammaproteobacteria.id.3303 | CD | Weighted mode | 0.815(0.448,1.484) | 0.504 |
| class.Gammaproteobacteria.id.3303 | CD | MR-Robust | 0.897(0.578,1.39) | 0.626 |
| class.Gammaproteobacteria.id.3303 | CD | MR-Egger | 1.399(0.468,4.178) | 0.548 |
| class.Gammaproteobacteria.id.3303 | CD | MRRAPS | 0.916(0.651,1.29) | 0.617 |
| class.Gammaproteobacteria.id.3303 | CD | MRPRESSO | 0.938(0.719,1.224) | 0.655 |
| class.Lentisphaeria.id.2250 | CD | IVW | 0.994(0.84,1.176) | 0.942 |
| class.Lentisphaeria.id.2250 | CD | Weighted median | 0.99(0.799,1.228) | 0.930 |
| class.Lentisphaeria.id.2250 | CD | Weighted mode | 0.949(0.686,1.314) | 0.754 |
| class.Lentisphaeria.id.2250 | CD | MR-Robust | 0.985(0.841,1.152) | 0.848 |
| class.Lentisphaeria.id.2250 | CD | MR-Egger | 1.065(0.59,1.92) | 0.835 |
| class.Lentisphaeria.id.2250 | CD | MRRAPS | 0.987(0.822,1.185) | 0.885 |
| class.Lentisphaeria.id.2250 | CD | MRPRESSO | 0.994(0.861,1.146) | 0.934 |
| class.Melainabacteria.id.1589 | CD | IVW | 0.93(0.792,1.093) | 0.379 |
| class.Melainabacteria.id.1589 | CD | Weighted median | 0.917(0.744,1.129) | 0.414 |
| class.Melainabacteria.id.1589 | CD | Weighted mode | 0.927(0.675,1.272) | 0.638 |
| class.Melainabacteria.id.1589 | CD | MR-Robust | 0.938(0.805,1.091) | 0.406 |
| class.Melainabacteria.id.1589 | CD | MR-Egger | 0.817(0.514,1.299) | 0.393 |
| class.Melainabacteria.id.1589 | CD | MRRAPS | 0.931(0.782,1.11) | 0.428 |
| class.Melainabacteria.id.1589 | CD | MRPRESSO | 0.93(0.828,1.046) | 0.257 |
| class.Methanobacteria.id.119 | CD | IVW | 0.917(0.763,1.103) | 0.357 |
| class.Methanobacteria.id.119 | CD | Weighted median | 0.96(0.792,1.164) | 0.677 |
| class.Methanobacteria.id.119 | CD | Weighted mode | 1.019(0.737,1.409) | 0.908 |
| class.Methanobacteria.id.119 | CD | MR-Robust | 0.919(0.767,1.102) | 0.363 |
| class.Methanobacteria.id.119 | CD | MR-Egger | 1.255(0.574,2.742) | 0.569 |
| class.Methanobacteria.id.119 | CD | MRRAPS | 0.925(0.77,1.109) | 0.399 |
| class.Methanobacteria.id.119 | CD | MRPRESSO | 0.917(0.763,1.103) | 0.378 |
| class.Mollicutes.id.3920 | CD | IVW | 1.028(0.83,1.274) | 0.801 |
| class.Mollicutes.id.3920 | CD | Weighted median | 1.073(0.798,1.442) | 0.643 |
| class.Mollicutes.id.3920 | CD | Weighted mode | 1.224(0.781,1.92) | 0.378 |
| class.Mollicutes.id.3920 | CD | MR-Robust | 1.031(0.839,1.267) | 0.772 |
| class.Mollicutes.id.3920 | CD | MR-Egger | 1.28(0.635,2.581) | 0.490 |
| class.Mollicutes.id.3920 | CD | MRRAPS | 1.035(0.824,1.3) | 0.765 |
| class.Mollicutes.id.3920 | CD | MRPRESSO | 1.028(0.83,1.274) | 0.805 |
| class.Negativicutes.id.2164 | CD | IVW | 0.899(0.678,1.191) | 0.458 |
| class.Negativicutes.id.2164 | CD | Weighted median | 0.987(0.676,1.443) | 0.947 |
| class.Negativicutes.id.2164 | CD | Weighted mode | 1.162(0.595,2.269) | 0.660 |
| class.Negativicutes.id.2164 | CD | MR-Robust | 0.913(0.656,1.271) | 0.589 |
| class.Negativicutes.id.2164 | CD | MR-Egger | 0.875(0.334,2.296) | 0.787 |
| class.Negativicutes.id.2164 | CD | MRRAPS | 0.908(0.667,1.235) | 0.537 |
| class.Negativicutes.id.2164 | CD | MRPRESSO | 0.899(0.678,1.191) | 0.474 |
| class.Verrucomicrobiae.id.4029 | CD | IVW | 0.915(0.721,1.161) | 0.464 |
| class.Verrucomicrobiae.id.4029 | CD | Weighted median | 0.923(0.689,1.237) | 0.591 |
| class.Verrucomicrobiae.id.4029 | CD | Weighted mode | 0.908(0.581,1.421) | 0.674 |
| class.Verrucomicrobiae.id.4029 | CD | MR-Robust | 0.895(0.691,1.159) | 0.399 |
| class.Verrucomicrobiae.id.4029 | CD | MR-Egger | 0.803(0.314,2.053) | 0.647 |
| class.Verrucomicrobiae.id.4029 | CD | MRRAPS | 0.904(0.719,1.135) | 0.384 |
| class.Verrucomicrobiae.id.4029 | CD | MRPRESSO | 0.915(0.721,1.161) | 0.479 |
| family.Acidaminococcaceae.id.2166 | CD | IVW | 1.197(0.872,1.642) | 0.266 |
| family.Acidaminococcaceae.id.2166 | CD | Weighted median | 1.221(0.821,1.815) | 0.325 |
| family.Acidaminococcaceae.id.2166 | CD | Weighted mode | 1.375(0.742,2.547) | 0.312 |
| family.Acidaminococcaceae.id.2166 | CD | MR-Robust | 1.208(0.881,1.656) | 0.240 |
| family.Acidaminococcaceae.id.2166 | CD | MR-Egger | 1.574(0.575,4.309) | 0.377 |
| family.Acidaminococcaceae.id.2166 | CD | MRRAPS | 1.242(0.902,1.711) | 0.185 |
| family.Acidaminococcaceae.id.2166 | CD | MRPRESSO | 1.197(0.872,1.642) | 0.308 |
| family.Actinomycetaceae.id.421 | CD | IVW | 0.952(0.729,1.243) | 0.717 |
| family.Actinomycetaceae.id.421 | CD | Weighted median | 0.909(0.644,1.285) | 0.590 |
| family.Actinomycetaceae.id.421 | CD | Weighted mode | 0.892(0.568,1.398) | 0.617 |
| family.Actinomycetaceae.id.421 | CD | MR-Robust | 0.95(0.769,1.173) | 0.635 |
| family.Actinomycetaceae.id.421 | CD | MR-Egger | 0.694(0.33,1.457) | 0.334 |
| family.Actinomycetaceae.id.421 | CD | MRRAPS | 0.951(0.712,1.27) | 0.732 |
| family.Actinomycetaceae.id.421 | CD | MRPRESSO | 0.952(0.747,1.213) | 0.711 |
| family.Alcaligenaceae.id.2875 | CD | IVW | 0.987(0.781,1.247) | 0.913 |
| family.Alcaligenaceae.id.2875 | CD | Weighted median | 1.033(0.746,1.431) | 0.844 |
| family.Alcaligenaceae.id.2875 | CD | Weighted mode | 1.091(0.593,2.007) | 0.779 |
| family.Alcaligenaceae.id.2875 | CD | MR-Robust | 0.985(0.781,1.24) | 0.895 |
| family.Alcaligenaceae.id.2875 | CD | MR-Egger | 0.373(0.135,1.028) | 0.057 |
| family.Alcaligenaceae.id.2875 | CD | MRRAPS | 0.98(0.761,1.261) | 0.873 |
| family.Alcaligenaceae.id.2875 | CD | MRPRESSO | 0.987(0.794,1.227) | 0.908 |
| family.Bacteroidaceae.id.917 | CD | IVW | 1.187(0.852,1.654) | 0.310 |
| family.Bacteroidaceae.id.917 | CD | Weighted median | 1.395(0.903,2.156) | 0.134 |
| family.Bacteroidaceae.id.917 | CD | Weighted mode | 1.576(0.767,3.24) | 0.216 |
| family.Bacteroidaceae.id.917 | CD | MR-Robust | 1.241(0.685,2.25) | 0.476 |
| family.Bacteroidaceae.id.917 | CD | MR-Egger | 1.676(0.245,11.459) | 0.598 |
| family.Bacteroidaceae.id.917 | CD | MRRAPS | 1.214(0.847,1.739) | 0.291 |
| family.Bacteroidaceae.id.917 | CD | MRPRESSO | 1.187(0.878,1.606) | 0.302 |
| family.BacteroidalesS24.7group.id.11173 | CD | IVW | 1.096(0.869,1.384) | 0.438 |
| family.BacteroidalesS24.7group.id.11173 | CD | Weighted median | 1.074(0.807,1.427) | 0.625 |
| family.BacteroidalesS24.7group.id.11173 | CD | Weighted mode | 1.028(0.64,1.653) | 0.909 |
| family.BacteroidalesS24.7group.id.11173 | CD | MR-Robust | 1.1(0.87,1.391) | 0.425 |
| family.BacteroidalesS24.7group.id.11173 | CD | MR-Egger | 0.812(0.281,2.352) | 0.702 |
| family.BacteroidalesS24.7group.id.11173 | CD | MRRAPS | 1.114(0.878,1.415) | 0.374 |
| family.BacteroidalesS24.7group.id.11173 | CD | MRPRESSO | 1.096(0.869,1.384) | 0.464 |
| family.Bifidobacteriaceae.id.433 | CD | IVW | 0.882(0.733,1.061) | 0.184 |
| family.Bifidobacteriaceae.id.433 | CD | Weighted median | 0.902(0.716,1.138) | 0.385 |
| family.Bifidobacteriaceae.id.433 | CD | Weighted mode | 0.944(0.669,1.33) | 0.741 |
| family.Bifidobacteriaceae.id.433 | CD | MR-Robust | 0.868(0.741,1.016) | 0.079 |
| family.Bifidobacteriaceae.id.433 | CD | MR-Egger | 1.204(0.59,2.46) | 0.610 |
| family.Bifidobacteriaceae.id.433 | CD | MRRAPS | 0.866(0.714,1.05) | 0.142 |
| family.Bifidobacteriaceae.id.433 | CD | MRPRESSO | 0.882(0.733,1.061) | 0.196 |
| family.Christensenellaceae.id.1866 | CD | IVW | 0.882(0.691,1.125) | 0.311 |
| family.Christensenellaceae.id.1866 | CD | Weighted median | 0.909(0.659,1.254) | 0.561 |
| family.Christensenellaceae.id.1866 | CD | Weighted mode | 0.924(0.602,1.418) | 0.718 |
| family.Christensenellaceae.id.1866 | CD | MR-Robust | 0.883(0.7,1.114) | 0.293 |
| family.Christensenellaceae.id.1866 | CD | MR-Egger | 0.965(0.572,1.63) | 0.895 |
| family.Christensenellaceae.id.1866 | CD | MRRAPS | 0.876(0.671,1.144) | 0.332 |
| family.Christensenellaceae.id.1866 | CD | MRPRESSO | 0.882(0.719,1.081) | 0.254 |
| family.Clostridiaceae1.id.1869 | CD | IVW | 0.819(0.605,1.107) | 0.193 |
| family.Clostridiaceae1.id.1869 | CD | Weighted median | 0.7(0.484,1.013) | 0.059 |
| family.Clostridiaceae1.id.1869 | CD | Weighted mode | 0.735(0.418,1.293) | 0.285 |
| family.Clostridiaceae1.id.1869 | CD | MR-Robust | 0.789(0.575,1.082) | 0.141 |
| family.Clostridiaceae1.id.1869 | CD | MR-Egger | 0.884(0.331,2.361) | 0.805 |
| family.Clostridiaceae1.id.1869 | CD | MRRAPS | 0.775(0.575,1.045) | 0.094 |
| family.Clostridiaceae1.id.1869 | CD | MRPRESSO | 0.819(0.605,1.107) | 0.226 |
| family.ClostridialesvadinBB60group.id.11286 | CD | IVW | 0.951(0.771,1.172) | 0.636 |
| family.ClostridialesvadinBB60group.id.11286 | CD | Weighted median | 0.904(0.693,1.18) | 0.459 |
| family.ClostridialesvadinBB60group.id.11286 | CD | Weighted mode | 0.899(0.541,1.494) | 0.682 |
| family.ClostridialesvadinBB60group.id.11286 | CD | MR-Robust | 0.949(0.764,1.179) | 0.637 |
| family.ClostridialesvadinBB60group.id.11286 | CD | MR-Egger | 1.213(0.664,2.218) | 0.530 |
| family.ClostridialesvadinBB60group.id.11286 | CD | MRRAPS | 0.963(0.762,1.216) | 0.750 |
| family.ClostridialesvadinBB60group.id.11286 | CD | MRPRESSO | 0.951(0.771,1.172) | 0.643 |
| family.Coriobacteriaceae.id.811 | CD | IVW | 0.961(0.765,1.208) | 0.736 |
| family.Coriobacteriaceae.id.811 | CD | Weighted median | 1.04(0.76,1.423) | 0.808 |
| family.Coriobacteriaceae.id.811 | CD | Weighted mode | 1.146(0.636,2.065) | 0.649 |
| family.Coriobacteriaceae.id.811 | CD | MR-Robust | 0.965(0.777,1.198) | 0.745 |
| family.Coriobacteriaceae.id.811 | CD | MR-Egger | 0.827(0.328,2.086) | 0.688 |
| family.Coriobacteriaceae.id.811 | CD | MRRAPS | 0.968(0.755,1.24) | 0.795 |
| family.Coriobacteriaceae.id.811 | CD | MRPRESSO | 0.961(0.78,1.186) | 0.718 |
| family.Defluviitaleaceae.id.1924 | CD | IVW | 1.193(0.992,1.435) | 0.061 |
| family.Defluviitaleaceae.id.1924 | CD | Weighted median | 1.099(0.864,1.398) | 0.440 |
| family.Defluviitaleaceae.id.1924 | CD | Weighted mode | 1.08(0.739,1.576) | 0.692 |
| family.Defluviitaleaceae.id.1924 | CD | MR-Robust | 1.146(0.774,1.697) | 0.495 |
| family.Defluviitaleaceae.id.1924 | CD | MR-Egger | 1.203(0.645,2.244) | 0.562 |
| family.Defluviitaleaceae.id.1924 | CD | MRRAPS | 1.194(0.975,1.463) | 0.086 |
| family.Defluviitaleaceae.id.1924 | CD | MRPRESSO | 1.193(1.053,1.351) | 0.018 |
| family.Desulfovibrionaceae.id.3169 | CD | IVW | 1.075(0.786,1.472) | 0.650 |
| family.Desulfovibrionaceae.id.3169 | CD | Weighted median | 1.09(0.772,1.537) | 0.625 |
| family.Desulfovibrionaceae.id.3169 | CD | Weighted mode | 1.134(0.721,1.782) | 0.586 |
| family.Desulfovibrionaceae.id.3169 | CD | MR-Robust | 1.063(0.804,1.405) | 0.668 |
| family.Desulfovibrionaceae.id.3169 | CD | MR-Egger | 1.204(0.521,2.779) | 0.664 |
| family.Desulfovibrionaceae.id.3169 | CD | MRRAPS | 1.06(0.8,1.404) | 0.683 |
| family.Desulfovibrionaceae.id.3169 | CD | MRPRESSO | 1.075(0.786,1.472) | 0.661 |
| family.Enterobacteriaceae.id.3469 | CD | IVW | 1.085(0.749,1.57) | 0.667 |
| family.Enterobacteriaceae.id.3469 | CD | Weighted median | 1.014(0.673,1.529) | 0.947 |
| family.Enterobacteriaceae.id.3469 | CD | Weighted mode | 0.836(0.403,1.735) | 0.631 |
| family.Enterobacteriaceae.id.3469 | CD | MR-Robust | 1.08(0.74,1.576) | 0.690 |
| family.Enterobacteriaceae.id.3469 | CD | MR-Egger | 0.805(0.063,10.339) | 0.868 |
| family.Enterobacteriaceae.id.3469 | CD | MRRAPS | 1.083(0.715,1.642) | 0.706 |
| family.Enterobacteriaceae.id.3469 | CD | MRPRESSO | 1.085(0.749,1.57) | 0.680 |
| family.Erysipelotrichaceae.id.2149 | CD | IVW | 1.044(0.802,1.36) | 0.747 |
| family.Erysipelotrichaceae.id.2149 | CD | Weighted median | 1.169(0.816,1.673) | 0.395 |
| family.Erysipelotrichaceae.id.2149 | CD | Weighted mode | 1.196(0.675,2.12) | 0.540 |
| family.Erysipelotrichaceae.id.2149 | CD | MR-Robust | 1.135(0.699,1.844) | 0.609 |
| family.Erysipelotrichaceae.id.2149 | CD | MR-Egger | 2.271(0.701,7.351) | 0.171 |
| family.Erysipelotrichaceae.id.2149 | CD | MRRAPS | 1.066(0.802,1.416) | 0.660 |
| family.Erysipelotrichaceae.id.2149 | CD | MRPRESSO | 1.044(0.803,1.359) | 0.752 |
| family.FamilyXI.id.1936 | CD | IVW | 0.943(0.824,1.079) | 0.394 |
| family.FamilyXI.id.1936 | CD | Weighted median | 0.99(0.835,1.174) | 0.905 |
| family.FamilyXI.id.1936 | CD | Weighted mode | 1(0.765,1.307) | 0.998 |
| family.FamilyXI.id.1936 | CD | MR-Robust | 0.947(0.825,1.088) | 0.444 |
| family.FamilyXI.id.1936 | CD | MR-Egger | 0.577(0.239,1.393) | 0.221 |
| family.FamilyXI.id.1936 | CD | MRRAPS | 0.942(0.813,1.091) | 0.425 |
| family.FamilyXI.id.1936 | CD | MRPRESSO | 0.943(0.855,1.04) | 0.277 |
| family.FamilyXIII.id.1957 | CD | IVW | 0.984(0.664,1.459) | 0.937 |
| family.FamilyXIII.id.1957 | CD | Weighted median | 1.164(0.759,1.784) | 0.488 |
| family.FamilyXIII.id.1957 | CD | Weighted mode | 1.36(0.654,2.831) | 0.411 |
| family.FamilyXIII.id.1957 | CD | MR-Robust | 1(0.666,1.501) | 0.998 |
| family.FamilyXIII.id.1957 | CD | MR-Egger | 0.372(0.069,2.014) | 0.251 |
| family.FamilyXIII.id.1957 | CD | MRRAPS | 0.931(0.596,1.453) | 0.753 |
| family.FamilyXIII.id.1957 | CD | MRPRESSO | 0.984(0.664,1.459) | 0.938 |
| family.Lachnospiraceae.id.1987 | CD | IVW | 1.128(0.913,1.395) | 0.264 |
| family.Lachnospiraceae.id.1987 | CD | Weighted median | 1.072(0.8,1.437) | 0.641 |
| family.Lachnospiraceae.id.1987 | CD | Weighted mode | 0.904(0.559,1.461) | 0.680 |
| family.Lachnospiraceae.id.1987 | CD | MR-Robust | 1.132(0.944,1.358) | 0.179 |
| family.Lachnospiraceae.id.1987 | CD | MR-Egger | 1.085(0.638,1.846) | 0.763 |
| family.Lachnospiraceae.id.1987 | CD | MRRAPS | 1.134(0.9,1.429) | 0.287 |
| family.Lachnospiraceae.id.1987 | CD | MRPRESSO | 1.128(0.942,1.352) | 0.208 |
| family.Lactobacillaceae.id.1836 | CD | IVW | 0.944(0.785,1.135) | 0.539 |
| family.Lactobacillaceae.id.1836 | CD | Weighted median | 0.867(0.682,1.103) | 0.245 |
| family.Lactobacillaceae.id.1836 | CD | Weighted mode | 0.849(0.587,1.228) | 0.384 |
| family.Lactobacillaceae.id.1836 | CD | MR-Robust | 0.888(0.476,1.658) | 0.710 |
| family.Lactobacillaceae.id.1836 | CD | MR-Egger | 0.95(0.57,1.584) | 0.845 |
| family.Lactobacillaceae.id.1836 | CD | MRRAPS | 0.934(0.764,1.141) | 0.502 |
| family.Lactobacillaceae.id.1836 | CD | MRPRESSO | 0.944(0.808,1.103) | 0.486 |
| family.Methanobacteriaceae.id.121 | CD | IVW | 0.917(0.763,1.103) | 0.357 |
| family.Methanobacteriaceae.id.121 | CD | Weighted median | 0.96(0.792,1.164) | 0.677 |
| family.Methanobacteriaceae.id.121 | CD | Weighted mode | 1.019(0.737,1.409) | 0.908 |
| family.Methanobacteriaceae.id.121 | CD | MR-Robust | 0.919(0.767,1.102) | 0.363 |
| family.Methanobacteriaceae.id.121 | CD | MR-Egger | 1.255(0.574,2.742) | 0.569 |
| family.Methanobacteriaceae.id.121 | CD | MRRAPS | 0.925(0.77,1.109) | 0.399 |
| family.Methanobacteriaceae.id.121 | CD | MRPRESSO | 0.917(0.763,1.103) | 0.378 |
| family.Oxalobacteraceae.id.2966 | CD | IVW | 1.117(0.936,1.334) | 0.220 |
| family.Oxalobacteraceae.id.2966 | CD | Weighted median | 1.196(0.987,1.449) | 0.068 |
| family.Oxalobacteraceae.id.2966 | CD | Weighted mode | 1.246(0.925,1.68) | 0.148 |
| family.Oxalobacteraceae.id.2966 | CD | MR-Robust | 1.138(0.928,1.395) | 0.216 |
| family.Oxalobacteraceae.id.2966 | CD | MR-Egger | 0.886(0.426,1.841) | 0.745 |
| family.Oxalobacteraceae.id.2966 | CD | MRRAPS | 1.121(0.948,1.324) | 0.182 |
| family.Oxalobacteraceae.id.2966 | CD | MRPRESSO | 1.117(0.936,1.334) | 0.241 |
| family.Pasteurellaceae.id.3689 | CD | IVW | 0.979(0.827,1.158) | 0.804 |
| family.Pasteurellaceae.id.3689 | CD | Weighted median | 1.111(0.883,1.399) | 0.369 |
| family.Pasteurellaceae.id.3689 | CD | Weighted mode | 1.213(0.818,1.798) | 0.336 |
| family.Pasteurellaceae.id.3689 | CD | MR-Robust | 0.979(0.815,1.176) | 0.822 |
| family.Pasteurellaceae.id.3689 | CD | MR-Egger | 1.098(0.742,1.624) | 0.640 |
| family.Pasteurellaceae.id.3689 | CD | MRRAPS | 0.973(0.793,1.193) | 0.791 |
| family.Pasteurellaceae.id.3689 | CD | MRPRESSO | 0.979(0.827,1.158) | 0.807 |
| family.Peptococcaceae.id.2024 | CD | IVW | 1.04(0.835,1.296) | 0.727 |
| family.Peptococcaceae.id.2024 | CD | Weighted median | 0.933(0.707,1.231) | 0.625 |
| family.Peptococcaceae.id.2024 | CD | Weighted mode | 0.898(0.644,1.253) | 0.527 |
| family.Peptococcaceae.id.2024 | CD | MR-Robust | 0.963(0.715,1.298) | 0.805 |
| family.Peptococcaceae.id.2024 | CD | MR-Egger | 1.143(0.657,1.99) | 0.636 |
| family.Peptococcaceae.id.2024 | CD | MRRAPS | 0.996(0.803,1.235) | 0.972 |
| family.Peptococcaceae.id.2024 | CD | MRPRESSO | 1.04(0.835,1.296) | 0.735 |
| family.Peptostreptococcaceae.id.2042 | CD | IVW | 1.009(0.812,1.253) | 0.939 |
| family.Peptostreptococcaceae.id.2042 | CD | Weighted median | 0.982(0.729,1.323) | 0.906 |
| family.Peptostreptococcaceae.id.2042 | CD | Weighted mode | 0.935(0.593,1.473) | 0.771 |
| family.Peptostreptococcaceae.id.2042 | CD | MR-Robust | 0.996(0.786,1.261) | 0.972 |
| family.Peptostreptococcaceae.id.2042 | CD | MR-Egger | 0.872(0.519,1.466) | 0.605 |
| family.Peptostreptococcaceae.id.2042 | CD | MRRAPS | 0.981(0.773,1.244) | 0.873 |
| family.Peptostreptococcaceae.id.2042 | CD | MRPRESSO | 1.009(0.812,1.253) | 0.940 |
| family.Porphyromonadaceae.id.943 | CD | IVW | 0.935(0.652,1.34) | 0.715 |
| family.Porphyromonadaceae.id.943 | CD | Weighted median | 0.919(0.607,1.393) | 0.692 |
| family.Porphyromonadaceae.id.943 | CD | Weighted mode | 0.996(0.493,2.013) | 0.992 |
| family.Porphyromonadaceae.id.943 | CD | MR-Robust | 0.938(0.66,1.332) | 0.719 |
| family.Porphyromonadaceae.id.943 | CD | MR-Egger | 1.511(0.361,6.325) | 0.572 |
| family.Porphyromonadaceae.id.943 | CD | MRRAPS | 0.958(0.663,1.385) | 0.819 |
| family.Porphyromonadaceae.id.943 | CD | MRPRESSO | 0.935(0.652,1.34) | 0.723 |
| family.Prevotellaceae.id.960 | CD | IVW | 0.8(0.642,0.998) | 0.048 |
| family.Prevotellaceae.id.960 | CD | Weighted median | 0.859(0.644,1.146) | 0.302 |
| family.Prevotellaceae.id.960 | CD | Weighted mode | 1.008(0.639,1.589) | 0.973 |
| family.Prevotellaceae.id.960 | CD | MR-Robust | 0.841(0.637,1.11) | 0.221 |
| family.Prevotellaceae.id.960 | CD | MR-Egger | 1.076(0.491,2.362) | 0.854 |
| family.Prevotellaceae.id.960 | CD | MRRAPS | 0.835(0.667,1.045) | 0.114 |
| family.Prevotellaceae.id.960 | CD | MRPRESSO | 0.8(0.642,0.998) | 0.067 |
| family.Rhodospirillaceae.id.2717 | CD | IVW | 1.017(0.859,1.204) | 0.846 |
| family.Rhodospirillaceae.id.2717 | CD | Weighted median | 0.983(0.793,1.219) | 0.878 |
| family.Rhodospirillaceae.id.2717 | CD | Weighted mode | 1.222(0.843,1.771) | 0.290 |
| family.Rhodospirillaceae.id.2717 | CD | MR-Robust | 1.017(0.89,1.163) | 0.803 |
| family.Rhodospirillaceae.id.2717 | CD | MR-Egger | 1.07(0.408,2.805) | 0.891 |
| family.Rhodospirillaceae.id.2717 | CD | MRRAPS | 1.017(0.845,1.224) | 0.857 |
| family.Rhodospirillaceae.id.2717 | CD | MRPRESSO | 1.017(0.922,1.122) | 0.744 |
| family.Rikenellaceae.id.967 | CD | IVW | 1.037(0.852,1.262) | 0.717 |
| family.Rikenellaceae.id.967 | CD | Weighted median | 1.095(0.836,1.434) | 0.509 |
| family.Rikenellaceae.id.967 | CD | Weighted mode | 1.172(0.726,1.89) | 0.516 |
| family.Rikenellaceae.id.967 | CD | MR-Robust | 1.059(0.846,1.325) | 0.617 |
| family.Rikenellaceae.id.967 | CD | MR-Egger | 1.132(0.606,2.113) | 0.698 |
| family.Rikenellaceae.id.967 | CD | MRRAPS | 1.048(0.847,1.297) | 0.664 |
| family.Rikenellaceae.id.967 | CD | MRPRESSO | 1.037(0.878,1.225) | 0.674 |
| family.Ruminococcaceae.id.2050 | CD | IVW | 0.8(0.613,1.044) | 0.100 |
| family.Ruminococcaceae.id.2050 | CD | Weighted median | 0.777(0.537,1.124) | 0.181 |
| family.Ruminococcaceae.id.2050 | CD | Weighted mode | 0.743(0.439,1.259) | 0.270 |
| family.Ruminococcaceae.id.2050 | CD | MR-Robust | 0.804(0.619,1.044) | 0.102 |
| family.Ruminococcaceae.id.2050 | CD | MR-Egger | 0.609(0.346,1.072) | 0.086 |
| family.Ruminococcaceae.id.2050 | CD | MRRAPS | 0.806(0.607,1.071) | 0.137 |
| family.Ruminococcaceae.id.2050 | CD | MRPRESSO | 0.8(0.613,1.044) | 0.135 |
| family.Streptococcaceae.id.1850 | CD | IVW | 1.051(0.843,1.311) | 0.659 |
| family.Streptococcaceae.id.1850 | CD | Weighted median | 1.192(0.881,1.613) | 0.255 |
| family.Streptococcaceae.id.1850 | CD | Weighted mode | 1.401(0.798,2.46) | 0.241 |
| family.Streptococcaceae.id.1850 | CD | MR-Robust | 1.059(0.836,1.34) | 0.636 |
| family.Streptococcaceae.id.1850 | CD | MR-Egger | 1.044(0.441,2.473) | 0.922 |
| family.Streptococcaceae.id.1850 | CD | MRRAPS | 1.068(0.834,1.366) | 0.603 |
| family.Streptococcaceae.id.1850 | CD | MRPRESSO | 1.051(0.843,1.311) | 0.664 |
| family.unknownfamily.id.1000001214 | CD | IVW | 0.947(0.801,1.12) | 0.522 |
| family.unknownfamily.id.1000001214 | CD | Weighted median | 0.935(0.747,1.17) | 0.559 |
| family.unknownfamily.id.1000001214 | CD | Weighted mode | 0.938(0.676,1.301) | 0.703 |
| family.unknownfamily.id.1000001214 | CD | MR-Robust | 0.946(0.804,1.114) | 0.508 |
| family.unknownfamily.id.1000001214 | CD | MR-Egger | 0.778(0.482,1.254) | 0.303 |
| family.unknownfamily.id.1000001214 | CD | MRRAPS | 0.944(0.787,1.132) | 0.532 |
| family.unknownfamily.id.1000001214 | CD | MRPRESSO | 0.947(0.818,1.096) | 0.484 |
| family.unknownfamily.id.1000005471 | CD | IVW | 1.054(0.874,1.27) | 0.584 |
| family.unknownfamily.id.1000005471 | CD | Weighted median | 1.001(0.773,1.296) | 0.993 |
| family.unknownfamily.id.1000005471 | CD | Weighted mode | 0.896(0.615,1.307) | 0.570 |
| family.unknownfamily.id.1000005471 | CD | MR-Robust | 1.03(0.844,1.256) | 0.773 |
| family.unknownfamily.id.1000005471 | CD | MR-Egger | 0.939(0.537,1.643) | 0.826 |
| family.unknownfamily.id.1000005471 | CD | MRRAPS | 1.03(0.845,1.256) | 0.770 |
| family.unknownfamily.id.1000005471 | CD | MRPRESSO | 1.054(0.874,1.27) | 0.593 |
| family.unknownfamily.id.1000006161 | CD | IVW | 1.152(1.007,1.318) | 0.039 |
| family.unknownfamily.id.1000006161 | CD | Weighted median | 1.162(0.965,1.4) | 0.113 |
| family.unknownfamily.id.1000006161 | CD | Weighted mode | 0.905(0.634,1.293) | 0.585 |
| family.unknownfamily.id.1000006161 | CD | MR-Robust | 1.149(0.997,1.325) | 0.055 |
| family.unknownfamily.id.1000006161 | CD | MR-Egger | 1.341(0.763,2.358) | 0.308 |
| family.unknownfamily.id.1000006161 | CD | MRRAPS | 1.151(0.99,1.338) | 0.068 |
| family.unknownfamily.id.1000006161 | CD | MRPRESSO | 1.152(1.007,1.318) | 0.059 |
| family.Veillonellaceae.id.2172 | CD | IVW | 1.043(0.855,1.272) | 0.676 |
| family.Veillonellaceae.id.2172 | CD | Weighted median | 1.173(0.909,1.515) | 0.220 |
| family.Veillonellaceae.id.2172 | CD | Weighted mode | 1.165(0.821,1.654) | 0.392 |
| family.Veillonellaceae.id.2172 | CD | MR-Robust | 1.08(0.884,1.319) | 0.452 |
| family.Veillonellaceae.id.2172 | CD | MR-Egger | 1.034(0.659,1.624) | 0.883 |
| family.Veillonellaceae.id.2172 | CD | MRRAPS | 1.09(0.898,1.322) | 0.383 |
| family.Veillonellaceae.id.2172 | CD | MRPRESSO | 1.043(0.855,1.272) | 0.681 |
| family.Verrucomicrobiaceae.id.4036 | CD | IVW | 0.915(0.721,1.161) | 0.464 |
| family.Verrucomicrobiaceae.id.4036 | CD | Weighted median | 0.923(0.689,1.236) | 0.589 |
| family.Verrucomicrobiaceae.id.4036 | CD | Weighted mode | 0.909(0.58,1.424) | 0.676 |
| family.Verrucomicrobiaceae.id.4036 | CD | MR-Robust | 0.894(0.69,1.159) | 0.399 |
| family.Verrucomicrobiaceae.id.4036 | CD | MR-Egger | 0.802(0.314,2.051) | 0.645 |
| family.Verrucomicrobiaceae.id.4036 | CD | MRRAPS | 0.904(0.719,1.135) | 0.384 |
| family.Verrucomicrobiaceae.id.4036 | CD | MRPRESSO | 0.915(0.721,1.161) | 0.479 |
| family.Victivallaceae.id.2255 | CD | IVW | 0.954(0.847,1.074) | 0.437 |
| family.Victivallaceae.id.2255 | CD | Weighted median | 0.943(0.813,1.094) | 0.440 |
| family.Victivallaceae.id.2255 | CD | Weighted mode | 0.957(0.755,1.212) | 0.714 |
| family.Victivallaceae.id.2255 | CD | MR-Robust | 0.957(0.859,1.066) | 0.428 |
| family.Victivallaceae.id.2255 | CD | MR-Egger | 0.868(0.499,1.509) | 0.616 |
| family.Victivallaceae.id.2255 | CD | MRRAPS | 0.954(0.838,1.086) | 0.474 |
| family.Victivallaceae.id.2255 | CD | MRPRESSO | 0.954(0.903,1.008) | 0.118 |
| genus Clostridiuminnocuumgroup.id.14397 | CD | IVW | 0.883(0.759,1.027) | 0.107 |
| genus Clostridiuminnocuumgroup.id.14397 | CD | Weighted median | 0.887(0.728,1.081) | 0.236 |
| genus Clostridiuminnocuumgroup.id.14397 | CD | Weighted mode | 0.893(0.666,1.198) | 0.450 |
| genus Clostridiuminnocuumgroup.id.14397 | CD | MR-Robust | 0.89(0.771,1.028) | 0.114 |
| genus Clostridiuminnocuumgroup.id.14397 | CD | MR-Egger | 1.601(0.742,3.457) | 0.230 |
| genus Clostridiuminnocuumgroup.id.14397 | CD | MRRAPS | 0.883(0.748,1.043) | 0.144 |
| genus Clostridiuminnocuumgroup.id.14397 | CD | MRPRESSO | 0.883(0.788,0.989) | 0.064 |
| genus Eubacteriumbrachygroup.id.11296 | CD | IVW | 0.928(0.798,1.079) | 0.330 |
| genus Eubacteriumbrachygroup.id.11296 | CD | Weighted median | 1.003(0.815,1.236) | 0.974 |
| genus Eubacteriumbrachygroup.id.11296 | CD | Weighted mode | 1.045(0.768,1.421) | 0.780 |
| genus Eubacteriumbrachygroup.id.11296 | CD | MR-Robust | 0.955(0.762,1.197) | 0.691 |
| genus Eubacteriumbrachygroup.id.11296 | CD | MR-Egger | 0.558(0.286,1.089) | 0.087 |
| genus Eubacteriumbrachygroup.id.11296 | CD | MRRAPS | 0.944(0.802,1.112) | 0.492 |
| genus Eubacteriumbrachygroup.id.11296 | CD | MRPRESSO | 0.928(0.798,1.078) | 0.356 |
| genus Eubacteriumcoprostanoligenesgroup.id.11375 | CD | IVW | 1.007(0.783,1.295) | 0.956 |
| genus Eubacteriumcoprostanoligenesgroup.id.11375 | CD | Weighted median | 1.03(0.734,1.445) | 0.864 |
| genus Eubacteriumcoprostanoligenesgroup.id.11375 | CD | Weighted mode | 1.204(0.666,2.177) | 0.539 |
| genus Eubacteriumcoprostanoligenesgroup.id.11375 | CD | MR-Robust | 1.006(0.799,1.268) | 0.957 |
| genus Eubacteriumcoprostanoligenesgroup.id.11375 | CD | MR-Egger | 1.318(0.49,3.549) | 0.584 |
| genus Eubacteriumcoprostanoligenesgroup.id.11375 | CD | MRRAPS | 1.004(0.765,1.319) | 0.975 |
| genus Eubacteriumcoprostanoligenesgroup.id.11375 | CD | MRPRESSO | 1.007(0.8,1.267) | 0.953 |
| genus Eubacteriumeligensgroup.id.14372 | CD | IVW | 1.257(0.909,1.738) | 0.166 |
| genus Eubacteriumeligensgroup.id.14372 | CD | Weighted median | 1.158(0.761,1.763) | 0.494 |
| genus Eubacteriumeligensgroup.id.14372 | CD | Weighted mode | 0.936(0.502,1.746) | 0.836 |
| genus Eubacteriumeligensgroup.id.14372 | CD | MR-Robust | 1.175(0.771,1.791) | 0.452 |
| genus Eubacteriumeligensgroup.id.14372 | CD | MR-Egger | 1.034(0.275,3.894) | 0.960 |
| genus Eubacteriumeligensgroup.id.14372 | CD | MRRAPS | 1.197(0.852,1.682) | 0.299 |
| genus Eubacteriumeligensgroup.id.14372 | CD | MRPRESSO | 1.257(0.909,1.738) | 0.209 |
| genus Eubacteriumfissicatenagroup.id.14373 | CD | IVW | 1.02(0.88,1.183) | 0.789 |
| genus Eubacteriumfissicatenagroup.id.14373 | CD | Weighted median | 0.965(0.795,1.171) | 0.715 |
| genus Eubacteriumfissicatenagroup.id.14373 | CD | Weighted mode | 0.943(0.728,1.223) | 0.659 |
| genus Eubacteriumfissicatenagroup.id.14373 | CD | MR-Robust | 0.952(0.848,1.069) | 0.409 |
| genus Eubacteriumfissicatenagroup.id.14373 | CD | MR-Egger | 1.808(0.823,3.974) | 0.140 |
| genus Eubacteriumfissicatenagroup.id.14373 | CD | MRRAPS | 0.989(0.844,1.159) | 0.888 |
| genus Eubacteriumfissicatenagroup.id.14373 | CD | MRPRESSO | 1.02(0.88,1.183) | 0.796 |
| genus Eubacteriumhalliigroup.id.11338 | CD | IVW | 0.698(0.485,1.007) | 0.054 |
| genus Eubacteriumhalliigroup.id.11338 | CD | Weighted median | 0.839(0.611,1.152) | 0.277 |
| genus Eubacteriumhalliigroup.id.11338 | CD | Weighted mode | 0.84(0.534,1.32) | 0.449 |
| genus Eubacteriumhalliigroup.id.11338 | CD | MR-Robust | 0.785(0.616,1.002) | 0.052 |
| genus Eubacteriumhalliigroup.id.11338 | CD | MR-Egger | 1.25(0.576,2.716) | 0.572 |
| genus Eubacteriumhalliigroup.id.11338 | CD | MRRAPS | 0.779(0.592,1.026) | 0.076 |
| genus Eubacteriumhalliigroup.id.11338 | CD | MRPRESSO | 0.698(0.485,1.007) | 0.073 |
| genus Eubacteriumnodatumgroup.id.11297 | CD | IVW | 0.978(0.866,1.105) | 0.725 |
| genus Eubacteriumnodatumgroup.id.11297 | CD | Weighted median | 1.02(0.87,1.197) | 0.804 |
| genus Eubacteriumnodatumgroup.id.11297 | CD | Weighted mode | 1.096(0.859,1.397) | 0.461 |
| genus Eubacteriumnodatumgroup.id.11297 | CD | MR-Robust | 1.026(0.883,1.192) | 0.740 |
| genus Eubacteriumnodatumgroup.id.11297 | CD | MR-Egger | 0.818(0.469,1.427) | 0.480 |
| genus Eubacteriumnodatumgroup.id.11297 | CD | MRRAPS | 1.004(0.882,1.143) | 0.951 |
| genus Eubacteriumnodatumgroup.id.11297 | CD | MRPRESSO | 0.978(0.866,1.105) | 0.732 |
| genus Eubacteriumoxidoreducensgroup.id.11339 | CD | IVW | 0.842(0.611,1.159) | 0.292 |
| genus Eubacteriumoxidoreducensgroup.id.11339 | CD | Weighted median | 0.816(0.584,1.14) | 0.232 |
| genus Eubacteriumoxidoreducensgroup.id.11339 | CD | Weighted mode | 0.772(0.446,1.338) | 0.357 |
| genus Eubacteriumoxidoreducensgroup.id.11339 | CD | MR-Robust | 0.838(0.606,1.16) | 0.287 |
| genus Eubacteriumoxidoreducensgroup.id.11339 | CD | MR-Egger | 0.458(0.201,1.044) | 0.063 |
| genus Eubacteriumoxidoreducensgroup.id.11339 | CD | MRRAPS | 0.8(0.572,1.12) | 0.194 |
| genus Eubacteriumoxidoreducensgroup.id.11339 | CD | MRPRESSO | 0.842(0.611,1.159) | 0.369 |
| genus Eubacteriumrectalegroup.id.14374 | CD | IVW | 1.123(0.833,1.514) | 0.448 |
| genus Eubacteriumrectalegroup.id.14374 | CD | Weighted median | 1.147(0.754,1.744) | 0.521 |
| genus Eubacteriumrectalegroup.id.14374 | CD | Weighted mode | 1.25(0.623,2.507) | 0.530 |
| genus Eubacteriumrectalegroup.id.14374 | CD | MR-Robust | 1.142(0.825,1.58) | 0.425 |
| genus Eubacteriumrectalegroup.id.14374 | CD | MR-Egger | 0.846(0.277,2.582) | 0.769 |
| genus Eubacteriumrectalegroup.id.14374 | CD | MRRAPS | 1.152(0.833,1.593) | 0.391 |
| genus Eubacteriumrectalegroup.id.14374 | CD | MRPRESSO | 1.123(0.838,1.504) | 0.461 |
| genus Eubacteriumruminantiumgroup.id.11340 | CD | IVW | 1.043(0.902,1.205) | 0.571 |
| genus Eubacteriumruminantiumgroup.id.11340 | CD | Weighted median | 1.069(0.877,1.302) | 0.508 |
| genus Eubacteriumruminantiumgroup.id.11340 | CD | Weighted mode | 1.101(0.751,1.615) | 0.621 |
| genus Eubacteriumruminantiumgroup.id.11340 | CD | MR-Robust | 1.044(0.91,1.197) | 0.537 |
| genus Eubacteriumruminantiumgroup.id.11340 | CD | MR-Egger | 1.218(0.706,2.099) | 0.478 |
| genus Eubacteriumruminantiumgroup.id.11340 | CD | MRRAPS | 1.045(0.894,1.222) | 0.581 |
| genus Eubacteriumruminantiumgroup.id.11340 | CD | MRPRESSO | 1.043(0.916,1.187) | 0.538 |
| genus Eubacteriumventriosumgroup.id.11341 | CD | IVW | 1.057(0.842,1.326) | 0.632 |
| genus Eubacteriumventriosumgroup.id.11341 | CD | Weighted median | 1.07(0.79,1.449) | 0.664 |
| genus Eubacteriumventriosumgroup.id.11341 | CD | Weighted mode | 1.051(0.617,1.79) | 0.855 |
| genus Eubacteriumventriosumgroup.id.11341 | CD | MR-Robust | 1.05(0.844,1.305) | 0.663 |
| genus Eubacteriumventriosumgroup.id.11341 | CD | MR-Egger | 0.733(0.264,2.032) | 0.550 |
| genus Eubacteriumventriosumgroup.id.11341 | CD | MRRAPS | 1.055(0.824,1.351) | 0.669 |
| genus Eubacteriumventriosumgroup.id.11341 | CD | MRPRESSO | 1.057(0.887,1.26) | 0.546 |
| genus Eubacteriumxylanophilumgroup.id.14375 | CD | IVW | 1.08(0.778,1.499) | 0.646 |
| genus Eubacteriumxylanophilumgroup.id.14375 | CD | Weighted median | 1.114(0.765,1.622) | 0.574 |
| genus Eubacteriumxylanophilumgroup.id.14375 | CD | Weighted mode | 1.384(0.757,2.533) | 0.291 |
| genus Eubacteriumxylanophilumgroup.id.14375 | CD | MR-Robust | 1.096(0.784,1.531) | 0.593 |
| genus Eubacteriumxylanophilumgroup.id.14375 | CD | MR-Egger | 1.71(0.601,4.86) | 0.314 |
| genus Eubacteriumxylanophilumgroup.id.14375 | CD | MRRAPS | 1.168(0.811,1.682) | 0.405 |
| genus Eubacteriumxylanophilumgroup.id.14375 | CD | MRPRESSO | 1.08(0.778,1.499) | 0.659 |
| genus Ruminococcusgauvreauiigroup.id.11342 | CD | IVW | 0.972(0.71,1.332) | 0.860 |
| genus Ruminococcusgauvreauiigroup.id.11342 | CD | Weighted median | 1.147(0.813,1.617) | 0.435 |
| genus Ruminococcusgauvreauiigroup.id.11342 | CD | Weighted mode | 1.329(0.724,2.44) | 0.358 |
| genus Ruminococcusgauvreauiigroup.id.11342 | CD | MR-Robust | 0.986(0.711,1.367) | 0.933 |
| genus Ruminococcusgauvreauiigroup.id.11342 | CD | MR-Egger | 0.577(0.124,2.675) | 0.482 |
| genus Ruminococcusgauvreauiigroup.id.11342 | CD | MRRAPS | 0.97(0.7,1.343) | 0.853 |
| genus Ruminococcusgauvreauiigroup.id.11342 | CD | MRPRESSO | 0.972(0.71,1.332) | 0.863 |
| genus Ruminococcusgnavusgroup.id.14376 | CD | IVW | 1.077(0.872,1.33) | 0.494 |
| genus Ruminococcusgnavusgroup.id.14376 | CD | Weighted median | 0.957(0.763,1.2) | 0.702 |
| genus Ruminococcusgnavusgroup.id.14376 | CD | Weighted mode | 0.921(0.667,1.272) | 0.618 |
| genus Ruminococcusgnavusgroup.id.14376 | CD | MR-Robust | 1.003(0.816,1.232) | 0.979 |
| genus Ruminococcusgnavusgroup.id.14376 | CD | MR-Egger | 0.608(0.237,1.561) | 0.301 |
| genus Ruminococcusgnavusgroup.id.14376 | CD | MRRAPS | 1.015(0.843,1.223) | 0.874 |
| genus Ruminococcusgnavusgroup.id.14376 | CD | MRPRESSO | 1.077(0.872,1.33) | 0.508 |
| genus Ruminococcustorquesgroup.id.14377 | CD | IVW | 0.838(0.491,1.43) | 0.516 |
| genus Ruminococcustorquesgroup.id.14377 | CD | Weighted median | 0.929(0.6,1.441) | 0.744 |
| genus Ruminococcustorquesgroup.id.14377 | CD | Weighted mode | 1.009(0.463,2.197) | 0.983 |
| genus Ruminococcustorquesgroup.id.14377 | CD | MR-Robust | 0.949(0.489,1.843) | 0.877 |
| genus Ruminococcustorquesgroup.id.14377 | CD | MR-Egger | 0.654(0.149,2.871) | 0.573 |
| genus Ruminococcustorquesgroup.id.14377 | CD | MRRAPS | 0.9(0.491,1.649) | 0.733 |
| genus Ruminococcustorquesgroup.id.14377 | CD | MRPRESSO | 0.838(0.491,1.43) | 0.532 |
| genus.Actinomyces.id.423 | CD | IVW | 0.941(0.764,1.158) | 0.563 |
| genus.Actinomyces.id.423 | CD | Weighted median | 0.955(0.734,1.242) | 0.730 |
| genus.Actinomyces.id.423 | CD | Weighted mode | 0.904(0.62,1.318) | 0.600 |
| genus.Actinomyces.id.423 | CD | MR-Robust | 0.95(0.78,1.158) | 0.613 |
| genus.Actinomyces.id.423 | CD | MR-Egger | 1(0.565,1.77) | 0.999 |
| genus.Actinomyces.id.423 | CD | MRRAPS | 0.949(0.756,1.19) | 0.648 |
| genus.Actinomyces.id.423 | CD | MRPRESSO | 0.941(0.786,1.126) | 0.528 |
| genus.Adlercreutzia.id.812 | CD | IVW | 0.98(0.789,1.217) | 0.854 |
| genus.Adlercreutzia.id.812 | CD | Weighted median | 1.004(0.756,1.334) | 0.976 |
| genus.Adlercreutzia.id.812 | CD | Weighted mode | 1.021(0.653,1.595) | 0.929 |
| genus.Adlercreutzia.id.812 | CD | MR-Robust | 0.991(0.726,1.353) | 0.956 |
| genus.Adlercreutzia.id.812 | CD | MR-Egger | 1.31(0.484,3.541) | 0.595 |
| genus.Adlercreutzia.id.812 | CD | MRRAPS | 0.984(0.778,1.246) | 0.896 |
| genus.Adlercreutzia.id.812 | CD | MRPRESSO | 0.98(0.828,1.159) | 0.818 |
| genus.Akkermansia.id.4037 | CD | IVW | 0.915(0.721,1.161) | 0.463 |
| genus.Akkermansia.id.4037 | CD | Weighted median | 0.923(0.689,1.236) | 0.589 |
| genus.Akkermansia.id.4037 | CD | Weighted mode | 0.905(0.577,1.419) | 0.664 |
| genus.Akkermansia.id.4037 | CD | MR-Robust | 0.894(0.69,1.159) | 0.399 |
| genus.Akkermansia.id.4037 | CD | MR-Egger | 0.802(0.314,2.045) | 0.644 |
| genus.Akkermansia.id.4037 | CD | MRRAPS | 0.903(0.719,1.135) | 0.383 |
| genus.Akkermansia.id.4037 | CD | MRPRESSO | 0.915(0.721,1.161) | 0.479 |
| genus.Alistipes.id.968 | CD | IVW | 0.985(0.768,1.262) | 0.903 |
| genus.Alistipes.id.968 | CD | Weighted median | 0.992(0.706,1.394) | 0.965 |
| genus.Alistipes.id.968 | CD | Weighted mode | 1.465(0.771,2.781) | 0.244 |
| genus.Alistipes.id.968 | CD | MR-Robust | 0.985(0.783,1.24) | 0.899 |
| genus.Alistipes.id.968 | CD | MR-Egger | 1.428(0.426,4.791) | 0.564 |
| genus.Alistipes.id.968 | CD | MRRAPS | 0.984(0.752,1.289) | 0.908 |
| genus.Alistipes.id.968 | CD | MRPRESSO | 0.985(0.789,1.229) | 0.894 |
| genus.Allisonella.id.2174 | CD | IVW | 1.031(0.874,1.217) | 0.715 |
| genus.Allisonella.id.2174 | CD | Weighted median | 1.075(0.887,1.304) | 0.460 |
| genus.Allisonella.id.2174 | CD | Weighted mode | 1.172(0.871,1.578) | 0.295 |
| genus.Allisonella.id.2174 | CD | MR-Robust | 1.04(0.873,1.241) | 0.659 |
| genus.Allisonella.id.2174 | CD | MR-Egger | 0.818(0.256,2.615) | 0.734 |
| genus.Allisonella.id.2174 | CD | MRRAPS | 1.044(0.876,1.245) | 0.628 |
| genus.Allisonella.id.2174 | CD | MRPRESSO | 1.031(0.874,1.217) | 0.725 |
| genus.Alloprevotella.id.961 | CD | IVW | 0.956(0.818,1.117) | 0.572 |
| genus.Alloprevotella.id.961 | CD | Weighted median | 0.949(0.786,1.146) | 0.588 |
| genus.Alloprevotella.id.961 | CD | Weighted mode | 0.951(0.726,1.246) | 0.713 |
| genus.Alloprevotella.id.961 | CD | MR-Robust | 0.957(0.847,1.082) | 0.486 |
| genus.Alloprevotella.id.961 | CD | MR-Egger | 0.929(0.214,4.033) | 0.921 |
| genus.Alloprevotella.id.961 | CD | MRRAPS | 0.956(0.805,1.134) | 0.603 |
| genus.Alloprevotella.id.961 | CD | MRPRESSO | 0.956(0.874,1.046) | 0.371 |
| genus.Anaerofilum.id.2053 | CD | IVW | 1.029(0.886,1.194) | 0.710 |
| genus.Anaerofilum.id.2053 | CD | Weighted median | 1.044(0.853,1.279) | 0.677 |
| genus.Anaerofilum.id.2053 | CD | Weighted mode | 1.028(0.724,1.461) | 0.876 |
| genus.Anaerofilum.id.2053 | CD | MR-Robust | 1.018(0.871,1.19) | 0.824 |
| genus.Anaerofilum.id.2053 | CD | MR-Egger | 0.831(0.384,1.799) | 0.638 |
| genus.Anaerofilum.id.2053 | CD | MRRAPS | 1.018(0.867,1.196) | 0.825 |
| genus.Anaerofilum.id.2053 | CD | MRPRESSO | 1.029(0.902,1.173) | 0.682 |
| genus.Anaerostipes.id.1991 | CD | IVW | 1.185(0.922,1.523) | 0.184 |
| genus.Anaerostipes.id.1991 | CD | Weighted median | 1.262(0.904,1.761) | 0.171 |
| genus.Anaerostipes.id.1991 | CD | Weighted mode | 1.354(0.769,2.384) | 0.293 |
| genus.Anaerostipes.id.1991 | CD | MR-Robust | 1.195(0.951,1.501) | 0.126 |
| genus.Anaerostipes.id.1991 | CD | MR-Egger | 1.362(0.557,3.331) | 0.498 |
| genus.Anaerostipes.id.1991 | CD | MRRAPS | 1.2(0.913,1.577) | 0.192 |
| genus.Anaerostipes.id.1991 | CD | MRPRESSO | 1.185(0.964,1.458) | 0.133 |
| genus.Anaerotruncus.id.2054 | CD | IVW | 0.823(0.645,1.052) | 0.120 |
| genus.Anaerotruncus.id.2054 | CD | Weighted median | 0.925(0.666,1.286) | 0.644 |
| genus.Anaerotruncus.id.2054 | CD | Weighted mode | 1.014(0.592,1.737) | 0.960 |
| genus.Anaerotruncus.id.2054 | CD | MR-Robust | 0.839(0.625,1.125) | 0.241 |
| genus.Anaerotruncus.id.2054 | CD | MR-Egger | 0.659(0.299,1.452) | 0.301 |
| genus.Anaerotruncus.id.2054 | CD | MRRAPS | 0.821(0.627,1.074) | 0.149 |
| genus.Anaerotruncus.id.2054 | CD | MRPRESSO | 0.823(0.685,0.99) | 0.061 |
| genus.Bacteroides.id.918 | CD | IVW | 1.187(0.852,1.654) | 0.310 |
| genus.Bacteroides.id.918 | CD | Weighted median | 1.395(0.903,2.156) | 0.134 |
| genus.Bacteroides.id.918 | CD | Weighted mode | 1.576(0.767,3.24) | 0.216 |
| genus.Bacteroides.id.918 | CD | MR-Robust | 1.241(0.685,2.25) | 0.476 |
| genus.Bacteroides.id.918 | CD | MR-Egger | 1.676(0.245,11.459) | 0.598 |
| genus.Bacteroides.id.918 | CD | MRRAPS | 1.214(0.847,1.739) | 0.291 |
| genus.Bacteroides.id.918 | CD | MRPRESSO | 1.187(0.878,1.606) | 0.302 |
| genus.Barnesiella.id.944 | CD | IVW | 1.072(0.853,1.347) | 0.550 |
| genus.Barnesiella.id.944 | CD | Weighted median | 0.997(0.726,1.369) | 0.986 |
| genus.Barnesiella.id.944 | CD | Weighted mode | 0.892(0.512,1.556) | 0.688 |
| genus.Barnesiella.id.944 | CD | MR-Robust | 1.075(0.855,1.353) | 0.535 |
| genus.Barnesiella.id.944 | CD | MR-Egger | 1.431(0.656,3.119) | 0.368 |
| genus.Barnesiella.id.944 | CD | MRRAPS | 1.096(0.85,1.413) | 0.481 |
| genus.Barnesiella.id.944 | CD | MRPRESSO | 1.072(0.853,1.347) | 0.561 |
| genus.Bifidobacterium.id.436 | CD | IVW | 0.914(0.757,1.105) | 0.353 |
| genus.Bifidobacterium.id.436 | CD | Weighted median | 0.904(0.716,1.14) | 0.393 |
| genus.Bifidobacterium.id.436 | CD | Weighted mode | 0.951(0.68,1.33) | 0.771 |
| genus.Bifidobacterium.id.436 | CD | MR-Robust | 0.888(0.749,1.053) | 0.173 |
| genus.Bifidobacterium.id.436 | CD | MR-Egger | 1.206(0.671,2.164) | 0.531 |
| genus.Bifidobacterium.id.436 | CD | MRRAPS | 0.899(0.738,1.095) | 0.290 |
| genus.Bifidobacterium.id.436 | CD | MRPRESSO | 0.914(0.757,1.105) | 0.364 |
| genus.Bilophila.id.3170 | CD | IVW | 1.124(0.807,1.566) | 0.488 |
| genus.Bilophila.id.3170 | CD | Weighted median | 1.098(0.775,1.558) | 0.598 |
| genus.Bilophila.id.3170 | CD | Weighted mode | 1.695(0.886,3.245) | 0.111 |
| genus.Bilophila.id.3170 | CD | MR-Robust | 1.146(0.808,1.625) | 0.446 |
| genus.Bilophila.id.3170 | CD | MR-Egger | 2.567(0.573,11.495) | 0.218 |
| genus.Bilophila.id.3170 | CD | MRRAPS | 1.207(0.859,1.696) | 0.278 |
| genus.Bilophila.id.3170 | CD | MRPRESSO | 1.124(0.807,1.566) | 0.501 |
| genus.Blautia.id.1992 | CD | IVW | 1.137(0.899,1.439) | 0.284 |
| genus.Blautia.id.1992 | CD | Weighted median | 1.142(0.821,1.588) | 0.431 |
| genus.Blautia.id.1992 | CD | Weighted mode | 1.043(0.599,1.815) | 0.882 |
| genus.Blautia.id.1992 | CD | MR-Robust | 1.134(0.88,1.461) | 0.330 |
| genus.Blautia.id.1992 | CD | MR-Egger | 1.164(0.691,1.96) | 0.568 |
| genus.Blautia.id.1992 | CD | MRRAPS | 1.143(0.858,1.521) | 0.360 |
| genus.Blautia.id.1992 | CD | MRPRESSO | 1.137(0.91,1.421) | 0.281 |
| genus.Butyricicoccus.id.2055 | CD | IVW | 0.89(0.582,1.361) | 0.591 |
| genus.Butyricicoccus.id.2055 | CD | Weighted median | 0.877(0.608,1.263) | 0.480 |
| genus.Butyricicoccus.id.2055 | CD | Weighted mode | 0.848(0.572,1.257) | 0.411 |
| genus.Butyricicoccus.id.2055 | CD | MR-Robust | 0.883(0.635,1.228) | 0.459 |
| genus.Butyricicoccus.id.2055 | CD | MR-Egger | 0.888(0.387,2.034) | 0.778 |
| genus.Butyricicoccus.id.2055 | CD | MRRAPS | 0.85(0.609,1.188) | 0.342 |
| genus.Butyricicoccus.id.2055 | CD | MRPRESSO | 0.89(0.582,1.361) | 0.608 |
| genus.Butyricimonas.id.945 | CD | IVW | 0.971(0.76,1.24) | 0.813 |
| genus.Butyricimonas.id.945 | CD | Weighted median | 1.019(0.774,1.341) | 0.894 |
| genus.Butyricimonas.id.945 | CD | Weighted mode | 1.005(0.64,1.577) | 0.984 |
| genus.Butyricimonas.id.945 | CD | MR-Robust | 0.988(0.773,1.263) | 0.925 |
| genus.Butyricimonas.id.945 | CD | MR-Egger | 0.955(0.411,2.221) | 0.915 |
| genus.Butyricimonas.id.945 | CD | MRRAPS | 0.988(0.766,1.274) | 0.925 |
| genus.Butyricimonas.id.945 | CD | MRPRESSO | 0.971(0.76,1.24) | 0.817 |
| genus.Butyrivibrio.id.1993 | CD | IVW | 0.861(0.772,0.96) | 0.007 |
| genus.Butyrivibrio.id.1993 | CD | Weighted median | 0.89(0.771,1.028) | 0.113 |
| genus.Butyrivibrio.id.1993 | CD | Weighted mode | 0.902(0.716,1.137) | 0.384 |
| genus.Butyrivibrio.id.1993 | CD | MR-Robust | 0.891(0.781,1.018) | 0.089 |
| genus.Butyrivibrio.id.1993 | CD | MR-Egger | 1.086(0.655,1.8) | 0.750 |
| genus.Butyrivibrio.id.1993 | CD | MRRAPS | 0.868(0.771,0.978) | 0.020 |
| genus.Butyrivibrio.id.1993 | CD | MRPRESSO | 0.861(0.792,0.936) | 0.004 |
| genus.CandidatusSoleaferrea.id.11350 | CD | IVW | 0.981(0.821,1.173) | 0.836 |
| genus.CandidatusSoleaferrea.id.11350 | CD | Weighted median | 0.946(0.75,1.192) | 0.638 |
| genus.CandidatusSoleaferrea.id.11350 | CD | Weighted mode | 0.937(0.642,1.367) | 0.734 |
| genus.CandidatusSoleaferrea.id.11350 | CD | MR-Robust | 0.982(0.847,1.139) | 0.809 |
| genus.CandidatusSoleaferrea.id.11350 | CD | MR-Egger | 0.542(0.081,3.621) | 0.527 |
| genus.CandidatusSoleaferrea.id.11350 | CD | MRRAPS | 0.981(0.807,1.192) | 0.847 |
| genus.CandidatusSoleaferrea.id.11350 | CD | MRPRESSO | 0.981(0.865,1.113) | 0.777 |
| genus.Catenibacterium.id.2153 | CD | IVW | 0.905(0.644,1.272) | 0.564 |
| genus.Catenibacterium.id.2153 | CD | Weighted median | 0.97(0.75,1.255) | 0.817 |
| genus.Catenibacterium.id.2153 | CD | Weighted mode | 1.075(0.786,1.471) | 0.649 |
| genus.Catenibacterium.id.2153 | CD | MR-Robust | 0.956(0.624,1.465) | 0.836 |
| genus.Catenibacterium.id.2153 | CD | MR-Egger | 2.187(0.065,73.123) | 0.662 |
| genus.Catenibacterium.id.2153 | CD | MRRAPS | 0.988(0.72,1.355) | 0.939 |
| genus.Catenibacterium.id.2153 | CD | MRPRESSO | 0.905(0.644,1.272) | 0.595 |
| genus.ChristensenellaceaeR.7group.id.11283 | CD | IVW | 0.984(0.737,1.315) | 0.915 |
| genus.ChristensenellaceaeR.7group.id.11283 | CD | Weighted median | 0.965(0.654,1.423) | 0.858 |
| genus.ChristensenellaceaeR.7group.id.11283 | CD | Weighted mode | 0.923(0.51,1.67) | 0.791 |
| genus.ChristensenellaceaeR.7group.id.11283 | CD | MR-Robust | 0.98(0.752,1.277) | 0.881 |
| genus.ChristensenellaceaeR.7group.id.11283 | CD | MR-Egger | 1.254(0.538,2.924) | 0.601 |
| genus.ChristensenellaceaeR.7group.id.11283 | CD | MRRAPS | 0.986(0.719,1.352) | 0.928 |
| genus.ChristensenellaceaeR.7group.id.11283 | CD | MRPRESSO | 0.984(0.762,1.272) | 0.906 |
| genus.Clostridiumsensustricto1.id.1873 | CD | IVW | 0.847(0.614,1.169) | 0.314 |
| genus.Clostridiumsensustricto1.id.1873 | CD | Weighted median | 0.972(0.674,1.403) | 0.881 |
| genus.Clostridiumsensustricto1.id.1873 | CD | Weighted mode | 1.073(0.673,1.709) | 0.768 |
| genus.Clostridiumsensustricto1.id.1873 | CD | MR-Robust | 0.85(0.615,1.175) | 0.326 |
| genus.Clostridiumsensustricto1.id.1873 | CD | MR-Egger | 1.244(0.625,2.477) | 0.533 |
| genus.Clostridiumsensustricto1.id.1873 | CD | MRRAPS | 0.891(0.619,1.281) | 0.532 |
| genus.Clostridiumsensustricto1.id.1873 | CD | MRPRESSO | 0.847(0.614,1.169) | 0.353 |
| genus.Collinsella.id.815 | CD | IVW | 0.968(0.705,1.33) | 0.842 |
| genus.Collinsella.id.815 | CD | Weighted median | 0.939(0.651,1.354) | 0.735 |
| genus.Collinsella.id.815 | CD | Weighted mode | 0.834(0.449,1.548) | 0.565 |
| genus.Collinsella.id.815 | CD | MR-Robust | 0.977(0.721,1.323) | 0.878 |
| genus.Collinsella.id.815 | CD | MR-Egger | 0.511(0.149,1.749) | 0.285 |
| genus.Collinsella.id.815 | CD | MRRAPS | 0.976(0.694,1.372) | 0.888 |
| genus.Collinsella.id.815 | CD | MRPRESSO | 0.968(0.705,1.33) | 0.846 |
| genus.Coprobacter.id.949 | CD | IVW | 1.019(0.817,1.27) | 0.870 |
| genus.Coprobacter.id.949 | CD | Weighted median | 1.016(0.793,1.302) | 0.901 |
| genus.Coprobacter.id.949 | CD | Weighted mode | 0.919(0.629,1.343) | 0.663 |
| genus.Coprobacter.id.949 | CD | MR-Robust | 1.022(0.832,1.256) | 0.833 |
| genus.Coprobacter.id.949 | CD | MR-Egger | 1.257(0.578,2.734) | 0.565 |
| genus.Coprobacter.id.949 | CD | MRRAPS | 1.048(0.843,1.303) | 0.670 |
| genus.Coprobacter.id.949 | CD | MRPRESSO | 1.019(0.817,1.27) | 0.873 |
| genus.Coprococcus1.id.11301 | CD | IVW | 0.94(0.745,1.187) | 0.605 |
| genus.Coprococcus1.id.11301 | CD | Weighted median | 0.909(0.668,1.237) | 0.545 |
| genus.Coprococcus1.id.11301 | CD | Weighted mode | 0.974(0.646,1.47) | 0.902 |
| genus.Coprococcus1.id.11301 | CD | MR-Robust | 0.951(0.788,1.148) | 0.600 |
| genus.Coprococcus1.id.11301 | CD | MR-Egger | 1.083(0.621,1.888) | 0.779 |
| genus.Coprococcus1.id.11301 | CD | MRRAPS | 0.951(0.737,1.226) | 0.696 |
| genus.Coprococcus1.id.11301 | CD | MRPRESSO | 0.94(0.775,1.141) | 0.546 |
| genus.Coprococcus2.id.11302 | CD | IVW | 1.085(0.853,1.381) | 0.505 |
| genus.Coprococcus2.id.11302 | CD | Weighted median | 1.078(0.802,1.45) | 0.617 |
| genus.Coprococcus2.id.11302 | CD | Weighted mode | 1.081(0.698,1.674) | 0.727 |
| genus.Coprococcus2.id.11302 | CD | MR-Robust | 1.07(0.85,1.347) | 0.564 |
| genus.Coprococcus2.id.11302 | CD | MR-Egger | 0.883(0.199,3.926) | 0.870 |
| genus.Coprococcus2.id.11302 | CD | MRRAPS | 1.086(0.832,1.418) | 0.544 |
| genus.Coprococcus2.id.11302 | CD | MRPRESSO | 1.085(0.981,1.201) | 0.148 |
| genus.Coprococcus3.id.11303 | CD | IVW | 1.193(0.911,1.563) | 0.199 |
| genus.Coprococcus3.id.11303 | CD | Weighted median | 1.159(0.808,1.663) | 0.422 |
| genus.Coprococcus3.id.11303 | CD | Weighted mode | 1.214(0.696,2.115) | 0.494 |
| genus.Coprococcus3.id.11303 | CD | MR-Robust | 1.198(0.938,1.529) | 0.147 |
| genus.Coprococcus3.id.11303 | CD | MR-Egger | 2.18(0.703,6.757) | 0.177 |
| genus.Coprococcus3.id.11303 | CD | MRRAPS | 1.21(0.902,1.623) | 0.204 |
| genus.Coprococcus3.id.11303 | CD | MRPRESSO | 1.193(0.94,1.515) | 0.177 |
| genus.DefluviitaleaceaeUCG011.id.11287 | CD | IVW | 1.252(1.027,1.526) | 0.026 |
| genus.DefluviitaleaceaeUCG011.id.11287 | CD | Weighted median | 1.173(0.91,1.511) | 0.218 |
| genus.DefluviitaleaceaeUCG011.id.11287 | CD | Weighted mode | 1.111(0.755,1.633) | 0.594 |
| genus.DefluviitaleaceaeUCG011.id.11287 | CD | MR-Robust | 1.209(0.802,1.824) | 0.365 |
| genus.DefluviitaleaceaeUCG011.id.11287 | CD | MR-Egger | 1.352(0.668,2.737) | 0.402 |
| genus.DefluviitaleaceaeUCG011.id.11287 | CD | MRRAPS | 1.257(1.01,1.564) | 0.040 |
| genus.DefluviitaleaceaeUCG011.id.11287 | CD | MRPRESSO | 1.252(1.106,1.418) | 0.006 |
| genus.Desulfovibrio.id.3173 | CD | IVW | 1.091(0.889,1.339) | 0.406 |
| genus.Desulfovibrio.id.3173 | CD | Weighted median | 1.049(0.796,1.382) | 0.734 |
| genus.Desulfovibrio.id.3173 | CD | Weighted mode | 1.001(0.688,1.456) | 0.996 |
| genus.Desulfovibrio.id.3173 | CD | MR-Robust | 1.055(0.844,1.319) | 0.638 |
| genus.Desulfovibrio.id.3173 | CD | MR-Egger | 1.102(0.592,2.051) | 0.758 |
| genus.Desulfovibrio.id.3173 | CD | MRRAPS | 1.056(0.855,1.306) | 0.612 |
| genus.Desulfovibrio.id.3173 | CD | MRPRESSO | 1.091(0.889,1.339) | 0.426 |
| genus.Dialister.id.2183 | CD | IVW | 0.896(0.673,1.193) | 0.452 |
| genus.Dialister.id.2183 | CD | Weighted median | 0.81(0.573,1.145) | 0.233 |
| genus.Dialister.id.2183 | CD | Weighted mode | 0.632(0.336,1.187) | 0.154 |
| genus.Dialister.id.2183 | CD | MR-Robust | 0.891(0.668,1.189) | 0.432 |
| genus.Dialister.id.2183 | CD | MR-Egger | 1.235(0.335,4.561) | 0.751 |
| genus.Dialister.id.2183 | CD | MRRAPS | 0.884(0.65,1.203) | 0.433 |
| genus.Dialister.id.2183 | CD | MRPRESSO | 0.896(0.673,1.193) | 0.470 |
| genus.Dorea.id.1997 | CD | IVW | 0.922(0.711,1.195) | 0.540 |
| genus.Dorea.id.1997 | CD | Weighted median | 0.9(0.637,1.271) | 0.549 |
| genus.Dorea.id.1997 | CD | Weighted mode | 0.851(0.493,1.469) | 0.563 |
| genus.Dorea.id.1997 | CD | MR-Robust | 0.964(0.501,1.858) | 0.914 |
| genus.Dorea.id.1997 | CD | MR-Egger | 0.931(0.477,1.817) | 0.834 |
| genus.Dorea.id.1997 | CD | MRRAPS | 0.946(0.714,1.255) | 0.702 |
| genus.Dorea.id.1997 | CD | MRPRESSO | 0.922(0.747,1.138) | 0.467 |
| genus.Eggerthella.id.819 | CD | IVW | 0.881(0.748,1.039) | 0.132 |
| genus.Eggerthella.id.819 | CD | Weighted median | 1.001(0.802,1.249) | 0.991 |
| genus.Eggerthella.id.819 | CD | Weighted mode | 1.042(0.719,1.511) | 0.827 |
| genus.Eggerthella.id.819 | CD | MR-Robust | 0.89(0.634,1.249) | 0.501 |
| genus.Eggerthella.id.819 | CD | MR-Egger | 1.175(0.532,2.595) | 0.689 |
| genus.Eggerthella.id.819 | CD | MRRAPS | 0.877(0.733,1.05) | 0.153 |
| genus.Eggerthella.id.819 | CD | MRPRESSO | 0.881(0.763,1.018) | 0.120 |
| genus.Eisenbergiella.id.11304 | CD | IVW | 1.061(0.906,1.243) | 0.464 |
| genus.Eisenbergiella.id.11304 | CD | Weighted median | 1.196(0.953,1.5) | 0.122 |
| genus.Eisenbergiella.id.11304 | CD | Weighted mode | 1.297(0.854,1.97) | 0.222 |
| genus.Eisenbergiella.id.11304 | CD | MR-Robust | 1.064(0.89,1.272) | 0.494 |
| genus.Eisenbergiella.id.11304 | CD | MR-Egger | 2.186(0.628,7.614) | 0.219 |
| genus.Eisenbergiella.id.11304 | CD | MRRAPS | 1.066(0.891,1.274) | 0.485 |
| genus.Eisenbergiella.id.11304 | CD | MRPRESSO | 1.061(0.907,1.241) | 0.476 |
| genus.Enterorhabdus.id.820 | CD | IVW | 0.841(0.672,1.052) | 0.129 |
| genus.Enterorhabdus.id.820 | CD | Weighted median | 0.928(0.688,1.253) | 0.627 |
| genus.Enterorhabdus.id.820 | CD | Weighted mode | 0.982(0.671,1.436) | 0.924 |
| genus.Enterorhabdus.id.820 | CD | MR-Robust | 0.846(0.661,1.082) | 0.182 |
| genus.Enterorhabdus.id.820 | CD | MR-Egger | 1.111(0.616,2.007) | 0.726 |
| genus.Enterorhabdus.id.820 | CD | MRRAPS | 0.839(0.651,1.081) | 0.174 |
| genus.Enterorhabdus.id.820 | CD | MRPRESSO | 0.841(0.676,1.045) | 0.179 |
| genus.Erysipelatoclostridium.id.11381 | CD | IVW | 0.873(0.736,1.037) | 0.123 |
| genus.Erysipelatoclostridium.id.11381 | CD | Weighted median | 0.901(0.712,1.141) | 0.388 |
| genus.Erysipelatoclostridium.id.11381 | CD | Weighted mode | 0.917(0.609,1.381) | 0.678 |
| genus.Erysipelatoclostridium.id.11381 | CD | MR-Robust | 0.887(0.747,1.054) | 0.174 |
| genus.Erysipelatoclostridium.id.11381 | CD | MR-Egger | 1.007(0.5,2.028) | 0.984 |
| genus.Erysipelatoclostridium.id.11381 | CD | MRRAPS | 0.884(0.734,1.065) | 0.193 |
| genus.Erysipelatoclostridium.id.11381 | CD | MRPRESSO | 0.873(0.746,1.022) | 0.113 |
| genus.ErysipelotrichaceaeUCG003.id.11384 | CD | IVW | 1.071(0.873,1.315) | 0.511 |
| genus.ErysipelotrichaceaeUCG003.id.11384 | CD | Weighted median | 0.945(0.72,1.238) | 0.680 |
| genus.ErysipelotrichaceaeUCG003.id.11384 | CD | Weighted mode | 0.85(0.566,1.276) | 0.432 |
| genus.ErysipelotrichaceaeUCG003.id.11384 | CD | MR-Robust | 1.012(0.734,1.395) | 0.943 |
| genus.ErysipelotrichaceaeUCG003.id.11384 | CD | MR-Egger | 0.793(0.454,1.386) | 0.415 |
| genus.ErysipelotrichaceaeUCG003.id.11384 | CD | MRRAPS | 1.027(0.834,1.265) | 0.800 |
| genus.ErysipelotrichaceaeUCG003.id.11384 | CD | MRPRESSO | 1.071(0.873,1.315) | 0.520 |
| genus.Escherichia.Shigella.id.3504 | CD | IVW | 1.124(0.875,1.443) | 0.360 |
| genus.Escherichia.Shigella.id.3504 | CD | Weighted median | 1.147(0.819,1.608) | 0.425 |
| genus.Escherichia.Shigella.id.3504 | CD | Weighted mode | 1.195(0.7,2.04) | 0.514 |
| genus.Escherichia.Shigella.id.3504 | CD | MR-Robust | 1.122(0.894,1.409) | 0.320 |
| genus.Escherichia.Shigella.id.3504 | CD | MR-Egger | 1.792(0.822,3.906) | 0.143 |
| genus.Escherichia.Shigella.id.3504 | CD | MRRAPS | 1.116(0.851,1.465) | 0.428 |
| genus.Escherichia.Shigella.id.3504 | CD | MRPRESSO | 1.124(0.893,1.414) | 0.345 |
| genus.Faecalibacterium.id.2057 | CD | IVW | 1.005(0.753,1.342) | 0.973 |
| genus.Faecalibacterium.id.2057 | CD | Weighted median | 0.999(0.7,1.426) | 0.997 |
| genus.Faecalibacterium.id.2057 | CD | Weighted mode | 1.012(0.644,1.591) | 0.958 |
| genus.Faecalibacterium.id.2057 | CD | MR-Robust | 1.002(0.759,1.323) | 0.989 |
| genus.Faecalibacterium.id.2057 | CD | MR-Egger | 1.307(0.743,2.302) | 0.353 |
| genus.Faecalibacterium.id.2057 | CD | MRRAPS | 1.017(0.74,1.397) | 0.919 |
| genus.Faecalibacterium.id.2057 | CD | MRPRESSO | 1.005(0.753,1.342) | 0.974 |
| genus.FamilyXIIIAD3011group.id.11293 | CD | IVW | 1(0.782,1.278) | 0.997 |
| genus.FamilyXIIIAD3011group.id.11293 | CD | Weighted median | 1.088(0.783,1.512) | 0.617 |
| genus.FamilyXIIIAD3011group.id.11293 | CD | Weighted mode | 1.233(0.685,2.221) | 0.484 |
| genus.FamilyXIIIAD3011group.id.11293 | CD | MR-Robust | 1.014(0.775,1.326) | 0.922 |
| genus.FamilyXIIIAD3011group.id.11293 | CD | MR-Egger | 1.012(0.332,3.089) | 0.983 |
| genus.FamilyXIIIAD3011group.id.11293 | CD | MRRAPS | 1.011(0.774,1.32) | 0.938 |
| genus.FamilyXIIIAD3011group.id.11293 | CD | MRPRESSO | 1(0.811,1.232) | 0.997 |
| genus.FamilyXIIIUCG001.id.11294 | CD | IVW | 1.315(1.008,1.717) | 0.044 |
| genus.FamilyXIIIUCG001.id.11294 | CD | Weighted median | 1.351(0.944,1.935) | 0.100 |
| genus.FamilyXIIIUCG001.id.11294 | CD | Weighted mode | 1.317(0.755,2.297) | 0.332 |
| genus.FamilyXIIIUCG001.id.11294 | CD | MR-Robust | 1.292(0.909,1.837) | 0.153 |
| genus.FamilyXIIIUCG001.id.11294 | CD | MR-Egger | 1.806(0.87,3.749) | 0.113 |
| genus.FamilyXIIIUCG001.id.11294 | CD | MRRAPS | 1.323(0.988,1.771) | 0.060 |
| genus.FamilyXIIIUCG001.id.11294 | CD | MRPRESSO | 1.315(1.085,1.595) | 0.027 |
| genus.Flavonifractor.id.2059 | CD | IVW | 0.999(0.679,1.472) | 0.998 |
| genus.Flavonifractor.id.2059 | CD | Weighted median | 0.823(0.551,1.231) | 0.343 |
| genus.Flavonifractor.id.2059 | CD | Weighted mode | 0.72(0.404,1.284) | 0.266 |
| genus.Flavonifractor.id.2059 | CD | MR-Robust | 0.996(0.693,1.433) | 0.984 |
| genus.Flavonifractor.id.2059 | CD | MR-Egger | 3.852(0.853,17.383) | 0.079 |
| genus.Flavonifractor.id.2059 | CD | MRRAPS | 1.112(0.714,1.733) | 0.638 |
| genus.Flavonifractor.id.2059 | CD | MRPRESSO | 0.999(0.679,1.472) | 0.998 |
| genus.Fusicatenibacter.id.11305 | CD | IVW | 0.913(0.723,1.155) | 0.449 |
| genus.Fusicatenibacter.id.11305 | CD | Weighted median | 0.942(0.693,1.281) | 0.705 |
| genus.Fusicatenibacter.id.11305 | CD | Weighted mode | 1.05(0.6,1.838) | 0.864 |
| genus.Fusicatenibacter.id.11305 | CD | MR-Robust | 0.936(0.719,1.219) | 0.623 |
| genus.Fusicatenibacter.id.11305 | CD | MR-Egger | 1.105(0.43,2.836) | 0.836 |
| genus.Fusicatenibacter.id.11305 | CD | MRRAPS | 0.949(0.736,1.224) | 0.686 |
| genus.Fusicatenibacter.id.11305 | CD | MRPRESSO | 0.913(0.723,1.155) | 0.459 |
| genus.Gordonibacter.id.821 | CD | IVW | 0.994(0.881,1.122) | 0.921 |
| genus.Gordonibacter.id.821 | CD | Weighted median | 0.951(0.816,1.108) | 0.519 |
| genus.Gordonibacter.id.821 | CD | Weighted mode | 0.915(0.725,1.153) | 0.450 |
| genus.Gordonibacter.id.821 | CD | MR-Robust | 0.952(0.828,1.094) | 0.484 |
| genus.Gordonibacter.id.821 | CD | MR-Egger | 1.254(0.743,2.116) | 0.397 |
| genus.Gordonibacter.id.821 | CD | MRRAPS | 0.987(0.865,1.127) | 0.846 |
| genus.Gordonibacter.id.821 | CD | MRPRESSO | 0.994(0.916,1.079) | 0.886 |
| genus.Haemophilus.id.3698 | CD | IVW | 1.02(0.822,1.267) | 0.854 |
| genus.Haemophilus.id.3698 | CD | Weighted median | 1.118(0.853,1.466) | 0.418 |
| genus.Haemophilus.id.3698 | CD | Weighted mode | 1.22(0.819,1.816) | 0.328 |
| genus.Haemophilus.id.3698 | CD | MR-Robust | 1.028(0.808,1.306) | 0.824 |
| genus.Haemophilus.id.3698 | CD | MR-Egger | 1.002(0.584,1.719) | 0.994 |
| genus.Haemophilus.id.3698 | CD | MRRAPS | 1.008(0.754,1.349) | 0.955 |
| genus.Haemophilus.id.3698 | CD | MRPRESSO | 1.02(0.822,1.267) | 0.859 |
| genus.Holdemanella.id.11393 | CD | IVW | 0.947(0.732,1.224) | 0.675 |
| genus.Holdemanella.id.11393 | CD | Weighted median | 0.924(0.7,1.22) | 0.577 |
| genus.Holdemanella.id.11393 | CD | Weighted mode | 0.759(0.439,1.312) | 0.324 |
| genus.Holdemanella.id.11393 | CD | MR-Robust | 0.944(0.725,1.229) | 0.668 |
| genus.Holdemanella.id.11393 | CD | MR-Egger | 1.294(0.581,2.885) | 0.528 |
| genus.Holdemanella.id.11393 | CD | MRRAPS | 0.989(0.737,1.326) | 0.940 |
| genus.Holdemanella.id.11393 | CD | MRPRESSO | 0.947(0.732,1.224) | 0.684 |
| genus.Holdemania.id.2157 | CD | IVW | 0.912(0.735,1.132) | 0.404 |
| genus.Holdemania.id.2157 | CD | Weighted median | 1.069(0.829,1.379) | 0.605 |
| genus.Holdemania.id.2157 | CD | Weighted mode | 1.185(0.783,1.794) | 0.423 |
| genus.Holdemania.id.2157 | CD | MR-Robust | 0.912(0.722,1.153) | 0.443 |
| genus.Holdemania.id.2157 | CD | MR-Egger | 1.455(0.801,2.646) | 0.218 |
| genus.Holdemania.id.2157 | CD | MRRAPS | 0.945(0.729,1.224) | 0.666 |
| genus.Holdemania.id.2157 | CD | MRPRESSO | 0.912(0.735,1.132) | 0.418 |
| genus.Howardella.id.2000 | CD | IVW | 0.938(0.817,1.077) | 0.364 |
| genus.Howardella.id.2000 | CD | Weighted median | 0.951(0.798,1.133) | 0.572 |
| genus.Howardella.id.2000 | CD | Weighted mode | 0.948(0.723,1.242) | 0.697 |
| genus.Howardella.id.2000 | CD | MR-Robust | 0.943(0.829,1.072) | 0.367 |
| genus.Howardella.id.2000 | CD | MR-Egger | 0.836(0.451,1.549) | 0.569 |
| genus.Howardella.id.2000 | CD | MRRAPS | 0.937(0.806,1.09) | 0.398 |
| genus.Howardella.id.2000 | CD | MRPRESSO | 0.938(0.852,1.033) | 0.227 |
| genus.Hungatella.id.11306 | CD | IVW | 0.832(0.673,1.029) | 0.090 |
| genus.Hungatella.id.11306 | CD | Weighted median | 0.936(0.703,1.245) | 0.648 |
| genus.Hungatella.id.11306 | CD | Weighted mode | 0.957(0.665,1.375) | 0.810 |
| genus.Hungatella.id.11306 | CD | MR-Robust | 0.878(0.546,1.412) | 0.590 |
| genus.Hungatella.id.11306 | CD | MR-Egger | 0.44(0.121,1.596) | 0.212 |
| genus.Hungatella.id.11306 | CD | MRRAPS | 0.843(0.671,1.06) | 0.144 |
| genus.Hungatella.id.11306 | CD | MRPRESSO | 0.832(0.673,1.029) | 0.166 |
| genus.Intestinibacter.id.11345 | CD | IVW | 0.95(0.759,1.189) | 0.654 |
| genus.Intestinibacter.id.11345 | CD | Weighted median | 0.995(0.758,1.305) | 0.970 |
| genus.Intestinibacter.id.11345 | CD | Weighted mode | 0.999(0.633,1.577) | 0.996 |
| genus.Intestinibacter.id.11345 | CD | MR-Robust | 0.947(0.751,1.195) | 0.647 |
| genus.Intestinibacter.id.11345 | CD | MR-Egger | 0.4(0.213,0.749) | 0.004 |
| genus.Intestinibacter.id.11345 | CD | MRRAPS | 0.935(0.751,1.164) | 0.545 |
| genus.Intestinibacter.id.11345 | CD | MRPRESSO | 0.95(0.759,1.189) | 0.660 |
| genus.Intestinimonas.id.2062 | CD | IVW | 0.977(0.819,1.166) | 0.800 |
| genus.Intestinimonas.id.2062 | CD | Weighted median | 1.022(0.805,1.298) | 0.857 |
| genus.Intestinimonas.id.2062 | CD | Weighted mode | 1.079(0.734,1.586) | 0.699 |
| genus.Intestinimonas.id.2062 | CD | MR-Robust | 0.978(0.842,1.136) | 0.767 |
| genus.Intestinimonas.id.2062 | CD | MR-Egger | 1.035(0.657,1.631) | 0.882 |
| genus.Intestinimonas.id.2062 | CD | MRRAPS | 0.973(0.803,1.178) | 0.776 |
| genus.Intestinimonas.id.2062 | CD | MRPRESSO | 0.977(0.836,1.143) | 0.779 |
| genus.Lachnoclostridium.id.11308 | CD | IVW | 0.849(0.655,1.102) | 0.219 |
| genus.Lachnoclostridium.id.11308 | CD | Weighted median | 0.961(0.675,1.367) | 0.823 |
| genus.Lachnoclostridium.id.11308 | CD | Weighted mode | 1.002(0.587,1.711) | 0.994 |
| genus.Lachnoclostridium.id.11308 | CD | MR-Robust | 0.927(0.658,1.305) | 0.663 |
| genus.Lachnoclostridium.id.11308 | CD | MR-Egger | 0.493(0.191,1.27) | 0.143 |
| genus.Lachnoclostridium.id.11308 | CD | MRRAPS | 0.888(0.671,1.175) | 0.405 |
| genus.Lachnoclostridium.id.11308 | CD | MRPRESSO | 0.849(0.655,1.102) | 0.242 |
| genus.Lachnospira.id.2004 | CD | IVW | 0.766(0.503,1.165) | 0.213 |
| genus.Lachnospira.id.2004 | CD | Weighted median | 0.702(0.427,1.155) | 0.164 |
| genus.Lachnospira.id.2004 | CD | Weighted mode | 0.562(0.253,1.251) | 0.158 |
| genus.Lachnospira.id.2004 | CD | MR-Robust | 0.752(0.488,1.16) | 0.198 |
| genus.Lachnospira.id.2004 | CD | MR-Egger | 0.167(0.023,1.215) | 0.077 |
| genus.Lachnospira.id.2004 | CD | MRRAPS | 0.71(0.464,1.085) | 0.113 |
| genus.Lachnospira.id.2004 | CD | MRPRESSO | 0.766(0.503,1.165) | 0.259 |
| genus.LachnospiraceaeFCS020group.id.11314 | CD | IVW | 1.211(0.964,1.521) | 0.100 |
| genus.LachnospiraceaeFCS020group.id.11314 | CD | Weighted median | 1.253(0.931,1.686) | 0.136 |
| genus.LachnospiraceaeFCS020group.id.11314 | CD | Weighted mode | 1.22(0.791,1.883) | 0.368 |
| genus.LachnospiraceaeFCS020group.id.11314 | CD | MR-Robust | 1.203(0.975,1.485) | 0.085 |
| genus.LachnospiraceaeFCS020group.id.11314 | CD | MR-Egger | 1.398(0.758,2.578) | 0.283 |
| genus.LachnospiraceaeFCS020group.id.11314 | CD | MRRAPS | 1.2(0.957,1.505) | 0.115 |
| genus.LachnospiraceaeFCS020group.id.11314 | CD | MRPRESSO | 1.211(0.964,1.521) | 0.126 |
| genus.LachnospiraceaeNC2004group.id.11316 | CD | IVW | 1.003(0.832,1.208) | 0.978 |
| genus.LachnospiraceaeNC2004group.id.11316 | CD | Weighted median | 1.078(0.852,1.365) | 0.532 |
| genus.LachnospiraceaeNC2004group.id.11316 | CD | Weighted mode | 1.103(0.765,1.59) | 0.599 |
| genus.LachnospiraceaeNC2004group.id.11316 | CD | MR-Robust | 1.028(0.757,1.396) | 0.859 |
| genus.LachnospiraceaeNC2004group.id.11316 | CD | MR-Egger | 1.138(0.517,2.505) | 0.748 |
| genus.LachnospiraceaeNC2004group.id.11316 | CD | MRRAPS | 1.003(0.818,1.229) | 0.979 |
| genus.LachnospiraceaeNC2004group.id.11316 | CD | MRPRESSO | 1.003(0.88,1.142) | 0.969 |
| genus.LachnospiraceaeND3007group.id.11317 | CD | IVW | 1.548(0.888,2.698) | 0.123 |
| genus.LachnospiraceaeND3007group.id.11317 | CD | Weighted median | 1.281(0.604,2.718) | 0.519 |
| genus.LachnospiraceaeND3007group.id.11317 | CD | Weighted mode | 1.129(0.49,2.6) | 0.776 |
| genus.LachnospiraceaeND3007group.id.11317 | CD | MR-Robust | 1.531(0.836,2.805) | 0.168 |
| genus.LachnospiraceaeND3007group.id.11317 | CD | MR-Egger | 8593.258(0.663,111456473.106) | 0.061 |
| genus.LachnospiraceaeND3007group.id.11317 | CD | MRRAPS | 1.609(0.803,3.226) | 0.180 |
| genus.LachnospiraceaeND3007group.id.11317 | CD | MRPRESSO | NA(NA,NA) | NA |
| genus.LachnospiraceaeNK4A136group.id.11319 | CD | IVW | 0.816(0.664,1.002) | 0.052 |
| genus.LachnospiraceaeNK4A136group.id.11319 | CD | Weighted median | 0.824(0.619,1.096) | 0.184 |
| genus.LachnospiraceaeNK4A136group.id.11319 | CD | Weighted mode | 0.967(0.647,1.444) | 0.869 |
| genus.LachnospiraceaeNK4A136group.id.11319 | CD | MR-Robust | 0.809(0.651,1.006) | 0.057 |
| genus.LachnospiraceaeNK4A136group.id.11319 | CD | MR-Egger | 1.02(0.665,1.565) | 0.928 |
| genus.LachnospiraceaeNK4A136group.id.11319 | CD | MRRAPS | 0.811(0.645,1.018) | 0.071 |
| genus.LachnospiraceaeNK4A136group.id.11319 | CD | MRPRESSO | 0.816(0.716,0.929) | 0.008 |
| genus.LachnospiraceaeUCG001.id.11321 | CD | IVW | 0.781(0.637,0.958) | 0.018 |
| genus.LachnospiraceaeUCG001.id.11321 | CD | Weighted median | 0.711(0.536,0.942) | 0.017 |
| genus.LachnospiraceaeUCG001.id.11321 | CD | Weighted mode | 0.698(0.435,1.119) | 0.135 |
| genus.LachnospiraceaeUCG001.id.11321 | CD | MR-Robust | 0.782(0.628,0.972) | 0.027 |
| genus.LachnospiraceaeUCG001.id.11321 | CD | MR-Egger | 1.134(0.422,3.05) | 0.803 |
| genus.LachnospiraceaeUCG001.id.11321 | CD | MRRAPS | 0.78(0.625,0.974) | 0.029 |
| genus.LachnospiraceaeUCG001.id.11321 | CD | MRPRESSO | 0.781(0.648,0.942) | 0.025 |
| genus.LachnospiraceaeUCG004.id.11324 | CD | IVW | 0.976(0.781,1.22) | 0.831 |
| genus.LachnospiraceaeUCG004.id.11324 | CD | Weighted median | 1.008(0.753,1.349) | 0.959 |
| genus.LachnospiraceaeUCG004.id.11324 | CD | Weighted mode | 1.029(0.636,1.665) | 0.907 |
| genus.LachnospiraceaeUCG004.id.11324 | CD | MR-Robust | 0.966(0.79,1.182) | 0.738 |
| genus.LachnospiraceaeUCG004.id.11324 | CD | MR-Egger | 1.082(0.442,2.647) | 0.864 |
| genus.LachnospiraceaeUCG004.id.11324 | CD | MRRAPS | 0.967(0.758,1.233) | 0.784 |
| genus.LachnospiraceaeUCG004.id.11324 | CD | MRPRESSO | 0.976(0.813,1.172) | 0.799 |
| genus.LachnospiraceaeUCG008.id.11328 | CD | IVW | 1.042(0.893,1.216) | 0.598 |
| genus.LachnospiraceaeUCG008.id.11328 | CD | Weighted median | 1.024(0.834,1.258) | 0.819 |
| genus.LachnospiraceaeUCG008.id.11328 | CD | Weighted mode | 1.055(0.751,1.484) | 0.756 |
| genus.LachnospiraceaeUCG008.id.11328 | CD | MR-Robust | 0.999(0.854,1.168) | 0.987 |
| genus.LachnospiraceaeUCG008.id.11328 | CD | MR-Egger | 1.396(0.618,3.154) | 0.422 |
| genus.LachnospiraceaeUCG008.id.11328 | CD | MRRAPS | 1.015(0.859,1.198) | 0.864 |
| genus.LachnospiraceaeUCG008.id.11328 | CD | MRPRESSO | 1.042(0.906,1.199) | 0.571 |
| genus.LachnospiraceaeUCG010.id.11330 | CD | IVW | 1.205(0.89,1.632) | 0.228 |
| genus.LachnospiraceaeUCG010.id.11330 | CD | Weighted median | 1.308(0.946,1.808) | 0.104 |
| genus.LachnospiraceaeUCG010.id.11330 | CD | Weighted mode | 1.46(0.941,2.266) | 0.091 |
| genus.LachnospiraceaeUCG010.id.11330 | CD | MR-Robust | 1.291(0.919,1.814) | 0.140 |
| genus.LachnospiraceaeUCG010.id.11330 | CD | MR-Egger | 1.359(0.595,3.101) | 0.467 |
| genus.LachnospiraceaeUCG010.id.11330 | CD | MRRAPS | 1.26(0.829,1.914) | 0.279 |
| genus.LachnospiraceaeUCG010.id.11330 | CD | MRPRESSO | 1.205(0.89,1.632) | 0.259 |
| genus.Lactobacillus.id.1837 | CD | IVW | 0.839(0.691,1.017) | 0.074 |
| genus.Lactobacillus.id.1837 | CD | Weighted median | 0.849(0.663,1.087) | 0.195 |
| genus.Lactobacillus.id.1837 | CD | Weighted mode | 0.846(0.596,1.199) | 0.347 |
| genus.Lactobacillus.id.1837 | CD | MR-Robust | 0.861(0.563,1.317) | 0.490 |
| genus.Lactobacillus.id.1837 | CD | MR-Egger | 1.023(0.552,1.898) | 0.942 |
| genus.Lactobacillus.id.1837 | CD | MRRAPS | 0.864(0.708,1.055) | 0.152 |
| genus.Lactobacillus.id.1837 | CD | MRPRESSO | 0.839(0.691,1.017) | 0.108 |
| genus.Lactococcus.id.1851 | CD | IVW | 0.994(0.85,1.162) | 0.936 |
| genus.Lactococcus.id.1851 | CD | Weighted median | 0.937(0.765,1.149) | 0.534 |
| genus.Lactococcus.id.1851 | CD | Weighted mode | 0.861(0.63,1.178) | 0.350 |
| genus.Lactococcus.id.1851 | CD | MR-Robust | 0.968(0.809,1.159) | 0.727 |
| genus.Lactococcus.id.1851 | CD | MR-Egger | 1.198(0.586,2.45) | 0.621 |
| genus.Lactococcus.id.1851 | CD | MRRAPS | 0.966(0.824,1.131) | 0.665 |
| genus.Lactococcus.id.1851 | CD | MRPRESSO | 0.994(0.85,1.162) | 0.938 |
| genus.Marvinbryantia.id.2005 | CD | IVW | 0.965(0.768,1.212) | 0.757 |
| genus.Marvinbryantia.id.2005 | CD | Weighted median | 1.017(0.741,1.395) | 0.916 |
| genus.Marvinbryantia.id.2005 | CD | Weighted mode | 1.055(0.623,1.787) | 0.841 |
| genus.Marvinbryantia.id.2005 | CD | MR-Robust | 0.972(0.772,1.224) | 0.807 |
| genus.Marvinbryantia.id.2005 | CD | MR-Egger | 0.989(0.418,2.341) | 0.980 |
| genus.Marvinbryantia.id.2005 | CD | MRRAPS | 0.966(0.754,1.237) | 0.783 |
| genus.Marvinbryantia.id.2005 | CD | MRPRESSO | 0.965(0.791,1.177) | 0.730 |
| genus.Methanobrevibacter.id.123 | CD | IVW | 1.064(0.807,1.403) | 0.659 |
| genus.Methanobrevibacter.id.123 | CD | Weighted median | 1.091(0.857,1.389) | 0.481 |
| genus.Methanobrevibacter.id.123 | CD | Weighted mode | 1.098(0.77,1.565) | 0.607 |
| genus.Methanobrevibacter.id.123 | CD | MR-Robust | 1.071(0.823,1.395) | 0.609 |
| genus.Methanobrevibacter.id.123 | CD | MR-Egger | 0.701(0.234,2.101) | 0.526 |
| genus.Methanobrevibacter.id.123 | CD | MRRAPS | 1.058(0.831,1.346) | 0.649 |
| genus.Methanobrevibacter.id.123 | CD | MRPRESSO | 1.064(0.807,1.403) | 0.675 |
| genus.Odoribacter.id.952 | CD | IVW | 1.515(1.093,2.099) | 0.013 |
| genus.Odoribacter.id.952 | CD | Weighted median | 1.482(0.962,2.283) | 0.074 |
| genus.Odoribacter.id.952 | CD | Weighted mode | 1.632(0.852,3.128) | 0.140 |
| genus.Odoribacter.id.952 | CD | MR-Robust | 1.518(1.187,1.941) | 0.001 |
| genus.Odoribacter.id.952 | CD | MR-Egger | 1.398(0.486,4.022) | 0.534 |
| genus.Odoribacter.id.952 | CD | MRRAPS | 1.527(1.061,2.197) | 0.023 |
| genus.Odoribacter.id.952 | CD | MRPRESSO | 1.515(1.196,1.919) | 0.014 |
| genus.Olsenella.id.822 | CD | IVW | 0.918(0.799,1.054) | 0.227 |
| genus.Olsenella.id.822 | CD | Weighted median | 0.86(0.715,1.035) | 0.110 |
| genus.Olsenella.id.822 | CD | Weighted mode | 0.842(0.616,1.152) | 0.282 |
| genus.Olsenella.id.822 | CD | MR-Robust | 0.912(0.779,1.068) | 0.254 |
| genus.Olsenella.id.822 | CD | MR-Egger | 0.579(0.346,0.968) | 0.037 |
| genus.Olsenella.id.822 | CD | MRRAPS | 0.912(0.786,1.057) | 0.220 |
| genus.Olsenella.id.822 | CD | MRPRESSO | 0.918(0.799,1.054) | 0.255 |
| genus.Oscillibacter.id.2063 | CD | IVW | 0.973(0.812,1.166) | 0.767 |
| genus.Oscillibacter.id.2063 | CD | Weighted median | 1.024(0.801,1.31) | 0.849 |
| genus.Oscillibacter.id.2063 | CD | Weighted mode | 1.031(0.693,1.535) | 0.878 |
| genus.Oscillibacter.id.2063 | CD | MR-Robust | 1.027(0.778,1.357) | 0.851 |
| genus.Oscillibacter.id.2063 | CD | MR-Egger | 1.075(0.522,2.213) | 0.845 |
| genus.Oscillibacter.id.2063 | CD | MRRAPS | 1.002(0.828,1.213) | 0.983 |
| genus.Oscillibacter.id.2063 | CD | MRPRESSO | 0.973(0.812,1.166) | 0.772 |
| genus.Oscillospira.id.2064 | CD | IVW | 0.862(0.646,1.15) | 0.312 |
| genus.Oscillospira.id.2064 | CD | Weighted median | 0.885(0.621,1.262) | 0.500 |
| genus.Oscillospira.id.2064 | CD | Weighted mode | 1.053(0.581,1.909) | 0.864 |
| genus.Oscillospira.id.2064 | CD | MR-Robust | 0.863(0.642,1.161) | 0.331 |
| genus.Oscillospira.id.2064 | CD | MR-Egger | 0.376(0.117,1.21) | 0.101 |
| genus.Oscillospira.id.2064 | CD | MRRAPS | 0.834(0.603,1.154) | 0.274 |
| genus.Oscillospira.id.2064 | CD | MRPRESSO | 0.862(0.646,1.15) | 0.342 |
| genus.Oxalobacter.id.2978 | CD | IVW | 1.292(1.126,1.482) | 0.000 |
| genus.Oxalobacter.id.2978 | CD | Weighted median | 1.3(1.072,1.576) | 0.008 |
| genus.Oxalobacter.id.2978 | CD | Weighted mode | 1.287(0.961,1.725) | 0.091 |
| genus.Oxalobacter.id.2978 | CD | MR-Robust | 1.274(1.096,1.481) | 0.002 |
| genus.Oxalobacter.id.2978 | CD | MR-Egger | 0.675(0.356,1.28) | 0.229 |
| genus.Oxalobacter.id.2978 | CD | MRRAPS | 1.287(1.106,1.497) | 0.001 |
| genus.Oxalobacter.id.2978 | CD | MRPRESSO | 1.292(1.14,1.465) | 0.003 |
| genus.Parabacteroides.id.954 | CD | IVW | 0.993(0.709,1.391) | 0.968 |
| genus.Parabacteroides.id.954 | CD | Weighted median | 1.096(0.708,1.695) | 0.682 |
| genus.Parabacteroides.id.954 | CD | Weighted mode | 1.174(0.657,2.097) | 0.588 |
| genus.Parabacteroides.id.954 | CD | MR-Robust | 1.004(0.723,1.394) | 0.981 |
| genus.Parabacteroides.id.954 | CD | MR-Egger | 1.883(0.648,5.467) | 0.245 |
| genus.Parabacteroides.id.954 | CD | MRRAPS | 0.993(0.689,1.432) | 0.970 |
| genus.Parabacteroides.id.954 | CD | MRPRESSO | 0.993(0.758,1.302) | 0.962 |
| genus.Paraprevotella.id.962 | CD | IVW | 0.969(0.82,1.145) | 0.709 |
| genus.Paraprevotella.id.962 | CD | Weighted median | 0.87(0.701,1.079) | 0.205 |
| genus.Paraprevotella.id.962 | CD | Weighted mode | 0.841(0.603,1.172) | 0.306 |
| genus.Paraprevotella.id.962 | CD | MR-Robust | 0.917(0.642,1.309) | 0.633 |
| genus.Paraprevotella.id.962 | CD | MR-Egger | 0.868(0.479,1.573) | 0.641 |
| genus.Paraprevotella.id.962 | CD | MRRAPS | 0.94(0.793,1.115) | 0.479 |
| genus.Paraprevotella.id.962 | CD | MRPRESSO | 0.969(0.82,1.145) | 0.716 |
| genus.Parasutterella.id.2892 | CD | IVW | 1.231(1.026,1.477) | 0.025 |
| genus.Parasutterella.id.2892 | CD | Weighted median | 1.094(0.854,1.402) | 0.478 |
| genus.Parasutterella.id.2892 | CD | Weighted mode | 1.054(0.737,1.507) | 0.773 |
| genus.Parasutterella.id.2892 | CD | MR-Robust | 1.169(0.93,1.468) | 0.180 |
| genus.Parasutterella.id.2892 | CD | MR-Egger | 1.06(0.608,1.848) | 0.838 |
| genus.Parasutterella.id.2892 | CD | MRRAPS | 1.201(0.985,1.465) | 0.070 |
| genus.Parasutterella.id.2892 | CD | MRPRESSO | 1.231(1.034,1.466) | 0.035 |
| genus.Peptococcus.id.2037 | CD | IVW | 1.103(0.957,1.271) | 0.177 |
| genus.Peptococcus.id.2037 | CD | Weighted median | 1.111(0.914,1.351) | 0.289 |
| genus.Peptococcus.id.2037 | CD | Weighted mode | 1.117(0.806,1.548) | 0.506 |
| genus.Peptococcus.id.2037 | CD | MR-Robust | 1.1(0.97,1.249) | 0.139 |
| genus.Peptococcus.id.2037 | CD | MR-Egger | 1.225(0.707,2.121) | 0.469 |
| genus.Peptococcus.id.2037 | CD | MRRAPS | 1.102(0.945,1.286) | 0.215 |
| genus.Peptococcus.id.2037 | CD | MRPRESSO | 1.103(0.969,1.255) | 0.161 |
| genus.Phascolarctobacterium.id.2168 | CD | IVW | 1.144(0.912,1.435) | 0.245 |
| genus.Phascolarctobacterium.id.2168 | CD | Weighted median | 1.233(0.907,1.677) | 0.181 |
| genus.Phascolarctobacterium.id.2168 | CD | Weighted mode | 1.298(0.801,2.104) | 0.289 |
| genus.Phascolarctobacterium.id.2168 | CD | MR-Robust | 1.17(0.935,1.463) | 0.169 |
| genus.Phascolarctobacterium.id.2168 | CD | MR-Egger | 2.532(0.913,7.028) | 0.074 |
| genus.Phascolarctobacterium.id.2168 | CD | MRRAPS | 1.177(0.92,1.507) | 0.194 |
| genus.Phascolarctobacterium.id.2168 | CD | MRPRESSO | 1.144(0.926,1.413) | 0.240 |
| genus.Prevotella7.id.11182 | CD | IVW | 1.028(0.904,1.17) | 0.675 |
| genus.Prevotella7.id.11182 | CD | Weighted median | 1.097(0.914,1.318) | 0.319 |
| genus.Prevotella7.id.11182 | CD | Weighted mode | 1.183(0.841,1.664) | 0.333 |
| genus.Prevotella7.id.11182 | CD | MR-Robust | 1.03(0.898,1.181) | 0.678 |
| genus.Prevotella7.id.11182 | CD | MR-Egger | 1.43(0.669,3.058) | 0.356 |
| genus.Prevotella7.id.11182 | CD | MRRAPS | 1.029(0.89,1.19) | 0.702 |
| genus.Prevotella7.id.11182 | CD | MRPRESSO | 1.028(0.907,1.165) | 0.675 |
| genus.Prevotella9.id.11183 | CD | IVW | 1.09(0.88,1.349) | 0.430 |
| genus.Prevotella9.id.11183 | CD | Weighted median | 1.052(0.8,1.382) | 0.718 |
| genus.Prevotella9.id.11183 | CD | Weighted mode | 0.797(0.471,1.35) | 0.399 |
| genus.Prevotella9.id.11183 | CD | MR-Robust | 1.091(0.86,1.383) | 0.474 |
| genus.Prevotella9.id.11183 | CD | MR-Egger | 0.673(0.349,1.299) | 0.238 |
| genus.Prevotella9.id.11183 | CD | MRRAPS | 1.056(0.825,1.353) | 0.665 |
| genus.Prevotella9.id.11183 | CD | MRPRESSO | 1.09(0.88,1.349) | 0.443 |
| genus.RikenellaceaeRC9gutgroup.id.11191 | CD | IVW | 1.169(1.031,1.326) | 0.015 |
| genus.RikenellaceaeRC9gutgroup.id.11191 | CD | Weighted median | 1.199(1.011,1.422) | 0.037 |
| genus.RikenellaceaeRC9gutgroup.id.11191 | CD | Weighted mode | 1.253(0.9,1.745) | 0.181 |
| genus.RikenellaceaeRC9gutgroup.id.11191 | CD | MR-Robust | 1.169(1.023,1.336) | 0.022 |
| genus.RikenellaceaeRC9gutgroup.id.11191 | CD | MR-Egger | 1.905(0.904,4.013) | 0.090 |
| genus.RikenellaceaeRC9gutgroup.id.11191 | CD | MRRAPS | 1.181(1.021,1.366) | 0.025 |
| genus.RikenellaceaeRC9gutgroup.id.11191 | CD | MRPRESSO | 1.169(1.031,1.326) | 0.032 |
| genus.Romboutsia.id.11347 | CD | IVW | 1.013(0.781,1.312) | 0.925 |
| genus.Romboutsia.id.11347 | CD | Weighted median | 1.023(0.76,1.377) | 0.879 |
| genus.Romboutsia.id.11347 | CD | Weighted mode | 0.942(0.614,1.444) | 0.784 |
| genus.Romboutsia.id.11347 | CD | MR-Robust | 1.042(0.829,1.311) | 0.723 |
| genus.Romboutsia.id.11347 | CD | MR-Egger | 1.244(0.615,2.515) | 0.543 |
| genus.Romboutsia.id.11347 | CD | MRRAPS | 1.029(0.822,1.287) | 0.805 |
| genus.Romboutsia.id.11347 | CD | MRPRESSO | 1.013(0.781,1.312) | 0.926 |
| genus.Roseburia.id.2012 | CD | IVW | 1.006(0.784,1.292) | 0.961 |
| genus.Roseburia.id.2012 | CD | Weighted median | 1.178(0.848,1.637) | 0.328 |
| genus.Roseburia.id.2012 | CD | Weighted mode | 1.304(0.818,2.08) | 0.265 |
| genus.Roseburia.id.2012 | CD | MR-Robust | 1.125(0.8,1.582) | 0.497 |
| genus.Roseburia.id.2012 | CD | MR-Egger | 1.534(0.736,3.201) | 0.254 |
| genus.Roseburia.id.2012 | CD | MRRAPS | 1.071(0.828,1.384) | 0.603 |
| genus.Roseburia.id.2012 | CD | MRPRESSO | 1.006(0.784,1.292) | 0.961 |
| genus.Ruminiclostridium5.id.11355 | CD | IVW | 0.731(0.458,1.167) | 0.189 |
| genus.Ruminiclostridium5.id.11355 | CD | Weighted median | 0.762(0.49,1.187) | 0.229 |
| genus.Ruminiclostridium5.id.11355 | CD | Weighted mode | 1.012(0.516,1.985) | 0.971 |
| genus.Ruminiclostridium5.id.11355 | CD | MR-Robust | 0.766(0.463,1.267) | 0.300 |
| genus.Ruminiclostridium5.id.11355 | CD | MR-Egger | 2.469(0.387,15.755) | 0.339 |
| genus.Ruminiclostridium5.id.11355 | CD | MRRAPS | 0.844(0.523,1.363) | 0.489 |
| genus.Ruminiclostridium5.id.11355 | CD | MRPRESSO | 0.731(0.458,1.167) | 0.219 |
| genus.Ruminiclostridium6.id.11356 | CD | IVW | 1.023(0.732,1.429) | 0.896 |
| genus.Ruminiclostridium6.id.11356 | CD | Weighted median | 0.883(0.668,1.166) | 0.380 |
| genus.Ruminiclostridium6.id.11356 | CD | Weighted mode | 0.818(0.551,1.213) | 0.318 |
| genus.Ruminiclostridium6.id.11356 | CD | MR-Robust | 0.885(0.679,1.154) | 0.365 |
| genus.Ruminiclostridium6.id.11356 | CD | MR-Egger | 0.559(0.249,1.254) | 0.158 |
| genus.Ruminiclostridium6.id.11356 | CD | MRRAPS | 0.923(0.73,1.169) | 0.507 |
| genus.Ruminiclostridium6.id.11356 | CD | MRPRESSO | 1.023(0.732,1.429) | 0.898 |
| genus.Ruminiclostridium9.id.11357 | CD | IVW | 1.096(0.744,1.614) | 0.643 |
| genus.Ruminiclostridium9.id.11357 | CD | Weighted median | 1.287(0.852,1.944) | 0.230 |
| genus.Ruminiclostridium9.id.11357 | CD | Weighted mode | 1.512(0.7,3.266) | 0.293 |
| genus.Ruminiclostridium9.id.11357 | CD | MR-Robust | 1.106(0.719,1.702) | 0.646 |
| genus.Ruminiclostridium9.id.11357 | CD | MR-Egger | 0.874(0.145,5.27) | 0.884 |
| genus.Ruminiclostridium9.id.11357 | CD | MRRAPS | 1.058(0.654,1.713) | 0.817 |
| genus.Ruminiclostridium9.id.11357 | CD | MRPRESSO | 1.096(0.744,1.614) | 0.654 |
| genus.RuminococcaceaeNK4A214group.id.11358 | CD | IVW | 0.838(0.644,1.092) | 0.191 |
| genus.RuminococcaceaeNK4A214group.id.11358 | CD | Weighted median | 0.861(0.622,1.192) | 0.368 |
| genus.RuminococcaceaeNK4A214group.id.11358 | CD | Weighted mode | 0.914(0.52,1.607) | 0.756 |
| genus.RuminococcaceaeNK4A214group.id.11358 | CD | MR-Robust | 0.831(0.639,1.081) | 0.167 |
| genus.RuminococcaceaeNK4A214group.id.11358 | CD | MR-Egger | 0.484(0.187,1.255) | 0.135 |
| genus.RuminococcaceaeNK4A214group.id.11358 | CD | MRRAPS | 0.809(0.614,1.066) | 0.132 |
| genus.RuminococcaceaeNK4A214group.id.11358 | CD | MRPRESSO | 0.838(0.644,1.092) | 0.212 |
| genus.RuminococcaceaeUCG002.id.11360 | CD | IVW | 0.952(0.793,1.143) | 0.599 |
| genus.RuminococcaceaeUCG002.id.11360 | CD | Weighted median | 0.876(0.675,1.137) | 0.320 |
| genus.RuminococcaceaeUCG002.id.11360 | CD | Weighted mode | 0.744(0.438,1.263) | 0.274 |
| genus.RuminococcaceaeUCG002.id.11360 | CD | MR-Robust | 0.958(0.785,1.17) | 0.675 |
| genus.RuminococcaceaeUCG002.id.11360 | CD | MR-Egger | 0.62(0.37,1.038) | 0.069 |
| genus.RuminococcaceaeUCG002.id.11360 | CD | MRRAPS | 0.946(0.771,1.16) | 0.593 |
| genus.RuminococcaceaeUCG002.id.11360 | CD | MRPRESSO | 0.952(0.804,1.127) | 0.575 |
| genus.RuminococcaceaeUCG003.id.11361 | CD | IVW | 1.033(0.661,1.616) | 0.885 |
| genus.RuminococcaceaeUCG003.id.11361 | CD | Weighted median | 0.815(0.587,1.13) | 0.220 |
| genus.RuminococcaceaeUCG003.id.11361 | CD | Weighted mode | 0.685(0.421,1.114) | 0.128 |
| genus.RuminococcaceaeUCG003.id.11361 | CD | MR-Robust | 0.837(0.634,1.105) | 0.210 |
| genus.RuminococcaceaeUCG003.id.11361 | CD | MR-Egger | 0.383(0.093,1.574) | 0.183 |
| genus.RuminococcaceaeUCG003.id.11361 | CD | MRRAPS | 0.86(0.634,1.167) | 0.332 |
| genus.RuminococcaceaeUCG003.id.11361 | CD | MRPRESSO | 1.033(0.661,1.616) | 0.888 |
| genus.RuminococcaceaeUCG004.id.11362 | CD | IVW | 0.951(0.777,1.164) | 0.628 |
| genus.RuminococcaceaeUCG004.id.11362 | CD | Weighted median | 1.009(0.779,1.307) | 0.944 |
| genus.RuminococcaceaeUCG004.id.11362 | CD | Weighted mode | 1.075(0.706,1.636) | 0.737 |
| genus.RuminococcaceaeUCG004.id.11362 | CD | MR-Robust | 0.964(0.755,1.23) | 0.766 |
| genus.RuminococcaceaeUCG004.id.11362 | CD | MR-Egger | 1.09(0.356,3.336) | 0.880 |
| genus.RuminococcaceaeUCG004.id.11362 | CD | MRRAPS | 0.95(0.762,1.185) | 0.652 |
| genus.RuminococcaceaeUCG004.id.11362 | CD | MRPRESSO | 0.951(0.836,1.083) | 0.467 |
| genus.RuminococcaceaeUCG005.id.11363 | CD | IVW | 1.022(0.821,1.272) | 0.847 |
| genus.RuminococcaceaeUCG005.id.11363 | CD | Weighted median | 1.043(0.777,1.399) | 0.781 |
| genus.RuminococcaceaeUCG005.id.11363 | CD | Weighted mode | 1.074(0.696,1.657) | 0.746 |
| genus.RuminococcaceaeUCG005.id.11363 | CD | MR-Robust | 0.975(0.807,1.179) | 0.798 |
| genus.RuminococcaceaeUCG005.id.11363 | CD | MR-Egger | 0.879(0.466,1.658) | 0.690 |
| genus.RuminococcaceaeUCG005.id.11363 | CD | MRRAPS | 0.987(0.779,1.251) | 0.915 |
| genus.RuminococcaceaeUCG005.id.11363 | CD | MRPRESSO | 1.022(0.826,1.264) | 0.846 |
| genus.RuminococcaceaeUCG009.id.11366 | CD | IVW | 0.769(0.645,0.919) | 0.004 |
| genus.RuminococcaceaeUCG009.id.11366 | CD | Weighted median | 0.783(0.618,0.993) | 0.043 |
| genus.RuminococcaceaeUCG009.id.11366 | CD | Weighted mode | 0.847(0.574,1.251) | 0.404 |
| genus.RuminococcaceaeUCG009.id.11366 | CD | MR-Robust | 0.772(0.662,0.901) | 0.001 |
| genus.RuminococcaceaeUCG009.id.11366 | CD | MR-Egger | 0.816(0.375,1.773) | 0.607 |
| genus.RuminococcaceaeUCG009.id.11366 | CD | MRRAPS | 0.764(0.628,0.93) | 0.007 |
| genus.RuminococcaceaeUCG009.id.11366 | CD | MRPRESSO | 0.769(0.661,0.896) | 0.006 |
| genus.RuminococcaceaeUCG010.id.11367 | CD | IVW | 1.33(0.976,1.812) | 0.071 |
| genus.RuminococcaceaeUCG010.id.11367 | CD | Weighted median | 1.391(0.934,2.07) | 0.104 |
| genus.RuminococcaceaeUCG010.id.11367 | CD | Weighted mode | 1.629(0.872,3.044) | 0.126 |
| genus.RuminococcaceaeUCG010.id.11367 | CD | MR-Robust | 1.335(1.023,1.743) | 0.033 |
| genus.RuminococcaceaeUCG010.id.11367 | CD | MR-Egger | 2.002(0.716,5.597) | 0.186 |
| genus.RuminococcaceaeUCG010.id.11367 | CD | MRRAPS | 1.34(0.954,1.883) | 0.092 |
| genus.RuminococcaceaeUCG010.id.11367 | CD | MRPRESSO | 1.33(1.022,1.731) | 0.088 |
| genus.RuminococcaceaeUCG011.id.11368 | CD | IVW | 0.99(0.861,1.138) | 0.884 |
| genus.RuminococcaceaeUCG011.id.11368 | CD | Weighted median | 0.952(0.793,1.142) | 0.595 |
| genus.RuminococcaceaeUCG011.id.11368 | CD | Weighted mode | 0.927(0.708,1.214) | 0.583 |
| genus.RuminococcaceaeUCG011.id.11368 | CD | MR-Robust | 0.954(0.768,1.184) | 0.667 |
| genus.RuminococcaceaeUCG011.id.11368 | CD | MR-Egger | 1.013(0.497,2.067) | 0.972 |
| genus.RuminococcaceaeUCG011.id.11368 | CD | MRRAPS | 0.977(0.841,1.136) | 0.766 |
| genus.RuminococcaceaeUCG011.id.11368 | CD | MRPRESSO | 0.99(0.879,1.115) | 0.869 |
| genus.RuminococcaceaeUCG013.id.11370 | CD | IVW | 0.844(0.66,1.078) | 0.173 |
| genus.RuminococcaceaeUCG013.id.11370 | CD | Weighted median | 0.755(0.539,1.058) | 0.102 |
| genus.RuminococcaceaeUCG013.id.11370 | CD | Weighted mode | 0.651(0.351,1.209) | 0.174 |
| genus.RuminococcaceaeUCG013.id.11370 | CD | MR-Robust | 0.851(0.643,1.126) | 0.259 |
| genus.RuminococcaceaeUCG013.id.11370 | CD | MR-Egger | 0.63(0.319,1.244) | 0.183 |
| genus.RuminococcaceaeUCG013.id.11370 | CD | MRRAPS | 0.832(0.612,1.13) | 0.239 |
| genus.RuminococcaceaeUCG013.id.11370 | CD | MRPRESSO | 0.844(0.665,1.071) | 0.189 |
| genus.RuminococcaceaeUCG014.id.11371 | CD | IVW | 1.403(1.105,1.781) | 0.005 |
| genus.RuminococcaceaeUCG014.id.11371 | CD | Weighted median | 1.487(1.089,2.032) | 0.013 |
| genus.RuminococcaceaeUCG014.id.11371 | CD | Weighted mode | 1.549(0.94,2.554) | 0.086 |
| genus.RuminococcaceaeUCG014.id.11371 | CD | MR-Robust | 1.416(1.095,1.831) | 0.008 |
| genus.RuminococcaceaeUCG014.id.11371 | CD | MR-Egger | 1.35(0.678,2.688) | 0.393 |
| genus.RuminococcaceaeUCG014.id.11371 | CD | MRRAPS | 1.411(1.082,1.839) | 0.011 |
| genus.RuminococcaceaeUCG014.id.11371 | CD | MRPRESSO | 1.403(1.205,1.634) | 0.001 |
| genus.Ruminococcus1.id.11373 | CD | IVW | 0.985(0.696,1.392) | 0.930 |
| genus.Ruminococcus1.id.11373 | CD | Weighted median | 0.876(0.584,1.313) | 0.520 |
| genus.Ruminococcus1.id.11373 | CD | Weighted mode | 0.832(0.42,1.649) | 0.599 |
| genus.Ruminococcus1.id.11373 | CD | MR-Robust | 0.97(0.665,1.413) | 0.872 |
| genus.Ruminococcus1.id.11373 | CD | MR-Egger | 1.021(0.358,2.915) | 0.969 |
| genus.Ruminococcus1.id.11373 | CD | MRRAPS | 0.974(0.683,1.39) | 0.886 |
| genus.Ruminococcus1.id.11373 | CD | MRPRESSO | 0.985(0.696,1.392) | 0.932 |
| genus.Ruminococcus2.id.11374 | CD | IVW | 1.048(0.845,1.299) | 0.669 |
| genus.Ruminococcus2.id.11374 | CD | Weighted median | 1.012(0.747,1.372) | 0.937 |
| genus.Ruminococcus2.id.11374 | CD | Weighted mode | 1.033(0.63,1.695) | 0.897 |
| genus.Ruminococcus2.id.11374 | CD | MR-Robust | 1.049(0.849,1.297) | 0.657 |
| genus.Ruminococcus2.id.11374 | CD | MR-Egger | 0.898(0.502,1.607) | 0.718 |
| genus.Ruminococcus2.id.11374 | CD | MRRAPS | 1.041(0.825,1.314) | 0.733 |
| genus.Ruminococcus2.id.11374 | CD | MRPRESSO | 1.048(0.854,1.286) | 0.660 |
| genus.Sellimonas.id.14369 | CD | IVW | 0.919(0.786,1.075) | 0.290 |
| genus.Sellimonas.id.14369 | CD | Weighted median | 0.891(0.747,1.064) | 0.202 |
| genus.Sellimonas.id.14369 | CD | Weighted mode | 0.916(0.675,1.245) | 0.576 |
| genus.Sellimonas.id.14369 | CD | MR-Robust | 0.919(0.783,1.078) | 0.299 |
| genus.Sellimonas.id.14369 | CD | MR-Egger | 0.599(0.288,1.247) | 0.171 |
| genus.Sellimonas.id.14369 | CD | MRRAPS | 0.903(0.76,1.072) | 0.244 |
| genus.Sellimonas.id.14369 | CD | MRPRESSO | 0.919(0.786,1.075) | 0.315 |
| genus.Senegalimassilia.id.11160 | CD | IVW | 1.023(0.665,1.574) | 0.916 |
| genus.Senegalimassilia.id.11160 | CD | Weighted median | 0.883(0.61,1.278) | 0.509 |
| genus.Senegalimassilia.id.11160 | CD | Weighted mode | 0.814(0.517,1.282) | 0.375 |
| genus.Senegalimassilia.id.11160 | CD | MR-Robust | 1.01(0.665,1.536) | 0.961 |
| genus.Senegalimassilia.id.11160 | CD | MR-Egger | 0.591(0.123,2.846) | 0.511 |
| genus.Senegalimassilia.id.11160 | CD | MRRAPS | 0.955(0.677,1.348) | 0.793 |
| genus.Senegalimassilia.id.11160 | CD | MRPRESSO | 1.023(0.665,1.574) | 0.921 |
| genus.Slackia.id.825 | CD | IVW | 0.992(0.797,1.235) | 0.943 |
| genus.Slackia.id.825 | CD | Weighted median | 0.958(0.725,1.264) | 0.761 |
| genus.Slackia.id.825 | CD | Weighted mode | 0.907(0.584,1.407) | 0.663 |
| genus.Slackia.id.825 | CD | MR-Robust | 0.989(0.802,1.221) | 0.921 |
| genus.Slackia.id.825 | CD | MR-Egger | 0.543(0.15,1.965) | 0.352 |
| genus.Slackia.id.825 | CD | MRRAPS | 0.988(0.78,1.251) | 0.917 |
| genus.Slackia.id.825 | CD | MRPRESSO | 0.992(0.803,1.226) | 0.944 |
| genus.Streptococcus.id.1853 | CD | IVW | 0.888(0.718,1.097) | 0.271 |
| genus.Streptococcus.id.1853 | CD | Weighted median | 0.761(0.566,1.023) | 0.070 |
| genus.Streptococcus.id.1853 | CD | Weighted mode | 0.675(0.362,1.259) | 0.217 |
| genus.Streptococcus.id.1853 | CD | MR-Robust | 0.868(0.662,1.139) | 0.309 |
| genus.Streptococcus.id.1853 | CD | MR-Egger | 0.967(0.435,2.149) | 0.935 |
| genus.Streptococcus.id.1853 | CD | MRRAPS | 0.858(0.675,1.092) | 0.214 |
| genus.Streptococcus.id.1853 | CD | MRPRESSO | 0.888(0.718,1.097) | 0.286 |
| genus.Subdoligranulum.id.2070 | CD | IVW | 1.013(0.776,1.323) | 0.923 |
| genus.Subdoligranulum.id.2070 | CD | Weighted median | 0.985(0.695,1.395) | 0.931 |
| genus.Subdoligranulum.id.2070 | CD | Weighted mode | 0.942(0.561,1.582) | 0.822 |
| genus.Subdoligranulum.id.2070 | CD | MR-Robust | 0.995(0.767,1.29) | 0.969 |
| genus.Subdoligranulum.id.2070 | CD | MR-Egger | 0.862(0.425,1.749) | 0.682 |
| genus.Subdoligranulum.id.2070 | CD | MRRAPS | 0.999(0.747,1.336) | 0.996 |
| genus.Subdoligranulum.id.2070 | CD | MRPRESSO | 1.013(0.812,1.264) | 0.910 |
| genus.Sutterella.id.2896 | CD | IVW | 0.981(0.781,1.233) | 0.868 |
| genus.Sutterella.id.2896 | CD | Weighted median | 1.017(0.755,1.37) | 0.910 |
| genus.Sutterella.id.2896 | CD | Weighted mode | 1.011(0.639,1.597) | 0.964 |
| genus.Sutterella.id.2896 | CD | MR-Robust | 1.041(0.84,1.289) | 0.716 |
| genus.Sutterella.id.2896 | CD | MR-Egger | 0.816(0.26,2.563) | 0.728 |
| genus.Sutterella.id.2896 | CD | MRRAPS | 1.015(0.792,1.3) | 0.909 |
| genus.Sutterella.id.2896 | CD | MRPRESSO | 0.981(0.801,1.201) | 0.855 |
| genus.Terrisporobacter.id.11348 | CD | IVW | 0.946(0.745,1.201) | 0.649 |
| genus.Terrisporobacter.id.11348 | CD | Weighted median | 0.935(0.697,1.255) | 0.657 |
| genus.Terrisporobacter.id.11348 | CD | Weighted mode | 0.908(0.622,1.324) | 0.616 |
| genus.Terrisporobacter.id.11348 | CD | MR-Robust | 0.938(0.765,1.15) | 0.538 |
| genus.Terrisporobacter.id.11348 | CD | MR-Egger | 0.932(0.483,1.797) | 0.833 |
| genus.Terrisporobacter.id.11348 | CD | MRRAPS | 0.945(0.728,1.227) | 0.671 |
| genus.Terrisporobacter.id.11348 | CD | MRPRESSO | 0.946(0.792,1.13) | 0.573 |
| genus.Turicibacter.id.2162 | CD | IVW | 1.111(0.89,1.388) | 0.352 |
| genus.Turicibacter.id.2162 | CD | Weighted median | 0.987(0.745,1.308) | 0.929 |
| genus.Turicibacter.id.2162 | CD | Weighted mode | 0.895(0.583,1.373) | 0.611 |
| genus.Turicibacter.id.2162 | CD | MR-Robust | 1.074(0.825,1.399) | 0.594 |
| genus.Turicibacter.id.2162 | CD | MR-Egger | 0.346(0.144,0.832) | 0.018 |
| genus.Turicibacter.id.2162 | CD | MRRAPS | 1.078(0.863,1.347) | 0.509 |
| genus.Turicibacter.id.2162 | CD | MRPRESSO | 1.111(0.89,1.388) | 0.376 |
| genus.Tyzzerella3.id.11335 | CD | IVW | 0.996(0.863,1.149) | 0.953 |
| genus.Tyzzerella3.id.11335 | CD | Weighted median | 1(0.823,1.216) | 0.998 |
| genus.Tyzzerella3.id.11335 | CD | Weighted mode | 1.016(0.718,1.437) | 0.930 |
| genus.Tyzzerella3.id.11335 | CD | MR-Robust | 0.992(0.857,1.148) | 0.915 |
| genus.Tyzzerella3.id.11335 | CD | MR-Egger | 2.379(1.112,5.092) | 0.026 |
| genus.Tyzzerella3.id.11335 | CD | MRRAPS | 0.991(0.844,1.164) | 0.913 |
| genus.Tyzzerella3.id.11335 | CD | MRPRESSO | 0.996(0.863,1.149) | 0.954 |
| genus.unknowngenus.id.1000000073 | CD | IVW | 0.951(0.771,1.172) | 0.636 |
| genus.unknowngenus.id.1000000073 | CD | Weighted median | 0.904(0.693,1.18) | 0.459 |
| genus.unknowngenus.id.1000000073 | CD | Weighted mode | 0.899(0.541,1.494) | 0.682 |
| genus.unknowngenus.id.1000000073 | CD | MR-Robust | 0.949(0.764,1.179) | 0.637 |
| genus.unknowngenus.id.1000000073 | CD | MR-Egger | 1.213(0.664,2.218) | 0.530 |
| genus.unknowngenus.id.1000000073 | CD | MRRAPS | 0.963(0.762,1.216) | 0.750 |
| genus.unknowngenus.id.1000000073 | CD | MRPRESSO | 0.951(0.771,1.172) | 0.643 |
| genus.unknowngenus.id.1000001215 | CD | IVW | 0.947(0.801,1.12) | 0.522 |
| genus.unknowngenus.id.1000001215 | CD | Weighted median | 0.935(0.747,1.17) | 0.559 |
| genus.unknowngenus.id.1000001215 | CD | Weighted mode | 0.938(0.676,1.301) | 0.703 |
| genus.unknowngenus.id.1000001215 | CD | MR-Robust | 0.946(0.804,1.114) | 0.508 |
| genus.unknowngenus.id.1000001215 | CD | MR-Egger | 0.778(0.482,1.254) | 0.303 |
| genus.unknowngenus.id.1000001215 | CD | MRRAPS | 0.944(0.787,1.132) | 0.532 |
| genus.unknowngenus.id.1000001215 | CD | MRPRESSO | 0.947(0.818,1.096) | 0.484 |
| genus.unknowngenus.id.1000005472 | CD | IVW | 1.054(0.874,1.27) | 0.584 |
| genus.unknowngenus.id.1000005472 | CD | Weighted median | 1.001(0.773,1.296) | 0.993 |
| genus.unknowngenus.id.1000005472 | CD | Weighted mode | 0.896(0.615,1.307) | 0.570 |
| genus.unknowngenus.id.1000005472 | CD | MR-Robust | 1.03(0.844,1.256) | 0.773 |
| genus.unknowngenus.id.1000005472 | CD | MR-Egger | 0.939(0.537,1.643) | 0.826 |
| genus.unknowngenus.id.1000005472 | CD | MRRAPS | 1.03(0.845,1.256) | 0.770 |
| genus.unknowngenus.id.1000005472 | CD | MRPRESSO | 1.054(0.874,1.27) | 0.593 |
| genus.unknowngenus.id.1000005479 | CD | IVW | 1.096(0.869,1.384) | 0.438 |
| genus.unknowngenus.id.1000005479 | CD | Weighted median | 1.074(0.807,1.427) | 0.625 |
| genus.unknowngenus.id.1000005479 | CD | Weighted mode | 1.028(0.64,1.653) | 0.909 |
| genus.unknowngenus.id.1000005479 | CD | MR-Robust | 1.1(0.87,1.391) | 0.425 |
| genus.unknowngenus.id.1000005479 | CD | MR-Egger | 0.812(0.281,2.352) | 0.702 |
| genus.unknowngenus.id.1000005479 | CD | MRRAPS | 1.114(0.878,1.415) | 0.374 |
| genus.unknowngenus.id.1000005479 | CD | MRPRESSO | 1.096(0.869,1.384) | 0.464 |
| genus.unknowngenus.id.1000006162 | CD | IVW | 1.152(1.007,1.318) | 0.039 |
| genus.unknowngenus.id.1000006162 | CD | Weighted median | 1.162(0.965,1.4) | 0.113 |
| genus.unknowngenus.id.1000006162 | CD | Weighted mode | 0.905(0.634,1.293) | 0.585 |
| genus.unknowngenus.id.1000006162 | CD | MR-Robust | 1.149(0.997,1.325) | 0.055 |
| genus.unknowngenus.id.1000006162 | CD | MR-Egger | 1.341(0.763,2.358) | 0.308 |
| genus.unknowngenus.id.1000006162 | CD | MRRAPS | 1.151(0.99,1.338) | 0.068 |
| genus.unknowngenus.id.1000006162 | CD | MRPRESSO | 1.152(1.007,1.318) | 0.059 |
| genus.unknowngenus.id.1868 | CD | IVW | 1.051(0.829,1.331) | 0.683 |
| genus.unknowngenus.id.1868 | CD | Weighted median | 1.271(0.957,1.688) | 0.098 |
| genus.unknowngenus.id.1868 | CD | Weighted mode | 1.423(0.905,2.239) | 0.127 |
| genus.unknowngenus.id.1868 | CD | MR-Robust | 1.067(0.739,1.541) | 0.729 |
| genus.unknowngenus.id.1868 | CD | MR-Egger | 0.907(0.408,2.015) | 0.811 |
| genus.unknowngenus.id.1868 | CD | MRRAPS | 1.049(0.8,1.376) | 0.730 |
| genus.unknowngenus.id.1868 | CD | MRPRESSO | 1.051(0.829,1.331) | 0.691 |
| genus.unknowngenus.id.2001 | CD | IVW | 1.082(0.862,1.36) | 0.496 |
| genus.unknowngenus.id.2001 | CD | Weighted median | 1.08(0.807,1.446) | 0.604 |
| genus.unknowngenus.id.2001 | CD | Weighted mode | 1.114(0.714,1.738) | 0.634 |
| genus.unknowngenus.id.2001 | CD | MR-Robust | 1.078(0.883,1.316) | 0.462 |
| genus.unknowngenus.id.2001 | CD | MR-Egger | 1.133(0.515,2.492) | 0.756 |
| genus.unknowngenus.id.2001 | CD | MRRAPS | 1.081(0.843,1.387) | 0.540 |
| genus.unknowngenus.id.2001 | CD | MRPRESSO | 1.082(0.908,1.29) | 0.402 |
| genus.unknowngenus.id.2041 | CD | IVW | 0.852(0.716,1.014) | 0.071 |
| genus.unknowngenus.id.2041 | CD | Weighted median | 0.806(0.639,1.017) | 0.070 |
| genus.unknowngenus.id.2041 | CD | Weighted mode | 0.798(0.562,1.134) | 0.208 |
| genus.unknowngenus.id.2041 | CD | MR-Robust | 0.811(0.638,1.03) | 0.085 |
| genus.unknowngenus.id.2041 | CD | MR-Egger | 0.728(0.431,1.229) | 0.235 |
| genus.unknowngenus.id.2041 | CD | MRRAPS | 0.833(0.689,1.007) | 0.059 |
| genus.unknowngenus.id.2041 | CD | MRPRESSO | 0.852(0.73,0.995) | 0.067 |
| genus.unknowngenus.id.2071 | CD | IVW | 1.19(0.964,1.471) | 0.106 |
| genus.unknowngenus.id.2071 | CD | Weighted median | 1.082(0.834,1.405) | 0.554 |
| genus.unknowngenus.id.2071 | CD | Weighted mode | 0.991(0.66,1.488) | 0.966 |
| genus.unknowngenus.id.2071 | CD | MR-Robust | 1.104(0.93,1.31) | 0.259 |
| genus.unknowngenus.id.2071 | CD | MR-Egger | 1.031(0.369,2.882) | 0.954 |
| genus.unknowngenus.id.2071 | CD | MRRAPS | 1.136(0.924,1.397) | 0.225 |
| genus.unknowngenus.id.2071 | CD | MRPRESSO | 1.19(0.964,1.471) | 0.126 |
| genus.unknowngenus.id.2755 | CD | IVW | 1.076(0.898,1.288) | 0.428 |
| genus.unknowngenus.id.2755 | CD | Weighted median | 1.108(0.872,1.408) | 0.401 |
| genus.unknowngenus.id.2755 | CD | Weighted mode | 1.131(0.764,1.672) | 0.539 |
| genus.unknowngenus.id.2755 | CD | MR-Robust | 1.071(0.916,1.251) | 0.390 |
| genus.unknowngenus.id.2755 | CD | MR-Egger | 0.832(0.373,1.856) | 0.654 |
| genus.unknowngenus.id.2755 | CD | MRRAPS | 1.075(0.884,1.308) | 0.469 |
| genus.unknowngenus.id.2755 | CD | MRPRESSO | 1.076(0.936,1.235) | 0.323 |
| genus.unknowngenus.id.826 | CD | IVW | 1.132(0.91,1.408) | 0.267 |
| genus.unknowngenus.id.826 | CD | Weighted median | 1.055(0.788,1.411) | 0.720 |
| genus.unknowngenus.id.826 | CD | Weighted mode | 0.956(0.613,1.491) | 0.842 |
| genus.unknowngenus.id.826 | CD | MR-Robust | 1.132(0.901,1.423) | 0.285 |
| genus.unknowngenus.id.826 | CD | MR-Egger | 1.237(0.675,2.266) | 0.492 |
| genus.unknowngenus.id.826 | CD | MRRAPS | 1.162(0.903,1.494) | 0.243 |
| genus.unknowngenus.id.826 | CD | MRPRESSO | 1.132(0.91,1.408) | 0.286 |
| genus.unknowngenus.id.959 | CD | IVW | 1.043(0.881,1.235) | 0.626 |
| genus.unknowngenus.id.959 | CD | Weighted median | 1.047(0.853,1.284) | 0.662 |
| genus.unknowngenus.id.959 | CD | Weighted mode | 1.018(0.705,1.47) | 0.923 |
| genus.unknowngenus.id.959 | CD | MR-Robust | 1.037(0.867,1.239) | 0.692 |
| genus.unknowngenus.id.959 | CD | MR-Egger | 0.815(0.288,2.309) | 0.701 |
| genus.unknowngenus.id.959 | CD | MRRAPS | 1.032(0.853,1.247) | 0.749 |
| genus.unknowngenus.id.959 | CD | MRPRESSO | 1.043(0.881,1.235) | 0.635 |
| genus.Veillonella.id.2198 | CD | IVW | 1.011(0.776,1.316) | 0.937 |
| genus.Veillonella.id.2198 | CD | Weighted median | 0.945(0.688,1.297) | 0.725 |
| genus.Veillonella.id.2198 | CD | Weighted mode | 0.911(0.559,1.485) | 0.710 |
| genus.Veillonella.id.2198 | CD | MR-Robust | 0.987(0.728,1.339) | 0.934 |
| genus.Veillonella.id.2198 | CD | MR-Egger | 0.86(0.202,3.661) | 0.838 |
| genus.Veillonella.id.2198 | CD | MRRAPS | 0.99(0.756,1.295) | 0.939 |
| genus.Veillonella.id.2198 | CD | MRPRESSO | 1.011(0.776,1.316) | 0.940 |
| genus.Victivallis.id.2256 | CD | IVW | 0.99(0.876,1.118) | 0.867 |
| genus.Victivallis.id.2256 | CD | Weighted median | 0.962(0.823,1.124) | 0.626 |
| genus.Victivallis.id.2256 | CD | Weighted mode | 0.937(0.739,1.187) | 0.588 |
| genus.Victivallis.id.2256 | CD | MR-Robust | 0.979(0.822,1.165) | 0.811 |
| genus.Victivallis.id.2256 | CD | MR-Egger | 1.04(0.465,2.327) | 0.924 |
| genus.Victivallis.id.2256 | CD | MRRAPS | 0.989(0.865,1.132) | 0.877 |
| genus.Victivallis.id.2256 | CD | MRPRESSO | 0.99(0.931,1.052) | 0.744 |
| order.Actinomycetales.id.420 | CD | IVW | 0.952(0.729,1.243) | 0.718 |
| order.Actinomycetales.id.420 | CD | Weighted median | 0.91(0.644,1.286) | 0.592 |
| order.Actinomycetales.id.420 | CD | Weighted mode | 0.895(0.57,1.404) | 0.628 |
| order.Actinomycetales.id.420 | CD | MR-Robust | 0.95(0.769,1.173) | 0.635 |
| order.Actinomycetales.id.420 | CD | MR-Egger | 0.694(0.33,1.459) | 0.335 |
| order.Actinomycetales.id.420 | CD | MRRAPS | 0.951(0.711,1.271) | 0.733 |
| order.Actinomycetales.id.420 | CD | MRPRESSO | 0.952(0.747,1.214) | 0.712 |
| order.Bacillales.id.1674 | CD | IVW | 0.995(0.87,1.137) | 0.937 |
| order.Bacillales.id.1674 | CD | Weighted median | 1.046(0.875,1.249) | 0.624 |
| order.Bacillales.id.1674 | CD | Weighted mode | 1.042(0.795,1.366) | 0.765 |
| order.Bacillales.id.1674 | CD | MR-Robust | 1.016(0.876,1.177) | 0.835 |
| order.Bacillales.id.1674 | CD | MR-Egger | 0.884(0.472,1.655) | 0.701 |
| order.Bacillales.id.1674 | CD | MRRAPS | 1.006(0.87,1.164) | 0.933 |
| order.Bacillales.id.1674 | CD | MRPRESSO | 0.995(0.893,1.108) | 0.924 |
| order.Bacteroidales.id.913 | CD | IVW | 1.058(0.834,1.341) | 0.643 |
| order.Bacteroidales.id.913 | CD | Weighted median | 1.097(0.789,1.524) | 0.582 |
| order.Bacteroidales.id.913 | CD | Weighted mode | 1.11(0.718,1.718) | 0.638 |
| order.Bacteroidales.id.913 | CD | MR-Robust | 1.018(0.814,1.274) | 0.873 |
| order.Bacteroidales.id.913 | CD | MR-Egger | 1.257(0.747,2.114) | 0.389 |
| order.Bacteroidales.id.913 | CD | MRRAPS | 1.02(0.789,1.318) | 0.880 |
| order.Bacteroidales.id.913 | CD | MRPRESSO | 1.058(0.84,1.331) | 0.641 |
| order.Bifidobacteriales.id.432 | CD | IVW | 0.882(0.733,1.061) | 0.184 |
| order.Bifidobacteriales.id.432 | CD | Weighted median | 0.902(0.716,1.138) | 0.385 |
| order.Bifidobacteriales.id.432 | CD | Weighted mode | 0.944(0.669,1.33) | 0.741 |
| order.Bifidobacteriales.id.432 | CD | MR-Robust | 0.868(0.741,1.016) | 0.079 |
| order.Bifidobacteriales.id.432 | CD | MR-Egger | 1.204(0.59,2.46) | 0.610 |
| order.Bifidobacteriales.id.432 | CD | MRRAPS | 0.866(0.714,1.05) | 0.142 |
| order.Bifidobacteriales.id.432 | CD | MRPRESSO | 0.882(0.733,1.061) | 0.196 |
| order.Burkholderiales.id.2874 | CD | IVW | 0.899(0.688,1.176) | 0.437 |
| order.Burkholderiales.id.2874 | CD | Weighted median | 1.024(0.702,1.494) | 0.902 |
| order.Burkholderiales.id.2874 | CD | Weighted mode | 1.097(0.588,2.049) | 0.771 |
| order.Burkholderiales.id.2874 | CD | MR-Robust | 0.923(0.654,1.303) | 0.651 |
| order.Burkholderiales.id.2874 | CD | MR-Egger | 0.449(0.179,1.126) | 0.088 |
| order.Burkholderiales.id.2874 | CD | MRRAPS | 0.917(0.687,1.223) | 0.555 |
| order.Burkholderiales.id.2874 | CD | MRPRESSO | 0.899(0.69,1.173) | 0.451 |
| order.Clostridiales.id.1863 | CD | IVW | 1.017(0.78,1.327) | 0.899 |
| order.Clostridiales.id.1863 | CD | Weighted median | 0.972(0.675,1.401) | 0.879 |
| order.Clostridiales.id.1863 | CD | Weighted mode | 0.97(0.548,1.716) | 0.916 |
| order.Clostridiales.id.1863 | CD | MR-Robust | 0.991(0.774,1.271) | 0.946 |
| order.Clostridiales.id.1863 | CD | MR-Egger | 0.894(0.241,3.313) | 0.867 |
| order.Clostridiales.id.1863 | CD | MRRAPS | 0.988(0.741,1.317) | 0.934 |
| order.Clostridiales.id.1863 | CD | MRPRESSO | 1.017(0.792,1.306) | 0.895 |
| order.Coriobacteriales.id.810 | CD | IVW | 0.961(0.765,1.208) | 0.736 |
| order.Coriobacteriales.id.810 | CD | Weighted median | 1.04(0.76,1.423) | 0.808 |
| order.Coriobacteriales.id.810 | CD | Weighted mode | 1.146(0.636,2.065) | 0.649 |
| order.Coriobacteriales.id.810 | CD | MR-Robust | 0.965(0.777,1.198) | 0.745 |
| order.Coriobacteriales.id.810 | CD | MR-Egger | 0.827(0.328,2.086) | 0.688 |
| order.Coriobacteriales.id.810 | CD | MRRAPS | 0.968(0.755,1.24) | 0.795 |
| order.Coriobacteriales.id.810 | CD | MRPRESSO | 0.961(0.78,1.186) | 0.718 |
| order.Desulfovibrionales.id.3156 | CD | IVW | 1.047(0.798,1.374) | 0.739 |
| order.Desulfovibrionales.id.3156 | CD | Weighted median | 1.076(0.783,1.479) | 0.653 |
| order.Desulfovibrionales.id.3156 | CD | Weighted mode | 1.128(0.715,1.779) | 0.606 |
| order.Desulfovibrionales.id.3156 | CD | MR-Robust | 1.03(0.802,1.322) | 0.818 |
| order.Desulfovibrionales.id.3156 | CD | MR-Egger | 1.234(0.583,2.616) | 0.582 |
| order.Desulfovibrionales.id.3156 | CD | MRRAPS | 1.03(0.798,1.331) | 0.818 |
| order.Desulfovibrionales.id.3156 | CD | MRPRESSO | 1.047(0.798,1.374) | 0.745 |
| order.Enterobacteriales.id.3468 | CD | IVW | 1.085(0.749,1.57) | 0.667 |
| order.Enterobacteriales.id.3468 | CD | Weighted median | 1.014(0.673,1.529) | 0.947 |
| order.Enterobacteriales.id.3468 | CD | Weighted mode | 0.836(0.403,1.735) | 0.631 |
| order.Enterobacteriales.id.3468 | CD | MR-Robust | 1.08(0.74,1.576) | 0.690 |
| order.Enterobacteriales.id.3468 | CD | MR-Egger | 0.805(0.063,10.339) | 0.868 |
| order.Enterobacteriales.id.3468 | CD | MRRAPS | 1.083(0.715,1.642) | 0.706 |
| order.Enterobacteriales.id.3468 | CD | MRPRESSO | 1.085(0.749,1.57) | 0.680 |
| order.Erysipelotrichales.id.2148 | CD | IVW | 1.044(0.802,1.36) | 0.747 |
| order.Erysipelotrichales.id.2148 | CD | Weighted median | 1.169(0.816,1.673) | 0.395 |
| order.Erysipelotrichales.id.2148 | CD | Weighted mode | 1.196(0.675,2.12) | 0.540 |
| order.Erysipelotrichales.id.2148 | CD | MR-Robust | 1.135(0.699,1.844) | 0.609 |
| order.Erysipelotrichales.id.2148 | CD | MR-Egger | 2.271(0.701,7.351) | 0.171 |
| order.Erysipelotrichales.id.2148 | CD | MRRAPS | 1.066(0.802,1.416) | 0.660 |
| order.Erysipelotrichales.id.2148 | CD | MRPRESSO | 1.044(0.803,1.359) | 0.752 |
| order.Gastranaerophilales.id.1591 | CD | IVW | 0.947(0.801,1.12) | 0.522 |
| order.Gastranaerophilales.id.1591 | CD | Weighted median | 0.935(0.747,1.17) | 0.559 |
| order.Gastranaerophilales.id.1591 | CD | Weighted mode | 0.938(0.676,1.301) | 0.703 |
| order.Gastranaerophilales.id.1591 | CD | MR-Robust | 0.946(0.804,1.114) | 0.508 |
| order.Gastranaerophilales.id.1591 | CD | MR-Egger | 0.778(0.482,1.254) | 0.303 |
| order.Gastranaerophilales.id.1591 | CD | MRRAPS | 0.944(0.787,1.132) | 0.532 |
| order.Gastranaerophilales.id.1591 | CD | MRPRESSO | 0.947(0.818,1.096) | 0.484 |
| order.Lactobacillales.id.1800 | CD | IVW | 1.012(0.796,1.286) | 0.923 |
| order.Lactobacillales.id.1800 | CD | Weighted median | 1.049(0.755,1.458) | 0.774 |
| order.Lactobacillales.id.1800 | CD | Weighted mode | 1.142(0.704,1.854) | 0.590 |
| order.Lactobacillales.id.1800 | CD | MR-Robust | 1.014(0.804,1.279) | 0.907 |
| order.Lactobacillales.id.1800 | CD | MR-Egger | 0.828(0.443,1.551) | 0.556 |
| order.Lactobacillales.id.1800 | CD | MRRAPS | 1.015(0.791,1.304) | 0.904 |
| order.Lactobacillales.id.1800 | CD | MRPRESSO | 1.012(0.796,1.286) | 0.924 |
| order.Methanobacteriales.id.120 | CD | IVW | 0.917(0.763,1.103) | 0.357 |
| order.Methanobacteriales.id.120 | CD | Weighted median | 0.96(0.792,1.164) | 0.677 |
| order.Methanobacteriales.id.120 | CD | Weighted mode | 1.019(0.737,1.409) | 0.908 |
| order.Methanobacteriales.id.120 | CD | MR-Robust | 0.919(0.767,1.102) | 0.363 |
| order.Methanobacteriales.id.120 | CD | MR-Egger | 1.255(0.574,2.742) | 0.569 |
| order.Methanobacteriales.id.120 | CD | MRRAPS | 0.925(0.77,1.109) | 0.399 |
| order.Methanobacteriales.id.120 | CD | MRPRESSO | 0.917(0.763,1.103) | 0.378 |
| order.MollicutesRF9.id.11579 | CD | IVW | 1.054(0.874,1.27) | 0.584 |
| order.MollicutesRF9.id.11579 | CD | Weighted median | 1.001(0.773,1.296) | 0.993 |
| order.MollicutesRF9.id.11579 | CD | Weighted mode | 0.896(0.615,1.307) | 0.570 |
| order.MollicutesRF9.id.11579 | CD | MR-Robust | 1.03(0.844,1.256) | 0.773 |
| order.MollicutesRF9.id.11579 | CD | MR-Egger | 0.939(0.537,1.643) | 0.826 |
| order.MollicutesRF9.id.11579 | CD | MRRAPS | 1.03(0.845,1.256) | 0.770 |
| order.MollicutesRF9.id.11579 | CD | MRPRESSO | 1.054(0.874,1.27) | 0.593 |
| order.NB1n.id.3953 | CD | IVW | 1.152(1.007,1.318) | 0.039 |
| order.NB1n.id.3953 | CD | Weighted median | 1.162(0.965,1.4) | 0.113 |
| order.NB1n.id.3953 | CD | Weighted mode | 0.905(0.634,1.293) | 0.585 |
| order.NB1n.id.3953 | CD | MR-Robust | 1.149(0.997,1.325) | 0.055 |
| order.NB1n.id.3953 | CD | MR-Egger | 1.341(0.763,2.358) | 0.308 |
| order.NB1n.id.3953 | CD | MRRAPS | 1.151(0.99,1.338) | 0.068 |
| order.NB1n.id.3953 | CD | MRPRESSO | 1.152(1.007,1.318) | 0.059 |
| order.Pasteurellales.id.3688 | CD | IVW | 0.979(0.827,1.158) | 0.804 |
| order.Pasteurellales.id.3688 | CD | Weighted median | 1.111(0.883,1.399) | 0.369 |
| order.Pasteurellales.id.3688 | CD | Weighted mode | 1.213(0.818,1.798) | 0.336 |
| order.Pasteurellales.id.3688 | CD | MR-Robust | 0.979(0.815,1.176) | 0.822 |
| order.Pasteurellales.id.3688 | CD | MR-Egger | 1.098(0.742,1.624) | 0.640 |
| order.Pasteurellales.id.3688 | CD | MRRAPS | 0.973(0.793,1.193) | 0.791 |
| order.Pasteurellales.id.3688 | CD | MRPRESSO | 0.979(0.827,1.158) | 0.807 |
| order.Rhodospirillales.id.2667 | CD | IVW | 1.046(0.872,1.254) | 0.630 |
| order.Rhodospirillales.id.2667 | CD | Weighted median | 0.984(0.77,1.257) | 0.897 |
| order.Rhodospirillales.id.2667 | CD | Weighted mode | 0.919(0.606,1.392) | 0.688 |
| order.Rhodospirillales.id.2667 | CD | MR-Robust | 1.041(0.878,1.233) | 0.645 |
| order.Rhodospirillales.id.2667 | CD | MR-Egger | 0.671(0.24,1.877) | 0.447 |
| order.Rhodospirillales.id.2667 | CD | MRRAPS | 1.044(0.857,1.271) | 0.671 |
| order.Rhodospirillales.id.2667 | CD | MRPRESSO | 1.046(0.895,1.221) | 0.583 |
| order.Selenomonadales.id.2165 | CD | IVW | 0.899(0.678,1.191) | 0.458 |
| order.Selenomonadales.id.2165 | CD | Weighted median | 0.987(0.676,1.443) | 0.947 |
| order.Selenomonadales.id.2165 | CD | Weighted mode | 1.162(0.595,2.269) | 0.660 |
| order.Selenomonadales.id.2165 | CD | MR-Robust | 0.913(0.656,1.271) | 0.589 |
| order.Selenomonadales.id.2165 | CD | MR-Egger | 0.875(0.334,2.296) | 0.787 |
| order.Selenomonadales.id.2165 | CD | MRRAPS | 0.908(0.667,1.235) | 0.537 |
| order.Selenomonadales.id.2165 | CD | MRPRESSO | 0.899(0.678,1.191) | 0.474 |
| order.Verrucomicrobiales.id.4030 | CD | IVW | 0.915(0.721,1.161) | 0.464 |
| order.Verrucomicrobiales.id.4030 | CD | Weighted median | 0.923(0.689,1.237) | 0.591 |
| order.Verrucomicrobiales.id.4030 | CD | Weighted mode | 0.908(0.581,1.421) | 0.674 |
| order.Verrucomicrobiales.id.4030 | CD | MR-Robust | 0.895(0.691,1.159) | 0.399 |
| order.Verrucomicrobiales.id.4030 | CD | MR-Egger | 0.803(0.314,2.053) | 0.647 |
| order.Verrucomicrobiales.id.4030 | CD | MRRAPS | 0.904(0.719,1.135) | 0.384 |
| order.Verrucomicrobiales.id.4030 | CD | MRPRESSO | 0.915(0.721,1.161) | 0.479 |
| order.Victivallales.id.2254 | CD | IVW | 0.994(0.84,1.176) | 0.942 |
| order.Victivallales.id.2254 | CD | Weighted median | 0.99(0.799,1.228) | 0.930 |
| order.Victivallales.id.2254 | CD | Weighted mode | 0.949(0.686,1.314) | 0.754 |
| order.Victivallales.id.2254 | CD | MR-Robust | 0.985(0.841,1.152) | 0.848 |
| order.Victivallales.id.2254 | CD | MR-Egger | 1.065(0.59,1.92) | 0.835 |
| order.Victivallales.id.2254 | CD | MRRAPS | 0.987(0.822,1.185) | 0.885 |
| order.Victivallales.id.2254 | CD | MRPRESSO | 0.994(0.861,1.146) | 0.934 |
| phylum.Actinobacteria.id.400 | CD | IVW | 0.909(0.701,1.179) | 0.474 |
| phylum.Actinobacteria.id.400 | CD | Weighted median | 0.862(0.632,1.175) | 0.348 |
| phylum.Actinobacteria.id.400 | CD | Weighted mode | 0.855(0.518,1.41) | 0.538 |
| phylum.Actinobacteria.id.400 | CD | MR-Robust | 0.912(0.707,1.176) | 0.477 |
| phylum.Actinobacteria.id.400 | CD | MR-Egger | 0.742(0.262,2.105) | 0.575 |
| phylum.Actinobacteria.id.400 | CD | MRRAPS | 0.907(0.692,1.189) | 0.481 |
| phylum.Actinobacteria.id.400 | CD | MRPRESSO | 0.909(0.701,1.179) | 0.483 |
| phylum.Bacteroidetes.id.905 | CD | IVW | 1.033(0.81,1.317) | 0.792 |
| phylum.Bacteroidetes.id.905 | CD | Weighted median | 1.075(0.765,1.512) | 0.676 |
| phylum.Bacteroidetes.id.905 | CD | Weighted mode | 1.091(0.706,1.686) | 0.695 |
| phylum.Bacteroidetes.id.905 | CD | MR-Robust | 0.981(0.734,1.31) | 0.894 |
| phylum.Bacteroidetes.id.905 | CD | MR-Egger | 1.538(0.915,2.587) | 0.104 |
| phylum.Bacteroidetes.id.905 | CD | MRRAPS | 0.992(0.764,1.289) | 0.953 |
| phylum.Bacteroidetes.id.905 | CD | MRPRESSO | 1.033(0.811,1.316) | 0.797 |
| phylum.Cyanobacteria.id.1500 | CD | IVW | 0.838(0.687,1.023) | 0.083 |
| phylum.Cyanobacteria.id.1500 | CD | Weighted median | 0.838(0.649,1.082) | 0.175 |
| phylum.Cyanobacteria.id.1500 | CD | Weighted mode | 0.834(0.562,1.238) | 0.369 |
| phylum.Cyanobacteria.id.1500 | CD | MR-Robust | 0.835(0.648,1.075) | 0.161 |
| phylum.Cyanobacteria.id.1500 | CD | MR-Egger | 0.729(0.369,1.439) | 0.362 |
| phylum.Cyanobacteria.id.1500 | CD | MRRAPS | 0.836(0.672,1.041) | 0.109 |
| phylum.Cyanobacteria.id.1500 | CD | MRPRESSO | 0.838(0.742,0.947) | 0.025 |
| phylum.Euryarchaeota.id.55 | CD | IVW | 0.998(0.859,1.16) | 0.979 |
| phylum.Euryarchaeota.id.55 | CD | Weighted median | 1.084(0.911,1.289) | 0.365 |
| phylum.Euryarchaeota.id.55 | CD | Weighted mode | 1.122(0.866,1.453) | 0.385 |
| phylum.Euryarchaeota.id.55 | CD | MR-Robust | 1.052(0.848,1.305) | 0.646 |
| phylum.Euryarchaeota.id.55 | CD | MR-Egger | 0.95(0.466,1.936) | 0.888 |
| phylum.Euryarchaeota.id.55 | CD | MRRAPS | 1.025(0.884,1.189) | 0.742 |
| phylum.Euryarchaeota.id.55 | CD | MRPRESSO | 0.998(0.859,1.16) | 0.979 |
| phylum.Firmicutes.id.1672 | CD | IVW | 0.876(0.654,1.172) | 0.372 |
| phylum.Firmicutes.id.1672 | CD | Weighted median | 0.901(0.648,1.252) | 0.534 |
| phylum.Firmicutes.id.1672 | CD | Weighted mode | 0.885(0.543,1.443) | 0.624 |
| phylum.Firmicutes.id.1672 | CD | MR-Robust | 0.9(0.678,1.195) | 0.465 |
| phylum.Firmicutes.id.1672 | CD | MR-Egger | 0.469(0.224,0.981) | 0.044 |
| phylum.Firmicutes.id.1672 | CD | MRRAPS | 0.872(0.667,1.141) | 0.318 |
| phylum.Firmicutes.id.1672 | CD | MRPRESSO | 0.876(0.654,1.172) | 0.387 |
| phylum.Lentisphaerae.id.2238 | CD | IVW | 0.965(0.822,1.131) | 0.658 |
| phylum.Lentisphaerae.id.2238 | CD | Weighted median | 0.973(0.79,1.2) | 0.800 |
| phylum.Lentisphaerae.id.2238 | CD | Weighted mode | 0.92(0.671,1.262) | 0.607 |
| phylum.Lentisphaerae.id.2238 | CD | MR-Robust | 0.955(0.826,1.104) | 0.535 |
| phylum.Lentisphaerae.id.2238 | CD | MR-Egger | 0.988(0.546,1.787) | 0.968 |
| phylum.Lentisphaerae.id.2238 | CD | MRRAPS | 0.955(0.803,1.135) | 0.600 |
| phylum.Lentisphaerae.id.2238 | CD | MRPRESSO | 0.965(0.84,1.108) | 0.624 |
| phylum.Proteobacteria.id.2375 | CD | IVW | 0.95(0.653,1.382) | 0.788 |
| phylum.Proteobacteria.id.2375 | CD | Weighted median | 0.977(0.665,1.435) | 0.906 |
| phylum.Proteobacteria.id.2375 | CD | Weighted mode | 1.047(0.557,1.968) | 0.887 |
| phylum.Proteobacteria.id.2375 | CD | MR-Robust | 0.947(0.662,1.354) | 0.765 |
| phylum.Proteobacteria.id.2375 | CD | MR-Egger | 0.763(0.215,2.708) | 0.675 |
| phylum.Proteobacteria.id.2375 | CD | MRRAPS | 0.946(0.649,1.378) | 0.771 |
| phylum.Proteobacteria.id.2375 | CD | MRPRESSO | 0.95(0.653,1.382) | 0.793 |
| phylum.Tenericutes.id.3919 | CD | IVW | 1.028(0.83,1.274) | 0.801 |
| phylum.Tenericutes.id.3919 | CD | Weighted median | 1.073(0.798,1.442) | 0.643 |
| phylum.Tenericutes.id.3919 | CD | Weighted mode | 1.224(0.781,1.92) | 0.378 |
| phylum.Tenericutes.id.3919 | CD | MR-Robust | 1.031(0.839,1.267) | 0.772 |
| phylum.Tenericutes.id.3919 | CD | MR-Egger | 1.28(0.635,2.581) | 0.490 |
| phylum.Tenericutes.id.3919 | CD | MRRAPS | 1.035(0.824,1.3) | 0.765 |
| phylum.Tenericutes.id.3919 | CD | MRPRESSO | 1.028(0.83,1.274) | 0.805 |
| phylum.Verrucomicrobia.id.3982 | CD | IVW | 0.946(0.735,1.218) | 0.667 |
| phylum.Verrucomicrobia.id.3982 | CD | Weighted median | 0.875(0.651,1.176) | 0.375 |
| phylum.Verrucomicrobia.id.3982 | CD | Weighted mode | 0.861(0.557,1.33) | 0.500 |
| phylum.Verrucomicrobia.id.3982 | CD | MR-Robust | 0.945(0.75,1.191) | 0.632 |
| phylum.Verrucomicrobia.id.3982 | CD | MR-Egger | 1.034(0.491,2.176) | 0.930 |
| phylum.Verrucomicrobia.id.3982 | CD | MRRAPS | 0.948(0.751,1.197) | 0.655 |
| phylum.Verrucomicrobia.id.3982 | CD | MRPRESSO | 0.946(0.735,1.218) | 0.676 |

Abbreviations: IVW, inverse-variance weighted; MR, Mendelian randomization; MR-RAPS, Robust Adjusted Profile Score; MR-PRESSO, Mendelian Randomization Pleiotropy RESidual Sum and Outlier; CD, Crohn’s disease.

**S10 Table. Effect estimates of the associations between 196 bacterial traits and risk of ulcerative colitis in MR analyses.**

| **Exposure** | **Outcome** | **Method** | **OR (95CI%)** | ***P*-value** |
| --- | --- | --- | --- | --- |
| class.Actinobacteria.id.419 | UC | IVW | 0.885(0.735,1.065) | 0.196 |
| class.Actinobacteria.id.419 | UC | Weighted median | 0.917(0.724,1.163) | 0.475 |
| class.Actinobacteria.id.419 | UC | Weighted mode | 0.949(0.68,1.326) | 0.761 |
| class.Actinobacteria.id.419 | UC | MR-Robust | 0.886(0.75,1.048) | 0.157 |
| class.Actinobacteria.id.419 | UC | MR-Egger | 0.702(0.38,1.295) | 0.258 |
| class.Actinobacteria.id.419 | UC | MRRAPS | 0.88(0.739,1.047) | 0.148 |
| class.Actinobacteria.id.419 | UC | MRPRESSO | 0.885(0.735,1.065) | 0.210 |
| class.Alphaproteobacteria.id.2379 | UC | IVW | 1.004(0.779,1.293) | 0.977 |
| class.Alphaproteobacteria.id.2379 | UC | Weighted median | 0.966(0.702,1.329) | 0.832 |
| class.Alphaproteobacteria.id.2379 | UC | Weighted mode | 0.918(0.566,1.491) | 0.731 |
| class.Alphaproteobacteria.id.2379 | UC | MR-Robust | 0.992(0.767,1.283) | 0.953 |
| class.Alphaproteobacteria.id.2379 | UC | MR-Egger | 1.746(0.692,4.402) | 0.238 |
| class.Alphaproteobacteria.id.2379 | UC | MRRAPS | 0.981(0.757,1.273) | 0.887 |
| class.Alphaproteobacteria.id.2379 | UC | MRPRESSO | 1.004(0.779,1.293) | 0.978 |
| class.Bacilli.id.1673 | UC | IVW | 0.978(0.774,1.235) | 0.849 |
| class.Bacilli.id.1673 | UC | Weighted median | 0.947(0.709,1.263) | 0.709 |
| class.Bacilli.id.1673 | UC | Weighted mode | 0.911(0.592,1.4) | 0.671 |
| class.Bacilli.id.1673 | UC | MR-Robust | 0.984(0.786,1.233) | 0.891 |
| class.Bacilli.id.1673 | UC | MR-Egger | 1.216(0.635,2.331) | 0.555 |
| class.Bacilli.id.1673 | UC | MRRAPS | 1.003(0.802,1.256) | 0.977 |
| class.Bacilli.id.1673 | UC | MRPRESSO | 0.978(0.774,1.235) | 0.851 |
| class.Bacteroidia.id.912 | UC | IVW | 0.932(0.74,1.174) | 0.549 |
| class.Bacteroidia.id.912 | UC | Weighted median | 0.985(0.726,1.337) | 0.924 |
| class.Bacteroidia.id.912 | UC | Weighted mode | 1.096(0.731,1.642) | 0.658 |
| class.Bacteroidia.id.912 | UC | MR-Robust | 0.95(0.746,1.209) | 0.674 |
| class.Bacteroidia.id.912 | UC | MR-Egger | 1.445(0.899,2.321) | 0.128 |
| class.Bacteroidia.id.912 | UC | MRRAPS | 0.947(0.748,1.199) | 0.651 |
| class.Bacteroidia.id.912 | UC | MRPRESSO | 0.932(0.74,1.174) | 0.561 |
| class.Betaproteobacteria.id.2867 | UC | IVW | 1.114(0.878,1.413) | 0.375 |
| class.Betaproteobacteria.id.2867 | UC | Weighted median | 1.085(0.799,1.475) | 0.601 |
| class.Betaproteobacteria.id.2867 | UC | Weighted mode | 1.053(0.642,1.728) | 0.838 |
| class.Betaproteobacteria.id.2867 | UC | MR-Robust | 1.079(0.824,1.414) | 0.579 |
| class.Betaproteobacteria.id.2867 | UC | MR-Egger | 1.444(0.612,3.405) | 0.402 |
| class.Betaproteobacteria.id.2867 | UC | MRRAPS | 1.102(0.85,1.429) | 0.463 |
| class.Betaproteobacteria.id.2867 | UC | MRPRESSO | 1.114(0.929,1.335) | 0.268 |
| class.Clostridia.id.1859 | UC | IVW | 1.267(0.972,1.652) | 0.081 |
| class.Clostridia.id.1859 | UC | Weighted median | 1.498(1.059,2.118) | 0.022 |
| class.Clostridia.id.1859 | UC | Weighted mode | 1.62(0.906,2.897) | 0.104 |
| class.Clostridia.id.1859 | UC | MR-Robust | 1.347(0.86,2.109) | 0.193 |
| class.Clostridia.id.1859 | UC | MR-Egger | 1.953(0.468,8.158) | 0.359 |
| class.Clostridia.id.1859 | UC | MRRAPS | 1.328(1.003,1.758) | 0.048 |
| class.Clostridia.id.1859 | UC | MRPRESSO | 1.267(0.972,1.652) | 0.108 |
| class.Coriobacteriia.id.809 | UC | IVW | 1.16(0.907,1.484) | 0.236 |
| class.Coriobacteriia.id.809 | UC | Weighted median | 1.142(0.842,1.549) | 0.392 |
| class.Coriobacteriia.id.809 | UC | Weighted mode | 1.115(0.647,1.922) | 0.696 |
| class.Coriobacteriia.id.809 | UC | MR-Robust | 1.177(0.881,1.574) | 0.270 |
| class.Coriobacteriia.id.809 | UC | MR-Egger | 1.331(0.471,3.761) | 0.589 |
| class.Coriobacteriia.id.809 | UC | MRRAPS | 1.182(0.906,1.54) | 0.218 |
| class.Coriobacteriia.id.809 | UC | MRPRESSO | 1.16(0.907,1.484) | 0.253 |
| class.Deltaproteobacteria.id.3087 | UC | IVW | 0.994(0.799,1.236) | 0.954 |
| class.Deltaproteobacteria.id.3087 | UC | Weighted median | 1.015(0.755,1.367) | 0.919 |
| class.Deltaproteobacteria.id.3087 | UC | Weighted mode | 1.041(0.686,1.58) | 0.849 |
| class.Deltaproteobacteria.id.3087 | UC | MR-Robust | 0.994(0.805,1.226) | 0.952 |
| class.Deltaproteobacteria.id.3087 | UC | MR-Egger | 1.124(0.598,2.115) | 0.716 |
| class.Deltaproteobacteria.id.3087 | UC | MRRAPS | 0.985(0.789,1.229) | 0.893 |
| class.Deltaproteobacteria.id.3087 | UC | MRPRESSO | 0.994(0.799,1.236) | 0.955 |
| class.Erysipelotrichia.id.2147 | UC | IVW | 0.948(0.745,1.206) | 0.664 |
| class.Erysipelotrichia.id.2147 | UC | Weighted median | 1.051(0.763,1.45) | 0.760 |
| class.Erysipelotrichia.id.2147 | UC | Weighted mode | 1.136(0.676,1.91) | 0.630 |
| class.Erysipelotrichia.id.2147 | UC | MR-Robust | 0.99(0.703,1.393) | 0.953 |
| class.Erysipelotrichia.id.2147 | UC | MR-Egger | 0.68(0.236,1.96) | 0.475 |
| class.Erysipelotrichia.id.2147 | UC | MRRAPS | 0.965(0.742,1.253) | 0.787 |
| class.Erysipelotrichia.id.2147 | UC | MRPRESSO | 0.948(0.776,1.158) | 0.610 |
| class.Gammaproteobacteria.id.3303 | UC | IVW | 0.901(0.674,1.203) | 0.478 |
| class.Gammaproteobacteria.id.3303 | UC | Weighted median | 0.893(0.614,1.298) | 0.553 |
| class.Gammaproteobacteria.id.3303 | UC | Weighted mode | 0.934(0.542,1.608) | 0.804 |
| class.Gammaproteobacteria.id.3303 | UC | MR-Robust | 0.904(0.718,1.14) | 0.394 |
| class.Gammaproteobacteria.id.3303 | UC | MR-Egger | 0.888(0.32,2.467) | 0.820 |
| class.Gammaproteobacteria.id.3303 | UC | MRRAPS | 0.899(0.655,1.234) | 0.509 |
| class.Gammaproteobacteria.id.3303 | UC | MRPRESSO | 0.901(0.733,1.107) | 0.358 |
| class.Lentisphaeria.id.2250 | UC | IVW | 0.825(0.706,0.965) | 0.016 |
| class.Lentisphaeria.id.2250 | UC | Weighted median | 0.859(0.696,1.06) | 0.156 |
| class.Lentisphaeria.id.2250 | UC | Weighted mode | 0.987(0.694,1.404) | 0.940 |
| class.Lentisphaeria.id.2250 | UC | MR-Robust | 0.827(0.709,0.964) | 0.015 |
| class.Lentisphaeria.id.2250 | UC | MR-Egger | 1.064(0.623,1.817) | 0.821 |
| class.Lentisphaeria.id.2250 | UC | MRRAPS | 0.82(0.69,0.975) | 0.024 |
| class.Lentisphaeria.id.2250 | UC | MRPRESSO | 0.825(0.711,0.959) | 0.040 |
| class.Melainabacteria.id.1589 | UC | IVW | 0.929(0.801,1.077) | 0.327 |
| class.Melainabacteria.id.1589 | UC | Weighted median | 0.924(0.76,1.123) | 0.424 |
| class.Melainabacteria.id.1589 | UC | Weighted mode | 0.907(0.656,1.254) | 0.554 |
| class.Melainabacteria.id.1589 | UC | MR-Robust | 0.929(0.818,1.056) | 0.259 |
| class.Melainabacteria.id.1589 | UC | MR-Egger | 1.027(0.675,1.563) | 0.901 |
| class.Melainabacteria.id.1589 | UC | MRRAPS | 0.928(0.79,1.09) | 0.361 |
| class.Melainabacteria.id.1589 | UC | MRPRESSO | 0.929(0.818,1.054) | 0.282 |
| class.Methanobacteria.id.119 | UC | IVW | 0.96(0.827,1.115) | 0.594 |
| class.Methanobacteria.id.119 | UC | Weighted median | 0.907(0.771,1.066) | 0.234 |
| class.Methanobacteria.id.119 | UC | Weighted mode | 0.9(0.702,1.156) | 0.410 |
| class.Methanobacteria.id.119 | UC | MR-Robust | 0.948(0.778,1.156) | 0.599 |
| class.Methanobacteria.id.119 | UC | MR-Egger | 1.027(0.535,1.972) | 0.937 |
| class.Methanobacteria.id.119 | UC | MRRAPS | 0.969(0.832,1.13) | 0.690 |
| class.Methanobacteria.id.119 | UC | MRPRESSO | 0.96(0.827,1.115) | 0.606 |
| class.Mollicutes.id.3920 | UC | IVW | 0.994(0.798,1.238) | 0.955 |
| class.Mollicutes.id.3920 | UC | Weighted median | 0.923(0.701,1.217) | 0.571 |
| class.Mollicutes.id.3920 | UC | Weighted mode | 0.921(0.58,1.464) | 0.728 |
| class.Mollicutes.id.3920 | UC | MR-Robust | 0.983(0.767,1.261) | 0.895 |
| class.Mollicutes.id.3920 | UC | MR-Egger | 0.709(0.348,1.443) | 0.343 |
| class.Mollicutes.id.3920 | UC | MRRAPS | 0.955(0.674,1.352) | 0.793 |
| class.Mollicutes.id.3920 | UC | MRPRESSO | 0.994(0.798,1.238) | 0.956 |
| class.Negativicutes.id.2164 | UC | IVW | 0.915(0.707,1.186) | 0.504 |
| class.Negativicutes.id.2164 | UC | Weighted median | 0.893(0.642,1.243) | 0.503 |
| class.Negativicutes.id.2164 | UC | Weighted mode | 0.88(0.552,1.403) | 0.591 |
| class.Negativicutes.id.2164 | UC | MR-Robust | 0.868(0.651,1.158) | 0.336 |
| class.Negativicutes.id.2164 | UC | MR-Egger | 0.701(0.296,1.658) | 0.418 |
| class.Negativicutes.id.2164 | UC | MRRAPS | 0.883(0.677,1.15) | 0.356 |
| class.Negativicutes.id.2164 | UC | MRPRESSO | 0.915(0.707,1.186) | 0.518 |
| class.Verrucomicrobiae.id.4029 | UC | IVW | 0.975(0.771,1.233) | 0.832 |
| class.Verrucomicrobiae.id.4029 | UC | Weighted median | 0.947(0.708,1.268) | 0.716 |
| class.Verrucomicrobiae.id.4029 | UC | Weighted mode | 0.7(0.385,1.272) | 0.242 |
| class.Verrucomicrobiae.id.4029 | UC | MR-Robust | 0.974(0.758,1.251) | 0.836 |
| class.Verrucomicrobiae.id.4029 | UC | MR-Egger | 0.455(0.209,0.989) | 0.047 |
| class.Verrucomicrobiae.id.4029 | UC | MRRAPS | 0.92(0.688,1.23) | 0.572 |
| class.Verrucomicrobiae.id.4029 | UC | MRPRESSO | 0.975(0.771,1.233) | 0.836 |
| family.Acidaminococcaceae.id.2166 | UC | IVW | 0.824(0.63,1.078) | 0.158 |
| family.Acidaminococcaceae.id.2166 | UC | Weighted median | 0.805(0.579,1.12) | 0.198 |
| family.Acidaminococcaceae.id.2166 | UC | Weighted mode | 0.785(0.483,1.276) | 0.329 |
| family.Acidaminococcaceae.id.2166 | UC | MR-Robust | 0.817(0.613,1.09) | 0.170 |
| family.Acidaminococcaceae.id.2166 | UC | MR-Egger | 0.846(0.379,1.89) | 0.683 |
| family.Acidaminococcaceae.id.2166 | UC | MRRAPS | 0.821(0.61,1.106) | 0.195 |
| family.Acidaminococcaceae.id.2166 | UC | MRPRESSO | 0.824(0.711,0.956) | 0.043 |
| family.Actinomycetaceae.id.421 | UC | IVW | 1.08(0.841,1.386) | 0.549 |
| family.Actinomycetaceae.id.421 | UC | Weighted median | 1.199(0.871,1.65) | 0.266 |
| family.Actinomycetaceae.id.421 | UC | Weighted mode | 1.22(0.804,1.851) | 0.349 |
| family.Actinomycetaceae.id.421 | UC | MR-Robust | 1.11(0.707,1.743) | 0.651 |
| family.Actinomycetaceae.id.421 | UC | MR-Egger | 1.338(0.659,2.716) | 0.420 |
| family.Actinomycetaceae.id.421 | UC | MRRAPS | 1.081(0.822,1.422) | 0.576 |
| family.Actinomycetaceae.id.421 | UC | MRPRESSO | 1.08(0.897,1.3) | 0.464 |
| family.Alcaligenaceae.id.2875 | UC | IVW | 0.983(0.792,1.22) | 0.876 |
| family.Alcaligenaceae.id.2875 | UC | Weighted median | 1.049(0.795,1.384) | 0.736 |
| family.Alcaligenaceae.id.2875 | UC | Weighted mode | 1.067(0.672,1.695) | 0.782 |
| family.Alcaligenaceae.id.2875 | UC | MR-Robust | 1.037(0.769,1.399) | 0.812 |
| family.Alcaligenaceae.id.2875 | UC | MR-Egger | 1.255(0.496,3.177) | 0.632 |
| family.Alcaligenaceae.id.2875 | UC | MRRAPS | 0.987(0.779,1.25) | 0.913 |
| family.Alcaligenaceae.id.2875 | UC | MRPRESSO | 0.983(0.866,1.116) | 0.795 |
| family.Bacteroidaceae.id.917 | UC | IVW | 0.895(0.66,1.216) | 0.479 |
| family.Bacteroidaceae.id.917 | UC | Weighted median | 0.882(0.59,1.321) | 0.543 |
| family.Bacteroidaceae.id.917 | UC | Weighted mode | 0.887(0.462,1.703) | 0.719 |
| family.Bacteroidaceae.id.917 | UC | MR-Robust | 0.89(0.622,1.273) | 0.523 |
| family.Bacteroidaceae.id.917 | UC | MR-Egger | 1.534(0.249,9.464) | 0.645 |
| family.Bacteroidaceae.id.917 | UC | MRRAPS | 0.884(0.636,1.229) | 0.464 |
| family.Bacteroidaceae.id.917 | UC | MRPRESSO | 0.895(0.665,1.206) | 0.491 |
| family.BacteroidalesS24.7group.id.11173 | UC | IVW | 0.953(0.789,1.152) | 0.622 |
| family.BacteroidalesS24.7group.id.11173 | UC | Weighted median | 0.93(0.723,1.195) | 0.569 |
| family.BacteroidalesS24.7group.id.11173 | UC | Weighted mode | 0.92(0.619,1.366) | 0.678 |
| family.BacteroidalesS24.7group.id.11173 | UC | MR-Robust | 0.944(0.789,1.129) | 0.527 |
| family.BacteroidalesS24.7group.id.11173 | UC | MR-Egger | 0.545(0.241,1.233) | 0.145 |
| family.BacteroidalesS24.7group.id.11173 | UC | MRRAPS | 0.936(0.762,1.148) | 0.524 |
| family.BacteroidalesS24.7group.id.11173 | UC | MRPRESSO | 0.953(0.795,1.143) | 0.622 |
| family.Bifidobacteriaceae.id.433 | UC | IVW | 0.763(0.631,0.922) | 0.005 |
| family.Bifidobacteriaceae.id.433 | UC | Weighted median | 0.912(0.733,1.133) | 0.405 |
| family.Bifidobacteriaceae.id.433 | UC | Weighted mode | 0.972(0.717,1.32) | 0.858 |
| family.Bifidobacteriaceae.id.433 | UC | MR-Robust | 0.808(0.661,0.988) | 0.038 |
| family.Bifidobacteriaceae.id.433 | UC | MR-Egger | 0.76(0.359,1.61) | 0.474 |
| family.Bifidobacteriaceae.id.433 | UC | MRRAPS | 0.782(0.646,0.947) | 0.012 |
| family.Bifidobacteriaceae.id.433 | UC | MRPRESSO | 0.763(0.631,0.922) | 0.010 |
| family.Christensenellaceae.id.1866 | UC | IVW | 0.878(0.697,1.107) | 0.272 |
| family.Christensenellaceae.id.1866 | UC | Weighted median | 0.786(0.578,1.068) | 0.124 |
| family.Christensenellaceae.id.1866 | UC | Weighted mode | 0.74(0.457,1.198) | 0.221 |
| family.Christensenellaceae.id.1866 | UC | MR-Robust | 0.878(0.699,1.103) | 0.264 |
| family.Christensenellaceae.id.1866 | UC | MR-Egger | 0.818(0.488,1.371) | 0.446 |
| family.Christensenellaceae.id.1866 | UC | MRRAPS | 0.879(0.689,1.121) | 0.298 |
| family.Christensenellaceae.id.1866 | UC | MRPRESSO | 0.878(0.697,1.107) | 0.298 |
| family.Clostridiaceae1.id.1869 | UC | IVW | 0.901(0.711,1.143) | 0.392 |
| family.Clostridiaceae1.id.1869 | UC | Weighted median | 0.877(0.638,1.206) | 0.421 |
| family.Clostridiaceae1.id.1869 | UC | Weighted mode | 0.842(0.556,1.276) | 0.418 |
| family.Clostridiaceae1.id.1869 | UC | MR-Robust | 0.943(0.803,1.107) | 0.472 |
| family.Clostridiaceae1.id.1869 | UC | MR-Egger | 0.846(0.419,1.712) | 0.643 |
| family.Clostridiaceae1.id.1869 | UC | MRRAPS | 0.926(0.715,1.2) | 0.560 |
| family.Clostridiaceae1.id.1869 | UC | MRPRESSO | 0.901(0.728,1.116) | 0.366 |
| family.ClostridialesvadinBB60group.id.11286 | UC | IVW | 0.919(0.782,1.08) | 0.303 |
| family.ClostridialesvadinBB60group.id.11286 | UC | Weighted median | 0.959(0.773,1.19) | 0.706 |
| family.ClostridialesvadinBB60group.id.11286 | UC | Weighted mode | 0.944(0.659,1.353) | 0.754 |
| family.ClostridialesvadinBB60group.id.11286 | UC | MR-Robust | 0.923(0.798,1.069) | 0.285 |
| family.ClostridialesvadinBB60group.id.11286 | UC | MR-Egger | 0.988(0.625,1.561) | 0.958 |
| family.ClostridialesvadinBB60group.id.11286 | UC | MRRAPS | 0.92(0.772,1.097) | 0.354 |
| family.ClostridialesvadinBB60group.id.11286 | UC | MRPRESSO | 0.919(0.811,1.042) | 0.207 |
| family.Coriobacteriaceae.id.811 | UC | IVW | 1.16(0.907,1.484) | 0.236 |
| family.Coriobacteriaceae.id.811 | UC | Weighted median | 1.142(0.842,1.549) | 0.392 |
| family.Coriobacteriaceae.id.811 | UC | Weighted mode | 1.115(0.647,1.922) | 0.696 |
| family.Coriobacteriaceae.id.811 | UC | MR-Robust | 1.177(0.881,1.574) | 0.270 |
| family.Coriobacteriaceae.id.811 | UC | MR-Egger | 1.331(0.471,3.761) | 0.589 |
| family.Coriobacteriaceae.id.811 | UC | MRRAPS | 1.182(0.906,1.54) | 0.218 |
| family.Coriobacteriaceae.id.811 | UC | MRPRESSO | 1.16(0.907,1.484) | 0.253 |
| family.Defluviitaleaceae.id.1924 | UC | IVW | 0.857(0.716,1.027) | 0.094 |
| family.Defluviitaleaceae.id.1924 | UC | Weighted median | 0.934(0.74,1.18) | 0.567 |
| family.Defluviitaleaceae.id.1924 | UC | Weighted mode | 0.97(0.67,1.403) | 0.871 |
| family.Defluviitaleaceae.id.1924 | UC | MR-Robust | 0.868(0.722,1.042) | 0.129 |
| family.Defluviitaleaceae.id.1924 | UC | MR-Egger | 0.991(0.524,1.873) | 0.977 |
| family.Defluviitaleaceae.id.1924 | UC | MRRAPS | 0.863(0.715,1.041) | 0.124 |
| family.Defluviitaleaceae.id.1924 | UC | MRPRESSO | 0.857(0.716,1.027) | 0.123 |
| family.Desulfovibrionaceae.id.3169 | UC | IVW | 1.079(0.86,1.355) | 0.511 |
| family.Desulfovibrionaceae.id.3169 | UC | Weighted median | 1.048(0.765,1.435) | 0.772 |
| family.Desulfovibrionaceae.id.3169 | UC | Weighted mode | 0.996(0.661,1.5) | 0.983 |
| family.Desulfovibrionaceae.id.3169 | UC | MR-Robust | 1.077(0.871,1.333) | 0.493 |
| family.Desulfovibrionaceae.id.3169 | UC | MR-Egger | 1.078(0.595,1.954) | 0.803 |
| family.Desulfovibrionaceae.id.3169 | UC | MRRAPS | 1.083(0.847,1.386) | 0.524 |
| family.Desulfovibrionaceae.id.3169 | UC | MRPRESSO | 1.079(0.863,1.349) | 0.521 |
| family.Enterobacteriaceae.id.3469 | UC | IVW | 0.899(0.677,1.193) | 0.461 |
| family.Enterobacteriaceae.id.3469 | UC | Weighted median | 0.941(0.659,1.344) | 0.739 |
| family.Enterobacteriaceae.id.3469 | UC | Weighted mode | 0.98(0.587,1.637) | 0.940 |
| family.Enterobacteriaceae.id.3469 | UC | MR-Robust | 0.969(0.718,1.309) | 0.839 |
| family.Enterobacteriaceae.id.3469 | UC | MR-Egger | 2.466(0.415,14.666) | 0.321 |
| family.Enterobacteriaceae.id.3469 | UC | MRRAPS | 0.945(0.707,1.265) | 0.706 |
| family.Enterobacteriaceae.id.3469 | UC | MRPRESSO | 0.899(0.677,1.193) | 0.485 |
| family.Erysipelotrichaceae.id.2149 | UC | IVW | 0.948(0.745,1.206) | 0.664 |
| family.Erysipelotrichaceae.id.2149 | UC | Weighted median | 1.051(0.763,1.45) | 0.760 |
| family.Erysipelotrichaceae.id.2149 | UC | Weighted mode | 1.136(0.676,1.91) | 0.630 |
| family.Erysipelotrichaceae.id.2149 | UC | MR-Robust | 0.99(0.703,1.393) | 0.953 |
| family.Erysipelotrichaceae.id.2149 | UC | MR-Egger | 0.68(0.236,1.96) | 0.475 |
| family.Erysipelotrichaceae.id.2149 | UC | MRRAPS | 0.965(0.742,1.253) | 0.787 |
| family.Erysipelotrichaceae.id.2149 | UC | MRPRESSO | 0.948(0.776,1.158) | 0.610 |
| family.FamilyXI.id.1936 | UC | IVW | 0.985(0.864,1.122) | 0.816 |
| family.FamilyXI.id.1936 | UC | Weighted median | 1.03(0.874,1.214) | 0.725 |
| family.FamilyXI.id.1936 | UC | Weighted mode | 1.087(0.844,1.401) | 0.516 |
| family.FamilyXI.id.1936 | UC | MR-Robust | 1.002(0.848,1.184) | 0.980 |
| family.FamilyXI.id.1936 | UC | MR-Egger | 1.166(0.472,2.882) | 0.739 |
| family.FamilyXI.id.1936 | UC | MRRAPS | 0.999(0.874,1.143) | 0.993 |
| family.FamilyXI.id.1936 | UC | MRPRESSO | 0.985(0.864,1.122) | 0.823 |
| family.FamilyXIII.id.1957 | UC | IVW | 0.916(0.713,1.177) | 0.493 |
| family.FamilyXIII.id.1957 | UC | Weighted median | 0.909(0.646,1.278) | 0.582 |
| family.FamilyXIII.id.1957 | UC | Weighted mode | 0.889(0.537,1.47) | 0.646 |
| family.FamilyXIII.id.1957 | UC | MR-Robust | 0.92(0.701,1.208) | 0.549 |
| family.FamilyXIII.id.1957 | UC | MR-Egger | 0.921(0.323,2.627) | 0.878 |
| family.FamilyXIII.id.1957 | UC | MRRAPS | 0.917(0.7,1.202) | 0.530 |
| family.FamilyXIII.id.1957 | UC | MRPRESSO | 0.916(0.716,1.173) | 0.502 |
| family.Lachnospiraceae.id.1987 | UC | IVW | 1.139(0.937,1.385) | 0.192 |
| family.Lachnospiraceae.id.1987 | UC | Weighted median | 1.208(0.921,1.586) | 0.173 |
| family.Lachnospiraceae.id.1987 | UC | Weighted mode | 1.272(0.855,1.892) | 0.235 |
| family.Lachnospiraceae.id.1987 | UC | MR-Robust | 1.152(0.95,1.396) | 0.151 |
| family.Lachnospiraceae.id.1987 | UC | MR-Egger | 1.405(0.867,2.275) | 0.167 |
| family.Lachnospiraceae.id.1987 | UC | MRRAPS | 1.152(0.933,1.422) | 0.189 |
| family.Lachnospiraceae.id.1987 | UC | MRPRESSO | 1.139(0.937,1.385) | 0.211 |
| family.Lactobacillaceae.id.1836 | UC | IVW | 0.857(0.713,1.03) | 0.100 |
| family.Lactobacillaceae.id.1836 | UC | Weighted median | 0.799(0.633,1.011) | 0.061 |
| family.Lactobacillaceae.id.1836 | UC | Weighted mode | 0.716(0.452,1.132) | 0.153 |
| family.Lactobacillaceae.id.1836 | UC | MR-Robust | 0.856(0.704,1.04) | 0.118 |
| family.Lactobacillaceae.id.1836 | UC | MR-Egger | 0.816(0.478,1.393) | 0.456 |
| family.Lactobacillaceae.id.1836 | UC | MRRAPS | 0.84(0.671,1.051) | 0.127 |
| family.Lactobacillaceae.id.1836 | UC | MRPRESSO | 0.857(0.713,1.03) | 0.135 |
| family.Methanobacteriaceae.id.121 | UC | IVW | 0.96(0.827,1.115) | 0.594 |
| family.Methanobacteriaceae.id.121 | UC | Weighted median | 0.907(0.771,1.066) | 0.234 |
| family.Methanobacteriaceae.id.121 | UC | Weighted mode | 0.9(0.702,1.156) | 0.410 |
| family.Methanobacteriaceae.id.121 | UC | MR-Robust | 0.948(0.778,1.156) | 0.599 |
| family.Methanobacteriaceae.id.121 | UC | MR-Egger | 1.027(0.535,1.972) | 0.937 |
| family.Methanobacteriaceae.id.121 | UC | MRRAPS | 0.969(0.832,1.13) | 0.690 |
| family.Methanobacteriaceae.id.121 | UC | MRPRESSO | 0.96(0.827,1.115) | 0.606 |
| family.Oxalobacteraceae.id.2966 | UC | IVW | 1.109(0.98,1.255) | 0.101 |
| family.Oxalobacteraceae.id.2966 | UC | Weighted median | 1.043(0.882,1.234) | 0.622 |
| family.Oxalobacteraceae.id.2966 | UC | Weighted mode | 1(0.755,1.324) | 0.998 |
| family.Oxalobacteraceae.id.2966 | UC | MR-Robust | 1.094(0.953,1.256) | 0.201 |
| family.Oxalobacteraceae.id.2966 | UC | MR-Egger | 0.955(0.575,1.585) | 0.858 |
| family.Oxalobacteraceae.id.2966 | UC | MRRAPS | 1.096(0.962,1.249) | 0.168 |
| family.Oxalobacteraceae.id.2966 | UC | MRPRESSO | 1.109(0.98,1.255) | 0.125 |
| family.Pasteurellaceae.id.3689 | UC | IVW | 1.036(0.873,1.23) | 0.682 |
| family.Pasteurellaceae.id.3689 | UC | Weighted median | 0.988(0.798,1.223) | 0.911 |
| family.Pasteurellaceae.id.3689 | UC | Weighted mode | 0.849(0.594,1.213) | 0.368 |
| family.Pasteurellaceae.id.3689 | UC | MR-Robust | 1.021(0.835,1.248) | 0.838 |
| family.Pasteurellaceae.id.3689 | UC | MR-Egger | 0.786(0.546,1.131) | 0.195 |
| family.Pasteurellaceae.id.3689 | UC | MRRAPS | 0.997(0.831,1.197) | 0.977 |
| family.Pasteurellaceae.id.3689 | UC | MRPRESSO | 1.036(0.873,1.23) | 0.688 |
| family.Peptococcaceae.id.2024 | UC | IVW | 0.819(0.682,0.985) | 0.033 |
| family.Peptococcaceae.id.2024 | UC | Weighted median | 0.833(0.646,1.074) | 0.159 |
| family.Peptococcaceae.id.2024 | UC | Weighted mode | 0.805(0.567,1.144) | 0.227 |
| family.Peptococcaceae.id.2024 | UC | MR-Robust | 0.819(0.705,0.952) | 0.009 |
| family.Peptococcaceae.id.2024 | UC | MR-Egger | 0.881(0.565,1.375) | 0.578 |
| family.Peptococcaceae.id.2024 | UC | MRRAPS | 0.816(0.667,0.998) | 0.047 |
| family.Peptococcaceae.id.2024 | UC | MRPRESSO | 0.819(0.701,0.957) | 0.033 |
| family.Peptostreptococcaceae.id.2042 | UC | IVW | 1.025(0.844,1.244) | 0.806 |
| family.Peptostreptococcaceae.id.2042 | UC | Weighted median | 1.187(0.902,1.563) | 0.221 |
| family.Peptostreptococcaceae.id.2042 | UC | Weighted mode | 1.211(0.755,1.943) | 0.427 |
| family.Peptostreptococcaceae.id.2042 | UC | MR-Robust | 1.124(0.663,1.907) | 0.663 |
| family.Peptostreptococcaceae.id.2042 | UC | MR-Egger | 0.778(0.501,1.207) | 0.262 |
| family.Peptostreptococcaceae.id.2042 | UC | MRRAPS | 1.025(0.812,1.292) | 0.838 |
| family.Peptostreptococcaceae.id.2042 | UC | MRPRESSO | 1.025(0.844,1.244) | 0.810 |
| family.Porphyromonadaceae.id.943 | UC | IVW | 0.968(0.681,1.376) | 0.857 |
| family.Porphyromonadaceae.id.943 | UC | Weighted median | 0.991(0.673,1.458) | 0.962 |
| family.Porphyromonadaceae.id.943 | UC | Weighted mode | 0.989(0.523,1.869) | 0.972 |
| family.Porphyromonadaceae.id.943 | UC | MR-Robust | 0.963(0.68,1.362) | 0.830 |
| family.Porphyromonadaceae.id.943 | UC | MR-Egger | 2.74(0.807,9.304) | 0.106 |
| family.Porphyromonadaceae.id.943 | UC | MRRAPS | 1(0.692,1.446) | 0.999 |
| family.Porphyromonadaceae.id.943 | UC | MRPRESSO | 0.968(0.681,1.376) | 0.861 |
| family.Prevotellaceae.id.960 | UC | IVW | 0.973(0.74,1.279) | 0.843 |
| family.Prevotellaceae.id.960 | UC | Weighted median | 0.919(0.696,1.214) | 0.553 |
| family.Prevotellaceae.id.960 | UC | Weighted mode | 0.849(0.552,1.306) | 0.457 |
| family.Prevotellaceae.id.960 | UC | MR-Robust | 0.946(0.711,1.259) | 0.702 |
| family.Prevotellaceae.id.960 | UC | MR-Egger | 1.075(0.389,2.97) | 0.889 |
| family.Prevotellaceae.id.960 | UC | MRRAPS | 0.991(0.738,1.33) | 0.952 |
| family.Prevotellaceae.id.960 | UC | MRPRESSO | 0.973(0.74,1.279) | 0.846 |
| family.Rhodospirillaceae.id.2717 | UC | IVW | 1.079(0.883,1.317) | 0.457 |
| family.Rhodospirillaceae.id.2717 | UC | Weighted median | 1.079(0.858,1.355) | 0.516 |
| family.Rhodospirillaceae.id.2717 | UC | Weighted mode | 1.047(0.712,1.54) | 0.814 |
| family.Rhodospirillaceae.id.2717 | UC | MR-Robust | 1.078(0.878,1.324) | 0.474 |
| family.Rhodospirillaceae.id.2717 | UC | MR-Egger | 0.601(0.194,1.862) | 0.378 |
| family.Rhodospirillaceae.id.2717 | UC | MRRAPS | 1.078(0.87,1.335) | 0.495 |
| family.Rhodospirillaceae.id.2717 | UC | MRPRESSO | 1.079(0.883,1.317) | 0.469 |
| family.Rikenellaceae.id.967 | UC | IVW | 1.133(0.922,1.392) | 0.234 |
| family.Rikenellaceae.id.967 | UC | Weighted median | 1.095(0.845,1.419) | 0.492 |
| family.Rikenellaceae.id.967 | UC | Weighted mode | 1.103(0.719,1.692) | 0.653 |
| family.Rikenellaceae.id.967 | UC | MR-Robust | 1.14(0.939,1.386) | 0.186 |
| family.Rikenellaceae.id.967 | UC | MR-Egger | 1.179(0.604,2.302) | 0.629 |
| family.Rikenellaceae.id.967 | UC | MRRAPS | 1.154(0.938,1.42) | 0.175 |
| family.Rikenellaceae.id.967 | UC | MRPRESSO | 1.133(0.922,1.392) | 0.248 |
| family.Ruminococcaceae.id.2050 | UC | IVW | 1.01(0.799,1.278) | 0.931 |
| family.Ruminococcaceae.id.2050 | UC | Weighted median | 0.896(0.638,1.257) | 0.524 |
| family.Ruminococcaceae.id.2050 | UC | Weighted mode | 0.826(0.512,1.334) | 0.435 |
| family.Ruminococcaceae.id.2050 | UC | MR-Robust | 0.998(0.75,1.329) | 0.989 |
| family.Ruminococcaceae.id.2050 | UC | MR-Egger | 0.991(0.586,1.675) | 0.973 |
| family.Ruminococcaceae.id.2050 | UC | MRRAPS | 1.009(0.77,1.323) | 0.948 |
| family.Ruminococcaceae.id.2050 | UC | MRPRESSO | 1.01(0.802,1.273) | 0.932 |
| family.Streptococcaceae.id.1850 | UC | IVW | 1.021(0.797,1.308) | 0.869 |
| family.Streptococcaceae.id.1850 | UC | Weighted median | 1.096(0.812,1.479) | 0.550 |
| family.Streptococcaceae.id.1850 | UC | Weighted mode | 1.23(0.712,2.123) | 0.457 |
| family.Streptococcaceae.id.1850 | UC | MR-Robust | 1.038(0.786,1.371) | 0.791 |
| family.Streptococcaceae.id.1850 | UC | MR-Egger | 1.098(0.416,2.896) | 0.850 |
| family.Streptococcaceae.id.1850 | UC | MRRAPS | 1.051(0.778,1.419) | 0.747 |
| family.Streptococcaceae.id.1850 | UC | MRPRESSO | 1.021(0.797,1.308) | 0.871 |
| family.unknownfamily.id.1000001214 | UC | IVW | 0.974(0.829,1.143) | 0.744 |
| family.unknownfamily.id.1000001214 | UC | Weighted median | 0.948(0.766,1.173) | 0.622 |
| family.unknownfamily.id.1000001214 | UC | Weighted mode | 0.908(0.661,1.246) | 0.550 |
| family.unknownfamily.id.1000001214 | UC | MR-Robust | 0.963(0.822,1.129) | 0.641 |
| family.unknownfamily.id.1000001214 | UC | MR-Egger | 0.934(0.577,1.514) | 0.783 |
| family.unknownfamily.id.1000001214 | UC | MRRAPS | 0.96(0.813,1.133) | 0.628 |
| family.unknownfamily.id.1000001214 | UC | MRPRESSO | 0.974(0.829,1.143) | 0.753 |
| family.unknownfamily.id.1000005471 | UC | IVW | 0.89(0.751,1.055) | 0.180 |
| family.unknownfamily.id.1000005471 | UC | Weighted median | 0.873(0.698,1.091) | 0.232 |
| family.unknownfamily.id.1000005471 | UC | Weighted mode | 0.875(0.59,1.296) | 0.504 |
| family.unknownfamily.id.1000005471 | UC | MR-Robust | 0.901(0.719,1.129) | 0.364 |
| family.unknownfamily.id.1000005471 | UC | MR-Egger | 0.691(0.419,1.14) | 0.148 |
| family.unknownfamily.id.1000005471 | UC | MRRAPS | 0.888(0.737,1.07) | 0.211 |
| family.unknownfamily.id.1000005471 | UC | MRPRESSO | 0.89(0.798,0.992) | 0.056 |
| family.unknownfamily.id.1000006161 | UC | IVW | 1.111(0.927,1.331) | 0.255 |
| family.unknownfamily.id.1000006161 | UC | Weighted median | 1.11(0.925,1.331) | 0.262 |
| family.unknownfamily.id.1000006161 | UC | Weighted mode | 1.088(0.811,1.46) | 0.573 |
| family.unknownfamily.id.1000006161 | UC | MR-Robust | 1.102(0.923,1.315) | 0.282 |
| family.unknownfamily.id.1000006161 | UC | MR-Egger | 0.552(0.287,1.063) | 0.076 |
| family.unknownfamily.id.1000006161 | UC | MRRAPS | 1.067(0.888,1.283) | 0.489 |
| family.unknownfamily.id.1000006161 | UC | MRPRESSO | 1.111(0.927,1.331) | 0.276 |
| family.Veillonellaceae.id.2172 | UC | IVW | 0.945(0.802,1.113) | 0.498 |
| family.Veillonellaceae.id.2172 | UC | Weighted median | 1.013(0.807,1.271) | 0.912 |
| family.Veillonellaceae.id.2172 | UC | Weighted mode | 1.043(0.744,1.462) | 0.809 |
| family.Veillonellaceae.id.2172 | UC | MR-Robust | 0.95(0.833,1.084) | 0.445 |
| family.Veillonellaceae.id.2172 | UC | MR-Egger | 0.971(0.678,1.392) | 0.873 |
| family.Veillonellaceae.id.2172 | UC | MRRAPS | 0.951(0.796,1.136) | 0.578 |
| family.Veillonellaceae.id.2172 | UC | MRPRESSO | 0.945(0.824,1.083) | 0.428 |
| family.Verrucomicrobiaceae.id.4036 | UC | IVW | 0.975(0.771,1.233) | 0.832 |
| family.Verrucomicrobiaceae.id.4036 | UC | Weighted median | 0.947(0.708,1.268) | 0.716 |
| family.Verrucomicrobiaceae.id.4036 | UC | Weighted mode | 0.7(0.385,1.271) | 0.241 |
| family.Verrucomicrobiaceae.id.4036 | UC | MR-Robust | 0.974(0.758,1.251) | 0.835 |
| family.Verrucomicrobiaceae.id.4036 | UC | MR-Egger | 0.454(0.209,0.987) | 0.046 |
| family.Verrucomicrobiaceae.id.4036 | UC | MRRAPS | 0.92(0.688,1.23) | 0.572 |
| family.Verrucomicrobiaceae.id.4036 | UC | MRPRESSO | 0.975(0.771,1.233) | 0.836 |
| family.Victivallaceae.id.2255 | UC | IVW | 1.057(0.924,1.211) | 0.419 |
| family.Victivallaceae.id.2255 | UC | Weighted median | 1.052(0.898,1.232) | 0.533 |
| family.Victivallaceae.id.2255 | UC | Weighted mode | 1.067(0.81,1.407) | 0.643 |
| family.Victivallaceae.id.2255 | UC | MR-Robust | 1.071(0.928,1.235) | 0.349 |
| family.Victivallaceae.id.2255 | UC | MR-Egger | 0.706(0.385,1.296) | 0.261 |
| family.Victivallaceae.id.2255 | UC | MRRAPS | 1.074(0.934,1.235) | 0.315 |
| family.Victivallaceae.id.2255 | UC | MRPRESSO | 1.057(0.924,1.211) | 0.435 |
| genus Clostridiuminnocuumgroup.id.14397 | UC | IVW | 0.95(0.823,1.096) | 0.480 |
| genus Clostridiuminnocuumgroup.id.14397 | UC | Weighted median | 0.938(0.775,1.135) | 0.512 |
| genus Clostridiuminnocuumgroup.id.14397 | UC | Weighted mode | 0.923(0.714,1.193) | 0.539 |
| genus Clostridiuminnocuumgroup.id.14397 | UC | MR-Robust | 0.925(0.82,1.043) | 0.201 |
| genus Clostridiuminnocuumgroup.id.14397 | UC | MR-Egger | 0.95(0.438,2.061) | 0.896 |
| genus Clostridiuminnocuumgroup.id.14397 | UC | MRRAPS | 0.929(0.797,1.083) | 0.346 |
| genus Clostridiuminnocuumgroup.id.14397 | UC | MRPRESSO | 0.95(0.823,1.096) | 0.500 |
| genus Eubacteriumbrachygroup.id.11296 | UC | IVW | 0.97(0.843,1.116) | 0.674 |
| genus Eubacteriumbrachygroup.id.11296 | UC | Weighted median | 0.939(0.78,1.132) | 0.510 |
| genus Eubacteriumbrachygroup.id.11296 | UC | Weighted mode | 0.911(0.692,1.2) | 0.508 |
| genus Eubacteriumbrachygroup.id.11296 | UC | MR-Robust | 0.93(0.745,1.16) | 0.519 |
| genus Eubacteriumbrachygroup.id.11296 | UC | MR-Egger | 0.718(0.382,1.348) | 0.303 |
| genus Eubacteriumbrachygroup.id.11296 | UC | MRRAPS | 0.961(0.824,1.12) | 0.610 |
| genus Eubacteriumbrachygroup.id.11296 | UC | MRPRESSO | 0.97(0.874,1.077) | 0.588 |
| genus Eubacteriumcoprostanoligenesgroup.id.11375 | UC | IVW | 0.904(0.718,1.138) | 0.389 |
| genus Eubacteriumcoprostanoligenesgroup.id.11375 | UC | Weighted median | 0.883(0.647,1.206) | 0.435 |
| genus Eubacteriumcoprostanoligenesgroup.id.11375 | UC | Weighted mode | 0.887(0.515,1.528) | 0.665 |
| genus Eubacteriumcoprostanoligenesgroup.id.11375 | UC | MR-Robust | 0.907(0.72,1.144) | 0.410 |
| genus Eubacteriumcoprostanoligenesgroup.id.11375 | UC | MR-Egger | 1.538(0.625,3.781) | 0.349 |
| genus Eubacteriumcoprostanoligenesgroup.id.11375 | UC | MRRAPS | 0.907(0.707,1.165) | 0.446 |
| genus Eubacteriumcoprostanoligenesgroup.id.11375 | UC | MRPRESSO | 0.904(0.74,1.103) | 0.340 |
| genus Eubacteriumeligensgroup.id.14372 | UC | IVW | 1.405(1.058,1.865) | 0.019 |
| genus Eubacteriumeligensgroup.id.14372 | UC | Weighted median | 1.365(0.95,1.96) | 0.092 |
| genus Eubacteriumeligensgroup.id.14372 | UC | Weighted mode | 1.293(0.73,2.29) | 0.379 |
| genus Eubacteriumeligensgroup.id.14372 | UC | MR-Robust | 1.402(1.099,1.789) | 0.007 |
| genus Eubacteriumeligensgroup.id.14372 | UC | MR-Egger | 1.672(0.573,4.882) | 0.347 |
| genus Eubacteriumeligensgroup.id.14372 | UC | MRRAPS | 1.413(1.03,1.938) | 0.032 |
| genus Eubacteriumeligensgroup.id.14372 | UC | MRPRESSO | 1.405(1.172,1.684) | 0.008 |
| genus Eubacteriumfissicatenagroup.id.14373 | UC | IVW | 1.06(0.909,1.236) | 0.456 |
| genus Eubacteriumfissicatenagroup.id.14373 | UC | Weighted median | 1.098(0.902,1.336) | 0.350 |
| genus Eubacteriumfissicatenagroup.id.14373 | UC | Weighted mode | 1.08(0.774,1.505) | 0.652 |
| genus Eubacteriumfissicatenagroup.id.14373 | UC | MR-Robust | 1.058(0.902,1.242) | 0.488 |
| genus Eubacteriumfissicatenagroup.id.14373 | UC | MR-Egger | 0.997(0.413,2.407) | 0.995 |
| genus Eubacteriumfissicatenagroup.id.14373 | UC | MRRAPS | 1.053(0.889,1.248) | 0.549 |
| genus Eubacteriumfissicatenagroup.id.14373 | UC | MRPRESSO | 1.06(0.909,1.236) | 0.478 |
| genus Eubacteriumhalliigroup.id.11338 | UC | IVW | 0.916(0.717,1.172) | 0.487 |
| genus Eubacteriumhalliigroup.id.11338 | UC | Weighted median | 1.022(0.768,1.361) | 0.879 |
| genus Eubacteriumhalliigroup.id.11338 | UC | Weighted mode | 1.045(0.653,1.673) | 0.853 |
| genus Eubacteriumhalliigroup.id.11338 | UC | MR-Robust | 0.914(0.719,1.161) | 0.461 |
| genus Eubacteriumhalliigroup.id.11338 | UC | MR-Egger | 1.676(1.081,2.6) | 0.021 |
| genus Eubacteriumhalliigroup.id.11338 | UC | MRRAPS | 0.965(0.757,1.23) | 0.774 |
| genus Eubacteriumhalliigroup.id.11338 | UC | MRPRESSO | 0.916(0.717,1.172) | 0.498 |
| genus Eubacteriumnodatumgroup.id.11297 | UC | IVW | 0.983(0.88,1.098) | 0.763 |
| genus Eubacteriumnodatumgroup.id.11297 | UC | Weighted median | 1.012(0.878,1.167) | 0.868 |
| genus Eubacteriumnodatumgroup.id.11297 | UC | Weighted mode | 1.071(0.853,1.345) | 0.553 |
| genus Eubacteriumnodatumgroup.id.11297 | UC | MR-Robust | 0.986(0.897,1.083) | 0.769 |
| genus Eubacteriumnodatumgroup.id.11297 | UC | MR-Egger | 1.111(0.682,1.811) | 0.673 |
| genus Eubacteriumnodatumgroup.id.11297 | UC | MRRAPS | 0.983(0.871,1.11) | 0.780 |
| genus Eubacteriumnodatumgroup.id.11297 | UC | MRPRESSO | 0.983(0.917,1.054) | 0.641 |
| genus Eubacteriumoxidoreducensgroup.id.11339 | UC | IVW | 0.847(0.672,1.067) | 0.159 |
| genus Eubacteriumoxidoreducensgroup.id.11339 | UC | Weighted median | 0.804(0.607,1.064) | 0.127 |
| genus Eubacteriumoxidoreducensgroup.id.11339 | UC | Weighted mode | 0.791(0.559,1.119) | 0.186 |
| genus Eubacteriumoxidoreducensgroup.id.11339 | UC | MR-Robust | 0.826(0.572,1.193) | 0.308 |
| genus Eubacteriumoxidoreducensgroup.id.11339 | UC | MR-Egger | 0.83(0.412,1.669) | 0.601 |
| genus Eubacteriumoxidoreducensgroup.id.11339 | UC | MRRAPS | 0.845(0.655,1.091) | 0.197 |
| genus Eubacteriumoxidoreducensgroup.id.11339 | UC | MRPRESSO | 0.847(0.718,0.999) | 0.143 |
| genus Eubacteriumrectalegroup.id.14374 | UC | IVW | 1.217(0.879,1.685) | 0.238 |
| genus Eubacteriumrectalegroup.id.14374 | UC | Weighted median | 1.515(1.021,2.25) | 0.039 |
| genus Eubacteriumrectalegroup.id.14374 | UC | Weighted mode | 1.672(0.895,3.123) | 0.107 |
| genus Eubacteriumrectalegroup.id.14374 | UC | MR-Robust | 1.241(0.854,1.803) | 0.258 |
| genus Eubacteriumrectalegroup.id.14374 | UC | MR-Egger | 0.887(0.256,3.082) | 0.851 |
| genus Eubacteriumrectalegroup.id.14374 | UC | MRRAPS | 1.247(0.882,1.764) | 0.212 |
| genus Eubacteriumrectalegroup.id.14374 | UC | MRPRESSO | 1.217(0.879,1.685) | 0.272 |
| genus Eubacteriumruminantiumgroup.id.11340 | UC | IVW | 1.124(0.975,1.294) | 0.106 |
| genus Eubacteriumruminantiumgroup.id.11340 | UC | Weighted median | 1.208(1,1.458) | 0.050 |
| genus Eubacteriumruminantiumgroup.id.11340 | UC | Weighted mode | 1.367(0.947,1.973) | 0.095 |
| genus Eubacteriumruminantiumgroup.id.11340 | UC | MR-Robust | 1.144(0.967,1.355) | 0.118 |
| genus Eubacteriumruminantiumgroup.id.11340 | UC | MR-Egger | 1.186(0.69,2.039) | 0.537 |
| genus Eubacteriumruminantiumgroup.id.11340 | UC | MRRAPS | 1.156(0.991,1.348) | 0.065 |
| genus Eubacteriumruminantiumgroup.id.11340 | UC | MRPRESSO | 1.124(0.975,1.294) | 0.125 |
| genus Eubacteriumventriosumgroup.id.11341 | UC | IVW | 0.727(0.561,0.942) | 0.016 |
| genus Eubacteriumventriosumgroup.id.11341 | UC | Weighted median | 0.671(0.494,0.912) | 0.011 |
| genus Eubacteriumventriosumgroup.id.11341 | UC | Weighted mode | 0.695(0.405,1.193) | 0.187 |
| genus Eubacteriumventriosumgroup.id.11341 | UC | MR-Robust | 0.733(0.573,0.937) | 0.013 |
| genus Eubacteriumventriosumgroup.id.11341 | UC | MR-Egger | 0.448(0.14,1.433) | 0.176 |
| genus Eubacteriumventriosumgroup.id.11341 | UC | MRRAPS | 0.722(0.556,0.937) | 0.014 |
| genus Eubacteriumventriosumgroup.id.11341 | UC | MRPRESSO | 0.727(0.561,0.942) | 0.030 |
| genus Eubacteriumxylanophilumgroup.id.14375 | UC | IVW | 1.097(0.828,1.454) | 0.519 |
| genus Eubacteriumxylanophilumgroup.id.14375 | UC | Weighted median | 1.112(0.787,1.572) | 0.546 |
| genus Eubacteriumxylanophilumgroup.id.14375 | UC | Weighted mode | 0.954(0.492,1.853) | 0.890 |
| genus Eubacteriumxylanophilumgroup.id.14375 | UC | MR-Robust | 1.107(0.815,1.504) | 0.515 |
| genus Eubacteriumxylanophilumgroup.id.14375 | UC | MR-Egger | 0.378(0.176,0.814) | 0.013 |
| genus Eubacteriumxylanophilumgroup.id.14375 | UC | MRRAPS | 1.08(0.797,1.463) | 0.619 |
| genus Eubacteriumxylanophilumgroup.id.14375 | UC | MRPRESSO | 1.097(0.828,1.454) | 0.537 |
| genus Ruminococcusgauvreauiigroup.id.11342 | UC | IVW | 0.889(0.676,1.168) | 0.398 |
| genus Ruminococcusgauvreauiigroup.id.11342 | UC | Weighted median | 0.917(0.678,1.24) | 0.574 |
| genus Ruminococcusgauvreauiigroup.id.11342 | UC | Weighted mode | 0.972(0.61,1.548) | 0.905 |
| genus Ruminococcusgauvreauiigroup.id.11342 | UC | MR-Robust | 0.911(0.703,1.18) | 0.479 |
| genus Ruminococcusgauvreauiigroup.id.11342 | UC | MR-Egger | 1.113(0.299,4.142) | 0.873 |
| genus Ruminococcusgauvreauiigroup.id.11342 | UC | MRRAPS | 0.903(0.698,1.169) | 0.439 |
| genus Ruminococcusgauvreauiigroup.id.11342 | UC | MRPRESSO | 0.889(0.676,1.168) | 0.416 |
| genus Ruminococcusgnavusgroup.id.14376 | UC | IVW | 0.958(0.826,1.111) | 0.569 |
| genus Ruminococcusgnavusgroup.id.14376 | UC | Weighted median | 0.937(0.771,1.14) | 0.517 |
| genus Ruminococcusgnavusgroup.id.14376 | UC | Weighted mode | 0.908(0.664,1.241) | 0.544 |
| genus Ruminococcusgnavusgroup.id.14376 | UC | MR-Robust | 0.958(0.82,1.119) | 0.588 |
| genus Ruminococcusgnavusgroup.id.14376 | UC | MR-Egger | 0.654(0.33,1.299) | 0.225 |
| genus Ruminococcusgnavusgroup.id.14376 | UC | MRRAPS | 0.959(0.817,1.127) | 0.613 |
| genus Ruminococcusgnavusgroup.id.14376 | UC | MRPRESSO | 0.958(0.835,1.099) | 0.551 |
| genus Ruminococcustorquesgroup.id.14377 | UC | IVW | 1.02(0.747,1.393) | 0.900 |
| genus Ruminococcustorquesgroup.id.14377 | UC | Weighted median | 0.789(0.558,1.115) | 0.179 |
| genus Ruminococcustorquesgroup.id.14377 | UC | Weighted mode | 0.748(0.491,1.138) | 0.175 |
| genus Ruminococcustorquesgroup.id.14377 | UC | MR-Robust | 0.857(0.281,2.612) | 0.787 |
| genus Ruminococcustorquesgroup.id.14377 | UC | MR-Egger | 0.49(0.246,0.975) | 0.042 |
| genus Ruminococcustorquesgroup.id.14377 | UC | MRRAPS | 0.942(0.707,1.254) | 0.680 |
| genus Ruminococcustorquesgroup.id.14377 | UC | MRPRESSO | 1.02(0.747,1.393) | 0.903 |
| genus.Actinomyces.id.423 | UC | IVW | 0.98(0.806,1.191) | 0.840 |
| genus.Actinomyces.id.423 | UC | Weighted median | 1.064(0.823,1.376) | 0.636 |
| genus.Actinomyces.id.423 | UC | Weighted mode | 1.147(0.802,1.64) | 0.454 |
| genus.Actinomyces.id.423 | UC | MR-Robust | 0.985(0.808,1.201) | 0.883 |
| genus.Actinomyces.id.423 | UC | MR-Egger | 1.492(0.871,2.556) | 0.146 |
| genus.Actinomyces.id.423 | UC | MRRAPS | 0.979(0.793,1.21) | 0.847 |
| genus.Actinomyces.id.423 | UC | MRPRESSO | 0.98(0.814,1.181) | 0.839 |
| genus.Adlercreutzia.id.812 | UC | IVW | 1.033(0.846,1.262) | 0.747 |
| genus.Adlercreutzia.id.812 | UC | Weighted median | 1.009(0.767,1.329) | 0.947 |
| genus.Adlercreutzia.id.812 | UC | Weighted mode | 0.986(0.632,1.538) | 0.949 |
| genus.Adlercreutzia.id.812 | UC | MR-Robust | 1.037(0.852,1.262) | 0.717 |
| genus.Adlercreutzia.id.812 | UC | MR-Egger | 1.52(0.606,3.814) | 0.373 |
| genus.Adlercreutzia.id.812 | UC | MRRAPS | 1.048(0.845,1.301) | 0.669 |
| genus.Adlercreutzia.id.812 | UC | MRPRESSO | 1.033(0.85,1.256) | 0.751 |
| genus.Akkermansia.id.4037 | UC | IVW | 0.975(0.771,1.233) | 0.831 |
| genus.Akkermansia.id.4037 | UC | Weighted median | 0.947(0.707,1.268) | 0.715 |
| genus.Akkermansia.id.4037 | UC | Weighted mode | 0.7(0.384,1.274) | 0.243 |
| genus.Akkermansia.id.4037 | UC | MR-Robust | 0.974(0.758,1.251) | 0.834 |
| genus.Akkermansia.id.4037 | UC | MR-Egger | 0.454(0.209,0.985) | 0.046 |
| genus.Akkermansia.id.4037 | UC | MRRAPS | 0.919(0.688,1.229) | 0.571 |
| genus.Akkermansia.id.4037 | UC | MRPRESSO | 0.975(0.771,1.233) | 0.834 |
| genus.Alistipes.id.968 | UC | IVW | 1.105(0.863,1.415) | 0.427 |
| genus.Alistipes.id.968 | UC | Weighted median | 1.177(0.847,1.636) | 0.333 |
| genus.Alistipes.id.968 | UC | Weighted mode | 1.237(0.696,2.199) | 0.468 |
| genus.Alistipes.id.968 | UC | MR-Robust | 1.112(0.861,1.436) | 0.417 |
| genus.Alistipes.id.968 | UC | MR-Egger | 3.905(1.273,11.979) | 0.017 |
| genus.Alistipes.id.968 | UC | MRRAPS | 1.139(0.867,1.497) | 0.350 |
| genus.Alistipes.id.968 | UC | MRPRESSO | 1.105(0.863,1.415) | 0.441 |
| genus.Allisonella.id.2174 | UC | IVW | 1.052(0.893,1.241) | 0.542 |
| genus.Allisonella.id.2174 | UC | Weighted median | 0.94(0.786,1.124) | 0.498 |
| genus.Allisonella.id.2174 | UC | Weighted mode | 0.906(0.718,1.141) | 0.401 |
| genus.Allisonella.id.2174 | UC | MR-Robust | 1.01(0.77,1.325) | 0.943 |
| genus.Allisonella.id.2174 | UC | MR-Egger | 0.286(0.123,0.666) | 0.004 |
| genus.Allisonella.id.2174 | UC | MRRAPS | 1.022(0.875,1.194) | 0.782 |
| genus.Allisonella.id.2174 | UC | MRPRESSO | 1.052(0.893,1.241) | 0.561 |
| genus.Alloprevotella.id.961 | UC | IVW | 1.094(0.935,1.28) | 0.262 |
| genus.Alloprevotella.id.961 | UC | Weighted median | 1.123(0.931,1.355) | 0.225 |
| genus.Alloprevotella.id.961 | UC | Weighted mode | 1.126(0.858,1.479) | 0.392 |
| genus.Alloprevotella.id.961 | UC | MR-Robust | 1.109(0.954,1.29) | 0.179 |
| genus.Alloprevotella.id.961 | UC | MR-Egger | 1.553(0.308,7.825) | 0.593 |
| genus.Alloprevotella.id.961 | UC | MRRAPS | 1.119(0.955,1.312) | 0.165 |
| genus.Alloprevotella.id.961 | UC | MRPRESSO | 1.094(0.935,1.28) | 0.313 |
| genus.Anaerofilum.id.2053 | UC | IVW | 0.93(0.789,1.097) | 0.389 |
| genus.Anaerofilum.id.2053 | UC | Weighted median | 0.991(0.819,1.199) | 0.924 |
| genus.Anaerofilum.id.2053 | UC | Weighted mode | 0.991(0.742,1.323) | 0.951 |
| genus.Anaerofilum.id.2053 | UC | MR-Robust | 0.943(0.803,1.106) | 0.469 |
| genus.Anaerofilum.id.2053 | UC | MR-Egger | 1.177(0.488,2.834) | 0.717 |
| genus.Anaerofilum.id.2053 | UC | MRRAPS | 0.94(0.798,1.106) | 0.455 |
| genus.Anaerofilum.id.2053 | UC | MRPRESSO | 0.93(0.789,1.097) | 0.409 |
| genus.Anaerostipes.id.1991 | UC | IVW | 1.033(0.818,1.304) | 0.785 |
| genus.Anaerostipes.id.1991 | UC | Weighted median | 1.007(0.746,1.359) | 0.964 |
| genus.Anaerostipes.id.1991 | UC | Weighted mode | 1.016(0.62,1.664) | 0.950 |
| genus.Anaerostipes.id.1991 | UC | MR-Robust | 1.015(0.781,1.319) | 0.912 |
| genus.Anaerostipes.id.1991 | UC | MR-Egger | 0.944(0.403,2.21) | 0.895 |
| genus.Anaerostipes.id.1991 | UC | MRRAPS | 1.032(0.8,1.332) | 0.807 |
| genus.Anaerostipes.id.1991 | UC | MRPRESSO | 1.033(0.898,1.187) | 0.657 |
| genus.Anaerotruncus.id.2054 | UC | IVW | 0.858(0.666,1.106) | 0.237 |
| genus.Anaerotruncus.id.2054 | UC | Weighted median | 0.889(0.642,1.233) | 0.482 |
| genus.Anaerotruncus.id.2054 | UC | Weighted mode | 0.909(0.517,1.596) | 0.739 |
| genus.Anaerotruncus.id.2054 | UC | MR-Robust | 0.852(0.659,1.101) | 0.222 |
| genus.Anaerotruncus.id.2054 | UC | MR-Egger | 1.121(0.49,2.565) | 0.787 |
| genus.Anaerotruncus.id.2054 | UC | MRRAPS | 0.844(0.644,1.105) | 0.217 |
| genus.Anaerotruncus.id.2054 | UC | MRPRESSO | 0.858(0.666,1.106) | 0.260 |
| genus.Bacteroides.id.918 | UC | IVW | 0.895(0.66,1.216) | 0.479 |
| genus.Bacteroides.id.918 | UC | Weighted median | 0.882(0.59,1.321) | 0.543 |
| genus.Bacteroides.id.918 | UC | Weighted mode | 0.887(0.462,1.703) | 0.719 |
| genus.Bacteroides.id.918 | UC | MR-Robust | 0.89(0.622,1.273) | 0.523 |
| genus.Bacteroides.id.918 | UC | MR-Egger | 1.534(0.249,9.464) | 0.645 |
| genus.Bacteroides.id.918 | UC | MRRAPS | 0.884(0.636,1.229) | 0.464 |
| genus.Bacteroides.id.918 | UC | MRPRESSO | 0.895(0.665,1.206) | 0.491 |
| genus.Barnesiella.id.944 | UC | IVW | 1.182(0.962,1.452) | 0.111 |
| genus.Barnesiella.id.944 | UC | Weighted median | 1.139(0.863,1.504) | 0.359 |
| genus.Barnesiella.id.944 | UC | Weighted mode | 1.087(0.699,1.69) | 0.713 |
| genus.Barnesiella.id.944 | UC | MR-Robust | 1.176(0.978,1.414) | 0.084 |
| genus.Barnesiella.id.944 | UC | MR-Egger | 1.296(0.651,2.581) | 0.461 |
| genus.Barnesiella.id.944 | UC | MRRAPS | 1.184(0.947,1.48) | 0.139 |
| genus.Barnesiella.id.944 | UC | MRPRESSO | 1.182(0.986,1.417) | 0.094 |
| genus.Bifidobacterium.id.436 | UC | IVW | 0.806(0.667,0.973) | 0.025 |
| genus.Bifidobacterium.id.436 | UC | Weighted median | 0.915(0.732,1.143) | 0.432 |
| genus.Bifidobacterium.id.436 | UC | Weighted mode | 0.952(0.683,1.326) | 0.771 |
| genus.Bifidobacterium.id.436 | UC | MR-Robust | 0.855(0.71,1.03) | 0.099 |
| genus.Bifidobacterium.id.436 | UC | MR-Egger | 0.617(0.342,1.111) | 0.108 |
| genus.Bifidobacterium.id.436 | UC | MRRAPS | 0.8(0.65,0.986) | 0.036 |
| genus.Bifidobacterium.id.436 | UC | MRPRESSO | 0.806(0.667,0.973) | 0.035 |
| genus.Bilophila.id.3170 | UC | IVW | 1.152(0.938,1.416) | 0.178 |
| genus.Bilophila.id.3170 | UC | Weighted median | 1.125(0.866,1.462) | 0.376 |
| genus.Bilophila.id.3170 | UC | Weighted mode | 1.138(0.745,1.739) | 0.549 |
| genus.Bilophila.id.3170 | UC | MR-Robust | 1.166(0.965,1.409) | 0.112 |
| genus.Bilophila.id.3170 | UC | MR-Egger | 0.74(0.287,1.91) | 0.534 |
| genus.Bilophila.id.3170 | UC | MRRAPS | 1.155(0.92,1.45) | 0.215 |
| genus.Bilophila.id.3170 | UC | MRPRESSO | 1.152(1.029,1.29) | 0.030 |
| genus.Blautia.id.1992 | UC | IVW | 1.025(0.825,1.274) | 0.824 |
| genus.Blautia.id.1992 | UC | Weighted median | 1.136(0.848,1.523) | 0.392 |
| genus.Blautia.id.1992 | UC | Weighted mode | 1.138(0.771,1.678) | 0.515 |
| genus.Blautia.id.1992 | UC | MR-Robust | 1.06(0.825,1.361) | 0.651 |
| genus.Blautia.id.1992 | UC | MR-Egger | 1.268(0.784,2.053) | 0.333 |
| genus.Blautia.id.1992 | UC | MRRAPS | 1.043(0.823,1.321) | 0.728 |
| genus.Blautia.id.1992 | UC | MRPRESSO | 1.025(0.854,1.23) | 0.795 |
| genus.Butyricicoccus.id.2055 | UC | IVW | 1.238(0.925,1.656) | 0.151 |
| genus.Butyricicoccus.id.2055 | UC | Weighted median | 1.04(0.756,1.43) | 0.811 |
| genus.Butyricicoccus.id.2055 | UC | Weighted mode | 1.057(0.762,1.466) | 0.741 |
| genus.Butyricicoccus.id.2055 | UC | MR-Robust | 1.223(0.932,1.604) | 0.147 |
| genus.Butyricicoccus.id.2055 | UC | MR-Egger | 0.942(0.564,1.574) | 0.821 |
| genus.Butyricicoccus.id.2055 | UC | MRRAPS | 1.207(0.921,1.582) | 0.172 |
| genus.Butyricicoccus.id.2055 | UC | MRPRESSO | 1.238(0.925,1.656) | 0.194 |
| genus.Butyricimonas.id.945 | UC | IVW | 1.022(0.854,1.222) | 0.814 |
| genus.Butyricimonas.id.945 | UC | Weighted median | 1.148(0.903,1.46) | 0.260 |
| genus.Butyricimonas.id.945 | UC | Weighted mode | 1.21(0.811,1.807) | 0.350 |
| genus.Butyricimonas.id.945 | UC | MR-Robust | 1.105(0.607,2.011) | 0.743 |
| genus.Butyricimonas.id.945 | UC | MR-Egger | 0.866(0.478,1.569) | 0.634 |
| genus.Butyricimonas.id.945 | UC | MRRAPS | 1.032(0.849,1.253) | 0.754 |
| genus.Butyricimonas.id.945 | UC | MRPRESSO | 1.022(0.883,1.182) | 0.777 |
| genus.Butyrivibrio.id.1993 | UC | IVW | 1(0.905,1.105) | 0.999 |
| genus.Butyrivibrio.id.1993 | UC | Weighted median | 1.014(0.883,1.165) | 0.847 |
| genus.Butyrivibrio.id.1993 | UC | Weighted mode | 1.017(0.794,1.302) | 0.894 |
| genus.Butyrivibrio.id.1993 | UC | MR-Robust | 0.993(0.898,1.099) | 0.895 |
| genus.Butyrivibrio.id.1993 | UC | MR-Egger | 1.28(0.806,2.032) | 0.296 |
| genus.Butyrivibrio.id.1993 | UC | MRRAPS | 0.985(0.885,1.096) | 0.776 |
| genus.Butyrivibrio.id.1993 | UC | MRPRESSO | 1(0.905,1.105) | 0.999 |
| genus.CandidatusSoleaferrea.id.11350 | UC | IVW | 0.936(0.793,1.104) | 0.430 |
| genus.CandidatusSoleaferrea.id.11350 | UC | Weighted median | 0.867(0.697,1.079) | 0.201 |
| genus.CandidatusSoleaferrea.id.11350 | UC | Weighted mode | 0.819(0.57,1.177) | 0.280 |
| genus.CandidatusSoleaferrea.id.11350 | UC | MR-Robust | 0.921(0.741,1.145) | 0.461 |
| genus.CandidatusSoleaferrea.id.11350 | UC | MR-Egger | 1.186(0.208,6.782) | 0.848 |
| genus.CandidatusSoleaferrea.id.11350 | UC | MRRAPS | 0.927(0.775,1.11) | 0.410 |
| genus.CandidatusSoleaferrea.id.11350 | UC | MRPRESSO | 0.936(0.803,1.091) | 0.416 |
| genus.Catenibacterium.id.2153 | UC | IVW | 0.866(0.735,1.022) | 0.089 |
| genus.Catenibacterium.id.2153 | UC | Weighted median | 0.832(0.669,1.035) | 0.099 |
| genus.Catenibacterium.id.2153 | UC | Weighted mode | 0.772(0.564,1.056) | 0.105 |
| genus.Catenibacterium.id.2153 | UC | MR-Robust | 0.865(0.754,0.993) | 0.039 |
| genus.Catenibacterium.id.2153 | UC | MR-Egger | 1.553(0.344,7.009) | 0.567 |
| genus.Catenibacterium.id.2153 | UC | MRRAPS | 0.863(0.719,1.036) | 0.114 |
| genus.Catenibacterium.id.2153 | UC | MRPRESSO | 0.866(0.754,0.996) | 0.115 |
| genus.ChristensenellaceaeR.7group.id.11283 | UC | IVW | 1.076(0.772,1.501) | 0.665 |
| genus.ChristensenellaceaeR.7group.id.11283 | UC | Weighted median | 1.176(0.808,1.71) | 0.397 |
| genus.ChristensenellaceaeR.7group.id.11283 | UC | Weighted mode | 1.18(0.692,2.013) | 0.543 |
| genus.ChristensenellaceaeR.7group.id.11283 | UC | MR-Robust | 1.136(0.816,1.581) | 0.450 |
| genus.ChristensenellaceaeR.7group.id.11283 | UC | MR-Egger | 1.012(0.363,2.819) | 0.981 |
| genus.ChristensenellaceaeR.7group.id.11283 | UC | MRRAPS | 1.128(0.823,1.545) | 0.454 |
| genus.ChristensenellaceaeR.7group.id.11283 | UC | MRPRESSO | 1.076(0.772,1.501) | 0.675 |
| genus.Clostridiumsensustricto1.id.1873 | UC | IVW | 0.741(0.552,0.995) | 0.046 |
| genus.Clostridiumsensustricto1.id.1873 | UC | Weighted median | 0.771(0.551,1.078) | 0.128 |
| genus.Clostridiumsensustricto1.id.1873 | UC | Weighted mode | 0.821(0.565,1.192) | 0.300 |
| genus.Clostridiumsensustricto1.id.1873 | UC | MR-Robust | 0.749(0.581,0.965) | 0.026 |
| genus.Clostridiumsensustricto1.id.1873 | UC | MR-Egger | 0.83(0.412,1.672) | 0.603 |
| genus.Clostridiumsensustricto1.id.1873 | UC | MRRAPS | 0.753(0.577,0.983) | 0.037 |
| genus.Clostridiumsensustricto1.id.1873 | UC | MRPRESSO | 0.741(0.552,0.995) | 0.094 |
| genus.Collinsella.id.815 | UC | IVW | 0.989(0.775,1.261) | 0.928 |
| genus.Collinsella.id.815 | UC | Weighted median | 1.035(0.764,1.403) | 0.824 |
| genus.Collinsella.id.815 | UC | Weighted mode | 1.15(0.711,1.86) | 0.569 |
| genus.Collinsella.id.815 | UC | MR-Robust | 0.994(0.788,1.254) | 0.962 |
| genus.Collinsella.id.815 | UC | MR-Egger | 0.996(0.387,2.567) | 0.994 |
| genus.Collinsella.id.815 | UC | MRRAPS | 0.989(0.756,1.292) | 0.934 |
| genus.Collinsella.id.815 | UC | MRPRESSO | 0.989(0.869,1.125) | 0.868 |
| genus.Coprobacter.id.949 | UC | IVW | 0.883(0.756,1.032) | 0.118 |
| genus.Coprobacter.id.949 | UC | Weighted median | 0.863(0.704,1.059) | 0.158 |
| genus.Coprobacter.id.949 | UC | Weighted mode | 0.857(0.636,1.153) | 0.308 |
| genus.Coprobacter.id.949 | UC | MR-Robust | 0.87(0.741,1.022) | 0.090 |
| genus.Coprobacter.id.949 | UC | MR-Egger | 0.942(0.556,1.596) | 0.824 |
| genus.Coprobacter.id.949 | UC | MRRAPS | 0.874(0.737,1.037) | 0.123 |
| genus.Coprobacter.id.949 | UC | MRPRESSO | 0.883(0.785,0.994) | 0.067 |
| genus.Coprococcus1.id.11301 | UC | IVW | 1.04(0.811,1.333) | 0.759 |
| genus.Coprococcus1.id.11301 | UC | Weighted median | 0.915(0.681,1.229) | 0.555 |
| genus.Coprococcus1.id.11301 | UC | Weighted mode | 0.886(0.615,1.277) | 0.517 |
| genus.Coprococcus1.id.11301 | UC | MR-Robust | 0.964(0.702,1.324) | 0.822 |
| genus.Coprococcus1.id.11301 | UC | MR-Egger | 0.901(0.485,1.674) | 0.741 |
| genus.Coprococcus1.id.11301 | UC | MRRAPS | 1.007(0.798,1.273) | 0.950 |
| genus.Coprococcus1.id.11301 | UC | MRPRESSO | 1.04(0.811,1.333) | 0.765 |
| genus.Coprococcus2.id.11302 | UC | IVW | 1.359(1.079,1.712) | 0.009 |
| genus.Coprococcus2.id.11302 | UC | Weighted median | 1.472(1.084,1.999) | 0.013 |
| genus.Coprococcus2.id.11302 | UC | Weighted mode | 1.56(0.936,2.602) | 0.088 |
| genus.Coprococcus2.id.11302 | UC | MR-Robust | 1.358(1.075,1.715) | 0.010 |
| genus.Coprococcus2.id.11302 | UC | MR-Egger | 1.006(0.225,4.492) | 0.993 |
| genus.Coprococcus2.id.11302 | UC | MRRAPS | 1.368(1.07,1.749) | 0.012 |
| genus.Coprococcus2.id.11302 | UC | MRPRESSO | 1.359(1.079,1.712) | 0.029 |
| genus.Coprococcus3.id.11303 | UC | IVW | 1.05(0.719,1.535) | 0.799 |
| genus.Coprococcus3.id.11303 | UC | Weighted median | 0.86(0.568,1.301) | 0.475 |
| genus.Coprococcus3.id.11303 | UC | Weighted mode | 0.811(0.383,1.716) | 0.584 |
| genus.Coprococcus3.id.11303 | UC | MR-Robust | 1.036(0.682,1.574) | 0.868 |
| genus.Coprococcus3.id.11303 | UC | MR-Egger | 2.587(0.6,11.149) | 0.202 |
| genus.Coprococcus3.id.11303 | UC | MRRAPS | 1.143(0.651,2.008) | 0.642 |
| genus.Coprococcus3.id.11303 | UC | MRPRESSO | 1.05(0.719,1.535) | 0.805 |
| genus.DefluviitaleaceaeUCG011.id.11287 | UC | IVW | 0.991(0.825,1.191) | 0.926 |
| genus.DefluviitaleaceaeUCG011.id.11287 | UC | Weighted median | 1(0.791,1.264) | 0.998 |
| genus.DefluviitaleaceaeUCG011.id.11287 | UC | Weighted mode | 1.019(0.699,1.484) | 0.923 |
| genus.DefluviitaleaceaeUCG011.id.11287 | UC | MR-Robust | 0.992(0.806,1.22) | 0.937 |
| genus.DefluviitaleaceaeUCG011.id.11287 | UC | MR-Egger | 1.254(0.65,2.422) | 0.499 |
| genus.DefluviitaleaceaeUCG011.id.11287 | UC | MRRAPS | 0.991(0.812,1.21) | 0.931 |
| genus.DefluviitaleaceaeUCG011.id.11287 | UC | MRPRESSO | 0.991(0.871,1.129) | 0.899 |
| genus.Desulfovibrio.id.3173 | UC | IVW | 0.989(0.824,1.188) | 0.906 |
| genus.Desulfovibrio.id.3173 | UC | Weighted median | 0.898(0.706,1.143) | 0.382 |
| genus.Desulfovibrio.id.3173 | UC | Weighted mode | 0.836(0.548,1.274) | 0.405 |
| genus.Desulfovibrio.id.3173 | UC | MR-Robust | 0.949(0.454,1.986) | 0.890 |
| genus.Desulfovibrio.id.3173 | UC | MR-Egger | 1.246(0.727,2.134) | 0.424 |
| genus.Desulfovibrio.id.3173 | UC | MRRAPS | 0.989(0.81,1.207) | 0.912 |
| genus.Desulfovibrio.id.3173 | UC | MRPRESSO | 0.989(0.872,1.122) | 0.868 |
| genus.Dialister.id.2183 | UC | IVW | 1.083(0.874,1.342) | 0.468 |
| genus.Dialister.id.2183 | UC | Weighted median | 1.033(0.772,1.384) | 0.825 |
| genus.Dialister.id.2183 | UC | Weighted mode | 1.002(0.654,1.535) | 0.993 |
| genus.Dialister.id.2183 | UC | MR-Robust | 1.02(0.75,1.387) | 0.900 |
| genus.Dialister.id.2183 | UC | MR-Egger | 1.262(0.476,3.345) | 0.640 |
| genus.Dialister.id.2183 | UC | MRRAPS | 1.056(0.837,1.332) | 0.648 |
| genus.Dialister.id.2183 | UC | MRPRESSO | 1.083(0.875,1.34) | 0.482 |
| genus.Dorea.id.1997 | UC | IVW | 1.004(0.789,1.279) | 0.973 |
| genus.Dorea.id.1997 | UC | Weighted median | 1.092(0.795,1.499) | 0.586 |
| genus.Dorea.id.1997 | UC | Weighted mode | 1.082(0.682,1.717) | 0.737 |
| genus.Dorea.id.1997 | UC | MR-Robust | 1.045(0.798,1.37) | 0.748 |
| genus.Dorea.id.1997 | UC | MR-Egger | 1.277(0.681,2.398) | 0.446 |
| genus.Dorea.id.1997 | UC | MRRAPS | 1.025(0.787,1.333) | 0.857 |
| genus.Dorea.id.1997 | UC | MRPRESSO | 1.004(0.829,1.216) | 0.967 |
| genus.Eggerthella.id.819 | UC | IVW | 1.026(0.881,1.195) | 0.739 |
| genus.Eggerthella.id.819 | UC | Weighted median | 1.038(0.85,1.269) | 0.714 |
| genus.Eggerthella.id.819 | UC | Weighted mode | 1.048(0.76,1.446) | 0.775 |
| genus.Eggerthella.id.819 | UC | MR-Robust | 1.031(0.855,1.242) | 0.750 |
| genus.Eggerthella.id.819 | UC | MR-Egger | 1.062(0.51,2.209) | 0.873 |
| genus.Eggerthella.id.819 | UC | MRRAPS | 1.027(0.87,1.211) | 0.755 |
| genus.Eggerthella.id.819 | UC | MRPRESSO | 1.026(0.901,1.169) | 0.706 |
| genus.Eisenbergiella.id.11304 | UC | IVW | 0.997(0.831,1.196) | 0.974 |
| genus.Eisenbergiella.id.11304 | UC | Weighted median | 1.034(0.829,1.29) | 0.768 |
| genus.Eisenbergiella.id.11304 | UC | Weighted mode | 1.084(0.734,1.599) | 0.685 |
| genus.Eisenbergiella.id.11304 | UC | MR-Robust | 0.998(0.825,1.207) | 0.986 |
| genus.Eisenbergiella.id.11304 | UC | MR-Egger | 1.519(0.346,6.668) | 0.580 |
| genus.Eisenbergiella.id.11304 | UC | MRRAPS | 0.994(0.82,1.205) | 0.953 |
| genus.Eisenbergiella.id.11304 | UC | MRPRESSO | 0.997(0.831,1.196) | 0.975 |
| genus.Enterorhabdus.id.820 | UC | IVW | 0.952(0.774,1.171) | 0.642 |
| genus.Enterorhabdus.id.820 | UC | Weighted median | 0.995(0.764,1.295) | 0.968 |
| genus.Enterorhabdus.id.820 | UC | Weighted mode | 1.035(0.744,1.441) | 0.839 |
| genus.Enterorhabdus.id.820 | UC | MR-Robust | 0.955(0.809,1.129) | 0.590 |
| genus.Enterorhabdus.id.820 | UC | MR-Egger | 1.266(0.732,2.19) | 0.398 |
| genus.Enterorhabdus.id.820 | UC | MRRAPS | 0.951(0.758,1.193) | 0.666 |
| genus.Enterorhabdus.id.820 | UC | MRPRESSO | 0.952(0.829,1.094) | 0.519 |
| genus.Erysipelatoclostridium.id.11381 | UC | IVW | 1.09(0.93,1.277) | 0.288 |
| genus.Erysipelatoclostridium.id.11381 | UC | Weighted median | 1.129(0.904,1.41) | 0.283 |
| genus.Erysipelatoclostridium.id.11381 | UC | Weighted mode | 1.112(0.759,1.63) | 0.586 |
| genus.Erysipelatoclostridium.id.11381 | UC | MR-Robust | 1.105(0.922,1.325) | 0.280 |
| genus.Erysipelatoclostridium.id.11381 | UC | MR-Egger | 0.498(0.262,0.945) | 0.033 |
| genus.Erysipelatoclostridium.id.11381 | UC | MRRAPS | 1.104(0.931,1.31) | 0.256 |
| genus.Erysipelatoclostridium.id.11381 | UC | MRPRESSO | 1.09(0.932,1.273) | 0.297 |
| genus.ErysipelotrichaceaeUCG003.id.11384 | UC | IVW | 1.021(0.85,1.225) | 0.825 |
| genus.ErysipelotrichaceaeUCG003.id.11384 | UC | Weighted median | 1.096(0.846,1.419) | 0.488 |
| genus.ErysipelotrichaceaeUCG003.id.11384 | UC | Weighted mode | 1.313(0.789,2.186) | 0.295 |
| genus.ErysipelotrichaceaeUCG003.id.11384 | UC | MR-Robust | 1.02(0.84,1.238) | 0.842 |
| genus.ErysipelotrichaceaeUCG003.id.11384 | UC | MR-Egger | 1.11(0.662,1.863) | 0.692 |
| genus.ErysipelotrichaceaeUCG003.id.11384 | UC | MRRAPS | 1.015(0.822,1.254) | 0.889 |
| genus.ErysipelotrichaceaeUCG003.id.11384 | UC | MRPRESSO | 1.021(0.85,1.225) | 0.828 |
| genus.Escherichia.Shigella.id.3504 | UC | IVW | 1.06(0.844,1.331) | 0.616 |
| genus.Escherichia.Shigella.id.3504 | UC | Weighted median | 1.056(0.779,1.432) | 0.725 |
| genus.Escherichia.Shigella.id.3504 | UC | Weighted mode | 1.048(0.615,1.784) | 0.864 |
| genus.Escherichia.Shigella.id.3504 | UC | MR-Robust | 1.061(0.849,1.325) | 0.604 |
| genus.Escherichia.Shigella.id.3504 | UC | MR-Egger | 1.12(0.552,2.271) | 0.753 |
[truncated: 401,845 more chars]
